# The Ground State Energy of a Two-Dimensional Bose Gas

**DOI:** 10.1007/s00220-023-04907-2

**Published:** 2024-02-23

**Authors:** Søren Fournais, Theotime Girardot, Lukas Junge, Leo Morin, Marco Olivieri

**Affiliations:** 1 Department of Mathematical Sciences, University of Copenhagen, Universitetsparken 5, Dk-2100 Copenhagen, OE Denmark; 2 Department of Mathematics, Aarhus University, Ny Munkegade 118, Aarhus C, 8000 Denmark

## Abstract

We prove the following formula for the ground state energy density of a dilute Bose gas with density $\rho$ in 2 dimensions in the thermodynamic limit

$$\begin{aligned} e^{\text {2D}}(\rho ) = 4\pi \rho ^2 Y\Big (1 - Y \vert \log Y \vert + \Big ( 2\Gamma + \frac{1}{2} + \log (\pi ) \Big ) Y \Big ) + o(\rho ^2 Y^{2}), \end{aligned}$$

as $\rho a^2 \rightarrow 0$. Here $Y= |\log (\rho a^2)|^{-1}$ and *a* is the scattering length of the two-body potential. This result in 2 dimensions corresponds to the famous Lee–Huang–Yang formula in 3 dimensions. The proof is valid for essentially all positive potentials with finite scattering length, in particular, it covers the crucial case of the hard core potential.

## Introduction

The calculation of the ground state energy of a dilute gas of bosons is of fundamental importance and has been the focus of much attention in recent years. This question can be posed in all dimensions of the ambient space, but of course, the most important case from the point of view of Physics is the 3-dimensional situation. However, also 1 and 2 dimensions are experimentally realizable. In this paper we study the 2-dimensional setting and prove an asymptotic formula analogous to the famous Lee–Huang–Yang formula in 3-dimensions.

Let us be more precise about the setting of the result. We consider positive, measurable potentials $v: {{\mathbb {R}}}^2 \rightarrow [0,+\infty ]$ that are radial. Given such a potential, we will let $a=a(v)$ be its scattering length (for details on the scattering length see Sect. 3) and define the Hamiltonian

1.1 $$\begin{aligned} H(N,L) = \sum _{j=1}^N -\Delta _j + \sum _{j<k} v(x_j-x_k), \end{aligned}$$

on $L^2(\Omega ^N)$, with $\Omega = [-\frac{L}{2}, \frac{L}{2}]^2$. The ground state energy density in the thermodynamic limit $e^{\text {2D}}(\rho )$ is then defined by

1.2 $$\begin{aligned} e^{\text {2D}}(\rho ) := \lim _{\begin{array}{c} L \rightarrow \infty \\ N/L^2 \rightarrow \rho \end{array}} L^{-2} \inf _{\Psi \in C_0^{\infty }(\Omega ^N)} \frac{ \langle \Psi , H(N,L) \Psi \rangle }{\Vert \Psi \Vert ^2}. \end{aligned}$$

It is a standard result that the limit exists, and actually our analysis of $e^{\text {2D}}(\rho )$ proceeds by giving upper bounds on the $\limsup$ and lower bounds on the $\liminf$. It is also well-known that the limit is independent of the boundary conditions. The fact that we consider $\Psi \in C_0^{\infty }$ in the formula above, corresponds to the choice of Dirichlet boundary conditions for concreteness.

### Theorem 1.1

(Main result). For any constants $C_0, \eta _0 >0$, there exist $C, \eta >0$ (depending only on $C_0$ and $\eta _0$) such that the following holds. If the (measurable) potential $v: {{\mathbb {R}}}^2 \rightarrow [0,+\infty ]$ is non-negative and radial with scattering length *a* and $\rho a^2 < C^{-1}$, and, furthermore,

1.3 $$\begin{aligned} v(x) \le \frac{C_0}{\vert x \vert ^{2}} \Big ( \frac{a}{\vert x \vert } \Big )^{\eta _0}, \qquad \text {for all } \vert x \vert \ge C_0 a . \end{aligned}$$

Then

1.4 $$\begin{aligned} \Big |e^{\text {2D}}(\rho ) - 4\pi \rho ^2 \delta _0 \Big (1 + \Big (2\Gamma + \frac{1}{2} + \log (\pi ) \Big ) \delta _0 \Big ) \Big |\le C \rho ^2 \delta ^{2+\eta }_0, \end{aligned}$$

with

1.5 $$\begin{aligned} \delta _0 := |\log (\rho a^2 |\log (\rho a^2)|^{-1})|^{-1}, \end{aligned}$$

where $\Gamma =0.577\ldots$ is the Euler–Mascheroni constant.

In terms of the simpler parameter $Y = |\log (\rho a^2)|^{-1}$, we get from (1.4), expanding $\delta _0$ in terms of *Y*, the three-term asymptotics

1.6 $$\begin{aligned} e^{\text {2D}}(\rho ) = 4\pi \rho ^2 Y\Big (1 - Y \vert \log Y \vert + \Big ( 2\Gamma + \frac{1}{2} + \log (\pi ) \Big ) Y \Big ) + {{\mathcal {O}}}(\rho ^2 Y^{2+\eta }). \end{aligned}$$

Here the third term in the asymptotics is analogous to the famous Lee–Huang–Yang term in the 3-dimensional situation.

Notice, in particular, that the decay assumption (1.3) is valid for potentials with compact support. So Theorem 1.1 applies to the very important special case of the hard core potential of radius *a*:

1.7 $$\begin{aligned} v_{\text {h}c}(x) = {\left\{ \begin{array}{ll} 0, &{} |x |> a,\\ +\infty , &{} |x |\le a. \end{array}\right. } \end{aligned}$$

For this potential the radius of the support is equal to its scattering length.

The proof of Theorem 1.1 will proceed by establishing upper and lower bounds. In Theorems 2.1 and 2.3 below, we will state more precisely the estimates for the upper and lower bounds, respectively, and the assumptions necessary for each of these. In Sect. 2 below, we will give an outline of the paper as well as these precise statements.

The first term $4\pi \rho ^2 Y$ in (1.6) was understood in [1] but a full proof was only given in 2001 in the paper [2]. Calculations beyond leading order were given in [3–6], but have so far not been rigorously proven. The recent papers [7, 8] give an analogous expansion of the ground state energy in the setting of the Gross–Pitaevskii regime, giving furthermore information about the excitation spectrum. The constant in the second order term was also found in [9] by restricting to quasi-free states in a special scaling regime.

In the 3-dimensional case, the asymptotic formula for the energy density (with $e^{\text {3D}}(\rho )$ defined analogously to (1.2) and *a* being here the 3-dimensional scattering length) is

1.8 $$\begin{aligned} e^{\text {3D}}(\rho ) = 4\pi a \rho ^2 \Big ( 1 + \frac{128}{15\sqrt{\pi }} \sqrt{\rho a^3} \Big ) + o( a \rho ^2\sqrt{\rho a^3} ). \end{aligned}$$

This is the famous Lee–Huang–Yang formula. The leading order term goes back to [10], and the second term—the Lee–Huang–Yang (LHY) term—were given in [11, 12]. Mathematically rigorous proofs of the leading order term were given in [13] (upper bound) and [14] (matching lower bound). Upper bounds for sufficiently regular potentials to the precision of the LHY-term were given in [15] (correct order only), [16] (first upper bound with correct constant on the LHY-term) with recent improvements in [17]. Lower bounds of second order were given in [18] (potentials in $L^1$) and [19] (general case including the hard core potential). The upper bound in 3-dimensions in the case of potentials with large $L^1$-norm, in particular the key example of hard core potentials, is still open.

As can be understood from this overview of results from the analysis of the 3*D* case, it is difficult to prove precise results on the energy when $\int _{{{\mathbb {R}}}^3} v$ is much larger than the scattering length *a*(*v*), i.e., the hard core case. In 2-dimensions the analogous comparison is between $\int _{{{\mathbb {R}}}^2} v$ and $\delta _0$, which always satisfy $\int _{{{\mathbb {R}}}^2} v \gg \delta _0$. So in 2-dimensions we face similar challenges as in the 3D hard core case, even for regular potentials. This is one of the reasons why progress on the 2D problem has been slower. It is therefore remarkable that Theorem 1.1 can be established, including both upper and lower bounds, without any extra assumptions on the potentials. Also, the 2*D* case comes with its own challenges due to the logaritmic divergences and changes of the lengthscales. In particular, the small parameter in 3*D* is $(\rho a^3)$, i.e. it is a power of the density parameter, whereas in the present 2*D* case, our small parameter is $Y = |\log (\rho a^2)|^{-1}$ which is logarithmic in the density.

Throughout the paper we will use the standard convention that $C>0$ will denote an arbitrarily large universal constant whose value can change from one line to the other.

***Notation*** We will use the following notation for Fourier transforms,

$$\begin{aligned} {\widehat{f}}(p) = {\widehat{f}}_p = \int e^{-ixp} f(x)\,\textrm{d}x. \end{aligned}$$

In the paper we will use the notation $A \ll B$ in a precise sense given by (H1).

## Strategies of the Proofs

### Upper bound

As upper bound we prove the following theorem.

#### Theorem 2.1

For any constants $C_0$, $\eta _0 >0$, there exists *C* (that depends only on $C_0$ and $\eta _0$) such that the following holds. Let $v : {\mathbb {R}}^2 \rightarrow [0,\infty ]$ be a non-negative, measurable and radial potential with scattering length $a<\infty$, and satisfying the following decay property,

2.1 $$\begin{aligned} v(x) \le \frac{C_0}{\vert x \vert ^{2}} \Big ( \frac{a}{\vert x \vert } \Big )^{\eta _0} \quad \text {for} \quad \vert x \vert \ge C_0 a . \end{aligned}$$

Then, if $$\rho a^2 < C^{-1}$$,

$$\begin{aligned} e^{\text {2D}}(\rho ) \le 4\pi \rho ^2 \delta _0 \Big (1 + \Big (2\Gamma + \frac{1}{2} + \log (\pi ) \Big ) \delta _0 \Big ) + C \rho ^2 \delta ^{3}_0\vert \log (\delta _0)\vert , \end{aligned}$$

with $\delta _0$ given by (1.5).

In order to prove Theorem 2.1, we will reduce the analysis to the case of compactly supported potentials on a smaller periodic box $\Lambda = \Lambda _\beta = [- \frac{L_{\beta }}{2},\frac{L_{\beta }}{2} ]^2$ with length

2.2 $$\begin{aligned} L_\beta = \rho ^{-1/2} Y^{-\beta }, \qquad \beta >0. \end{aligned}$$

In this box, if the density is $\rho$, the number of particles is $N = \rho L_{\beta }^2 = Y^{-2\beta }\gg 1$. Throughout the paper we find conditions on $\beta$ over which we will optimize. For a potential *v* with ${{\,\mathrm{\textrm{supp}}\,}}v \subseteq B(0,\frac{L_{\beta }}{2})$, we consider the following Hamiltonian acting on the Fock space ${\mathscr {F}}_s(L^2(\Lambda _\beta ))$,

2.3 $$\begin{aligned} {\mathcal {H}}_v = \bigoplus _{n \ge 0} \bigg ( \sum _{i=1}^n - \Delta _{x_i}^{\text {per}} + \sum _{1 \le i<j \le n} v^{\text {per}}(x_i-x_j) \bigg ) . \end{aligned}$$

Here $\Delta ^{\text {per}}$ is the periodic Laplacian, and $v^{\text {per}}(x) = \sum _{m \in {{\mathbb {Z}}}^2}v(x+L_{\beta }m)$ is the periodic version of *v*. Note that for any $p \in \frac{2\pi }{L_\beta } {\mathbb {Z}}^2$, the Fourier coefficient of $v^{\text {per}}$ is equal to the Fourier transform ${{\widehat{v}}} (p)$, because the radius of the support of *v* is smaller than $L_{\beta }$. In this setting we prove the following result.

#### Theorem 2.2

For any $\beta \ge \frac{3}{2}$, there exists $C >0$, depending only on $\beta$ such that the following holds. Let $\rho >0$ and $v : {\mathbb {R}}^2 \rightarrow [0,\infty ]$ be a non-negative, measurable and radial potential with scattering length *a* and ${{\,\mathrm{\textrm{supp}}\,}}v \subset B(0,R)$ for some $R >0$. If $\rho R^2 \le Y^{2 \beta + 2}$ and $\rho a^2 \le C^{-1}$, then there exists a normalized trial state $\Psi \in {\mathscr {F}}_s(L^2(\Lambda _\beta ))$, such that,

$$\begin{aligned} \langle {\mathcal {H}}_v \rangle _\Psi \le 4\pi L_\beta ^2 \rho ^2 \delta _0 \Big (1 + \Big (2\Gamma + \frac{1}{2} + \log (\pi ) \Big ) \delta _0 \Big ) + CL_{\beta }^2 \rho ^2 \delta ^{3}_0\vert \log (\delta _0)\vert . \end{aligned}$$

Moreover $\Psi$ satisfies $\langle {\mathcal {N}} \rangle _\Psi \ge N (1- C Y^2) ,$ and $\langle {\mathcal {N}}^2 \rangle _{\Psi } \le 9 N^2$, where ${\mathcal {N}}$ is the number operator on ${\mathscr {F}}_s(L^2(\Lambda _{\beta }))$ and $N=\rho L_\beta ^2= Y^{- 2 \beta }$.

#### Strategy for the upper bound

1. We will show in “Appendix A” how Theorem 2.1 follows from Theorem 2.2. This corresponds to go from the result on the box $\Lambda _{\beta }$ to the thermodynamic limit.
2. The rest of the proof, Sects. 4 and 5, is dedicated to the proof of Theorem 2.2. We first prove in Sect. 4 a weaker upper bound with the assumption that the potential is regular enough. We call it a *soft* potential. Under this assumption, we use a quasi-free trial state $\Phi$ built over a Weyl transform $W_{N_{0}}$ to create the condensate and a unitary $T_{\nu }$ to deal with the excitations. We then minimize over the parameters of this state. This is an adaptation of the method of [15, 20–22] to the 2D case. We show in Theorem 4.1 that, with a good choice of $\Phi$ to our level of precision, we have
  2.4 $$\begin{aligned} \langle {\mathcal {H}}_v \rangle _\Phi\le & {} 4\pi L_\beta ^2 \rho ^2 \delta _0 \Big (1 + \Big (2\Gamma + \frac{1}{2} + \log (\pi ) \Big ) \delta _0 \Big )\nonumber \\{} & {} + CL_{\beta }^2 \rho ^2 \delta _0({\widehat{v}}_0-{\widehat{g}}_0)+CL_{\beta }^2 \rho ^2 \delta ^{2}_0{\widehat{v}}_0. \end{aligned}$$
  Here $g = \varphi v$ and $\varphi$ is the scattering solution associated to *v* (see Sect. 3 for the precise definition of $\varphi$ and with parameter $\delta _0$). This provides a first upper bound, but it is not enough to prove Theorem 2.2, unless *v* admits a Fourier transform and ${\widehat{v}}_{0}$ is of order ${\widehat{g}}_{0}$.
3. In Sect. 5 we explain how to reduce from any *v* to a soft potential. To this end, we take care of the influence of the potential on a much shorter length scale by introducing $\varphi _b$ as the scattering solution normalized at
  2.5 $$\begin{aligned} b=\rho ^{-\frac{1}{2}}Y^{\beta +\frac{1}{2}} \end{aligned}$$
  and use it to build a *Jastrow function* as follows
  2.6 $$\begin{aligned} F_n(x_1, \ldots , x_n) = \prod _{1 \le i< j \le n} f(x_i-x_j), \end{aligned}$$
  with $f= \min (1,\varphi _b)$. Then our complete trial state will be the following product state
  2.7 $$\begin{aligned} \Psi = \bigoplus _{n \ge 1} F_n \Phi _n , \quad F_n , \Phi _n \in L^2_s(\Lambda _\beta ^n), \end{aligned}$$
  where $\Phi = \sum \Phi _n$ is a quasi-free state. When we compute the energy of such a state $\Psi$ we get
  2.8 $$\begin{aligned} \langle {\mathcal {H}}_v \rangle _\Psi \le \langle {\mathcal {H}}_{{\widetilde{v}}} \rangle _\Phi + \langle {\mathcal {R}} \rangle _\Phi , \end{aligned}$$
  where ${\mathcal {R}}$ is an error term and ${\widetilde{v}}$ is the following soft potential,
  2.9 $$\begin{aligned} {\widetilde{v}} = 2 f'(b) \delta _{\{\vert x \vert = b\}}. \end{aligned}$$
  The power of *Y* driven by the parameter $\beta$ in *b* is chosen minimal such that $\Vert \Psi \Vert ^{2}=\Vert \Phi \Vert ^{2}+O(Y^2)$, see Lemma 5.3. For the result to apply for the widest range of potentials we will want to choose $\beta$ as small as possible.
  The potential in (2.9) is soft in the sense that it has a decaying Fourier transform and $\widehat{\widetilde{v}}_0 \simeq \widehat{{\widetilde{g}}}_0$ (see Lemma 3.10 for precise estimates). Then we can take for $\Phi$ the optimal quasi-free state satisfying (2.4) for ${\widetilde{v}}$ and this turns out to be enough to prove Theorem 2.2.

#### Remarks

Since the Jastrow factor (2.6) encodes all 2-particle interactions—at least on short scales—it is a natural trial state for getting upper bounds on the energy. In particular, it has been used to get the correct first order upper bound, both in 3D [13] and 2D [2]. In the product state $\Psi$, the Jastrow factor deals with short distance correlations between particles (when $\vert x_i - x_j \vert \le b$), while long range effects are dealt with by the quasi-free state $\Phi$. In the case of hard core potentials, the Jastrow factor also imposes the necessary condition that our state vanishes whenever two particles are too close.

We emphasize the following major differences between 2D and 3D. To be able to reduce to the quasi-free state $\Phi$, we need to bound ${\mathcal {O}}(N^2)$ terms of the form $f(x_i-x_j)$ by 1. The number of particles *N* in our box is not too large (powers of $|\log (\rho a^2)|$, since the relevant length-scale is $\rho ^{-1/2}$ up to logarithmic factors) thus making this error controllable. This is not at all the case in dimension 3, because the number of particles in the box is of order $(\rho a^3)^{-2}$ (since the relevant length-scale in this case is $\frac{1}{\rho a^2}$). However, a similar state as ours was successfully used in the 3D Gross–Pitaevskii regime [22] (length-scale $\frac{1}{\sqrt{\rho a}}$). In this regime the number of particles is $(\rho a^3)^{-\frac{1}{2}}$, which allows the authors, with substantially more work, to get through to a good upper bound. More precisely, they use more accurate bounds on the Jastrow factor compared to our Sect. 5 and obtain the LHY order in the box. See Remark 5.1 for additional information.

Finally, one should notice that $\Phi$ is a quasi-free state, and does not include the soft pair interactions that were necessary in [16, 17] to get the correct upper bound in 3D. Indeed, for a quasi-free state $\Phi$ the second order energy bounds are in terms of ${\widehat{v}}_0$ and to get the correct constant one needs to change ${\widehat{v}}_0$’s into ${\widehat{g}}_0$’s. This is the role of soft pairs. However, our potential ${\widetilde{v}}$ from (2.9) already satisfies $\widehat{\widetilde{v}}_0 - \widehat{{\widetilde{g}}}_0 = {{\mathcal {O}}}(Y^2 \vert \log Y \vert )$ (see Lemma 3.10) and this replacement only gives errors of order $\rho ^2 Y^3 \vert \log Y \vert$. It is possible that we could add the soft pair interactions into $\Phi$ to reduce this error at the expense of a much longer and more technical proof.

We conclude this section by proving Theorem 2.1 using Theorem 2.2 and the classical theory of localization to smaller boxes which is added for convenience in “Appendix A”.

### Lower bound

In this section we provide the strategy of proof for the theorem below.

#### Theorem 2.3

For any constant $\eta _1 >0$ there exist $C, \eta >0$ (depending only on $\eta _1$) such that the following holds. Let $\rho >0$ and $v: {{\mathbb {R}}}^2 \rightarrow [0,+\infty ]$ be a non-negative, measurable and radial potential with scattering length $a < \infty$. If $\rho a^2 < C^{-1}$ and

2.10 $$\begin{aligned} \int _{ \{\vert x \vert > \rho ^{-1/2} \}} v(x) \log \Big ( \frac{\vert x \vert }{a} \Big ) ^2 \textrm{d}x \le Y^{\eta _1}, \end{aligned}$$

then

2.11 $$\begin{aligned} e^{\text {2D}}(\rho ) \ge 4\pi \rho ^2 \delta _0 \Big (1 + \Big (2\Gamma + \frac{1}{2} + \log (\pi ) \Big ) \delta _0 \Big ) + C \rho ^2 \delta ^{2+\eta }_0, \end{aligned}$$

with $\delta _0$ as defined in (1.5).

We introduce the lengths

2.12 $$\begin{aligned} \ell = \rho ^{-1/2} Y^{-1/2-\alpha }, \qquad \ell _{\delta _{0}} = \frac{1}{2} e^{\Gamma } \rho ^{-1/2} Y^{-1/2}, \end{aligned}$$

for a certain $\alpha \in (0,1)$, the second of which being called the *healing length*. The proof of Theorem 2.3 will depend on a precise choice of a number of parameters. For convenience these and the relations between them have been collected in “Appendix H”.

We work at three different lengthscales:

- the thermodynamical scale, in the box $\Omega = [-L/2, L/2]^2$, where we state the main result in the limit $L \rightarrow + \infty$;
- the large box scale $\Lambda = [-\ell /2, \ell /2]^2$, where we prove most of the results and by the sliding localization techniques we integrate over all these boxes to prove the lower bound in the whole thermodynamical box;
- the small box scale $B = [-d \ell /2, d \ell /2]^2$, with $d \ll 1$, where we derive a bound for the number of particles excited out from the condensate, fundamental for the general strategy, obtaining the Bose–Einstein condensation (BEC).

The relations

2.13 $$\begin{aligned} d \ell \ll \ell _{\delta _0}\ll \ell \ll L, \end{aligned}$$

guarantee that the boxes are in a chain of inclusions.

#### Strategy for the lower bound

The overall strategy for the lower bound has the same structure as in the 3*D* hard core case analyzed in [19]. Therefore, many of the steps below are the same as in that case. We will only indicate when a step differs from its 3*D* counterpart. However, the 2*D* case comes with its own challenges due to the logaritmic divergences and changes of the lengthscales.

1. In Sect. 6.1 we reformulate the problem in a grand canonical setting, adding a chemical potential $\rho _{\mu }$ to the Hamiltonian, in order to control the distribution of particles in later localization steps. The resulting Hamiltonian ${{\mathcal {H}}}_{\rho _{\mu }}$ acts on the symmetric Fock space ${{\mathcal {F}}}_{\textrm{s}}(L^2(\Omega ))$. We also reduce the analysis to compactly supported potentials with norm^1^$\Vert v\Vert _1 \le Y^{-1/8}$, using the analysis of the scattering equation from Sect. 3 (the details of this part are different from the 3*D* case). Theorem 2.3 is shown to be a consequence of Theorem 6.1.
2. In order to prove Theorem 6.1, in Sect. 6.2 we use a sliding localization technique to reduce the problem from the thermodynamical box $\Omega$ to the large box $\Lambda$. The result of this procedure is an inequality of the form (in the quadratic form sense)
  $$\begin{aligned} {{\mathcal {H}}}_{\rho _{\mu }} \ge \int _{{{\mathbb {R}}}^2} {{\mathcal {H}}}_{\Lambda _u}(\rho _{\mu }) \,du, \end{aligned}$$
  where ${{\mathcal {H}}}_{\Lambda _u}(\rho _{\mu })$ is a Hamiltonian localized to a box $\Lambda _u$ which is the translation of the fixed box $\Lambda$ to be centered at *u*. The main result is then reduced to the proof of an analogous lower bound for ${{\mathcal {H}}}_{\Lambda }(\rho _{\mu })$, namely Theorem 6.7. The next sections focus on this proof.
3. We split the potential energy on the large box in Sect. 7.1 by means of projectors *P* and *Q* onto and outside the condensate, respectively, or in other words onto the zero momentum sector and its complement. The splitting produces terms involving from 0 to 4 *Q* projectors. This is similar to the approach in [11, 12]. By an algebraic identity (see Lemma 7.1), we identify a positive term ${\mathcal {Q}}_4^{\text {ren}}$ that can be discarded for a lower bound. This procedure also changes the terms with 0 to 3 *Q*’s. By this procedure, all occurrences of the potential *v* are replaced by the function *g* related to the scattering equation and to the parameter $\delta _0$. This idea has its roots in [23] and was a key step in [18, 19]. Since ${{\widehat{g}}}(0)=8\pi \delta _0 \ll {{\widehat{v}}}(0)$, this can be interpreted as a renormalization procedure.
4. In [16] it was understood how the interaction of the so-called *soft pairs* contributes significantly to the energy. These correspond to two interacting high-momenta producing one 0-momentum and one low-momentum. This is the main contribution of the 3*Q* term. The soft pairs appear after estimating the other parts of the 3*Q* term to be of lower order. This is done in two steps, the first (restriction to low outgoing momentum) is proved in Sect. 7.2 and the second one (high incoming momenta) in Lemma 8.2, the latter being easier treated in second quantization.
5. A key step in both 2 and 3 dimensions is to be able to focus on states where the operator counting the number of excitations satisfies a norm bound. To handle the 3 dimensional hard-core case, in [19] it was realized that such a bound is only possible when restricting to excitations with low momentum. In the 2 dimensional case, we face this difficulty even if the potential *v* has small integral (i.e., it is soft). The reason for this difficulty is that the bound on the excitations involves the integral of *v*, i.e. ${\widehat{v}}(0)$. This has to be compared to the main term of the energy, where the relevant parameter is ${\widehat{g}}(0)$, and as previously noticed, in 2 dimensions ${{\widehat{g}}}(0)=8\pi \delta _0 \ll {{\widehat{v}}}(0)$. The solution to this problem follows the same general approach as in [19], namely to not bound all excitations but only those with low momentum. This is the result of Theorem 7.7. The analysis for this bound is carried out in Sect. 7.3 and based on estimates on Bose–Einstein Condensation from Theorem 7.6 proven in “Appendix D”. Some other important ingredients of the proof are delegated to “Appendix E”. Theorem 7.7 and its proof are somewhat simpler and more along the lines of an IMS-localization estimate than the ones in [19].
6. Section 8 contains lower bounds that use a second quantization formalism in momenta space. We first write the Hamiltonian in this formalism in Sect. 8.2. Then we use the *c*-number substitution in Sect. 8.3, thus reducing to a problem of minimization for particles outside the condensate. The operators related to the condensate act as numbers over the class of coherent states over which we minimize. After this procedure we arrive at an operator containing terms of order up to 3 creation and annihilation operators of non-zero momenta.
7. In Sect. 9 we distinguish the two cases where the density of particles in the condensate $\rho _z$ is far from or close to $\rho _{\mu }$, the expected density. Since we have Bose–Einstein condensation, we expect on physical grounds to be in the second case, and indeed fairly rough bounds suffice in the first case. These are given in Sect. 9.1. In the second case, $\rho _z \approx \rho _{\mu }$, a more careful analysis is needed. We use standard techniques, collected in “Appendix B”, to diagonalize the main quadratic part of the Hamiltonian the ground state energy of which appears as an integral. This integral is calculated in “Appendix C”, and we show how together with the constant term of the Hamiltonian, we get the energy to the desired precision. What remains at this point is to show how the remainders, including the localized 3*Q* term, are error terms, and this is the content of the technical Sect. 9.3. There we show how the contribution of the soft pairs is compensated by the remaining quadratic part of the Hamiltonian. Here in particular, the logarithmic divergencies specific to the 2-dimensional situation makes many estimates delicate and require extra localizations in momentum space.
8. Finally, in Sect. 9.4 we use all the previous results to give a proof of Theorem 6.7, with the choices of the parameters in “Appendix H”, where all the conditions used to prove the lower bound are collected.
9. In the proof we need two technical estimates, namely (8.12), (8.13) and (E1), which are taken from the 3D case and are independent of dimension. They are only stated and we refer to [19] for the proof.

## The Scattering Solution in 2 Dimensions

### Basic theory

In this section we establish the notation and results surrounding the two dimensional two body scattering problem. The standard properties of the scattering solutions stated below are well known and can be found in [24, Appendix A]. We will only consider radial and positive potentials $v:{\mathbb {R}}^2\rightarrow [0,\infty ]$, furthermore if *v* is compactly supported we denote by *R* the radius of the support of *v*, i.e., $v(x)=0$ if $\vert x\vert \ge R$.

#### Definition 3.1

For a compactly supported *v* its scattering length $a=a(v)$ is defined as

3.1 $$\begin{aligned} \frac{2\pi }{\log (\frac{\widetilde{R}}{a})}=\inf \Big \{\int _{B(0,\widetilde{R})}\Big (\vert \nabla u \vert ^2+\frac{1}{2}v \vert u \vert ^2 \Big )\textrm{d}x\quad \Big \vert \quad u \in H^1(B(0,\widetilde{R})),\quad u \vert _{\partial B(0,\widetilde{R})}=1\Big \},\nonumber \\ \end{aligned}$$

where $\widetilde{R}> R$ is arbitrary.

By the positivity of the right hand side we find $a\le R$. It is also easy to verify that *a* is an increasing function of *v* and is independent of $\widetilde{R}>R$. Furthermore for any $\widetilde{R}$ the above functional has a unique minimizer $\varphi _{v,\widetilde{R}}=\log (\frac{\widetilde{R}}{a(v)})^{-1}\varphi _v^{(0)}(x)$, where, for $v\in L^1({\mathbb {R}}^2)$, we have

3.2 $$\begin{aligned} -\Delta \varphi ^{(0)}_v+\frac{1}{2}v\varphi ^{(0)}_v =0 \quad \hbox { on}\ {\mathbb {R}}^2, \end{aligned}$$

in the distributional sense. Furthermore,

$$\begin{aligned}\varphi ^{(0)}_v(r)=\log \Big (\frac{r}{a(v)}\Big ), \quad \hbox { for}\ r\ge R,\end{aligned}$$

and $\varphi ^{(0)}_v$ is a monotone, non-decreasing and non-negative, radial function. We will omit the *v* in the notation of the scattering length if the potential is clear from the context.

The logarithm in the 2*D*-scattering solution is clearly unbounded for large values of *r*. This is a major difference to the 3*D* behaviour (where the scattering solution behaves as $1-\frac{a}{r}$ at infinity). Therefore the scattering solution normalized to 1 at a certain length $\widetilde{R}$ is of much greater importance. Using the parameter

3.3 $$\begin{aligned} \delta =\frac{1}{2}\log \Big (\frac{\widetilde{R}}{a}\Big )^{-1},\qquad \text {i.e.} \qquad \widetilde{R}=ae^{\frac{1}{2\delta }}, \end{aligned}$$

we define on ${\mathbb {R}}^2$

3.4 $$\begin{aligned} \varphi =\varphi _{v,\delta } = 2 \delta \varphi ^{(0)}, \qquad \omega = 1- \varphi , \qquad g = v \varphi =v(1-\omega ). \end{aligned}$$

Clearly,

3.5 $$\begin{aligned} -\Delta \omega = \frac{1}{2} g, \end{aligned}$$

and, using the divergence theorem,

3.6 $$\begin{aligned} \int g \,\textrm{d}x = 8\pi \delta . \end{aligned}$$

We remark here again a difference between the 2*D* and 3*D* case: in 3*D*, $\varphi$ would be normalized to 1 at infinity and (3.6) would have an *a* instead of $\delta$.

#### Remark 3.2

(On the parameters $\delta$ and ${\widetilde{R}}$) We clearly have some freedom in the choice of $\delta$, which amounts to determine a normalization lengthscale ${\widetilde{R}}$ for $\varphi$. Throughout the paper, we will need $\delta$ to be of the same order as $Y = \vert \log (\rho a^2) \vert ^{-1}$, namely

3.7 $$\begin{aligned} \frac{Y}{2}\le \delta \le 2 Y, \qquad \text {or, equivalently, } \quad (\rho a^2)^{-1/4} \le \frac{{\widetilde{R}}}{a} \le (\rho a^2)^{-1}. \end{aligned}$$

With this condition we can always exchange *Y* and $\delta$ when estimating errors. We thus get upper and lower bounds on the energy depending on the parameter $\delta$. In both cases, it turns out that the optimal choice is given by (1.5), i.e.

3.8 $$\begin{aligned} \delta = \delta _0 = |\log (\rho a^2 |\log (\rho a^2)|^{-1})|^{-1}, \end{aligned}$$

which corresponds to

3.9 $$\begin{aligned} \frac{{\widetilde{R}}}{a} = (\rho a^2 Y)^{-1/2}. \end{aligned}$$

See also Remarks 4.9 and C.4.

### Potentials without compact support

#### Definition 3.3

For a potential *v* without compact support the scattering length is defined as

$$\begin{aligned}a(v)=\lim _{n\rightarrow \infty }a(v\mathbb {1}_{B(0,n)}).\end{aligned}$$

Since *a* is an increasing function of *v* the limit exits if and only if $\{a(v\mathbb {1}_{B(0,n)})\}_n$ is bounded, which by [25, Lemma 1] is true if and only if there exists a $\widetilde{b}>0$ such that

$$\begin{aligned}\int _{\{\vert x\vert >\widetilde{b}\}}v(x)\log \Big (\frac{\vert x\vert }{\widetilde{b}}\Big )^2 \textrm{d}x<\infty .\end{aligned}$$

We need to localize our potentials to have compact support. The next result estimates the change this localization induces in the scattering length.

#### Lemma 3.4

For a potential *v* with finite scattering length *a* and $R>a$, let $v_R=\mathbb {1}_{B(0,R)}v$ and $a_R$ be its associated scattering length. Then,

3.10 $$\begin{aligned} 0\le \frac{2\pi }{\log (\frac{R}{a})}-\frac{2\pi }{\log (\frac{{R}}{a_R})}\le \frac{1}{2} \int _{\{\vert x\vert > R\}}v(x)\frac{\log (\frac{\vert x\vert }{a})^2}{\log (\frac{R}{a})^2}\textrm{d}x. \end{aligned}$$

#### Proof

Let $\varphi _1$ be the scattering solution for $v_R$ normalized at *R*, and let

3.11 $$\begin{aligned} \varphi _n(x):={\left\{ \begin{array}{ll} \varphi _1(x)\frac{\log (\frac{R}{a})}{\log (n)}, &{}\vert x\vert \le R,\\ \frac{\log (\frac{\vert x\vert }{a})}{\log (n)}, &{}\vert x\vert \ge R. \end{array}\right. } \end{aligned}$$

Notice that $\varphi _n$ is normalized at $a\cdot n$ and continuous. We use it as a trial function in the variational problem of $v_n=\mathbb {1}_{B(0,a\cdot n)}v$, with $n\cdot a>R$, to get (with $a_n:=a(v_n)$)

3.12 $$\begin{aligned} \frac{2\pi }{\log (\frac{a\cdot n}{a_n})}\le \int _{\{\vert x\vert< R\}}\Big ( \vert \nabla \varphi _n\vert ^2+\frac{1}{2}v_n\varphi _n^2\Big ) \textrm{d}x+\int _{\{R<\vert x\vert < a\cdot n\}}\Big ( \vert \nabla \varphi _n\vert ^2+\frac{1}{2}v_n\varphi _n^2\Big ) \textrm{d}x.\nonumber \\ \end{aligned}$$

Since $\varphi _n$ is just a multiple of the scattering solution of $\varphi _1$ inside *R* the first integral gives

3.13 $$\begin{aligned} \int _{\{\vert x\vert < R\}} \Big (\vert \nabla \varphi _n\vert ^2+\frac{1}{2}v_n\varphi _n^2 \Big )\textrm{d}x=\frac{2\pi }{\log (\frac{R}{a_R})}\frac{\log (\frac{R}{a})^2}{\log (n)^2}. \end{aligned}$$

The second term is directly calculated using the explicit formula for $\varphi _n$,

3.14 $$\begin{aligned}{} & {} \int _{\{R<\vert x\vert < a\cdot n\}}\Big (\vert \nabla \varphi _n\vert ^2+\frac{1}{2}v_n\varphi _n^2\Big ) \textrm{d}x \nonumber \\{} & {} \quad \le \frac{2\pi \log (\frac{a\cdot n}{R})}{\log (n)^2}+\frac{1}{2\log (n)^2}\int _{\{\vert x\vert >R\}}v\log \Big (\frac{\vert x\vert }{a}\Big )^2. \end{aligned}$$

By (3.13), multiplying (3.12) through with $\log (n)^2$ and letting $n\rightarrow \infty$, whereby $a_n\rightarrow a$, yields

$$\begin{aligned}2\pi \log \Big (\frac{R}{a}\Big )\le \frac{2\pi \log (\frac{R}{a})^2}{\log (\frac{R}{a_R})}+\frac{1}{2}\int _{\{\vert x\vert >R\}}v\log \Big (\frac{\vert x\vert }{a}\Big )^2\textrm{d}x.\end{aligned}$$

The result then follows by dividing through with $\log (\frac{R}{a})^2$. $\square$

### Compactly supported potentials with large integrals

We state and prove here in the 2*D* setting a similar approximation result as the one found in [19, Theorem 1.6] for the scattering length in 3*D*.

#### Lemma 3.5

For a radial, positive $v\in L^1({\mathbb {R}}^2)$ with support contained in *B*(0, *R*) there exists, for any $T>0$, a $v_T:{\mathbb {R}}^2\rightarrow [0,+\infty ]$ satisfying

3.15 $$\begin{aligned} 0\le v_T(x)\le v(x), \quad \text { for all } x\in {\mathbb {R}}^2, \text { and }\qquad \int v_T\le 4 \pi T,\ \end{aligned}$$

and such that

3.16 $$\begin{aligned} \frac{2\pi }{\log (\frac{R}{a})}-\frac{2\pi }{\log (\frac{R}{a_T})}\le \frac{2\pi }{\log (\frac{R}{a})^2T}. \end{aligned}$$

#### Proof

Due to the integrability assumption on *v* we may define

$$\begin{aligned}R_T=\inf \Big \{R'>0: \int _{\{\vert x\vert \ge R'\}}v\textrm{d}x< 4 \pi T\Big \}\end{aligned}$$

and

3.17 $$\begin{aligned} v_T:=v\mathbb {1}_{\{\vert x\vert >R_T\}}. \end{aligned}$$

Clearly,

3.18 $$\begin{aligned} \int v_T=4 \pi T. \end{aligned}$$

Also, we may assume $R_T>0$. Otherwise there is nothing to prove.

Let $\varphi$ be the scattering solution of *v* and $\varphi _T$ the scattering solution of $v_T$ both normalized at $\widetilde{R}>R$. We have from (3.6), using that $\varphi _T$ is a non-decreasing function,

$$\begin{aligned}\frac{4\pi }{\log (\frac{\widetilde{R}}{a_T})}=\int v_T\varphi _T\textrm{d}x\ge \varphi _T(R_T)\int v_T=4\pi \varphi (R_T)T,\end{aligned}$$

and hence

3.19 $$\begin{aligned} \varphi (R_T)\le \frac{1}{\log (\frac{\widetilde{R}}{a_T})T}. \end{aligned}$$

Next we define

$$\begin{aligned}u=\mathbb {1}_{\{\vert x\vert >R_T\}}(\varphi _T-\omega _T\varphi _T(R_T))\qquad \text {where}\quad \omega _T(x)=1-\frac{\log (\frac{\vert x\vert }{R_T})}{\log (\frac{\widetilde{R}}{R_T})}.\end{aligned}$$

Observe that $u(\widetilde{R})=1$ and we may therefore apply it as a trial function in the functional for *a* to get

3.20 $$\begin{aligned} \frac{2\pi }{\log (\frac{\widetilde{R}}{a})}\le \int _{\{\vert x\vert <\widetilde{R}\}}\Big (\vert \nabla u\vert ^2+\frac{1}{2}vu^2\Big )\textrm{d}x:=E_1+E_2+E_3, \end{aligned}$$

with

$$\begin{aligned} \begin{aligned}&E_1=\int _{\{R_T<\vert x\vert<\widetilde{R}\} } \Big (\vert \nabla \varphi _T\vert ^2+ \frac{1}{2}v_T\varphi _T^2 \Big ) \textrm{d}x=\frac{2\pi }{\log (\frac{\widetilde{R}}{a_T})},\\&E_2=-2\varphi _T(R_T)\int _{\{R_T<\vert x\vert<\widetilde{R}\}} \Big (\nabla \varphi _T\nabla \omega _T+ \frac{1}{2}v_T\varphi _T\omega _T \Big ) \textrm{d}x=0,\\&E_3=\varphi _T(R_T)^2\int _{\{R_T<\vert x\vert <\widetilde{R}\}} \Big (\vert \nabla \omega _T \vert ^2+ \frac{1}{2}v_T\omega _T^2 \Big )\textrm{d}x. \end{aligned} \end{aligned}$$

For $E_2$ we integrated by parts and used that $\varphi _T$ is harmonic inside $B(0,R_T)$, thus constant, which makes the boundary term vanish. For $E_3$ we use that $\omega _T\le 1$ on the given interval, so combining (3.18), (3.19) and (3.20) yields

3.21 $$\begin{aligned} \frac{2\pi }{\log (\frac{\widetilde{R}}{a})}-\frac{2\pi }{\log (\frac{\widetilde{R}}{a_T})}\le E_3\le \frac{2\pi }{\log (\frac{\widetilde{R}}{a_T})^2}\bigg ( \frac{1}{\log (\frac{\widetilde{R}}{R_T})T^2}+\frac{1}{T}\bigg ). \end{aligned}$$

Using that $a\ge a_T$ we may replace $a_T$ with *a* on the right hand side. Secondly, we observe that the function

$$\begin{aligned}\bigg (\frac{1}{\log (\frac{\widetilde{R}}{a})}-\frac{1}{\log (\frac{\widetilde{R}}{a_T})}\bigg )\log \Big (\frac{\widetilde{R}}{a}\Big )^2,\end{aligned}$$

is increasing in $\widetilde{R}$ so we may replace $\widetilde{R}$ with *R* in the above expression and use (3.21) to get

$$\begin{aligned} \frac{2\pi }{\log (\frac{R}{a})}-\frac{2\pi }{\log (\frac{R}{a_T})}\le \frac{2\pi }{\log (\frac{R}{a})^2}\bigg ( \frac{1}{\log (\frac{\widetilde{R}}{R_T})T^2}+\frac{1}{T}\bigg ). \end{aligned}$$

Now the result follows by letting $\widetilde{R}$ go to infinity. $\square$

We are ready to prove the main theorem of this section which gives us the ability to deal with a wide range of potentials including, most notably, the hard core.

#### Theorem 3.6

For a radial, positive potential $v:{\mathbb {R}}^2\rightarrow [0,\infty ]$ with finite scattering length *a* there exists, for any $R>a$ and $T,\epsilon >0$, a potential $v_{T,R,\epsilon }$ such that

3.22 $$\begin{aligned} {{\,\mathrm{\textrm{supp}}\,}}(v_{T,R,\epsilon })\subset B(0,R),\qquad 0\le v_{T,R,\epsilon }(x)\le v(x),\qquad \int v_{T,R,\epsilon }\le 4\pi T, \end{aligned}$$

and its scattering length $a_{T,R,\epsilon }$ satisfies

3.23 $$\begin{aligned} \frac{2\pi }{\log (\frac{R}{a})}-\frac{2\pi }{\log (\frac{R}{a_{T,R,\epsilon }})}\le \frac{1}{\log (\frac{R}{a})^2}\bigg (\frac{2\pi }{T}(1+\epsilon )+ \frac{1}{2} \int _{\{\vert x\vert > R\}}v(x)\log \Big (\frac{\vert x\vert }{a}\Big )^2\bigg ) \textrm{d}x.\nonumber \\ \end{aligned}$$

#### Proof

Lemma 3.5 applied to $v_R^n=\mathbb {1}_{B(0,R)}\min (n,v)$ yields a $v_{R,T}^n$ satisfying all three conditions of (3.22) and

3.24 $$\begin{aligned} \frac{2\pi }{\log (\frac{R}{a_R^n})}-\frac{2\pi }{\log (\frac{R}{a_{T,R}^n})}\le \frac{2\pi }{\log (\frac{R}{a})^2T}, \end{aligned}$$

for all $n\in {\mathbb {N}}$. In the above we used that $a_{T,R}^n\le a_R^n\le a_R\le a$ (where $a_{T,R}^n, a_R^n, a_R$ are the scattering lengths of $v_{T,R}^n, v_{R}^n, v_R$, respectively). Choosing $n_0$ large enough such that $a_R^{n_0}$ is close enough to $a_R$ gives an $a_{T,R,\epsilon }:=a_{T,R}^{n_0}$ satisfying

3.25 $$\begin{aligned} \frac{2\pi }{\log (\frac{R}{a_R})}-\frac{2\pi }{\log (\frac{R}{a_{T,R,\epsilon }})}\le \frac{2\pi }{\log (\frac{R}{a})^2T}(1+\epsilon ). \end{aligned}$$

We conclude using (3.10) which gives the integral term of (3.23). $\square$

### Fourier analysis on the scattering equation

Due to Theorem 3.6 we may assume our potentials to be compactly supported and $L^1$, thus making the Fourier transform well defined. The scattering solution $\varphi$ will be the one defined in (3.4) which is normalized to 1 outside the support of *v*. In order to discuss the Fourier transform of the scattering solution, we recall some standard results surrounding the Fourier transform of the logarithm. We denote by ${\mathcal {S}}$ and ${\mathcal {S}}'$ the Schwartz space and the space of tempered distribution on ${\mathbb {R}}^2$, respectively.

#### Lemma 3.7

For $D>0$, let $L_D$ denote the tempered distribution given by the function $\log (|x |/D)$ in ${{\mathbb {R}}}^2$. The Fourier transform of $L_D$ satisfies for any $h\in {\mathcal {S}}$

3.26 $$\begin{aligned} \langle \widehat{L_D} , h \rangle _{{{\mathcal {S}}}', {{\mathcal {S}}}} =-(2\pi ) \int _{{{\mathbb {R}}}^2} \frac{h(p)-h(0) \mathbb {1}_{\{|p |\le 2 e^{-\Gamma }D^{-1}\}}}{p^2}\,\textrm{d}p, \end{aligned}$$

where $\Gamma$ denotes the Euler–Mascheroni constant,

3.27 $$\begin{aligned} \Gamma := - \int _0^{\infty } e^{-x} \log x\, \textrm{d}x \approx 0.5772. \end{aligned}$$

The proof is an exercise in distribution theory, with details for instance given in the recent book [26, Theorem 4.73].

It follows from (3.26) that, for any $f \in {\mathcal {S}}$,

3.28 $$\begin{aligned}&\iint _{{{\mathbb {R}}}^2 \times {{\mathbb {R}}}^2} \overline{f(x)} f(y) \log \Big (\frac{|x-y|}{D}\Big )\, \textrm{d}x \textrm{d}y\nonumber \\ {}&\quad = -2\pi \int _{{{\mathbb {R}}}^2} \frac{|{\widehat{f}}(p)|^2-|{\widehat{f}}(0)|^2 \mathbb {1}_{\{|p |\le 2 e^{-\Gamma }D^{-1}\}}}{p^2}\, \textrm{d}p. \end{aligned}$$

Using the notation from (3.4) and (3.5), we may compute the Fourier transform of $\omega$. In the 3*D* case one gets that ${\widehat{\omega }}(p) = \frac{{\widehat{g}}(p)}{2p^2}$, but in 2*D* this formula has to be corrected by a distribution supported at the origin according to Lemma 3.7, see Lemma 3.8 below.

#### Lemma 3.8

Let ${\widehat{\omega }}$ denote the Fourier transform of $\omega$. Then ${\widehat{\omega }}$ is the tempered distribution given by

3.29 $$\begin{aligned} \langle {\widehat{\omega }},u\rangle _{{{\mathcal {S}}}', {{\mathcal {S}}}} = \int \frac{{\widehat{g}}(p) u(p) - {\widehat{g}}(0) u(0) \mathbb {1}_{\{|p |\le \ell _{\delta }^{-1}\}} }{2p^2} \textrm{d}p,\end{aligned}$$

for any $u\in {\mathcal {S}}$ where, recalling the definition of $\widetilde{R}$ in (3.3),

3.30 $$\begin{aligned} \ell _{\delta }:= \frac{a e^{\Gamma }}{2} e^{\frac{1}{2\delta }} =\frac{1}{2}e^\Gamma \widetilde{R}. \end{aligned}$$

Notice that if $\delta =\delta _0$ from (1.5), then $\ell _{\delta }$ coincides with $\ell _{\delta _0}$ introduced in (2.12).

#### Proof

We first recall the definition (3.4) of $\omega$ and write

3.31 $$\begin{aligned} \omega = - \frac{{{\widehat{g}}}(0)}{4\pi } \log \Big (\frac{r}{\tilde{R}}\Big ) + {\widetilde{\omega }}, \end{aligned}$$

where ${\widetilde{\omega }}$ is compactly supported, and we recall ${{\widehat{g}}}(0) = 8 \pi \delta$. Hence, using the Fourier transform of the logarithm as recalled in Lemma 3.7,

3.32 $$\begin{aligned} \langle {\widehat{\omega }},u\rangle _{{{\mathcal {S}}}', {{\mathcal {S}}}} = {{\widehat{g}}}(0) \int _{{{\mathbb {R}}}^2} \frac{u(p)-u(0) \mathbb {1}_{\{|p |\le 2 e^{-\Gamma }{\tilde{R}}^{-1}\}}}{2p^2}\, \textrm{d}p + \int \widehat{{\widetilde{\omega }}}(p) u(p)\, \textrm{d}p. \end{aligned}$$

Using the scattering equation (3.5) we find $\widehat{g}(p) = 2p^2 {{\widehat{\omega }}} (p) = {{\widehat{g}}}(0) + 2p^2 \widehat{{\widetilde{\omega }}}(p)$, where we used that the logarithm is the fundamental solution of the Laplacian. Since $\widehat{{\widetilde{\omega }}}$ is a smooth function we deduce

$$\begin{aligned}\widehat{{\widetilde{\omega }}}(p) = \frac{{{\widehat{g}}}(p) - {{\widehat{g}}}(0)}{2p^2},\end{aligned}$$

and this concludes the proof. $\square$

Thanks to the previous lemma we are able to prove some important properties of $\widehat{g\omega }(0)$ which are going to be key through all the paper.

#### Lemma 3.9

The following identity holds

3.33 $$\begin{aligned} \widehat{g\omega }(0) = \int _{{\mathbb {R}}^2} \frac{{\widehat{g}}(k)^2 - {\widehat{g}}(0)^2 \mathbb {1}_{\{|k|\le \ell ^{-1}_{\delta }\}}}{2 k^2}\, \textrm{d}k \end{aligned}$$

and, furthermore, the following bounds hold

3.34 $$\begin{aligned} |\widehat{g\omega }(0) |&\le C \delta , \end{aligned}$$

3.35 $$\begin{aligned} \bigg \vert \int _{\{|k|\le \ell _{\delta }^{-1}\}} \frac{{\widehat{g}}(k)^2 - {\widehat{g}}(0)^2}{2 k^2}\, \textrm{d}k\bigg \vert&\le C R^2 \delta ^2 \ell ^{-2}_{\delta }, \end{aligned}$$

3.36 $$\begin{aligned} \bigg \vert \int _{\{|k |\ge \ell _{\delta }^{-1}\}} \frac{{\widehat{g}}(k)^2}{2 k^2}\,\textrm{d}k\bigg \vert&\le C \delta . \end{aligned}$$

#### Proof

Formula (3.33) is formally given by an application of Lemma 3.8 choosing $u = {\widehat{g}}$. Since ${\widehat{g}}$ is not a Schwartz function, we need to apply a regularization argument, by truncating in momentum space. This truncation can then be removed at the end and one arrives at (3.33).

The first bound (3.34) follows because in the support of *g*, $\omega \le 1$ and ${\widehat{g}}_0 = 8\pi \delta$. The last bound (3.36) follows once we have proved the second one. In order to do that, we consider a Taylor expansion to the second order of $\vert {\widehat{g}}_{k}-{\widehat{g}}_{0}\vert \le C\vert k\vert ^{2}\Vert {\widehat{g}}_{k}''\Vert _{\infty }$ as a radial function, due to the symmetry of *g*. We use that *v* has a compact support *R* and the definition

3.37 $$\begin{aligned} {\widehat{g}}_{k}=\int _{{\mathbb {R}}^{2}}v\varphi e^{-ik.x}\textrm{d}x \end{aligned}$$

to bound ${\widehat{g}}_{k}''$ by $R^{2}{\widehat{g}}_{0}$ to obtain

3.38 $$\begin{aligned} \bigg \vert \int _{\{|k |\le \ell _{\delta }^{-1}\}} \frac{{\widehat{g}}_k^2 - {\widehat{g}}_0^2}{2 k^2}\,\textrm{d}k\bigg \vert \le CR^2 |{\widehat{g}}_0|^2 \int _{\{|k |\le \ell _{\delta }^{-1}\}} \textrm{d}k = CR^2 \delta ^2 \ell ^{-2}_{\delta }. \end{aligned}$$

$$\square $$

### Spherical measure potentials

For the upper bound, we will change the potential in order to ensure small $L^1$ norm. For a potential *v* supported in *B*(0, *R*) and $b>R$, let

$$\begin{aligned}f(x):=\min \Big (1,\varphi ^{(0)}(x)\log \Big (\frac{b}{a}\Big )^{-1} \Big ).\end{aligned}$$

Thus, *f* is the scattering solution in *B*(0, *b*) normalized at *b* and extended by one. The new potential $\widetilde{v}$ will then be described by the deviation of *f* being the actual scattering solution, i.e.,

3.39 $$\begin{aligned} \widetilde{v}=2\Big (-\Delta f+\frac{1}{2}vf\Big ), \end{aligned}$$

where the above equality is to be thought of in a distributional sense. The factor 2 is important and should be thought of as the number of particles involved in the scattering process. A quick calculation shows that

3.40 $$\begin{aligned} \widetilde{v}=2f'(b)\delta _{\{\vert x\vert =b\}}=2\frac{1}{b\log (\frac{b}{a})}\delta _{\{\vert x\vert =b\}}, \end{aligned}$$

where $\delta _{\{\vert x\vert =b\}}$ is the uniform measure on the circle $\{\vert x \vert =b\}$ normalized so that $\int \delta _{\{\vert x\vert =b\}} = 2\pi b$, and where $f'(b)$ is to be understood as the radial derivative (from the left) of *f* at length *b*. We show in Sect. 5 how we reduce to this potential. The simple, but essential properties of $\widetilde{v}$ are stated in the lemma below.

#### Lemma 3.10

Let *v* and $\widetilde{v}$ be given as above. We use the notation $\widetilde{a}=a(\widetilde{v})$ and $a=a(v)$. Furthermore, let ${\widetilde{\varphi }}$ be the scattering solution of $\widetilde{v}$ normalized at $\widetilde{R}>b$ and $\widetilde{g} = \widetilde{v} {\widetilde{\varphi }}$. Then

1. The scattering lengths agree, i.e., $\widetilde{a}=a$.
2. $\widehat{ {\widetilde{v}}}(p)=2f'(b) b J_0(b\vert p\vert )$, where $J_0$ is the zeroth spherical Bessel function. In particular there exists a universal constant $C>0$ such that
  3.41 $$\begin{aligned} \vert \widehat{ \widetilde{v} }(p)\vert \le C \frac{\widehat{{\widetilde{v}}}(0)}{\sqrt{b\vert p\vert }}. \end{aligned}$$
3. $$\displaystyle \widehat{ {\widetilde{v}}}(0):=\langle v,1\rangle = \frac{4 \pi }{\log (b / a)}$$, and $$\displaystyle \widehat{\widetilde{g}}(0)=\widehat{ {\widetilde{v}} {{\widetilde{\varphi }}}}(0) = \frac{4\pi }{\log (\widetilde{R}/a)}.$$

#### Proof

The potential $\widetilde{v}$ is a spherical measure on the sphere $\{\vert x\vert =b\}$ and thus ${\widetilde{\varphi }}$ is harmonic both inside and outside this sphere. We may therefore conclude from the continuity of ${\widetilde{\varphi }}$ that

3.42 $$\begin{aligned} \log (\widetilde{R} / {\widetilde{a}}) {\widetilde{\varphi }}\left( r\right) = {\left\{ \begin{array}{ll} \log (b/{\widetilde{a}}), &{} \text{ if } r\le b, \\ \log (r/ {\widetilde{a}}), &{} \text{ if } r > b. \end{array}\right. } \end{aligned}$$

From the scattering equation

$$\begin{aligned}-\Delta {\widetilde{\varphi }}+\frac{1}{2}\widetilde{v}{\widetilde{\varphi }}=0,\end{aligned}$$

applied to a $u\in C_{c}^{\infty }({\mathbb {R}}^{2})$ we obtain, using Green’s formula,

3.43 $$\begin{aligned} -\int _{\{\vert x\vert =b\}}u\nabla {\widetilde{\varphi }}\cdot \textrm{d}\textbf{n}=f'(b){\widetilde{\varphi }}(b)\int _{\{\vert x\vert =b\}}u \end{aligned}$$

and then deduce

3.44 $$\begin{aligned} {\widetilde{\varphi }}'(b)=f'(b){\widetilde{\varphi }}(b) \end{aligned}$$

where ${\widetilde{\varphi }}'(b)$ denotes the outgoing radial derivative of ${\widetilde{\varphi }}$ at length *b*. Combining (3.42) and (3.44) yields 1. Property 2. is a direct consequence of $\widetilde{v}$ being a uniform measure on the sphere $\{\vert x\vert \} =b$ and the behaviour of $J_0$ at infinity. Finally, the identities in 3. follow immediately after realizing that

$$\begin{aligned}g={\widetilde{\varphi }}(b)v.\end{aligned}$$

$$\square $$

## Upper Bound for a Soft Potential

We denote by ${\mathscr {M}}_c$ the set of potentials of the form $v = v_{\textrm{reg}} + v_{\textrm{m}}$, where $v_{\textrm{reg}} \in L^{1}({{\mathbb {R}}}^2)$ is radial, positive and has compact support, and where $v_{\textrm{m}} = C \delta _{\{|x|=r\}}$ for some $C \ge 0$ and $r>0$. If $v \in {\mathscr {M}}_c$, it admits a bounded and continuous Fourier transform ${{\widehat{v}}}$. The aim of this section is to prove an upper bound on the ground state energy of

4.1 $$\begin{aligned} {\mathcal {H}}_v = \bigoplus _{n \ge 0} \bigg ( \sum _{i=1}^n - \Delta _{x_i}^{\text {per}} + \sum _{1 \le i<j \le n} v^{\text {per}}(x_i-x_j) \bigg ) \end{aligned}$$

on the box $\Lambda _\beta = [ - \frac{L_\beta }{2} , \frac{L_\beta }{2} ]^2$ for potentials $v \in {\mathscr {M}}_c$, under some additional decay assumption on the Fourier transform of *v*. We recall that $L_\beta = \rho ^{- \frac{1}{2}} Y^{-\beta }$.

In this section, we will denote by $\varphi$ the scattering solution of the given *v*, normalized at length $\widetilde{R}$, and $g=\varphi v$, see (3.4). Notice here that the theory of Sect. 3 extends to potentials $v \in {\mathscr {M}}_c$; for this we use in particular, that if $u \in H^1({{\mathbb {R}}}^2)$, then $u|_{\{|x|=r\}} \in L^2$ so the variational problem in Definition 3.1 is well posed. In particular, the scattering equation (3.2) is valid in the distributional sense. We recall that $0 \le {\widehat{g}}_0=8\pi \delta \le C Y$ by (3.6) and (3.3). We prove the following upper bound, which is very similar in spirit to the upper bound of [15] in the 3*D* case.

### Theorem 4.1

For any given $c_0>0$ and $\beta \ge \frac{3}{2}$, there exists $C_\beta >0$ (only depending on $c_0$ and $\beta$) such that the following holds. Let $\rho >0$ and $v \in {\mathscr {M}}_c$ be a radial positive measure with scattering length *a* and ${{\,\mathrm{\textrm{supp}}\,}}v \subset B(0,R)$, for some $R>0$. Let ${{\mathcal {H}}}_v$ be as defined in (2.3). Assume that

4.2 $$\begin{aligned} \vert {\widehat{g}}_p \vert \le c_0 \frac{{\widehat{g}}_0}{\sqrt{R \vert p \vert }}, \qquad \forall \vert p \vert \ge a^{-1}. \end{aligned}$$

Then, if $\rho R^2 \le Y$ and $\rho a^2 \le C_{\beta }^{-1}$, one can find a normalized trial state $\Phi \in {\mathscr {F}}_s(L^2(\Lambda _\beta ))$ satisfying

$$\begin{aligned} \langle {\mathcal {H}}_v \rangle _\Phi&\le 4\pi L_\beta ^2 \rho ^2 \delta _0 \Big (1 + \Big (2\Gamma + \frac{1}{2} + \log (\pi ) \Big ) \delta _0 \Big ) + CL_{\beta }^2 \rho ^2 \delta _0({\widehat{v}}_0-{\widehat{g}}_0)+CL_{\beta }^2 \rho ^2 \delta ^{2}_0{\widehat{v}}_0 \end{aligned}$$

with $\langle {\mathcal {N}} \rangle _\Phi = N ,$ and $\langle {\mathcal {N}}^2 \rangle _\Phi \le 9 N^2$, where $N = \rho L_\beta ^2 = Y^{-2\beta }$.

### Remark 4.2

Note that this result is much weaker than Theorem 2.2. Indeed, the remainders are only of order $\rho ^2 L_\beta ^2 \delta _0^2$ and $\rho ^2 L_\beta ^2 \delta _0$ and thus much larger than the 2D-LHY term, unless ${\widehat{v}}_0 = {\widehat{g}}_0 + o(\delta _0)$. Moreover, Theorem 4.1 only holds for potentials with finite integral and, in particular, it does not allow for a hard core. However, in the proof of Theorem 2.2 in Sect. 5 we will show how to reduce to such potentials. More precisely, we will apply Theorem 4.1 to a surface potential of the form (3.40) (with the choice of *b* given in (2.5)).

### Remark 4.3

The specific $\delta =\delta _{0}$ defined in (1.5) is chosen to minimize the upper bound (4.22) up to the LHY precision. This corresponds to fixing the normalisation length of the soft potential $\widetilde{R}=ae^{\frac{1}{2\delta }}$. See also Remarks 3.2, 4.9 and C.4.

The rest of Sect. 4 is dedicated to the proof of Theorem 4.1. We will give an explicit trial state and state several technical calculations as lemmas. In the end we collect the pieces and finish the proof.

### A quasi-free state

We will define our trial state $\Phi$ in second quantization formalism. On the bosonic Fock space ${\mathscr {F}}( L^2(\Lambda _\beta ))$, we will denote by $a_p^\dagger$ and $a_p$ the creation and annihilation operators associated to the function $x \mapsto \vert \Lambda _\beta \vert ^{-\frac{1}{2}} \exp (ipx)$, for $p \in \Lambda ^*_\beta = (\frac{2\pi }{L_\beta } {\mathbb {Z}})^2$. Our quasi-free state is $\Phi = T_{\nu } W_{N_0} \Omega$ where $\Omega$ is the vacuum, $W_{N_0}$ creates the condensate and $T_\nu$ the excitations:

4.3 $$\begin{aligned} W_{N_0} = \exp \Big ( \sqrt{N_0} (a_0^\dagger - a_0) \Big ), \quad T_{\nu } = \exp \Big (\frac{1}{2} \sum _{p\ne 0} \nu _p (a_p^\dagger a_{-p}^\dagger - a_p a_{-p}) \Big ), \end{aligned}$$

for a given $N_0\le N$ associated with $\rho _0 := N_0/L_{\beta }^2$. These operators have the nice properties that

4.4 $$\begin{aligned} W_{N_0}^* a_0 W_{N_0} = a_0 + \sqrt{ N_0 }, \quad \text { and } \quad T_\nu ^* a_p T_\nu = \cosh (\nu _p) a_p + \sinh (\nu _p) a_{-p}^\dagger . \end{aligned}$$

In particular, for any *p*, $q \in \Lambda ^*_\beta$,

4.5 $$\begin{aligned}{} & {} \langle a_q^\dagger a_p \rangle _\Phi = {\left\{ \begin{array}{ll} N_0, &{} \text { if } p=q=0, \\ 0, &{} \text { if } p\ne q, \\ \gamma _q, &{} \text { if } p=q \ne 0, \end{array}\right. } \quad \text {and} \nonumber \\{} & {} \langle a_q a_p \rangle _\Phi =\langle a_q^\dagger a_p^\dagger \rangle _\Phi = {\left\{ \begin{array}{ll} N_0, &{} \text { if } p=q=0, \\ 0, &{} \text { if } p\ne -q, \\ \alpha _q, &{} \text { if } p=-q \ne 0, \end{array}\right. } \end{aligned}$$

where $\alpha _p = \cosh (\nu _p) \sinh (\nu _p)$ and $\gamma _p = \sinh (\nu _p)^2$. We choose the coefficient $\nu _p$ such that

4.6 $$\begin{aligned} \alpha _p = \frac{-\rho _0 {\widehat{g}}_p}{2 \sqrt{p^4 + 2 \rho _0 {\widehat{g}}_p p^2}} , \qquad \gamma _p = \frac{p^2 + \rho _0 {\widehat{g}}_p - \sqrt{p^4 + 2 \rho _0 {\widehat{g}}_p p^2}}{2 \sqrt{p^4 + 2 \rho _0 {\widehat{g}}_p p^2}}\ge 0 , \end{aligned}$$

this specific choice coming from a minimization of the energy

$$\begin{aligned}(p^2 + \rho _0 {\widehat{g}}_p) \gamma _p + \rho _0 {{\widehat{g}}}_p \alpha _p\end{aligned}$$

obtained in Lemma 4.6 up to changing ${\widehat{v}}$ into ${\widehat{g}}$. Note that by $(\cosh (x)^{2}- \sinh (x)^{2})=1$ we have $\alpha _p^2 = \gamma _p (\gamma _p + 1)$, making it a possible choice. These coefficients satisfy the following estimates.

#### Lemma 4.4

We estimate the sum (over $\Lambda _{\beta }^{*}$) of $\alpha _{p}$ and $\gamma _{p}$:

4.7 $$\begin{aligned} \sum _{p\ne 0}\left| \alpha _{p}\right| \le C N, \quad \text {and}\quad \sum _{p\ne 0}\gamma _{p}\le C N \delta . \end{aligned}$$

#### Proof

We start from the expression of $\alpha _{p}$ (4.6) and split the sum between $\vert p \vert \le \sqrt{\rho _0 {\widehat{g}}_0}$ and $\vert p \vert \ge \sqrt{\rho _0 {\widehat{g}}_0 }$:

$$\begin{aligned} \sum _{p\ne 0}\left| \alpha _{p}\right|&\le C\sqrt{\rho _0 {\widehat{g}}_0}\sum _{0<\vert p\vert \le \sqrt{\rho _0 {\widehat{g}}_0}}\frac{1}{\vert p\vert }+ C\rho _0\sum _{\vert p\vert \ge \sqrt{\rho _0 {\widehat{g}}_0}}\frac{ \vert {\widehat{g}}_{p}\vert }{\vert p\vert ^{2}}\\&\le CL_\beta ^{2} \sqrt{\rho _0 {\widehat{g}}_0}\int _{0}^{\sqrt{\rho _0 {\widehat{g}}_0}}\textrm{d}u + C L_\beta ^{2} \rho _0 {\widehat{g}}_0\int _{\sqrt{\rho _0 {\widehat{g}}_0}}^{a^{-1}}\frac{\textrm{d}u}{u}+ C L_\beta ^{2}\rho _0 \int _{ a^{-1}}^{+\infty }\frac{ {\widehat{g}}_0}{R^{1/2}u^{3/2}} \textrm{d}u\\&\le CL_\beta ^{2}\rho _0 {\widehat{g}}_0 (1 + \vert \log (a^2 \rho _0 {\widehat{g}}_0) \vert ) \le C N, \end{aligned}$$

where we used the decay of ${\widehat{g}}_{p}$ at infinity (4.2) and the bound $a \le R$. For $\gamma _{p}$ we also split the sum this way. For $p\le \sqrt{\rho _0 {\widehat{g}}_0}$ we obtain that

$$\begin{aligned} \sum _{\vert p\vert \le \sqrt{\rho _0 {\widehat{g}}_{0}}}\vert \gamma _{p}\vert \le C\sum _{\vert p\vert \le \sqrt{\rho _0 {\widehat{g}}_{0}}}\frac{\sqrt{\rho _0 {\widehat{g}}_{0}}}{\vert p\vert } \le CL_\beta ^{2} \rho _0 {\widehat{g}}_{0} \le C N_0 \delta . \end{aligned}$$

For $p\ge \sqrt{\rho _0 {\widehat{g}}_0}$ we expand the square root and find

$$\begin{aligned} \sum _{\vert p\vert \ge \sqrt{\rho _0 {\widehat{g}}_{0}}}\vert \gamma _{p}\vert \le C\sum _{\vert p\vert \ge \sqrt{\rho _0 {\widehat{g}}_{0}}}\frac{\left( \rho _0 {\widehat{g}}_{0}\right) ^{2}}{\vert p\vert ^{4} } \le CL^{2}_\beta \rho _0 {\widehat{g}}_{0} \le C N_0 \delta , \end{aligned}$$

which concludes the proof. $\square$

Finally choose $N_0$ such that

4.8 $$\begin{aligned} \rho L_{\beta }^2 = N=N_0 + \sum _{p \ne 0} \gamma _p . \end{aligned}$$

Note that with this choice $\Phi$ has the expected average number of particles as stated in the next lemma.

#### Lemma 4.5

The state $\Phi = T_{\nu } W_{N_0} \Omega$ satisfies

$$\begin{aligned} \langle {\mathcal {N}} \rangle _ \Phi = N , \qquad \langle {{\mathcal {N}}}^2 \rangle _\Phi \le C N^2 , \end{aligned}$$

where ${\mathcal {N}} = \sum _{p \in \Lambda _\beta ^*} a_p^\dagger a_p$ is the number operator.

#### Proof

First we can use the property (4.5) to find

4.9 $$\begin{aligned} \langle {\mathcal {N}} \rangle _\Phi = N_0 + \sum _{p\ne 0} \gamma _p = N. \end{aligned}$$

For ${\mathcal {N}}^2$ we split the sums according to zero and non-zero momenta, and then conjugate by $W_{N_{0}}$,

$$\begin{aligned} \langle {\mathcal {N}}^2 \rangle _\Phi&= \sum _{q,p} \langle a_p^\dagger a_p a_q^\dagger a_q \rangle _\Phi =N_{0}^{2}+N_{0}+\sum _{q \ne 0}\langle a_0^\dagger a_0 a_q^\dagger a_q+\text {h.c} \rangle _\Phi + \sum _{q\ne 0,p\ne 0} \langle a_p^\dagger a_p a_q^\dagger a_q \rangle _\Phi \\&=N_{0}^{2}+N_{0}\Big ( 1+2\sum _{p\ne 0} \gamma _p \Big )+\sum _{q\ne 0, p\ne 0} \langle a_p^\dagger a_p a_q^\dagger a_q \rangle _\Phi . \end{aligned}$$

Now we use Lemma 4.4 and apply Wick’s Theorem [27, Theorem 10.2] to the state $T_\nu \Omega$ to find

$$\begin{aligned} \langle {\mathcal {N}}^2 \rangle _\Phi&\le 4N^{2}+\sum _{q\ne 0,p\ne 0} \Big ( \langle a_p^\dagger a_p \rangle _\Phi \langle a_q^\dagger a_q \rangle _\Phi + \langle a_p^\dagger a_q^\dagger \rangle _\Phi \langle a_p a_q \rangle _\Phi + \langle a_p^\dagger a_q \rangle _\Phi \langle a_p a_q^\dagger \rangle _\Phi \Big )\\&\le 4N^{2}+ \Big ( \sum _{p\ne 0} \gamma _p\Big )^{2} + \sum _{p\ne 0} \alpha _p^{2}+ \sum _{p\ne 0} (\gamma _{p}^{2}+\gamma _{p})\le C N^{2} \, \end{aligned}$$

using Lemma 4.4. $$\square $$

### Energy of $\Phi$

In order to get an upper bound on the energy of $\Phi$ we first introduce the quantity

4.10 $$\begin{aligned} D(A,B) = \frac{1}{(4\pi ^2)^2} \int {\widehat{v}}* A (p) B(p) \textrm{d}p = \frac{1}{(4\pi ^2)^2} \langle B , {\widehat{v}}* A \rangle , \end{aligned}$$

and observe that it is symmetric in the entries. Then we prove the following result.

#### Lemma 4.6

Under the assumptions of Theorem 4.1, there exists a constant $C>0$, independent of *v* and $\rho$, such that

$$\begin{aligned} \vert \Lambda _\beta \vert ^{-1} \langle {\mathcal {H}}_v \rangle _\Phi \le \frac{\rho ^2}{2 } {\widehat{v}}_0 + \int \Big ((p^2 + \rho _0 {\widehat{v}}_p) \gamma _p + \rho _0 {\widehat{v}}_p \alpha _p \Big )\frac{\textrm{d}p}{4\pi ^2} + \frac{1}{2} D(\alpha , \alpha ) + C {\widehat{v}}_0 \rho ^2 Y^3. \end{aligned}$$

#### Proof

One can write ${\mathcal {H}}_v$ in second quantization in momentum variable,

$$\begin{aligned} {\mathcal {H}}_v = \sum _{p \in \Lambda _\beta ^*} p^2 a_p^\dagger a_p + \frac{1}{2\vert \Lambda _\beta \vert } \sum _{p,q,r} {\widehat{v}}_r a^\dagger _{p+r} a^\dagger _q a_{q+r} a_p , \end{aligned}$$

and express the energy of $\Phi$ in terms of $\alpha _{p}$ and $\gamma _{p}$ as follows. We conjugate by $W_{N_0}$ using (4.4), which amounts to change the $a_{0}$’s in $\sqrt{N_{0}}$. Since $\Phi = T_{\nu } W_{N_0} \Omega$ with no $a_{0}$ in $T_{\nu }$ (see (4.3)), when we apply $\Phi$ we find

4.11 $$\begin{aligned} \langle {\mathcal {H}}_v \rangle _\Phi&= \sum _{p \ne 0} p^2 \langle a_p^\dagger a_p \rangle _\Phi + \frac{N_0^2}{2\vert \Lambda _\beta \vert } {\widehat{v}}_0 + \frac{N_0}{ \vert \Lambda _\beta \vert } \sum _{p \ne 0} ( {{\widehat{v}}}_0 + {{\widehat{v}}}_p) \langle a_p^\dagger a_p \rangle _{\Phi }\nonumber \\ {}&\quad + \frac{N_0}{\vert \Lambda _\beta \vert } \sum _{r\ne 0} {{\widehat{v}}}_r \langle a_r a_{-r} \rangle _\Phi + \frac{\sqrt{N_0}}{\vert \Lambda _\beta \vert } \sum _{\begin{array}{c} q,r\ne 0 \\ q+r \ne 0 \end{array}} {{\widehat{v}}}_r \langle a_r^\dagger a_q^\dagger a_{q+r} \rangle _{\Phi }\nonumber \\&\quad + \frac{\sqrt{N_0}}{\vert \Lambda _\beta \vert } \sum _{\begin{array}{c} p,r\ne 0 \\ p+r \ne 0 \end{array}} {{\widehat{v}}}_{r} \langle a_{p+r}^\dagger a_{-r}^\dagger a_{p} \rangle _\Phi + \frac{1}{2 \vert \Lambda _\beta \vert } \sum _{\begin{array}{c} p,q \ne 0 \\ p+r, q+r \ne 0 \end{array}} {{\widehat{v}}}_r \langle a_{p+r}^\dagger a_q^\dagger a_{q+r} a_p \rangle _\Phi . \end{aligned}$$

We can use Wick’s Theorem [27, Theorem 10.2] to the state $T_\nu \Omega$. By definition of $\alpha _p$ and $\gamma _p$ in (4.5) together with $N^2 = (N_0 + \sum _{p\ne 0} \gamma _p)^2$ we deduce

4.12 $$\begin{aligned} \langle {\mathcal {H}}_v \rangle _\Phi&= \frac{N^2}{2 \vert \Lambda _\beta \vert } {\widehat{v}}_0 + \sum _{p\ne 0} p^2 \gamma _p + \frac{N_0}{\vert \Lambda _\beta \vert } \sum _{p\ne 0} \Big ( {\widehat{v}}_p \gamma _p + {\widehat{v}}_p \alpha _p \Big )\nonumber \\&\quad + \frac{1}{2 \vert \Lambda _\beta \vert }\sum _{\begin{array}{c} q\ne 0\\ q+r \ne 0 \end{array}} {\widehat{v}}_r \alpha _q \alpha _{q+r} + \frac{1}{2 \vert \Lambda _\beta \vert }\sum _{\begin{array}{c} q\ne 0\\ q+r \ne 0 \end{array}} {\widehat{v}}_r \gamma _q \gamma _{q+r} . \end{aligned}$$

We bound the last term in the above using Lemma 4.4. With $\rho = N \vert \Lambda _\beta \vert ^{-1}$ and $\rho _{0}=N_{0} \vert \Lambda _\beta \vert ^{-1}$ we deduce

4.13 $$\begin{aligned} \vert \Lambda _\beta \vert ^{-1} \langle {\mathcal {H}}_v \rangle _\Phi&\le \frac{1}{2} \rho ^2 {\widehat{v}}_0 + \frac{1}{\vert \Lambda _\beta \vert } \sum _{p \ne 0} \Big ((p^2 + \rho _0 {\widehat{v}}_p) \gamma _p + \rho _0 {\widehat{v}}_p \alpha _p \Big )\nonumber \\ {}&\quad + \frac{1}{2\vert \Lambda _\beta \vert ^2} \sum _{\begin{array}{c} q\ne 0\\ q+r \ne 0 \end{array}} {\widehat{v}}_r \alpha _q \alpha _{q+r} + C {\widehat{v}}_0 \rho ^2 Y^2 . \end{aligned}$$

Up to errors ${\mathcal {E}} \le C {\widehat{v}}_0 \rho ^2 Y^{\frac{1}{2}+\beta }$, we can approximate these Riemann sums by integrals (see Lemma G.1) and the lemma follows. In fact, the requirement $\beta \ge 3/2$ in Theorem 4.1 comes from here. $\square$

#### Lemma 4.7

Under the assumptions of Theorem 4.1, there exists a constant $C>0,$ independent of *v* and $\rho$, such that

$$\begin{aligned}&\vert \Lambda _\beta \vert ^{-1} \langle {\mathcal {H}}_{v} \rangle _\Phi \\&\le \frac{\rho ^2}{2} {{\widehat{g}}}_0 + \frac{1}{2} \int \Big ( \sqrt{p^4 + 2 \rho _0 p^2 {{\widehat{g}}}_p} -p^2 - \rho _0 \widehat{g}_p+ \rho _0^2\frac{{{\widehat{g}}}^2_p - {{\widehat{g}}}_0^2 \mathbb {1}_{\{p \le 2 e^{-\Gamma } \widetilde{R}^{-1}\}}}{2p^2} \Big )\frac{\textrm{d}p }{4 \pi ^2} \\ {}&\quad + \frac{1}{2} D(\alpha + \rho _0 {{\widehat{\omega }}}, \alpha + \rho _0 {{\widehat{\omega }}}) + C \rho ^2 Y ( {\widehat{v}}_0 - \widehat{g}_0 ) + C {\widehat{v}}_0 \rho ^2 Y^2 . \end{aligned}$$

#### Proof

We recall the definition (3.4) of $\omega$, and we insert $\rho _0 {\widehat{\omega }}$ into $D(\alpha ,\alpha )$,

$$\begin{aligned} D(\alpha ,\alpha ) = - \rho _0^2 D({{\widehat{\omega }}}, {{\widehat{\omega }}}) - 2 \rho _0 D(\alpha , {{\widehat{\omega }}}) + D(\alpha + \rho _0 {{\widehat{\omega }}}, \alpha + \rho _0 {{\widehat{\omega }}}) . \end{aligned}$$

Inserting this into Lemma 4.6 we find

4.14 $$\begin{aligned} \frac{\langle {\mathcal {H}}_v \rangle _\Phi }{\vert \Lambda _\beta \vert }&\le \frac{\rho ^2}{2} {\widehat{v}}_0 - \frac{\rho _0^2}{2} D({{\widehat{\omega }}}, {{\widehat{\omega }}}) + \int \Big ((p^2 + \rho _0 {\widehat{v}}_p) \gamma _p + \rho _0 ({\widehat{v}}_p - \widehat{v \omega }_p) \alpha _p\Big ) \frac{\textrm{d}p}{4\pi ^2} \nonumber \\&\quad + \frac{1}{2} D(\alpha + \rho _0 {{\widehat{\omega }}}, \alpha + \rho _0 {{\widehat{\omega }}}) + C {\widehat{v}}_0 \rho ^2 Y^2 . \end{aligned}$$

Now note that ${{\widehat{g}}} _p = {\widehat{v}}_p - ( {\widehat{v}}* {{\widehat{\omega }}})_p$ and,

$$\begin{aligned} \frac{\rho ^2}{2} {\widehat{v}}_0&= \frac{\rho ^2}{2} {{\widehat{g}}} _0 + \frac{\rho _0^2}{2} (\widehat{v \omega })_0 + \frac{\rho ^2 -\rho _0^2 }{2} (\widehat{v \omega })_0 \\&= \frac{\rho ^2}{2} {{\widehat{g}}}_0 + \frac{\rho _0^2}{2} (\widehat{g\omega })_0 + \frac{\rho _0^2}{2} (\widehat{v \omega ^2})_0 + \frac{\rho ^2 -\rho _0^2 }{2} (\widehat{v \omega })_0. \end{aligned}$$

This equality inserted in (4.14), together with $D({{\widehat{\omega }}}, {{\widehat{\omega }}}) = (\widehat{v \omega ^2})_0$ implies

4.15 $$\begin{aligned} \frac{\langle {\mathcal {H}}_v \rangle _\Phi }{\vert \Lambda \vert }&= \frac{\rho ^2}{2} {{\widehat{g}}}_0 + \frac{\rho _0^2}{2} (\widehat{g\omega })_0 + \int \Big ((p^2 + \rho _0 {\widehat{v}}_p) \gamma _p + \rho _0 {{\widehat{g}}}_p \alpha _p \Big )\frac{\textrm{d}p}{4\pi ^2} \nonumber \\&\quad + \frac{1}{2} D(\alpha + \rho _0 {{\widehat{\omega }}}, \alpha + \rho _0 {{\widehat{\omega }}}) + \frac{\rho ^2 - \rho _0^2}{2} (\widehat{v\omega })_0 + C {\widehat{v}}_0 \rho ^2 Y^2 . \end{aligned}$$

Our choice of $\gamma$ and $\alpha$ minimizes the integral where we replaced ${\widehat{v}}_p$ by ${{\widehat{g}}}_p$, and by explicit computation using the definition (4.6) of $\alpha$ and $\gamma$ we find

4.16 $$\begin{aligned} \int (p^2 + \rho _0 {{\widehat{g}}}_p) \gamma _p + \rho _0 {{\widehat{g}}}_p \alpha _p \frac{\textrm{d}p}{2 \pi ^2} = \frac{1}{2} \int \Big (\sqrt{p^4 + 2 \rho _0 p^2 {{\widehat{g}}}_p}-p^2 - \rho _0 {{\widehat{g}}}_p \Big )\frac{\textrm{d}p }{4 \pi ^2} .\nonumber \\ \end{aligned}$$

Moreover the formula for $\widehat{g\omega }$ from Lemma 3.9 yields

$$\begin{aligned} (\widehat{g\omega })_0 = \langle {{\widehat{\omega }}}, {{\widehat{g}}} \rangle = \int \frac{{{\widehat{g}}}^2_p - {{\widehat{g}}}_0^2 \mathbb {1}_{\{p \le \ell _\delta ^{-1}\}}}{2p^2} \frac{\textrm{d}p}{4 \pi ^2}, \end{aligned}$$

where $\ell _\delta = \frac{1}{2} e^{\Gamma } \widetilde{R}$. Inserting this and (4.16) into (4.15) we find

$$\begin{aligned} \frac{\langle {\mathcal {H}}_{v} \rangle _\Phi }{\vert \Lambda \vert }&= \frac{\rho ^2}{2} {{\widehat{g}}}_0 + \frac{1}{2} \int \Big ( \sqrt{p^4 + 2 \rho _0 p^2 {{\widehat{g}}}_p} -p^2 - \rho _0 {{\widehat{g}}}_p+ \rho _0^2\frac{{{\widehat{g}}}^2_p - {{\widehat{g}}}_0^2 \mathbb {1}_{\{p \le \ell ^{-1}_{\delta }\}}}{2p^2} \Big )\frac{\textrm{d}p }{4 \pi ^2} \\&\quad + \frac{1}{2} D(\alpha + \rho _0 {{\widehat{\omega }}}, \alpha + \rho _0 {{\widehat{\omega }}}) \\&\quad + \frac{\rho ^2 - \rho _0^2}{2} (\widehat{v\omega })_0 + \rho _0 \int ({\widehat{v}}_p - {{\widehat{g}}}_p) \gamma _p \frac{\textrm{d}p}{4 \pi ^2}+ C {\widehat{v}}_0 \rho ^2 Y^2, \end{aligned}$$

where the last integral comes from the replacement of ${\widehat{v}}_p$ by ${{\widehat{g}}}_p$ in the first term of the integral in (4.15). Since $\rho - \rho _0 \le C\rho Y$ (Lemma 4.4 and Lemma G.1) and $\vert {\widehat{v}}_p - {{\widehat{g}}}_p \vert \le (\widehat{v\omega })_0 = {\widehat{v}}_0 - {{\widehat{g}}}_0$, we can bound

$$\begin{aligned} \frac{\rho ^2 - \rho _0^2}{2} (\widehat{v\omega })_0 + \rho _0 \int ({\widehat{v}}_p - {{\widehat{g}}}_p) \gamma _p \frac{\textrm{d}p}{4 \pi ^2} \le C \rho ^2 Y ({\widehat{v}}_0 - {{\widehat{g}}}_0), \end{aligned}$$

and the lemma follows. $\square$

In the following lemma we estimate the remainder term from Lemma 4.7.

#### Lemma 4.8

There is a $C>0$ independent of *v* and $\rho$ such that:

$$\begin{aligned} D(\alpha + \rho _0 {{\widehat{\omega }}}, \alpha + \rho _0 {{\widehat{\omega }}}) \le \rho _{0}^{2}\delta ^{2}{\widehat{v}}_{0} \Big \vert \frac{1}{\delta }-\frac{1}{Y}+\log \delta \Big \vert +\rho _{0}^{2}\delta ^{2}{\widehat{v}}_{0} \Big (\frac{1}{\delta }-\frac{1}{Y}+\log \delta \Big )^{2}+ C {\widehat{v}}_0 \rho _0^2 {\widehat{g}}_0^2 .\end{aligned}$$

In particular, with $\delta = \delta _0$ defined in (1.5) we deduce

$$\begin{aligned} D(\alpha + \rho _0 {{\widehat{\omega }}}, \alpha + \rho _0 {{\widehat{\omega }}}) \le C {{\widehat{v}}}_0 \rho ^2 \delta _0^2. \end{aligned}$$

#### Proof

We recall the definition of $\ell _{\delta }$ in (3.30). We first estimate $h_p := \langle \alpha + \rho _0 {{\widehat{\omega }}}, {\widehat{v}}_{p-\cdot } \rangle$, using Lemma 3.8 as

4.17 $$\begin{aligned} h_p&= \int \Big (\frac{ \rho _0 {{\widehat{g}}}_q {\widehat{v}}_{p-q} - \rho _0 {{\widehat{g}}}_0 {\widehat{v}}_p \mathbb {1}_{\{\vert q\vert< \sqrt{\rho _{0} \widehat{g}_0}\}}}{2 q^2}+\frac{ \rho _0 {{\widehat{g}}}_0 {\widehat{v}}_p \mathbb {1}_{\{ \ell ^{-1}_{\delta }< \vert q\vert <\sqrt{\rho _{0} {{\widehat{g}}}_0} \}}}{2 q^2} \nonumber \\&\quad - \frac{\rho _0 {{\widehat{g}}}_q {\widehat{v}}_{p-q}}{2 \sqrt{ q^4 + 2 \rho _0 q^2 {{\widehat{g}}}_q}}\Big ) \frac{\textrm{d}q}{4 \pi ^2}\nonumber \\&= h_p^{(1)} + h_p^{(2)} +h_p^{(3)}, \end{aligned}$$

with

4.18 $$\begin{aligned} \vert h_p^{(1)} \vert&= \Big \vert \int _{\{\vert q \vert> \sqrt{\rho _0 {{\widehat{g}}}_0}\}} \frac{\rho _0 {{\widehat{g}}}_q {\widehat{v}}_{p-q}}{2q^2} \Big ( 1 - \frac{1}{\sqrt{1+ \frac{2 \rho _0 \widehat{g}_q}{q^2}}} \Big ) \frac{\textrm{d}q}{4 \pi ^2} \Big \vert \nonumber \\&\le C {\widehat{v}}_0 \int _{\{\vert q \vert > \sqrt{\rho _0 {{\widehat{g}}}_0}\}} \frac{(\rho _0 {{\widehat{g}}}_0)^2}{q^4} \textrm{d}q \le C {\widehat{v}}_0 \rho _0 {\widehat{g}}_0 . \end{aligned}$$

We also calculate

4.19 $$\begin{aligned} \vert h_p^{(2)} \vert&=\Big \vert \int _{\{ \ell ^{-1}_{\delta }<\vert q \vert < \sqrt{\rho _0 {{\widehat{g}}}_0}\}} \frac{\rho _0 {{\widehat{g}}}_0 {\widehat{v}}_{p}}{2q^2} \frac{\textrm{d}q}{4 \pi ^2} \Big \vert \nonumber \\&\le C\rho _{0}{{\widehat{g}}}_0{\widehat{v}}_0\Big \vert \log \Big (\sqrt{\rho _{0}a^{2}}\sqrt{{{\widehat{g}}}_0}\frac{e^{\Gamma }}{2}e^{\frac{1}{2\delta }} \Big )\Big \vert \nonumber \\&= \rho _{0}\delta {\widehat{v}}_0 \Big \vert \frac{1}{\delta }-\frac{1}{Y}+\log \delta +C \Big \vert . \end{aligned}$$

In the case where $\ell ^{-1}_{\delta }\ge \sqrt{\rho _{0}{\widehat{g}}_{0}}$, the same estimates hold true. Only the inequalities inside the indicator function in (4.17) change. Using (3.30) we have,

$$\begin{aligned} \vert h_p^{(3)} \vert =&\, \Big \vert \int _{\{\vert q \vert<\sqrt{\rho _{0} {{\widehat{g}}}_0}\}} \frac{\rho _0 ({{\widehat{g}}}_q {\widehat{v}}_{p-q} - {{\widehat{g}}}_0 {\widehat{v}}_p ) }{2 q^2} \frac{\textrm{d}q}{4\pi ^2} - \int _{\{\vert q \vert< \sqrt{\rho _0 {\widehat{g}}_0}\}} \frac{\rho _0 {{\widehat{g}}}_q {\widehat{v}}_{p-q}}{2 \sqrt{q^4 + 2 \rho _0 q^2 {{\widehat{g}}}_q}} \frac{\textrm{d}q }{4 \pi ^2} \Big \vert \\ \le&\, \int _{\{\vert q \vert< \sqrt{\rho _{0} {{\widehat{g}}}_0}\}} \frac{\rho _0 {{\widehat{g}}}_0 \vert {\widehat{v}}_{p-q} - {\widehat{v}}_p \vert +\rho _0 \vert {\widehat{g}}_q - {\widehat{g}}_0 \vert {\widehat{v}}_0}{2 q^2} \frac{\textrm{d}q}{4\pi ^2}\\&+ C {\widehat{v}}_0 \sqrt{\rho _0 {{\widehat{g}}}_0} \int _{\{\vert q \vert< \sqrt{\rho _{0} {{\widehat{g}}}_0}\}} \frac{1}{\vert q \vert } \textrm{d}q \\&\le C \Vert \nabla {\widehat{v}}\Vert _{\infty } \int _{\{\vert q \vert < \sqrt{\rho _{0} {{\widehat{g}}}_0}\}} \frac{\rho _0 {{\widehat{g}}}_0}{\vert q \vert } \textrm{d}q + C {\widehat{v}}_0 \rho _0 {\widehat{g}}_0 \le C{\widehat{v}}_0 \rho _0 {\widehat{g}}_0, \end{aligned}$$

where we used $\ell ^{-1}_{\delta }\sim \sqrt{\rho {\widehat{g}}_0}\le R$ and $\Vert \nabla {\widehat{v}}_0\Vert _{\infty }\le R{\widehat{v}}_0$. In the end we obtain

$$\begin{aligned} \vert h_p \vert&\le C{\widehat{v}}_0 \rho _0 {\widehat{g}}_0 +\rho _{0}\delta {\widehat{v}}_0 \Big (\frac{1}{\delta }-\frac{1}{Y}+\log \delta +C \Big ). \end{aligned}$$

Similarly we have bounds on the gradient of *h*, namely

4.20 $$\begin{aligned} \vert \nabla h_p \vert \le CR{\widehat{v}}_0 \rho _0{\widehat{g}}_0. \end{aligned}$$

Now we turn to

$$\begin{aligned} D(\alpha + \rho _0 {{\widehat{\omega }}}, \alpha + \rho _0 {{\widehat{\omega }}})&= \langle \alpha + \rho _0 {{\widehat{\omega }}}, h \rangle \\&= \int \Big (\frac{ \rho _0 {{\widehat{g}}}_q h_{q} - \rho _0 {{\widehat{g}}}_0 h_0 \mathbb {1}_{\{\vert q \vert > \ell ^{-1}_{\delta }\}}}{2 q^2} - \frac{\rho _0 {{\widehat{g}}}_q h_{q}}{2 \sqrt{ q^4 + 2 \rho _0 q^2 {{\widehat{g}}}_q}} \Big )\frac{\textrm{d}q}{4 \pi ^2}, \end{aligned}$$

which we in the same way write as $D_1 + D_2+ D_{3}$ with

$$\begin{aligned} \vert D_1\vert&= \Big \vert \int _{\{\vert q \vert> \sqrt{\rho _0{\widehat{g}}_0}\}} \frac{\rho _0 {{\widehat{g}}}_q h_q}{2 q^2} \Big ( 1 - \frac{1}{\sqrt{1+ \frac{2 \rho _0 {{\widehat{g}}}_q}{q^2}}} \Big ) \frac{\textrm{d}q}{4 \pi ^2} \Big \vert \\&\le \frac{\Vert h \Vert _{\infty }}{8\pi ^{2}} \int _{\{\vert q \vert > \sqrt{\rho _0{\widehat{g}}_0}\}} \frac{(\rho _0 {{\widehat{g}}}_0)^2}{q^4} \textrm{d}q \\&\le C{\widehat{v}}_0 \rho _0^{2} {\widehat{g}}_0^{2}+\rho _{0}^{2}\delta ^{2}{\widehat{v}}_{0} \Big (\frac{1}{\delta }-\frac{1}{Y}+\log \delta +C \Big ), \end{aligned}$$

and using the bounds on *h*, we find $\vert D_1 \vert \le C {\widehat{v}}_0 \rho _0^2 {\widehat{g}}_0^2$. The technique to bound $D_{2}$ is the same as for $h^{(2)}$ and its provides

$$\begin{aligned} \vert D_2 \vert = \Big \vert \int _{\{\ell ^{-1}_{\delta }< \vert q \vert < \sqrt{\rho _0 {\widehat{g}}_0} \}} \frac{ \rho _0 {{\widehat{g}}}_0 h_0 }{2 q^2} \frac{\textrm{d}q}{4 \pi ^2}\Big \vert \le {\widehat{v}}_0 \rho _0^2\delta ^{2} \Big (\frac{1}{\delta }-\frac{1}{Y}+\log \delta +C \Big )^{2}. \end{aligned}$$

Lastly $D_3$ is bounded just as $h^{(3)}$,

$$\begin{aligned} \vert D_3 \vert&= \Big \vert \int _{\{\vert q \vert< \sqrt{\rho _0{\widehat{g}}_0}\}} \frac{\rho _0 ({{\widehat{g}}}_q h_{q} - {{\widehat{g}}}_0 h_0 ) }{2 q^2} \frac{\textrm{d}q}{4\pi ^2} - \int _{\{\vert q \vert < \sqrt{\rho _0{\widehat{g}}_0}\}} \frac{\rho _0 {{\widehat{g}}}_q h_{q}}{2 \sqrt{q^4 + 2 \rho _0 q^2 {{\widehat{g}}}_q}} \frac{\textrm{d}q }{4 \pi ^2} \Big \vert \\&\le C {\widehat{v}}_0 \rho _0^2 {\widehat{g}}_0^2, \end{aligned}$$

from which the first result follows. The second comes from the fact that when $\delta =\delta _{0}$ we have

$$\begin{aligned} \delta _{0}^{-1}&=Y^{-1}+\vert \log Y\vert +O(Y\vert \log Y\vert ^{2}),\\ \log \delta _{0}&=\log Y +\log (1-Y\vert \log Y\vert ), \end{aligned}$$

providing that

4.21 $$\begin{aligned} \vert \delta _{0}^{-1}-Y^{-1}+\log \delta _{0}\vert \le C. \end{aligned}$$

$$\square $$

Now we have all necessary ingredients to conclude the proof of Theorem 4.1.

#### Proof of Theorem 4.1

We take the trial state $\Phi$ defined in Sect. 4.1, which has the expected bounds on number of particles from Lemma 4.5. The energy of $\Phi$ is bounded by Lemma 4.7 together with Lemma 4.8, and using $\delta _0\ge \frac{1}{2}Y$ we find

$$\begin{aligned} \frac{\langle {\mathcal {H}}_{v} \rangle _\Phi }{\vert \Lambda _\beta \vert }&\le \frac{\rho ^2}{2} {{\widehat{g}}}_0 + \frac{1}{2} \int \Big ( \sqrt{p^4 + 2 \rho _0 p^2 {{\widehat{g}}}_p} -p^2 - \rho _0 {{\widehat{g}}}_p+ \rho _0^2\frac{{{\widehat{g}}}^2_p - {{\widehat{g}}}_0^2 \mathbb {1}_{\{\vert p\vert \le \ell _{\delta }^{-1}\}}}{2p^2} \Big )\frac{\textrm{d}p }{4 \pi ^2} \\&\quad + \rho _{0}^{2}\delta ^{2}{\widehat{v}}_{0} \Big (\frac{1}{\delta }-\frac{1}{Y}+\log \delta \Big )+\rho _{0}^{2}\delta ^{2}{\widehat{v}}_{0} \Big (\frac{1}{\delta }-\frac{1}{Y}+\log \delta \Big )^{2}\\ {}&\quad +C \rho ^2 \delta ({\widehat{v}}_0 - {{\widehat{g}}}_0)+ C {\widehat{v}}_0 \rho _0^2 {\widehat{g}}_0^2. \end{aligned}$$

Now this integral can be estimated by Proposition C.3 and using $\rho -\rho _0\le C\rho Y$ we find

4.22 $$\begin{aligned} \frac{\langle {\mathcal {H}}_{v} \rangle _\Phi }{\vert \Lambda _\beta \vert }&\le \frac{\rho ^2}{2} {{\widehat{g}}}_0 + 4 \pi \rho ^2 \delta ^2 \Big ( \frac{1}{\delta } - \frac{1}{Y} + \log \delta + \Big ( \frac{1}{2} + 2 \Gamma + \log \pi \Big ) \Big ) \nonumber \\&\quad +\frac{1}{2} \rho ^{2}\delta ^{2}{\widehat{v}}_{0} \Big \vert \frac{1}{\delta }-\frac{1}{Y}+\log \delta \Big \vert +\frac{1}{2}\rho ^{2}\delta ^{2}{\widehat{v}}_{0} \Big (\frac{1}{\delta }-\frac{1}{Y}+\log \delta \Big )^{2} \nonumber \\&\quad +C \rho ^2 \delta ({\widehat{v}}_0 - {{\widehat{g}}}_0)+ C {\widehat{v}}_0 \rho ^2 {\widehat{g}}_0^2. \end{aligned}$$

Finally, with ${{\widehat{g}}}_0 = 8 \pi \delta$ and the specific choice $\delta = \delta _0$ we deduce

4.23 $$\begin{aligned} \frac{\langle {\mathcal {H}}_{v} \rangle _\Phi }{\vert \Lambda _\beta \vert }&\le 4\pi \rho ^2 \delta _0 \Big (1 + \Big (2\Gamma + \frac{1}{2} + \log (\pi ) \Big ) \delta _0 \Big )+ C \rho ^2 \delta _0({\widehat{v}}_0-{\widehat{g}}_0)+C \rho ^2 \delta ^{2}_0{\widehat{v}}_0. \end{aligned}$$

$$\square $$

#### Remark 4.9

In the case of the spherical measure potential (3.40) (with the choice of *b* given in (2.5)), one can see that the upper bound (4.22) is minimized (to the available energy precision) by the choice $\delta = \delta _0$. Indeed, even though the first three terms suggest to choose the smallest $\delta$ possible, including the remaining contributions yields a minimizer of the form

4.24 $$\begin{aligned} \delta = Y (1 + cY\vert \log Y \vert ). \end{aligned}$$

Notice that first line in (4.22) is independent of the choice of *c* to our precision. We pick for simplicity $c=-1$ to obtain our $\delta _{0}$ providing useful cancelations, see (4.21). This also fixes the value of $\widetilde{R}=ae^{\frac{1}{2\delta }}$.

## General Upper Bound

In this section we prove Theorem 2.2, using the results of Sect. 4. We let $\beta \ge \frac{3}{2}$ be given and we work on the box $\Lambda _\beta = [ - \frac{L_\beta }{2} , \frac{L_\beta }{2} ]^2$ of size $L_\beta = \rho ^{- \frac{1}{2}} Y^{- \beta }$. Moreover, the number of particles at density $\rho$ is $N= Y^{- 2 \beta }$.

### Trial state

Let *v* be a non-negative measurable and radial potential with scattering length *a* and ${{\,\mathrm{\textrm{supp}}\,}}(v) \subset B(0,R)$, with $\rho R^2 \le Y^{2 \beta +2}$. We consider $\varphi _b$ the associated scattering solution normalized at length $b = \rho ^{-1/2} Y^{\beta + \frac{1}{2}}$. In other words $\varphi _{b} = 2 \delta _\beta \varphi ^{(0)}$ with $\delta _\beta = \frac{1}{2} \log \left( b / a \right) ^{-1}$, see (3.3). Note that $R \ll b$. Let $f=\min \left( 1,\varphi _b\right)$ be the truncated scattering solution. It satisfies

5.1 $$\begin{aligned} -\Delta f (x) + \frac{1}{2} v(x) f(x) = 0 \quad \text {on } B(0,b) , \end{aligned}$$

and is normalized such that $f(x) = 1$ for $\vert x \vert \ge b$. We define a grand canonical trial state as

5.2 $$\begin{aligned} \Psi = \sum _{n \ge 0} \Phi _n F_n \in {\mathscr {F}}_s\big (L^{2}\left( \Lambda _{\beta }\right) \big ) \end{aligned}$$

where $\Phi = \sum _n \Phi _n \in {\mathscr {F}}_s\big (L^{2}\left( \Lambda _{\beta }\right) \big )$ is a quasi-free state defined in (4.3) and $F_n$ is the Jastrow factor

5.3 $$\begin{aligned} F_n(x_1, \ldots , x_n) = \prod _{1 \le i < j \le n} f(x_i -x_j) . \end{aligned}$$

We will use the notation $f(x_i -x_j)=f_{ij}$ and $\nabla f (x_i - x_j) = \nabla f_{ij}$. Finally note that

5.4 $$\begin{aligned} \nabla _{i}F_n(x_1, \ldots , x_n)=\sum _{\begin{array}{c} j=1\\ j\ne i \end{array}}^{n}\nabla _{i}f_{ij}\frac{F_n}{f_{ij}}. \end{aligned}$$

#### Remark 5.1

To estimate the energy of $\Psi$ we use the bound

5.5 $$\begin{aligned} 1\ge \prod _{1 \le i<j \le n} f(x_i-x_j)^2 \ge 1 - \sum _{1 \le i<j \le n} (1- f(x_i-x_j)^2). \end{aligned}$$

A similar trial state is used in [22] in 3 dimensions but there it is necessary to expand the product (5.5) to one order higher to be able to reach the LHY precision. This substantially complicates the estimates in that case.

### Reduction to a soft potential

In this section we prove that the energy $\langle \Psi , {\mathcal {H}}_v \Psi \rangle$ can be bounded by $\langle \Phi , {\mathcal {H}}_{{\widetilde{v}}} \Phi \rangle$ where ${\widetilde{v}}$ is a nicer potential. This is the effect of the Jastrow factor $F_n$, and we are thus reduced to optimizing the choice of the quasi-free state $\Phi$ according to the potential ${\widetilde{v}}$.

#### Lemma 5.2

Consider the radial potential $\widetilde{v}(x) = 2 f'(b) \delta _{\{\vert x \vert = b\}}$ (with $f'$ being understood as the radial derivative). Then the state $\Psi$ defined in (5.2) satisfies

$$\begin{aligned} \langle \Psi , {\mathcal {H}}_v \Psi \rangle \le \langle \Phi , {\mathcal {H}}_{{\widetilde{v}}} \Phi \rangle - \langle \Phi , {\mathcal {R}} \Phi \rangle ,\end{aligned}$$

where $${\mathcal {R}}= \oplus _n {\mathcal {R}}_n$$ with

$$\begin{aligned} {\mathcal {R}}_n = \sum _{\{i,j,k\}} \frac{\nabla f_{ij}}{f_{ij}} \cdot \frac{\nabla f_{ik}}{f_{ik}} F_n^2, \end{aligned}$$

where we introduced the notation

$$\begin{aligned}\{i,j,k\}=\{\text {set of pairwise distinct indices}\,\, i, j, k \,\,\text {running from}\,\, 1\,\, \text {to}\,\, n\}. \end{aligned}$$

#### Proof

The energy of the *n*-th sector state is

5.6 $$\begin{aligned} \langle \Psi _n , {\mathcal {H}}_n \Psi _n \rangle&= \sum _{i=1}^n \int _{\Lambda ^n} \big (F_n^2 \vert \nabla _i \Phi _n \vert ^2 + \vert \nabla _i F_n \vert ^2 \Phi _n^2 + 2 F_n \nabla _i F_n \cdot \Phi _n \nabla _i \Phi _n \big )\textrm{d}x \nonumber \\&\quad + \sum _{1\le i<j\le n} \int _{\Lambda ^n} v(x_i-x_j) F_n^2 \Phi _n^2 \textrm{d}x. \end{aligned}$$

The second term in (5.6) can be written via (5.4) as

5.7 $$\begin{aligned} \sum _{i=1}^n \int _{\Lambda ^n} \vert \nabla _i F_n \vert ^2 \Phi _n^2 \textrm{d}x = \sum _{i\ne j} \int _{\Lambda ^n} \vert \nabla f_{ij} \vert ^2 \frac{F_n^2}{f_{ij}^2} \Phi _n^2 \textrm{d}x + \sum _{\{i,j,k\}} \int _{\Lambda ^n} \frac{\nabla f_{ij}}{f_{ij}} \cdot \frac{\nabla f_{ik}}{f_{ik}} F_n^2 \Phi _n^2 \textrm{d}x . \end{aligned}$$

Note that, in the first part of (5.7) the integration in $x_i$ is only supported on the ball $\vert x_i - x_j \vert \le b$, because $f_{ij} =1$ outside this ball. We integrate by parts on this ball to find

5.8 $$\begin{aligned}&\sum _{i=1}^n \int _{\Lambda ^n} \vert \nabla _i F_n \vert ^2 \Phi _n^2 \textrm{d}x \\&= - \sum _{i \ne j} \int _{\{\vert x_{i}-x_{j}\vert \le b\}} \Delta f_{ij} \frac{F_n^2}{f_{ij}} \Phi _n^2 \textrm{d}x - \sum _{i \ne j} \int _{\Lambda ^n} \nabla f_{ij} \frac{F_n^2}{f_{ij}} \cdot \nabla _i (\Phi _n^2) \textrm{d}x \nonumber \\&\quad - \sum _{\{i,j,k\}} \int _{\Lambda ^n} \frac{\nabla f_{ij}}{f_{ij}} \cdot \frac{\nabla f_{ik}}{f_{ik}} F_n^2 \Phi _n^2 \textrm{d}x + \sum _{i \ne j} \int _{\Lambda ^{n-1}} \int _{\{\vert x_i - x_j \vert = b\}} \partial _r f(b) F_n^2 \Phi _n^2 \textrm{d}x_i \textrm{d}{\hat{x}}_i, \nonumber \end{aligned}$$

where ${\hat{x}}_i=(x_{1}, \ldots x_{i-1} , x_{i+1}, \ldots , x_n)$. The second term in the right hand side of (5.8) is precisely $-2F_n \nabla _i F_n \cdot \Phi _n \nabla _i \Phi _n$ thanks to (5.4). We use the scattering equation (5.1) to transform

5.9 $$\begin{aligned} \sum _{i \ne j} \int _{\{\vert x_{i}-x_{j}\vert \le b\}} \Delta f_{ij} \frac{F_n^2}{f_{ij}} \Phi _n^2 \textrm{d}x =\sum _{1\le i<j\le n} v(x_i-x_j) F_n^2 \Phi _n^2 \textrm{d}x, \end{aligned}$$

in (5.8) (note that there is no half factor because the sum is on $i<j$). Using (5.8) and (5.9) in (5.6) we deduce

5.10 $$\begin{aligned} \langle \Psi _n , {\mathcal {H}}_n \Psi _n \rangle&= \sum _{i=1}^n \int _{\Lambda ^n} F_n^2 \vert \nabla _i \Phi _n \vert ^2 + 2 \sum _{i<j} \int _{\Lambda ^{n-1}} \int _{\{\vert x_i - x_j \vert = b\}} \partial _r f(b) F_n^2 \Phi _n^2 \textrm{d}x_i \textrm{d}{\hat{x}}_i \nonumber \\ {}&\quad - \sum _{\{i,j,k\}} \int _{\Lambda ^n} \frac{\nabla f_{ij}}{f_{ij}} \cdot \frac{\nabla f_{ik}}{f_{ik}} F_n^2 \Phi _n^2 \textrm{d}x. \end{aligned}$$

In the first two terms we bound $F_n$ by 1, and the last one we consider as a remainder. Thus,

$$\begin{aligned} \langle \Psi _n , {\mathcal {H}}_n \Psi _n \rangle \le \int _{\Lambda ^n} \vert \nabla \Phi _n \vert ^2 + \sum _{i<j} \int _{\Lambda ^n} \Phi _n^2 \widetilde{v}(x_i-x_j) \textrm{d}x - {\mathcal {R}}_n .\end{aligned}$$

$$\square $$

We comment here how in the proof we used nowhere that $\Phi$ is a quasi-free state, therefore the lemma holds true for more general $\Phi \in {\mathscr {F}}_s(L^2(\Lambda _{\beta }))$.

### Number of particles in our trial state

Now for $\Phi$ we choose the quasi-free state given by Theorem 4.1, applied to the potential ${\widetilde{v}}$. We recall that $\Phi = W_{N_0} T_\nu \Omega$ is defined in (4.3), and $\Psi$ in (5.2). In this section we prove the following two lemmas, giving estimates on the norm of $\Psi$ and the average number of particles in $\Psi$. The idea is to use the properties of $F_n$ to derive the bounds on $\Psi _n = F_n \Phi _n$ from the bounds on the quasi-free state $\Phi$.

#### Lemma 5.3

There is a $C>0$, independent of *v* and $\rho$, such that

$$\begin{aligned}\Vert \Psi \Vert ^2 \ge \Vert \Phi \Vert ^2 \Big ( 1 - C N Y^{2\beta + 2} \Big ) .\end{aligned}$$

#### Proof

The norm of our trial state is bounded from below by

5.11 $$\begin{aligned} \Vert \Psi \Vert ^2&= \sum _{n \ge 0} \int _{\Lambda ^n} F_n^2(x) \Phi _n^2(x) \textrm{d}x \nonumber \\ {}&\ge \sum _{n \ge 0} \Big ( \int _{\Lambda ^n} \Phi _n^2 \textrm{d}x - \sum _{1\le i<j\le n} \int _{\Lambda ^n} (1 - f(x_i-x_j)^2) \Phi _n^2(x) \textrm{d}x \Big ), \end{aligned}$$

where we used the inequality

5.12 $$\begin{aligned} \prod _{1 \le i<j \le n} f(x_i-x_j)^2 \ge 1 - \sum _{1 \le i<j \le n} (1- f(x_i-x_j)^2). \end{aligned}$$

The second term is the 2-body interaction potential energy of $\Phi$, thus we can write it as

5.13 $$\begin{aligned} \sum _{n \ge 0} \sum _{1 \le i <j \le n} \int (1-f(x_i-x_j)^2) \Phi _n^2 \textrm{d}x&= \frac{1}{2 \vert \Lambda _{\beta } \vert } \sum _{p,q,r} \widehat{(1-f^2)}_r \langle a_{q}^* a_{p+r}^* a_{q+r} a_p \Phi , \Phi \rangle \nonumber \\&\le \frac{\widehat{(1-f^2)}_0}{2 \vert \Lambda _{\beta } \vert } \sum _{p,q,r} \langle a_{q}^* a_{p+r}^* a_{q+r} a_p \Phi , \Phi \rangle . \end{aligned}$$

Since $\Phi = W_{N_0} T_\nu \Omega$ is a quasi-free state we can estimate this term as already done in (4.11). We first conjugate by $W_{N_0}$ which amounts to change the $a_{0}$’s into $N_0 \le N$. Together with Lemma 4.4 and Wick’s theorem we deduce

5.14 $$\begin{aligned} \sum _{p,q,r} \langle a_{q}^* a_{p+r}^* a_{q+r} a_p \rangle _\Phi&\le CN^{2} +\sum _{\begin{array}{c} p\ne 0,q\ne 0,r\ne 0\\ p+r\ne 0, q+r\ne 0 \end{array}} \langle a_{q}^* a_{p+r}^* a_{q+r} a_p \rangle _\Phi . \end{aligned}$$

Then we use again Wick’s Theorem to estimate the remaining sum, which is then bounded by $C N^2$ by Lemma 4.4. Thus Eq. (5.13) gives

5.15 $$\begin{aligned} \sum _{n \ge 0} \sum _{1 \le i <j \le n} \int (1-f(x_i-x_j)^2) \Phi _n^2 \textrm{d}x \le C \frac{N^2}{\vert \Lambda _{\beta } \vert } \int _{\Lambda } (1 - f(x)^2) \textrm{d}x \, \Vert \Phi \Vert ^2 .\qquad \end{aligned}$$

Using $\frac{\textrm{d}}{\textrm{d}r} [ r^2 \log \left( \frac{r}{a} \right) ^2 - r^2 \log \left( \frac{r}{a} \right) + \frac{r^2}{2} ] = 2 r \log \left( \frac{r}{a} \right) ^2$ and $a\le R\le b$ we have that

5.16 $$\begin{aligned} \int _\Lambda (1-f(x)^2) \textrm{d}x&= 2\pi \int _R^b \bigg ( 1 - \frac{\log \left( \frac{r}{a} \right) ^2}{\log \left( \frac{b}{a} \right) ^2} \bigg ) r \textrm{d}r + 2 \pi \int _0^R (1 - f(r)^2) r\textrm{d}r \nonumber \\ {}&\le C \frac{b^2}{\log \left( \frac{b}{a} \right) } + C R^2 \le C \rho ^{-1} Y^{2 \beta + 2}, \end{aligned}$$

where we used $\rho R^2 \le Y^{2 \beta + 2}$ and $b^2 = \rho ^{-1} Y^{2 \beta + 1}$. We use this last bound in (5.15) and (5.11) to get

$$\begin{aligned} \Vert \Psi \Vert ^2 \ge \Vert \Phi \Vert ^2 \big ( 1 - C N Y^{2 \beta + 2} \big ) . \end{aligned}$$

$$\square $$

#### Lemma 5.4

There is a $C>0$ independent of $\rho$ and *v* such that,

$$\begin{aligned} \langle \Psi , {\mathcal {N}} \Psi \rangle \ge N(1- CY^2)\Vert \Psi \Vert ^2 , \qquad \langle \Psi , {\mathcal {N}}^2 \Psi \rangle \le C N^2 \Vert \Psi \Vert ^2. \end{aligned}$$

#### Proof

First we have by Lemmas 4.5 and 5.3 that

$$\begin{aligned} \langle \Psi , {\mathcal {N}} ^2\Psi \rangle = \sum _{n \ge 0} n^{2} \int _{\Lambda ^n} F_n^2 \Phi _n^2 \textrm{d}x \le \sum _{n \ge 0} n^2 \Vert \Phi _n \Vert ^2 =\langle \Phi , {\mathcal {N}}^2 \Phi \rangle \le C N^2 \Vert \Psi \Vert ^2. \end{aligned}$$

For the bound on $\langle {\mathcal {N}} \rangle _{\Psi }$ we use the same idea as in the proof of Lemma 5.3. From inequality (5.12) we deduce

5.17 $$\begin{aligned} \langle \Psi , {\mathcal {N}}\Psi \rangle&= \sum _{n \ge 0} n \int _{\Lambda ^n} F_n^2 \Phi _n^2 \textrm{d}x \nonumber \\&\ge \sum _{n\ge 0} n \Big ( \int _{\Lambda ^n} \Phi _n^2 \textrm{d}x - \sum _{i<j} \int _{\Lambda ^n} (1-f(x_i-x_j)^2) \Phi _n^2\textrm{d}x \Big ). \end{aligned}$$

In the second term we recognize a number operator and a 2-particles interaction energy, which can be rewritten as

$$\begin{aligned} \sum _{n \ge 0} n \sum _{i<j} \int _{\Lambda ^n} (1-f(x_i-x_j)^2) \Phi _n^2 \textrm{d}x = \sum _{k,p,q,r \in \Lambda ^*} \frac{\widehat{(1 - f^2)}_r}{2L_\beta ^2} \langle a_k^* a_k a_{p+r}^* a_q^* a_{q+r} a_p \Phi , \Phi \rangle . \end{aligned}$$

We can compute this term using the same techniques as for (5.13), i.e., extract the $a_{0}$’s and then apply Wick’s Theorem yielding many terms of the form $A_1 A_2 A_3$ with $A_i \in \lbrace \langle a_0^\dagger a_0 \rangle _\Phi , \sum _{p \ne 0} \alpha _p , \sum _{p \ne 0} \gamma _p \rbrace$ (see (4.5)). These terms are bounded by $N^3$ by Lemma 4.4. Thus

$$\begin{aligned}\sum _{n \ge 0} n \sum _{i<j} \int _{\Lambda ^n} (1-f(x_i-x_j)^2) \Phi _n^2 \textrm{d}x \le C \frac{ N^3 }{L_\beta ^2} \int (1-f(x)^2 )\textrm{d}x \Vert \Phi \Vert ^2. \end{aligned}$$

Now we use the inequality (5.16) to bound the right hand side of the quantity above and plug it in (5.17) to obtain

$$\begin{aligned} \langle \Psi , {\mathcal {N}} \Psi \rangle _{} \ge (N - C N^2 Y^{2 \beta + 2} ) \Vert \Psi \Vert ^2= N (1-C Y^2)\Vert \Psi \Vert ^2, \end{aligned}$$

where in the equality used that $N=Y^{-2\beta }$. $\square$

### Remainder term

Here we prove that the remainder term in Lemma 5.2 is indeed small.

#### Lemma 5.5

There is a $C>0$ independent of *v* and $\rho$ such that

$$\begin{aligned} \vert \langle \Phi , {\mathcal {R}} \Phi \rangle \vert \le C L_\beta ^{2} \rho ^{2} Y^{2 \beta + 2} \Vert \Phi \Vert ^{2}. \end{aligned}$$

#### Proof

The remainder term can be bounded by

$$\begin{aligned} \vert \langle \Phi , {\mathcal {R}} \Phi \rangle \vert \le \sum _{n \ge 3} \sum _{\{i,j,k\}} \int _{\Lambda ^n} W(x_i-x_k) W(x_i - x_j) \Phi _n^2 \textrm{d}x , \end{aligned}$$

where $W(x) = \vert f(x) \nabla f(x) \vert$. This is a three-body interaction potential, which can be rewritten in second quantization as

$$\begin{aligned} \vert \langle \Phi , {\mathcal {R}} \Phi \rangle \vert \le \frac{1}{\vert \Lambda _\beta \vert ^2} \sum _{p,q,r,k,\ell \in \Lambda ^*} {\widehat{W}}_k {\widehat{W}}_\ell \langle a^*_{p+\ell + k} a^*_{q-k} a^*_{r-\ell } a_r a_q a_p \rangle _{\Phi } \, \Vert \Phi \Vert ^2 . \end{aligned}$$

We can again use Wick’s Theorem to estimate this part, and since Lemma 4.4 provides

$$\begin{aligned}\max \Big \{\sum _{p \ne 0} \alpha _p , \sum _{p \ne 0} \gamma _p\Big \} \le N,\end{aligned}$$

we find

5.18 $$\begin{aligned} \vert \langle \Phi , {\mathcal {R}} \Phi \rangle \vert \le C \frac{N^3}{\vert \Lambda _\beta \vert ^2} {{\widehat{W}}}_0 ^2 \, \Vert \Phi \Vert ^2. \end{aligned}$$

Now since $f(x) = \log \left( \frac{b}{a} \right) ^{-1} \log \Big ( \frac{\vert x \vert }{a} \Big )$ outside the support of *v* and is radially increasing, we have

$$\begin{aligned} {\widehat{W}}_0&\le 2\pi \int _R^b \frac{\log \left( \frac{r}{a} \right) }{\log \left( \frac{b}{a} \right) ^2} \textrm{d}r + 2\pi \int _0^R f(r) f'(r) r \textrm{d}r \\&\le C \frac{b}{\log \left( \frac{b}{a} \right) } + C R\Big (\frac{ \log \left( \frac{R}{a} \right) }{\log \left( \frac{b}{a} \right) }\Big )^{2} \le C \rho ^{-1/2} Y^{\beta + 1} , \end{aligned}$$

where we used $\vert \log \left( \frac{b}{a} \right) \vert ^{-1} \le Y$ and $\vert \log \left( \frac{R}{a} \right) \vert \le \vert \log \left( \frac{b}{a} \right) \vert$. Inserting this bound in (5.18) we get the result. $\square$

### Conclusion: Proof of Theorem 2.2

Using Lemmas 5.2, 5.3 and 5.5 we know that our trial state $\Psi$ satisfies

5.19 $$\begin{aligned} \langle {\mathcal {H}}_v \rangle _\Psi \le \Big ( \langle {\mathcal {H}}_{{\widetilde{v}}} \rangle _\Phi + C L_\beta ^2 \rho ^2 Y^{2\beta + 2} \Big ) \Big ( 1 + CNY^{2\beta + 2} \Big ). \end{aligned}$$

For $\Phi$ we choose the quasi-free state given by Theorem 4.1 applied to the soft potential ${\widetilde{v}}$. Recall the definition of $\widetilde{g}$ from Lemma 3.10. We deduce that

5.20 $$\begin{aligned} \frac{1}{\vert \Lambda _\beta \vert } \langle {\mathcal {H}}_{\widetilde{v}} \rangle _\Phi \le 4\pi \rho ^2 \delta _0 \Big (1 + \Big (2\Gamma + \frac{1}{2} + \log (\pi ) \Big ) \delta _0 \Big ) + C \rho ^2 \delta _0\big (\widehat{\widetilde{v}}(0)-\widehat{\widetilde{g}}(0)\big )+C\rho ^2 \delta ^{2}_0\widehat{{\widetilde{v}}}(0). \end{aligned}$$

From Lemma 3.10 we have

$$\begin{aligned} \widehat{{\widetilde{v}}}(0)=\frac{4\pi }{\log b/a}\quad \text {and}\quad \widehat{\widetilde{g}}(0)=\frac{4\pi }{\log \widetilde{R}/a}= 8\pi \delta _0, \end{aligned}$$

where we recall from (3.3) that $\widetilde{R} = a e^{\frac{1}{2\delta _0}}$. Therefore, remembering the choices $b=\rho ^{-1/2} Y^{1/2+\beta }$ and $\beta \ge 3/2$, we can estimate $(\widehat{{\widetilde{v}}}(0)-\widehat{\widetilde{g}}(0))\le CY^{2}\log Y$ and we get

5.21 $$\begin{aligned} \frac{1}{\vert \Lambda _\beta \vert } \langle {\mathcal {H}}_{\widetilde{v}} \rangle _\Phi \le 4\pi \rho ^2 \delta _0 \Big (1 + \Big (2\Gamma + \frac{1}{2} + \log (\pi ) \Big ) \delta _0 \Big ) + \beta C \rho ^2 \delta _0^3\vert \log (\delta _0)\vert +C\rho ^2 \delta ^{3}_0. \end{aligned}$$

We insert this into (5.19) together with $N = \rho L_\beta ^2 = Y^{-2\beta }$ and $Y\le 2\delta _0$, which concludes the proof of Theorem 2.2. $\square$

## Localization to Large Boxes for the Lower Bound

In this section we reduce the proof of Theorem 2.3 to an analogous statement localized to a box of size $\ell$ defined in (6.6), namely Theorem 6.7.

### Grand canonical ensemble

We rewrite the Hamiltonian in a grand canonical setting to approach the problem in the Fock space description. To emphasize the fact that the density parameter appears through a chemical potential in this setting, we introduce the notation $\rho _\mu >0$ as new parameter. The corresponding *Y* will be $Y = \vert \log ( \rho _\mu a^2) \vert ^{-1}$ and we fix $\delta$ to be

6.1 $$\begin{aligned} \delta = \delta _{\mu }, \qquad \delta _{\mu } := \frac{1}{ |\log (\rho _{\mu } a^2 |\log (\rho _{\mu } a^2)|^{-1})|}. \end{aligned}$$

This corresponds to normalizing the scattering solution at length $\widetilde{R} = (\rho _{\mu } Y)^{-1/2}$ in (3.3). With this choice we recall the definition (3.4) of *g*. This definition is analogous to the one of $\delta _0$ (1.5) but with $\rho _{\mu }$ in place of $\rho$. We are going to choose, a posteriori, $\rho _{\mu } = \rho$ which implies $\delta _{\mu } = \delta _0$.

That this choice of $\delta$ is optimal follows by an evaluation of the relevant integral giving the constant in the correction term in (1.4). Please see Remark C.4 for the evaluation of this integral and the discussion of the optimal choice.

We consider the operator ${\mathcal {H}}_{\rho _{\mu }}$ acting on the symmetric Fock space ${\mathscr {F}}_s(L^2(\Omega ))$ and commuting with the number operator, whose action on the *N*-bosons space is

6.2 $$\begin{aligned} {\mathcal {H}}_{\rho _{\mu },N}&= H(N,L) - 8 \pi \delta \rho _{\mu } N = \sum _{j=1}^N -\Delta _j + \sum _{i<j} v(x_i-x_j)- 8 \pi \delta \rho _{\mu } N \nonumber \\&= \sum _{j=1}^N \Big (-\Delta _j - \rho _{\mu } \int _{{\mathbb {R}}^2} g(x_j-y)\,\textrm{d}y\Big ) + \sum _{i<j} v(x_i-x_j). \end{aligned}$$

We define the ground state energy density of ${\mathcal {H}}_{\rho _{\mu }}$:

6.3 $$\begin{aligned} e_0(\rho _{\mu }) := \lim _{|\Omega |\rightarrow +\infty } \frac{1}{|\Omega |} \inf _{\Psi \in {\mathscr {F}}_s(L^2(\Omega ))\setminus \{0\}} \frac{\langle \Psi \,|\, {\mathcal {H}}_{\rho _{\mu }} \,|\,\Psi \rangle }{\Vert \Psi \Vert ^2}. \end{aligned}$$

In the rest of the paper we prove the following lower bound on $e_0(\rho _\mu )$.

#### Theorem 6.1

There exists *C*, $\eta > 0$ such that the following holds. Let $\rho _\mu > 0$ and $v\in L^1(\Omega )$ be a positive, spherically symmetric potential with scattering length *a* and ${{\,\mathrm{\textrm{supp}}\,}}(v) \subset B(0,R)$ such that $\Vert v \Vert _1 \le Y^{-1/8}$ and $R \le \rho _\mu ^{-1/2}$. Then, if $\rho _\mu a^2 \le C^{-1}$, we have, for any $\rho _\mu > 0$,

6.4 $$\begin{aligned} e_0(\rho _{\mu }) \ge - 4 \pi \rho _{\mu }^2 \delta \Big (1 - \Big ( 2 \Gamma + \frac{1}{2} + \log \pi \Big ) \delta \Big ) - C \rho _{\mu }^2 \delta ^{2+\eta }. \end{aligned}$$

We now show that Theorem 6.1 implies the main lower bound Theorem 2.3.

#### Proof of Theorem 2.3

We start by reducing the problem to a potential which is $L^1$ and compactly supported. For a given *v* satisfying the assumptions of Theorem 2.3, we apply Theorem 3.6 with $T = (4 \pi Y)^{-1/8}$, $R= \rho ^{-1/2}$ and $\varepsilon = 1$. This provides us with a potential $\widetilde{v} = v_{T,R,\epsilon }$ to which we can apply Theorem 6.1. Then for this new potential we use the ground state of ${\mathcal {H}}_N$ as a trial function for ${\mathcal {H}}_{\rho _{\mu }}$ and get

$$\begin{aligned} e^{\text {2D}}(\rho , {\widetilde{v}} )&\ge e_0(\rho _{\mu } , {\widetilde{v}} ) + 8 \pi {{\widetilde{\delta }}} \rho \rho _{\mu } \\&\ge - 4 \pi \rho _{\mu }^2 {{\widetilde{\delta }}} \Big (1 - \Big (2 \Gamma + \frac{1}{2} + \log \pi \Big ) {{\widetilde{\delta }}} \Big ) - C \rho _{\mu }^2 {{\widetilde{\delta }}}^{2+\eta } + 8 \pi {{\widetilde{\delta }}} \rho \rho _{\mu }, \end{aligned}$$

where ${{\widetilde{\delta }}} = \frac{1}{\vert \log ( \rho {\widetilde{a}}^2 \vert \log (\rho {\widetilde{a}}^2) \vert ^{-1} ) \vert }$ and ${\widetilde{a}}$ is the scattering length of ${\widetilde{v}}$. Since ${\widetilde{v}} \le v$ we have $e^{\text {2D}}(\rho ,v) \ge e^{\text {2D}}(\rho , {\widetilde{v}})$. Moreover, by Eq. (3.23) we can change ${{\widetilde{\delta }}}$ into $\delta$ up to an error of order

6.5 $$\begin{aligned} \frac{1}{\log \left( \frac{R}{a}\right) ^2 T} + \frac{1}{\log \left( \frac{R}{a}\right) ^2} \int _{\{\vert x \vert > R\}} v(x) \log \Big ( \frac{ \vert x \vert }{a} \Big )^2 \textrm{d}x \le C \delta ^{2+\min \left( \frac{1}{8}, \eta _1 \right) }. \end{aligned}$$

Choosing $\rho _{\mu } = \rho$ concludes the proof. $\square$

### Reduction to large boxes

We now make use of the sliding localization technique developed in [28] to reduce the proof of Theorem 2.3 to a localized problem in a large box $\Lambda \subset \Omega$. We introduce the length scale

6.6 $$\begin{aligned} \ell := K_{\ell }\, \rho _{\mu }^{-1/2} Y^{-\frac{1}{2}}, \end{aligned}$$

where $K_{\ell } \gg 1$ is a parameter fixed in “Appendix H”, and we carry out the analysis in the large box

6.7 $$\begin{aligned} \Lambda := \Big [-\frac{\ell }{2}, \frac{\ell }{2}\Big ]^2. \end{aligned}$$

For any $u \in {\mathbb {R}}^2$, we denote by

6.8 $$\begin{aligned} \Lambda _u := \ell u + \Lambda \end{aligned}$$

the translated large box. Let us introduce the localization functions: the sharp characteristic function

6.9 $$\begin{aligned} \theta _u := \mathbb {1}_{\Lambda _u} \end{aligned}$$

and the regular one: let $\chi \in C_0^{M}({\mathbb {R}}^2)$, for $M \in {\mathbb {N}}$ with ${{\,\mathrm{\textrm{supp}}\,}}\chi = [-\frac{1}{2},\frac{1}{2}]^2$ be the spherically symmetric function defined in “Appendix F”, and

6.10 $$\begin{aligned} \chi _{\Lambda }(x) := \chi \Big ( \frac{x}{\ell }\Big ), \qquad \chi _u (x) := \chi _{\Lambda }(x-\ell u). \end{aligned}$$

The parameter *M* is fixed in “Appendix H”. Define the following projections on $L^2(\Lambda )$,

6.11 $$\begin{aligned} P := \ell ^{-2} | \mathbb {1}_{\Lambda } \rangle \langle \mathbb {1}_{\Lambda } |, \qquad Q := \mathbb {1}- P, \end{aligned}$$

i.e. *P* is the orthogonal projection in $L^2(\Lambda )$ onto the constant functions and *Q* is the orthogonal projection to the complement. Using these definitions, we define the following operators on ${\mathscr {F}}_s(L^2(\Lambda ))$ through their action on any *N*-particles sector:

6.12 $$\begin{aligned} n_0:= \sum _{j=1}^N P_j, \qquad n_{+} := \sum _{j=1}^N Q_j = N - n_0. \end{aligned}$$

The definition is based on the idea that low energy eigenstates of the system should concentrate in the constant function. Thus, $n_0$ counts the number of particles in the condensate and $n_{+}$ the number of particles excited out of the condensate.

We start by stating the result for the kinetic energy.

#### Lemma 6.2

(Kinetic energy localization). Let $-\Delta _u^{{\mathcal {N}}}$ denote the Neumann Laplacian in $\Lambda _u$ and $-\Delta$ the Laplacian on ${{\mathbb {R}}}^2$. If the regularity of $\chi$ is $M >5$ and the positive parameters $\varepsilon _{N}, \varepsilon _T, d, s, b$ are smaller than some universal constant, then for all $\ell >0$ we have

6.13 $$\begin{aligned} -\Delta \ge \int _{{\mathbb {R}}^2} {{\mathcal {T}}}_u \,\textrm{d}u, \end{aligned}$$

in terms of quadratic forms in $H^1({\mathbb {R}}^2)$, where

6.14 $$\begin{aligned} {\mathcal {T}}_u&:= \varepsilon _{N}(-\Delta _u^{{\mathcal {N}}}) + (1-\varepsilon _{N})({\mathcal {T}}_u^{\text {Neu,s}}+ {\mathcal {T}}_u^{\text {Neu,l}} + {\mathcal {T}}_u^{\text {gap}} + {\mathcal {T}}_u^{\text {kin}} ), \end{aligned}$$

with

6.15 $$\begin{aligned} {\mathcal {T}}_u^{\text {Neu,s}}&:= \frac{\varepsilon _T}{2(d\ell )^2}\frac{-\Delta _u^{{\mathcal {N}}}}{-\Delta _u^{{\mathcal {N}}} + (d\ell )^{-2}}, \end{aligned}$$

6.16 $$\begin{aligned} {\mathcal {T}}_u^{\text {Neu,l}}&:= \frac{b}{\ell ^2} Q_u, \end{aligned}$$

6.17 $$\begin{aligned} {\mathcal {T}}_u^{\text {gap}}&:= b \frac{\varepsilon _T}{(d\ell )^2} Q_u \mathbb {1}_{(d^{-2}\ell ^{-1}, + \infty )} (\sqrt{-\Delta }) Q_u, \end{aligned}$$

6.18 $$\begin{aligned} {\mathcal {T}}_u^{\text {kin}}&:= Q_u \chi _u \left\{ (1-\varepsilon _T)\left[ \sqrt{-\Delta } -\frac{1}{2s\ell }\right] _+^2 + \varepsilon _T \left[ \sqrt{-\Delta } -\frac{1}{2 d s\ell }\right] _+^2 \right\} \chi _u Q_u. \end{aligned}$$

#### Proof

The proof is identical to the one of [28, Lemma 3.7] and its adaptation to our context in [19, Lemma 6.4], which are independent of dimension. $\square$

#### Remark 6.3

The kinetic energy is composed of several terms which have to remedy some problems related to the main kinetic energy term and play the following roles:

- ${\mathcal {T}}_u^{\text {kin}}$ is the main kinetic energy term;
- $-\Delta ^{{\mathcal {N}}}$ is the Neumann Laplacian and compensates the loss of ellipticity at the boundary caused by the localization function $\chi$ in ${\mathcal {T}}^{\text {kin}}_u$;
- ${\mathcal {T}}_u^{\text {Neu,s}}$ is the Neumann gap in the small box. Worth to remark is that, for large momenta, it behaves like a gap, while for small momenta its action is like a Neumann Laplacian;
- ${\mathcal {T}}_u^{\text {Neu,l}}$ is a fraction *b* of the Neumann gap in the large box. We don’t think of *b* as a parameter but as a fixed small constant. In the remaining we then choose and fix the value of *b*.
- ${\mathcal {T}}_u^{\text {gap}}$ is another spectral gap which we need in order to control the number of excitations with large momenta.

The localization of the potential energy relies on a direct calculation of the integral which can be found in [28, Proposition 3.1]. Assuming that $R \ell ^{-1}$ is sufficiently small, we can introduce the following localized potentials

6.19 $$\begin{aligned} W(x)&:= \frac{v(x)}{\chi *\chi (x/\ell )},&w(x,y)&:= \chi _{\Lambda } (x) W(x-y)\chi _{\Lambda }(y), \end{aligned}$$

6.20 $$\begin{aligned} W_1(x)&:= \frac{g(x)}{\chi *\chi (x/\ell )},&w_{1}(x,y)&:= \chi _{\Lambda } (x) W_1(x-y)\chi _{\Lambda }(y), \end{aligned}$$

6.21 $$\begin{aligned} W_2(x)&:= \frac{g(x) + g(x) \omega (x)}{\chi *\chi (x/\ell )},&w_{2}(x,y)&:= \chi _{\Lambda } (x) W_2(x-y)\chi _{\Lambda }(y), \end{aligned}$$

where we observe that $W, W_1, W_2$ and $w, w_1, w_2$ are localized versions of $v, g, (1+ \omega ) g$, respectively, defined in (3.4).

Furthermore, we introduce the translated versions for $u \in \Lambda$

6.22 $$\begin{aligned} w_{1,u}(x,y) = w_1(x-\ell u,y-\ell u) \end{aligned}$$

and similarly for $w_{2,u}$ and $w_{u}$. We are going to make use of the following approximation result. We recall the defintion of the lengthscale $\ell _{\delta }$ from (3.30), which, with our choice $\delta = \delta _{\mu }$ from (6.1) becomes

6.23 $$\begin{aligned} \ell _{\delta } = \frac{e^{\Gamma }}{2} \rho _{\mu }^{-1/2} Y^{-1/2}, \end{aligned}$$

and corresponds to the so-called healing length.

#### Lemma 6.4

There exists a universal constant $C>0$ such that, if $R \ell ^{-1} < C^{-1}$, we have

- $W_1$ can be approximated by *g* up to the following error
  6.24 $$\begin{aligned} 0 \le W_1(x) -g(x) \le C g(x) \frac{\min \{|x|^2,R^2\}}{\ell ^2}, \end{aligned}$$
  and in particular $\Vert W_1 \Vert _{L^1} \le 8\pi \delta (1 + C R^2 \ell ^{-2})$ due to (3.6).
- For any $h \in L^1({\mathbb {R}}^2)$ such that $h(x) = h(-x)$ and ${{\,\mathrm{\textrm{supp}}\,}}h \subseteq B(0,R)$,
  6.25 $$\begin{aligned} \bigg \vert h * \chi _{\Lambda }(x) - \chi _{\Lambda }(x) \int _{{\mathbb {R}}^2} \textrm{d}x\; h(x) \bigg \vert \le C\max _{i,j} \Vert \partial _i \partial _j \chi \Vert _{\infty } \frac{R^2}{\ell ^2} \Vert h\Vert _{L^1}. \end{aligned}$$
- It also holds
  6.26 $$\begin{aligned} \bigg \vert \frac{1}{(2\pi )^2} \int _{{\mathbb {R}}^2} dk\, \frac{{\widehat{W}}_1(k)^2 - {\widehat{W}}_1^2(0) \mathbb {1}_{\{|k|\le \ell _{\delta }^{-1}\}}}{2k^2} - \widehat{g \omega }(0)\bigg \vert \le C\frac{R^2}{\ell ^2}\delta . \end{aligned}$$
- It holds
  6.27 $$\begin{aligned} \bigg \vert \int _{{\mathbb {R}}^2} \frac{({\widehat{W}}_1(k) - {\widehat{g}}(k))^2 - ({\widehat{W}}_1(0) - {\widehat{g}}(0))^2\mathbb {1}_{\{|k| \le \ell _{\delta }^{-1}\}}}{2 k^2} \textrm{d}k \bigg \vert \le C\frac{R^4}{\ell ^4} \widehat{g \omega }(0).\qquad \end{aligned}$$

#### Proof

For (6.24) we use that the support of *g* is contained in the set $\{|x|<R\}$, therefore it is enough to give here our estimate. Using the symmetries of $\chi$, the normalization $\Vert \chi \Vert _2 = 1$ (Appendix F) and a Taylor expansion we see that

$$\begin{aligned} \left| 1 - \frac{1}{\chi * \chi (x/\ell )} \right|&\le \frac{1}{|\chi * \chi (x/\ell )|} \left| \int _{{\mathbb {R}}^2} \chi (y) [\chi (y) - \chi (x/\ell -y)] \right| \textrm{d}y \\&\le C \frac{|x|^2}{\ell ^2} \max _{i,j}\Vert \partial _i \partial _j \chi \Vert _{\infty }, \end{aligned}$$

which implies the first bound. (6.25) is proved similarily. For the bound (6.26), by the Lemma 3.8 we know that

6.28 $$\begin{aligned} (\widehat{g \omega })_0 = \frac{1}{(2\pi )^2} \int _{{\mathbb {R}}^2} \frac{{\widehat{g}}_k^2 - {\widehat{g}}_0^2 \mathbb {1}_{\{|k|\le \ell _{\delta }^{-1}\}}}{2k^2}\,\textrm{d}k, \end{aligned}$$

and using (3.28) for both the expressions of *W* and *g* we get

$$\begin{aligned} \frac{1}{(2\pi )^2}\bigg \vert \int _{{\mathbb {R}}^2} \,&\frac{ {\widehat{g}}^2_k - {\widehat{g}}_0^2 \mathbb {1}_{\{|k|\le \ell _{\delta }^{-1}\}} - {\widehat{W}}_1^2(k) + {\widehat{W}}_1^2(0)\mathbb {1}_{\{|k|\le \ell _{\delta }^{-1}\}}}{2k^2} \textrm{d}k \bigg \vert \\&\le - C\iint |g(x) g(y) -W_1(x) W_1(y)|\log \Big ( \frac{x-y}{\ell _{\delta }} \Big ) \textrm{d}x\textrm{d}y \\&\le - \frac{C}{\ell ^2} \iint |x|^2 g(x) g(y) \log \Big ( \frac{x-y}{\ell _{\delta }}\Big ) \textrm{d}x\textrm{d}y\\&= \frac{C}{\ell ^2} \int |x|^2 g(x) \omega (x) \textrm{d}x \\&\le C\frac{R^2}{\ell ^2} \delta , \end{aligned}$$

where we used first (6.24), then the fact that in 2 dimension the $\log$ term produces a convolution with the Green’s function of the Laplacian and finally formulas (3.5) and (3.6) (together with the bounds $\omega \le 1$ in the support of *g* and $2R < \ell _{\delta }$). The last inequality has a similar proof and is omitted. $\square$

We now give a result of localization to large boxes for the potential part in the Hamiltonian (6.2).

#### Lemma 6.5

(Localization of the potential). The following identity holds

6.29 $$\begin{aligned}{} & {} -\rho _{\mu }\sum _{j=1}^N \int _{{\mathbb {R}}^2} g(x_j-y) \textrm{d}y + \sum _{i<j} v(x_i-x_j) \nonumber \\{} & {} \quad = \int _{{\mathbb {R}}^2} \bigg [ -\rho _{\mu } \sum _{j=1}^N \int _{{\mathbb {R}}^2} w_{1,u}(x_j,y) \textrm{d}y\, + \,\sum _{i<j} w_u(x_i,x_j) \bigg ] \textrm{d}u. \end{aligned}$$

#### Proof

It is proven by direct calculation following the same lines as [28, Proposition 3.1]. $\square$

Therefore, joining the results from Lemmas 6.2, 6.5 and introducing the large box Hamiltonian acting on ${\mathscr {F}}_s(L^2(\Lambda _u))$ as

6.30 $$\begin{aligned} {\mathcal {H}}_{\Lambda _u}(\rho _{\mu })_N := \sum _{j=1}^N {\mathcal {T}}^{(j)}_u -\rho _{\mu } \sum _{j=1}^N \int _{{\mathbb {R}}^2} w_{1,u}(x_j,y) \textrm{d}y \, + \,\sum _{i<j} w_u(x_i,x_j), \end{aligned}$$

where ${\mathcal {T}}^{(j)}_u$ is (6.14) for the $x_j$ variable, and the ground state energy and its density

6.31 $$\begin{aligned} E_{\Lambda } (\rho _{\mu }) := \inf \textrm{Spec} ({\mathcal {H}}_{\Lambda }(\rho _{\mu })),\qquad e_{\Lambda } (\rho _{\mu }) := \frac{1}{\ell ^2}E_{\Lambda } (\rho _{\mu }) , \end{aligned}$$

we are able to prove the following. Recall that $e_0$ is defined in (6.3).

#### Lemma 6.6

Under the assumptions of Lemma 6.2,

6.32 $$\begin{aligned} e_0(\rho _{\mu }) \ge e_{\Lambda }(\rho _{\mu }). \end{aligned}$$

#### Proof

By direct application of Lemma 6.2 and Lemma 6.5 we have

6.33 $$\begin{aligned} {\mathcal {H}}_{\rho _{\mu },N}(\rho _{\mu }) \ge \int _{\ell ^{-1}(\Omega + B(0,\ell /2))}{\mathcal {H}}_{\Lambda _u}(\rho _{\mu })_N \textrm{d}u \ge \ell ^{-2}|\Omega + B(0,\ell /2)| E_{\Lambda } (\rho _{\mu }), \end{aligned}$$

where the last inequality is guaranteed by the unitary equivalence ${\mathcal {H}}_{\Lambda _u} \cong {\mathcal {H}}_{\Lambda _{u'}}$ via the relation

6.34 $$\begin{aligned} w_{u'}(x,y) = w_u (x- \ell (u'-u), y - \ell (u'-u)). \end{aligned}$$

The proof is concluded taking the infimum of the spectrum of the left-hand side and dividing by $|\Omega |$ observing that $\frac{\vert \Omega + B(0,\ell /2)\vert }{|\Omega |} \xrightarrow [|\Omega | \rightarrow + \infty ]{} 1$. $\square$

Therefore, Lemma 6.6 shows that in order to prove our main result Theorem 6.1, it is enough to give an analogous estimate on the Hamiltonian on the large box, and it is the content of the next theorem.

#### Theorem 6.7

There exist $C, \eta >0$ such that the following holds. Let $\rho _\mu >0$ and $v\in L^1(\Omega )$ be a positive, spherically symmetric potential with scattering length *a* and ${{\,\mathrm{\textrm{supp}}\,}}(v) \subset B(0,R)$ such that $\Vert v \Vert _1 \le Y^{-1/8}$ and $R \le \rho _\mu ^{-1/2}$. Then, if $\rho _\mu a^2 \le C^{-1}$, and the parameters are chosen as in “Appendix H”, we have

6.35 $$\begin{aligned} E_{\Lambda }(\rho _{\mu }) \ge - 4 \pi \ell ^2 \rho _{\mu }^2 \delta \Big (1 - \Big ( 2 \Gamma + \frac{1}{2} + \log \pi \Big ) \delta \Big ) - C \ell ^2 \rho _{\mu }^2 \delta ^{2 + \eta }. \end{aligned}$$

The proof of Theorem 6.7 is given in the remaining sections of the article.

## Lower Bounds in Position Space

### Splitting of the potential

By the definitions (6.11) of the projectors *P* and *Q*, we see that we can split the potential in a way presented in the lemma below.

#### Lemma 7.1

We have, recalling the definitions in (6.19), that

7.1 $$\begin{aligned} -\rho _{\mu } \sum _{j=1}^N \int _{{\mathbb {R}}^2} w_{1}(x_j,y) \textrm{d}y + \,\frac{1}{2}\sum _{i\ne j} w(x_i,x_j)&= \sum _{j=0}^4 {{\mathcal {Q}}}_j^{\text {r}en} \end{aligned}$$

with

7.2 $$\begin{aligned} 0 \le {{\mathcal {Q}}}_4^{\text {r}en}&:= \frac{1}{2} \sum _{i\ne j} \Big [ Q_i Q_j + \left( P_i P_j + P_i Q_j + Q_i P _j\right) \omega (x_i-x_j) \Big ] w(x_i,x_j) \nonumber \\&\,\quad \times \Big [ Q_j Q_i + \omega (x_i-x_j) \left( P_j P_i + P_j Q_i + Q_j P_i\right) \Big ], \end{aligned}$$

7.3 $$\begin{aligned} {{\mathcal {Q}}}_3^{\text {r}en}&:= \sum _{i\ne j} P_i Q_j w_1(x_i,x_j) Q_i Q_j + h.c. , \end{aligned}$$

as well as

7.4 $$\begin{aligned} {{\mathcal {Q}}}_2^{\text {r}en}&:= \sum _{i\ne j} P_i Q_j w_2(x_i,x_j) Q_i P_j + \sum _{i\ne j} P_i Q_j w_2(x_i,x_j) P_i Q_j \end{aligned}$$

7.5 $$\begin{aligned}&\quad +\frac{1}{2}\sum _{i\ne j} P_iP_j w_1(x_i,x_j) Q_i Q_j + h.c.-\rho _{\mu } \sum _{j=1}^N Q_i \int _{{\mathbb {R}}^2} w_1(x_i,y) \textrm{d}y \, Q_i, \end{aligned}$$

7.6 $$\begin{aligned} {{\mathcal {Q}}}_1^{\text {r}en}&:=\sum _{i,j} Q_i P_j w_2(x_i,x_j) P_i P_j - \rho _{\mu }\sum _{i=1}^N Q_i \int _{{\mathbb {R}}^2} w_1(x_i,y) \textrm{d}y \, P_i + h.c., \end{aligned}$$

and

7.7 $$\begin{aligned} {{\mathcal {Q}}}_0^{\text {r}en}&:= \frac{1}{2} \sum _{i \ne j} P_i P_j w_2(x_i,x_j) P_i P_j - \rho _{\mu } \sum _{j=1}^N P_j \int _{{\mathbb {R}}^2} w_1(x_j,y) \textrm{d}y \, P_j. \end{aligned}$$

#### Proof

It follows from an elementary calculation, using that $P+Q=\mathbb {1}$ on $L^2(\Lambda )$ and, where needed, the identity

7.8 $$\begin{aligned} w_1 = w_2 -w \omega + w \omega ^2. \end{aligned}$$

$$\square $$

We rewrite now some of the previous *Q* terms in the lemma below.

#### Lemma 7.2

With the notation $\rho _0 = \frac{n_0}{\ell ^2}$ we have

7.9 $$\begin{aligned} {\mathcal {Q}}_0^{\text {r}en}&= \frac{\rho _0(n_0 -1)}{2} ({\widehat{g}}(0) + \widehat{g\omega }(0)) -\rho _{\mu } n_0 {\widehat{g}}(0), \end{aligned}$$

7.10 $$\begin{aligned} {\mathcal {Q}}_1^{\text {r}en}&= ( \rho _0 - \rho _{\mu }) \sum _{i=1}^N Q_i \chi _{\Lambda }(x_i) W_1 *\chi _{\Lambda }(x_i) P_i + h.c. \nonumber \\&\quad +\rho _0 \sum _{i=1}^N Q_i \chi _{\Lambda }(x_i) ((W_1 \omega ) * \chi _{\Lambda })(x_i) P_i + h.c., \end{aligned}$$

and

7.11 $$\begin{aligned} {\mathcal {Q}}_2^{\text {r}en}&\ge \sum _{i \ne j} P_i Q_j w_2(x_i,x_j) Q_i P_j + \frac{1}{2} \sum _{i \ne j} (P_i P_j w_1(x_i,x_j) Q_i Q_j + h.c.) \nonumber \\&\quad + ((\rho _0 - \rho _{\mu }){\widehat{W}}_1(0) + \rho _0 \widehat{W_1 \omega }(0)) \sum _{j=1}^N Q_j \chi _{\Lambda }(x_j)^2 Q_j - C (\rho _{\mu } + \rho _0) \delta \Big (\frac{R}{\ell }\Big )^2 n _+. \end{aligned}$$

#### Proof

The first two identities are straightforward after having observed that

7.12 $$\begin{aligned} \sum _{j=1}^N P_j w_1(x_i,x_j) P_j = \frac{1}{\ell ^2}\sum _{j=1}^N P_j \int _{\Lambda } w_1(x_i,y) \textrm{d}y= \rho _0 \int _{\Lambda } w_1(x_i,y) \textrm{d}y, \end{aligned}$$

and

7.13 $$\begin{aligned} \int _{\Lambda } w_1(x_i,y)dy = \chi _{\Lambda }(x_i) (W_1 * \chi _{\Lambda })(x_i). \end{aligned}$$

For the ${\mathcal {Q}}_2^{\text {r}en}$ term, the only parts which require a different approach are

7.14 $$\begin{aligned} (\rho _0 - \rho _{\mu }) \sum _{j=1}^N Q_j \chi _{\Lambda }(x_j) W_1 * \chi _{\Lambda }(x_j) Q_j + \rho _0 \sum _{j=1}^N Q_j \chi _{\Lambda }(x_j) ((W_1\omega )* \chi _{\Lambda })(x_j) Q_j.\nonumber \\ \end{aligned}$$

Using Eq. (6.25) of Lemma 6.4 we can bound

7.15 $$\begin{aligned} \sum _{j=1}^N Q_j \chi _{\Lambda }(x_i) W_1 * \chi _{\Lambda }(x_i) Q_j&\ge \Vert W_1\Vert _{L^1} \sum _{j=1}^N Q_j \chi _{\Lambda }(x_j)^2 Q_j \nonumber \\&\quad - C\max _{i,j}\Vert \partial _i \partial _j \chi \Vert _{\infty } \frac{R^2}{\ell ^2 }\Vert W_1\Vert _{L^1} \Vert \chi \Vert _{\infty } n_+. \end{aligned}$$

Recalling that $\Vert W_1\Vert _{L^1} \le C \delta$ (Lemma 6.4) and acting similarly for the other term, this concludes the proof. $\square$

As a direct consequence of the lemma above, we can derive the following first lower bound for the large box Hamiltonian.

#### Corollary 7.3

The following bound holds for the Hamiltonian in the large box

7.16 $$\begin{aligned} {\mathcal {H}}_{\Lambda }(\rho _{\mu })\big |_N&\ge \sum _{j=1}^N {\mathcal {T}}^{(j)} + \frac{\rho _0(n_0 -1)}{2} ({\widehat{g}}(0) + \widehat{g\omega }(0)) -\rho _{\mu } n_0 {\widehat{g}}_0 \end{aligned}$$

7.17 $$\begin{aligned}&\quad + \Big (\rho _0- \rho _{\mu }\Big ) \sum _{i=1}^N Q_i \chi _{\Lambda }(x_i) W_1 *\chi _{\Lambda }(x_i) P_i + h.c. \end{aligned}$$

7.18 $$\begin{aligned}&\quad +\rho _0 \sum _{i=1}^N Q_i \chi _{\Lambda }(x_i) ((W_1 \omega ) * \chi _{\Lambda })(x_i) P_i + h.c. \end{aligned}$$

7.19 $$\begin{aligned}&\quad +\sum _{i \ne j} P_i Q_j w_2(x_i,x_j) Q_i P_j + \frac{1}{2} \sum _{i \ne j} (P_i P_j w_1(x_i,x_j) Q_i Q_j + h.c.) \end{aligned}$$

7.20 $$\begin{aligned}&\quad + ((\rho _0 - \rho _{\mu }){\widehat{W}}_1(0) + \rho _0 \widehat{W_1 \omega }(0)) \sum _{j=1}^N Q_j \chi _{\Lambda }(x_j)^2 Q_j \end{aligned}$$

7.21 $$\begin{aligned}&\quad - C (\rho _{\mu } + \rho _0) \delta \Big (\frac{R}{\ell }\Big )^2 n _+ + {\mathcal {Q}}_3^{\text {ren}} + {\mathcal {Q}}_4^{\text {ren}}. \end{aligned}$$

In the lemma below we prove an estimate which is going to be useful in Sect. 7.2 to localize the ${\mathcal {Q}}_3^{\text {ren}}$ term.

#### Lemma 7.4

Let $Q'$ be a possibly non self-adjoint operator on $L^2(\Lambda )$ such that $Q Q'= Q'$ and $\Vert Q'\Vert \le 1$. Then for all $c \in (0,1)$ there is a $C >0$ such that, if $R \le \ell$,

$$\begin{aligned}{} & {} \sum _{i \ne j} (P_i Q'_{j} w_1(x_i,x_j)Q_i Q_j + h.c.) \\{} & {} \quad \ge - \frac{1}{4} {\mathcal {Q}}_4^{\text {ren}} - \sum _{i \ne j} (P_i Q'_{j} w_1 \omega P_i P_j + h.c.) - \delta n_0\Big ( c K_{\ell }^{-2} \frac{n_+}{\ell ^2} + C \frac{ K_{\ell }^{2}}{\ell ^2}\sum _{j=1}^N Q_j' (Q_j')^{\dagger } \Big ). \end{aligned}$$

#### Proof

The idea is to reobtain the $Q_4$ term in the inequalities.

7.22 $$\begin{aligned} \sum _{i \ne j} (P_i Q'_{j} w_1 Q_i Q_j + h.c.)&= \sum _{i \ne j} P_i Q'_{j} w_1 \left[ Q_i Q_j + \omega (P_iP_j +P_iQ_j + Q_i P_j) \right] + h.c. \nonumber \\&\quad - \sum _{i \ne j} P_i Q'_{j} w_1\omega (P_iP_j +P_iQ_j + Q_i P_j) + h.c. \end{aligned}$$

We use Cauchy–Schwarz inequality on both the terms on the right-hand side. The first line of (7.22), using that $w_1 \le w$, is controlled by

$$\begin{aligned} C \sum _{i \ne j} P_i Q'_{j} w_1 (P_i Q'_{j})^{\dagger } + \frac{1}{4} {\mathcal {Q}}_4^{\text {ren}}&= C\frac{n_0}{\ell ^2} \sum _{j=1}^N Q'_{j} \chi _{\Lambda }(x_j)(W_1 * \chi _{\Lambda })(x_j) (Q'_{j})^{\dagger } + \frac{1}{4} {\mathcal {Q}}_4^{\text {ren}} \\&\le C \frac{n_0}{\ell ^2} \Vert \chi _{\Lambda }\Vert _{\infty }^2 \delta \sum _{j=1}^N Q_j'(Q_j')^{\dagger } + \frac{1}{4} {\mathcal {Q}}_4^{\text {ren}}, \end{aligned}$$

where we used (7.12), (7.13), the bound $\Vert W_1\Vert _{L^1} \le C \delta ( 1 + R^2 \ell ^{-2} )$ and $R \le \ell$. For the second line of (7.22) we keep the *PP* contribution and treat the other terms separately. They can be estimated as above. For instance,

7.23 $$\begin{aligned} \sum _{i\ne j} ( P_i Q'_j w_1 \omega P_i Q_j + h.c.)&\le \varepsilon ^{-1}\sum _{i\ne j} P_i Q'_j w_1 \omega (P_i Q'_j)^\dagger + \varepsilon \sum _{i \ne j } P_i Q_j w_1 \omega P_i Q_j \nonumber \\&\le C\delta \frac{n_0}{\ell ^2} \Big ( \varepsilon ^{-1}\sum _{j=1}^N Q_j' (Q_j')^{\dagger } + \varepsilon n_+\Big ), \end{aligned}$$

where we used the Cauchy–Schwarz inequality with weight $\varepsilon >0$. Choosing $\varepsilon = cC^{-1} K_{\ell }^{-2}$ with $c \in (0,1)$, we get

7.24 $$\begin{aligned} \sum _{i\ne j} P_i Q'_j w_1 \omega P_i Q_j \le c^{-1} C^2\delta \frac{n_0 d^2 K_{\ell }^{2}}{(d\ell )^2} \sum _{j=1}^N Q_j' (Q_j')^{\dagger } + c \delta n_0 K_{\ell }^{-2}\frac{n_+}{\ell ^2}, \end{aligned}$$

and the lemma follows. $\square$

### Localization of 3*Q* term

In this section we show how we can restrict the action of one of the *Q* projectors in the ${\mathcal {Q}}^{\text {ren}}_3$ term to low momenta. More precisely we define the following two sets of low and high momenta respectively,

7.25 $$\begin{aligned} {\mathcal {P}}_L :=\{p \in {\mathbb {R}}^2\,|\; |p| \le d^{-2}\ell ^{-1}\}, \qquad {\mathcal {P}}_H :=\{p \in {\mathbb {R}}^2\,|\; |p| \ge K_H \ell ^{-1}\}. \end{aligned}$$

We choose the parameters *d* and $K_H$ satisfying (H6) so that the two sets are disjoint. We will localize the *Q* projector using the following cutoff function,

7.26 $$\begin{aligned} f_L(r) := f (d^2 \ell r ), \qquad f(r) := {\left\{ \begin{array}{ll} 1, &{}\text {if} \quad r \le 1,\\ 0, &{}\text {if} \quad r \ge 2, \end{array}\right. } \end{aligned}$$

where $f \in C^{\infty }({\mathbb {R}})$ is a non-increasing function. The localized projectors are

7.27 $$\begin{aligned} Q_L := Q f_L(\sqrt{-\Delta }), \qquad {\overline{Q}}_L := Q - Q_L, \end{aligned}$$

and the localized version of ${\mathcal {Q}}_3^{\text {ren}}$ is

7.28 $$\begin{aligned} {\mathcal {Q}}^{\text {low}}_3 := \sum _{i \ne j} (P_i Q_{L,j} w_1(x_i,x_j)Q_i Q_j + h.c.). \end{aligned}$$

The number of high excitations, namely the number of bosons outside from the condensate and with momenta not in ${\mathcal {P}}_L$ is

7.29 $$\begin{aligned} n_+^H := \sum _{j=1}^N Q_j \, \mathbb {1}_{(d^{-2}\ell ^{-1} , \infty )}(\sqrt{-\Delta _j}) Q_j. \end{aligned}$$

It is easy to see that

7.30 $$\begin{aligned} \sum _{j=1}^N {\overline{Q}}_{L,j}{\overline{Q}}_{L,j}^{\dagger } \le n_+^H. \end{aligned}$$

The next lemma shows how the ${\mathcal {Q}}_3^{\text {ren}}$ term added to a small contribution from ${\mathcal {Q}}_4^{\text {ren}}$ and to the spectral gap from the kinetic energy (see (6.14)), can be bounded above by ${\mathcal {Q}}_3^{\text {low}}$.

#### Lemma 7.5

Assume $R \le \ell$ and the relation (H26) between the parameters. Then there exists $C>0$ such that, for any *n*-particles state $\Psi \in {\mathscr {F}}_s(L^2(\Lambda ))$ with $n \le 2 \rho _\mu \ell ^2$,

$$\begin{aligned} \langle {\mathcal {Q}}_3^{\text {ren}}\rangle _\Psi + \frac{1}{4} \langle {\mathcal {Q}}_4^{\text {ren}} \rangle _\Psi + \frac{b}{100}\Big ( \frac{\langle n_+ \rangle _\Psi }{\ell ^2} + \varepsilon _T\frac{\langle n_+^H \rangle _\Psi }{(d\ell )^2}\Big ) \ge \langle {\mathcal {Q}}_3^{\text {low}}\rangle _\Psi - C \delta \frac{n^2}{\ell ^2} (d^{2M-2} + R^2 \ell ^{-2}) \end{aligned}$$

where the fixed number *b* was introduced in Lemma 6.2.

#### Proof

By definition

7.31 $$\begin{aligned} {\mathcal {Q}}_3^{\text {ren}} - {\mathcal {Q}}_3^{\text {low}} = \sum _{i \ne j} (P_i {\overline{Q}}_{L,j} w_1(x_i,x_j)Q_i Q_j + h.c.). \end{aligned}$$

We use now Lemma 7.4 with $Q' = {\overline{Q}}_L$ and the estimate (7.30) to get

7.32 $$\begin{aligned} {\mathcal {Q}}_3^{\text {ren}} - {\mathcal {Q}}_3^{\text {low}}&\ge -\frac{1}{4} {\mathcal {Q}}_4^{\text {ren}} - \sum _{i \ne j} (P_i {\overline{Q}}_{L,j} w_1 \omega P_i P_j + h.c.) \nonumber \\&\quad - \delta n_0\Big ( c K_{\ell }^{-2} \frac{n_+}{\ell ^2} + C \frac{d^2 K_{\ell }^{2}}{(d\ell )^2}n_+^H \Big ). \end{aligned}$$

By (7.12) we have

7.33 $$\begin{aligned}{} & {} \sum _{i \ne j} (P_i {\overline{Q}}_{L,j} w_1 \omega P_i P_j + h.c.) \nonumber \\{} & {} \quad = \frac{n_0}{\ell ^2} \bigg (\sum _{j=1}^N {\overline{Q}}_{L,j} \chi _{\Lambda }(x_j) \big ( \Vert W_1 \omega \Vert _{L^1} \chi _\Lambda (x_j) + \varepsilon (x_j) \big ) P_j + h.c. \bigg ), \end{aligned}$$

with $\varepsilon (x_j) = W_1 \omega * \chi _\Lambda (x_j) - \Vert W_1 \omega \Vert _{L^1} \chi _\Lambda (x_j)$. The $\varepsilon (x_j)$-term can be bounded using a Cauchy–Schwarz inequality and (6.25),

7.34 $$\begin{aligned} \frac{n_0}{\ell ^2} \bigg (\sum _{j=1}^N {\overline{Q}}_{L,j} \chi _{\Lambda }(x_j) \varepsilon (x_j) P_j + h.c. \bigg )&\le C \frac{n_0}{\ell ^2} \sum _{j=1}^N ( {\overline{Q}}_{L,j} \chi _\Lambda \varepsilon {\overline{Q}}_{L,j}^\dagger + P_j \chi _\Lambda \varepsilon P_j ) \nonumber \\&\le C \frac{n_0 R^2}{\ell ^4} \delta (n_+^H + n_0). \end{aligned}$$

For the other term we take $M-1 \le 2\widetilde{M} \le M$ and using the notation $D_M := (\ell ^{-2}-\Delta _j)^{\widetilde{M}}$, we write

7.35 $$\begin{aligned} {\overline{Q}}_{L,j} \chi _{\Lambda }(x_j)^2 P_j + h.c. = {\overline{Q}}_{L,j} D_M^{-1} [D_M\chi _{\Lambda }(x_j)^2] P_j + h.c. \end{aligned}$$

Therefore, by Cauchy–Schwarz inequality with weight $\varepsilon _0 >0$,

$$\begin{aligned} {\overline{Q}}_{L,j} \chi _{\Lambda }(x_j)^2 P_j + h.c. \le \varepsilon _0 P_j + \varepsilon _0^{-1} \Vert D_M \chi _{\Lambda }^2\Vert ^2_{\infty } {\overline{Q}}_{L,j} D_M^{-2} ({\overline{Q}}_{L,j})^{\dagger }. \end{aligned}$$

Now using that $\Vert D_M \chi ^2_{\Lambda }\Vert \le C \ell ^{-2\widetilde{M}}$ and that ${\overline{Q}}_{L}$ cut momenta lower than $d^{-2}\ell ^{-1}$ we obtain

7.36 $$\begin{aligned} {\overline{Q}}_{L,j} \chi _{\Lambda }(x_j)^2 P_j + h.c.&\le \varepsilon _0 P_j + \varepsilon ^{-1}_0 C \ell ^{-4\widetilde{M}} {\overline{Q}}_{L,j}(\ell ^{-2}-\Delta _j)^{-2\widetilde{M}} ({\overline{Q}}_{L,j})^{\dagger } \nonumber \\&\le \varepsilon _0 P_j + \varepsilon _0^{-1} C d^{8 \widetilde{M}} {\overline{Q}}_{L,j} ({\overline{Q}}_{L,j})^{\dagger }. \end{aligned}$$

Therefore choosing $\varepsilon _0 = d^{4 \widetilde{M}}$, we have

7.37 $$\begin{aligned} \frac{n_0}{\ell ^2} \bigg (\sum _{j=1}^N {\overline{Q}}_{L,j} \chi _{\Lambda }(x_j)^2 \Vert W_1 \omega \Vert _{L^1} P_j + h.c. \bigg ) \le C \delta d^{2M-2} \frac{n_0 }{\ell ^2}(n_+^H + n_0). \end{aligned}$$

Inserting (7.34) and (7.37) into (7.33) we find

7.38 $$\begin{aligned} \sum _{i \ne j} (P_i {\overline{Q}}_{L,j} w_1 \omega P_i P_j + h.c.) \le C \delta \frac{n_0}{\ell ^2} ( n_+^H + n_0) (d^{2M-2} + R^2 \ell ^{-2}). \end{aligned}$$

We use this last bound in (7.32) and apply it to the state $\Psi$,

7.39 $$\begin{aligned} \langle&{\mathcal {Q}}_3^{\text {ren}}\rangle _\Psi - \langle {\mathcal {Q}}_3^{\text {low}} \rangle _\Psi \nonumber \\&\ge -\frac{1}{4} \langle {\mathcal {Q}}_4^{\text {ren}} \rangle _\Psi - C \delta \frac{n}{\ell ^2} ( \langle n_+^H \rangle _\Psi + n ) (d^{2M-2} + R^2 \ell ^{-2}) \nonumber \\&\quad - c \delta n K_\ell ^{-2} \frac{\langle n_+ \rangle _\Psi }{\ell ^2} - C \delta n d^2 K_\ell ^2 \frac{\langle n_+^H \rangle _\Psi }{(d\ell )^2} \nonumber \\&\ge -\frac{1}{4} \langle {\mathcal {Q}}_4^{\text {ren}} \rangle _\Psi - C \delta \frac{n^2}{\ell ^2} (d^{2M-2} + R^2 \ell ^{-2}) - c \frac{\langle n_+ \rangle _\Psi }{\ell ^2} - C d^2 K_\ell ^4 \frac{\langle n_+^H \rangle _\Psi }{(d\ell )^2}, \end{aligned}$$

where we used $n \le 2 \rho _\mu \ell ^2$ and $\ell ^2 = K_\ell ^2 \rho _\mu ^{-1} Y^{-1}$. We conclude by choosing $c = \frac{b}{100}$ and using the relation (H26) between the parameters. $\square$

### A priori bounds and localization of the number of excitations

The purpose of this section is to get bounds on the number of excitations of the system. First of all, Theorem 7.6 gives a priori bounds on $n_+$.

#### Theorem 7.6

There exists a universal constant $C >0$ such that, if $\Psi \in {\mathscr {F}}_s(L^2(\Lambda ))$ is a normalized *n*-bosons vector which satisfies

7.40 $$\begin{aligned} \langle {\mathcal {H}}_{\Lambda }(\rho _{\mu })\rangle _{\Psi } \le -4 \pi \rho _{\mu }^2 \ell ^2 Y \left( 1 - C K_B^2 Y \vert \log Y \vert \right) , \end{aligned}$$

with $K_{B}$ fixed in “Appendix H”, then

7.41 $$\begin{aligned} \langle n_+\rangle _{\Psi }&\le C K_B^2 K_{\ell }^2 \rho _{\mu } \ell ^2 Y |\log Y|, \end{aligned}$$

7.42 $$\begin{aligned} \langle {\mathcal {Q}}_4^{\text {ren}}\rangle _{\Psi }&\le C K_B^2 K_{\ell }^2 \rho _{\mu }^2 \ell ^2 Y^2 |\log Y|, \end{aligned}$$

7.43 $$\begin{aligned} \left| \rho _{\mu } - \frac{n}{\ell ^2}\right|&\le C K_B K_{\ell }\rho _{\mu } Y^{1/2} |\log Y|^{1/2}. \end{aligned}$$

#### Proof

It is proved in “Appendix D”, using a second localization to "small boxes" of size $\ll \ell _\delta$. $\square$

We also need to bound the number of low excitations, defined in terms of our modified kinetic energy ${\mathcal {T}}$. More precisely we define, for a certain $\widetilde{K}_H \gg 1$ fixed in “Appendix H”, the projectors

7.44 $$\begin{aligned} {\overline{Q}}_H = \mathbb {1}_{(0, \widetilde{K}_H^2 \ell ^{-2})} ({\mathcal {T}}), \qquad Q_H = Q- {\overline{Q}}_H, \end{aligned}$$

which satisfy

7.45 $$\begin{aligned} P + {\overline{Q}}_H + Q_H = 1_{\Lambda }. \end{aligned}$$

We will consider the operators

7.46 $$\begin{aligned} n_+^L := \sum _j {\overline{Q}}_{H,j}, \qquad \widetilde{n}_+^H := \sum _{j} Q_{H,j}, \end{aligned}$$

for which we prove the following result.

#### Theorem 7.7

(Restriction on $n_+^L$). There exist *C*, $\eta > 0$ such that the following holds. Let $\Psi \in {\mathscr {F}}_s(L^2(\Lambda ))$ be a normalized *n*-particle vector which satisfies (7.40). Assume that the potential *v* is such that $\Vert v \Vert _1 \le Y^{-1/8}$. Then, for ${\mathcal {M}} \gg 1$ satisfying condition (H24) there exists a sequence $\{\Psi ^{m}\}_{m \in {\mathbb {Z}}} \subseteq {\mathscr {F}}_s(L^2(\Lambda ))$ such that $\sum _m \Vert \Psi ^m \Vert ^2 = 1$ and

7.47 $$\begin{aligned} \Psi ^{m} = \mathbb {1}_{[0,\frac{{\mathcal {M}}}{2} +m]}(n_+^L) \Psi ^{m}, \end{aligned}$$

and such that the following lower bound holds true

$$\begin{aligned}{} & {} \langle \Psi , {\mathcal {H}}_{\Lambda }(\rho _{\mu }) \Psi \rangle \ge \sum _{2 |m|\le {\mathcal {M}} }\langle \Psi ^{m}, {\mathcal {H}}_{\Lambda }(\rho _{\mu }) \Psi ^m\rangle -C \rho _{\mu }^2 \ell ^2 Y^{2+\eta } \\{} & {} \quad -4 \pi \rho _{\mu }^2 \ell ^2 Y \Big ( 1 - C K_B^2 Y \vert \log Y \vert \Big )\sum _{2|m| >{\mathcal {M}}} \Vert \Psi ^m\Vert ^2. \end{aligned}$$

Notice that, if such a state $\Psi$ does not exist, then our lower bound is already proven (see when we apply Theorem 7.7 in (9.80)). From this result we see that, in order to prove Theorem 6.7, we only need to bound the energy of states satisfying the bound $n_+^L \le {\mathcal {M}}$. In the remainder of this section, we prove Theorem 7.7. The following lemma states that for a lower bound we can restrict to states with finitely many excitations $n_+$, up to small enough errors. The proof of this lemma is inspired by the localization of large matrices result in [29]. It is also really similar to the bounds in [30, Proposition 21]. It could be interpreted as an analogue of the standard IMS localization formula. The error produced is written in terms of the following quantities $d_1^L$ and $d_2^L$:

7.48 $$\begin{aligned} d_{1}^L&:= -\rho _{\mu } \sum _{i} (P_i + Q_{H,i})\int w_1(x_i,y) \textrm{d}y \; {\overline{Q}}_{H,i} + h.c. \nonumber \\&\quad +\sum _{i \ne j} (P_i + Q_{H,i}){\overline{Q}}_{H,j} w(x_i,x_j) {\overline{Q}}_{H,i} {\overline{Q}}_{H,j} + h.c. \nonumber \\&\quad + \sum _{i \ne j} {\overline{Q}}_{H,i} (P_j + Q_{H,j}) w(x_i,x_j) (P_i + Q_{H,i})(P_j + Q_{H,j}) + h.c. \end{aligned}$$

and

7.49 $$\begin{aligned} d_2^L&:= \sum _{i \ne j} (P_i + Q_{H,i})(P_j + Q_{H,j}) w(x_i,x_j) {\overline{Q}}_{H,j} {\overline{Q}}_{H,i} +h.c. \end{aligned}$$

#### Lemma 7.8

Let $\theta : {\mathbb {R}}\rightarrow [0,1]$ be any compactly supported Lipschitz function such that $\theta (s) = 1$ for $\vert s \vert < \frac{1}{8}$ and $\theta (s) = 0$ for $\vert s \vert > \frac{1}{4}$. For any ${\mathcal {M}} >0$, define $c_{{\mathcal {M}}} >0$ and $\theta _{{\mathcal {M}}}$ such that

$$\begin{aligned} \theta _{{\mathcal {M}}}(s) = c_{{\mathcal {M}}} \theta \Big ( \frac{s}{{\mathcal {M}}} \Big ) , \qquad \sum _{s \in {\mathbb {Z}}} \theta _{{\mathcal {M}}}(s)^2 = 1 .\end{aligned}$$

Then there exists a $C>0$ depending only on $\theta$ such that, for any normalized state $\Psi \in {\mathscr {F}}_s(L^2(\Lambda ))$,

7.50 $$\begin{aligned} \langle \Psi , {\mathcal {H}}_{\Lambda }(\rho _\mu ) \Psi \rangle \ge \sum _{m \in {\mathbb {Z}}} \langle \Psi ^m , {\mathcal {H}}_\Lambda (\rho _\mu ) \Psi ^m \rangle - \frac{C}{{\mathcal {M}}^2} \left( \vert \langle d_1^L \rangle _\Psi \vert + \vert \langle d_2^L \rangle _\Psi \vert \right) , \end{aligned}$$

where $$\Psi ^m = \theta _{{\mathcal {M}}}(n_+^L - m) \Psi $$.

#### Proof

Notice that ${\mathcal {H}}_{\Lambda }$ only contains terms that change $n_+^L$ by $0, \pm 1$ or $\pm 2$. Therefore, we write our operator as ${\mathcal {H}}_\Lambda (\rho _\mu ) = \sum _{\vert k \vert \le 2} {\mathcal {H}}_k$, with ${\mathcal {H}}_k n_+^L = (n_+^L + k) {\mathcal {H}}_k$. Moreover, ${\mathcal {H}}_k + {\mathcal {H}}_{-k} = d^L_k$ for $k=1,2$. We use this decomposition to estimate the localized energy,

$$\begin{aligned} \sum _{m \in {\mathbb {Z}}} \langle \Psi ^m, {\mathcal {H}}_{\Lambda }\Psi ^m \rangle&= \sum _{m \in {\mathbb {Z}}} \sum _{\vert k \vert \le 2} \langle \theta _{{\mathcal {M}}}(n_+^L -m) \theta _{{\mathcal {M}}}(n_+^L-m+k) \Psi , {\mathcal {H}}_k \Psi \rangle \\&= \sum _{m, s \in {\mathbb {Z}}} \sum _{\vert k \vert \le 2} \langle \theta _{{\mathcal {M}}}(s-m) \theta _{{\mathcal {M}}}(s-m+k) \mathbb {1}_{\{n_+^L =s\}} \Psi , {\mathcal {H}}_k \Psi \rangle \\&= \sum _{m,s \in {\mathbb {Z}}} \sum _{\vert k \vert \le 2} \theta _{{\mathcal {M}}}(m) \theta _{{\mathcal {M}}}(m+k) \langle \mathbb {1}_{\{n_+^L = s\}} \Psi , {\mathcal {H}}_k \Psi \rangle , \end{aligned}$$

where in the last line we changed the index *m* into $s-m$. We can sum on *s* to recognize

7.51 $$\begin{aligned} \sum _{m \in {\mathbb {Z}}} \langle \Psi ^m, {\mathcal {H}}_{\Lambda } \Psi ^m \rangle = \sum _{m \in {\mathbb {Z}}} \sum _{\vert k \vert \le 2} \theta _{{\mathcal {M}}}(m) \theta _{{\mathcal {M}}}(m+k) \langle \Psi , {\mathcal {H}}_k \Psi \rangle . \end{aligned}$$

Furthermore the energy of $\Psi$ can be rewritten as

7.52 $$\begin{aligned} \langle \Psi , {\mathcal {H}}_{\Lambda } \Psi \rangle = \sum _{\vert k \vert \le 2} \langle \Psi , {\mathcal {H}}_k \Psi \rangle = \sum _{m \in {\mathbb {Z}}} \sum _{\vert k \vert \le 2} \theta _{{\mathcal {M}}}(m)^2 \langle \Psi , {\mathcal {H}}_k \Psi \rangle , \end{aligned}$$

by definition of $\theta _{{\mathcal {M}}}$. Thus, the localization error is

7.53 $$\begin{aligned} \sum _{m \in {\mathbb {Z}}} \langle \Psi ^m, {\mathcal {H}}_{\Lambda } \Psi ^m \rangle - \langle \Psi , {\mathcal {H}}_{\Lambda } \Psi \rangle = \sum _{\vert k \vert \le 2} \delta _k \langle \Psi , {\mathcal {H}}_k \Psi \rangle , \end{aligned}$$

with

7.54 $$\begin{aligned} \delta _k = \sum _{m \in {\mathbb {Z}}} \big ( \theta _{{\mathcal {M}}} (m) \theta _{{\mathcal {M}}}(m+k) - \theta _{{\mathcal {M}}}(m)^2 \big ) = - \frac{1}{2} \sum _m \big ( \theta _{{\mathcal {M}}}(m) - \theta _{{\mathcal {M}}}(m+k) \big )^2.\nonumber \\ \end{aligned}$$

Since $\delta _0 = 0$, $\delta _k = \delta _{-k}$ and $d_k^L = {\mathcal {H}}_k + {\mathcal {H}}_{-k}$ we find

7.55 $$\begin{aligned} \sum _{m \in {\mathbb {Z}}} \langle \Psi ^m, {\mathcal {H}}_{\Lambda } \Psi ^m \rangle - \langle \Psi , {\mathcal {H}}_{\Lambda } \Psi \rangle = \delta _1 \langle d_1^L \rangle _\Psi + \delta _2 \langle d_2^L \rangle _\Psi , \end{aligned}$$

and only remains to prove that $\vert \delta _k \vert \le C {\mathcal {M}}^{-2}$. Using (7.54) and the definition of $\theta _{{\mathcal {M}}}$,

7.56 $$\begin{aligned} \vert \delta _k \vert = \frac{c_{{\mathcal {M}}}^2}{2} \sum _{m \in {\mathbb {Z}}} \Big [ \theta \Big ( \frac{m}{{\mathcal {M}}} \Big ) - \theta \Big ( \frac{m+k}{{\mathcal {M}}} \Big ) \Big ]^2. \end{aligned}$$

We can restrict the sum to $m \in \big [- \frac{ {\mathcal {M}}}{2} , \frac{ {\mathcal {M}}}{2} \big ]$, since the other terms vanish due to $\theta$ being a cutoff function. This sum contains ${\mathcal {M}} + 1$ terms which we can bound using the Lipschitz constant *L* of $\theta$,

7.57 $$\begin{aligned} \vert \delta _k \vert \le c_{{\mathcal {M}}}^2\frac{ {\mathcal {M}} + 1}{2} \frac{L^2 k^2}{{\mathcal {M}}^2} \le \frac{2 L^2 k^2}{{\mathcal {M}}^2}, \end{aligned}$$

where in the last inequality we used

7.58 $$\begin{aligned} c_{{\mathcal {M}}}^2 = \Big ( \sum _{s \in {\mathbb {Z}}} \theta \Big ( \frac{s}{{\mathcal {M}}} \Big )^2 \Big ) ^{-1} \le \frac{1}{ {\mathcal {M}} / 4 + 1}. \end{aligned}$$

$$\square $$

To estimate the error in (7.50), we need the following bounds on $d_1^L$ and $d_2^L$.

#### Lemma 7.9

Let $\widetilde{{\mathcal {M}}} >0$ and $\Psi \in {\mathscr {F}}_s(L^2(\Lambda ))$ be a normalized *n*-bosons vector satisfying

$$\begin{aligned}\Psi = \mathbb {1}_{[0,\widetilde{{\mathcal {M}}}]}(n_+^L)\Psi .\end{aligned}$$

Then, assuming the choices of parameters in “Appendix H” we have

7.59 $$\begin{aligned}&\vert \langle d_{1}^L \rangle _\Psi \vert + \vert \langle d_2^L\rangle _{\Psi } \vert \\&\le \rho _{\mu }^2 \ell ^2 \Vert v\Vert _1 \Big ( \frac{\langle n_+\rangle _{\Psi }^{1/2}}{n^{1/2}} + \frac{\widetilde{{\mathcal {M}}}^{1/2} \langle n_+\rangle _{\Psi }^{1/2}}{n} \varepsilon _N^{-1/4} \widetilde{K}_H + \frac{\widetilde{{\mathcal {M}}} \langle n_+\rangle _{\Psi }}{n^2} \varepsilon _N^{-1/2} \widetilde{K}_H^2 \Big ) +C \langle {\mathcal {Q}}_4^{\text {ren}}\rangle _{\Psi }.\nonumber \end{aligned}$$

#### Proof

We give the proof in “Appendix E”. $\square$

Now we can combine Lemmas 7.8, 7.9 and Theorem 7.6 to prove Theorem 7.7.

#### Proof of Theorem 7.7

Given a *n*-sector state $\Psi \in L^2(\Lambda ^n)$ satisfying (7.40), we can apply Lemma 7.8 and write $\Psi ^m = \theta _{{\mathcal {M}}}(n_+^L -m)\Psi$. In (7.50) we split the sum into two. The first part, for $|m| < \frac{1}{2} {\mathcal {M}}$, we keep. For $|m| > \frac{1}{2} {\mathcal {M}}$, $\Psi _m$ satisfies

7.60 $$\begin{aligned} \langle n_+\rangle _{\Psi ^m} \ge \langle n_+^L\rangle _{\Psi ^m} \ge \frac{ {\mathcal {M}}}{4}\Vert \Psi ^m \Vert ^2 , \end{aligned}$$

due to the cutoff $\theta _{{\mathcal {M}}}(n_+^L - m)$. Thanks to condition (H24) on ${\mathcal {M}}$, this is a larger bound than (7.41), and thus the assumption of Theorem 7.6 cannot be satisfied for $\Psi ^m$ and we must have the lower bound

7.61 $$\begin{aligned} \langle \Psi ^m, {\mathcal {H}}_{\Lambda }(\rho _{\mu }) \Psi ^m\rangle \ge -4 \pi \rho _{\mu }^2 \ell ^2 Y \Big ( 1 - C K_B^2 Y \vert \log Y \vert \Big )\Vert \Psi ^m \Vert ^2. \end{aligned}$$

We finally bound the last term in (7.50), using Lemma 7.9 with $\widetilde{{\mathcal {M}}} = n$,

$$\begin{aligned} \vert \langle d_{1}^L \rangle _\Psi \vert + \vert \langle d_2^L\rangle _{\Psi } \vert \le \rho _\mu ^2 \ell ^2 \Vert v \Vert _1^2 \Big ( \frac{1+ \varepsilon _N^{-1/4}\widetilde{K}_H}{n^{1/2}} \langle n_+ \rangle _\Psi + \frac{\varepsilon _N^{-1/2} {\widetilde{K}}_H^2}{n} \langle n_+ \rangle _\Psi \Big ) + C \langle {\mathcal {Q}}_4^{\text {ren}} \rangle _\Psi . \end{aligned}$$

Now we use the condensation estimate (7.41) and the bound (7.42) on $Q_4^{\text {ren}}$ to obtain

7.62 $$\begin{aligned}&\vert \langle d_{1}^L \rangle _\Psi \vert + \vert \langle d_2^L\rangle _{\Psi } \vert \\&\le \rho ^2_{\mu } \ell ^2 \Vert v\Vert _1 \Big ( Y^{1/2} |\log Y|^{1/2} K_{\ell } K_B \widetilde{K}_H \varepsilon _N^{-1/4}+ Y |\log Y| K_{\ell }^2 K_B^2 \widetilde{K}_H^2 \varepsilon _N^{-1/2}\Big ).\nonumber \end{aligned}$$

The relation (H13) between the parameters implies that the largest term in (7.62) is the first one. Using the conditions (H11) and (H24) on $\varepsilon _N$ and ${\mathcal {M}}$ respectively, and the assumptions on $\Vert v \Vert _1$ we find

7.63 $$\begin{aligned} \frac{ |\langle d_{1}^L \rangle _\Psi \vert + \vert \langle d_2^L\rangle _{\Psi } |}{{\mathcal {M}}^2} \le \rho ^2_{\mu } \ell ^2 Y^{2+\eta } . \end{aligned}$$

Using the estimates (7.61) for $m > \frac{1}{2} {\mathcal {M}}$ and (7.63) in formula (7.50) we conclude the proof. $\square$

## Lower Bounds in Second Quantization

### Second quantization formalism

We rewrite the Hamiltonian in the second quantization formalism. Let us introduce the operators, where $\#$ can be nothing or $\dagger$ for the annihilation or creation operators on the space ${\mathscr {F}}_s(L^2(\Lambda ))$, respectively,

8.1 $$\begin{aligned} a_0^{\#} := \frac{1}{\ell } \,a^{\#}(\theta ), \qquad \text {and} \qquad [a_0, a_0^{\dagger }] = 1, \end{aligned}$$

being the creation and annihilation operators for bosons with zero momentum, where $\theta$ is the sharp localization function on $\Lambda$ (see (6.9)). For $k \in {\mathbb {R}}^2 \setminus \{0\}$ we also define

8.2 $$\begin{aligned} \widetilde{a}_k^{\#} := \frac{1}{\ell }\, a^{\#}(Q e^{ikx}\theta ), \end{aligned}$$

the creation and annihilation operators for bosons with non-zero momentum with *Q* defined in (6.11), and their regular analogous

8.3 $$\begin{aligned} a_k^{\#} :=\frac{1}{\ell } \, a^{\#}(Q e^{ikx}\chi _{\Lambda }), \end{aligned}$$

where $\chi _{\Lambda }$ is the regular localization function defined in “Appendix F”. We have the usual commutation relations, for $k,h \in {\mathbb {R}}^2\setminus \{0\}$

8.4 $$\begin{aligned}{}[{\widetilde{a}}_k, {\widetilde{a}}_h ] = [a_k, a_h] = 0, \qquad \text {and} \qquad [\widetilde{a}_k,\widetilde{a}^{\dagger }_h] = \frac{1}{\ell ^2}\,\langle Qe^{ikx}, Q e^{ihx} \rangle . \end{aligned}$$

Using that $P = \mathbb {1}-Q$ and ${{\widehat{\chi }}}_\Lambda (k) = \ell ^2 {{\widehat{\chi }}}(k\ell )$,

8.5 $$\begin{aligned}{}[a_k, a_h^{\dagger }] = \frac{1}{\ell ^2}\, \langle Q e^{ikx}\chi _{\Lambda },\, Q e^{ihx} \chi _{\Lambda } \rangle = \widehat{\chi ^2}((k-h)\ell ) -{\widehat{\chi }}(k \ell ) \overline{{\widehat{\chi }}}(h \ell ), \end{aligned}$$

and

8.6 $$\begin{aligned}{}[a_k, a_h^{\dagger }] \le 1. \end{aligned}$$

Let us observe, first of all, that

8.7 $$\begin{aligned} n_0 = a^{\dagger }_0 a_0, \qquad n_+ = \frac{\ell ^2}{(2\pi )^2} \int \widetilde{a}^{\dagger }_k \widetilde{a}_k \textrm{d}k. \end{aligned}$$

Let us introduce, for $k \in {\mathbb {R}}^2$, the kinetic Fourier multiplier

8.8 $$\begin{aligned} \tau (k) := (1-\varepsilon _T)\Big [|k| -\frac{1}{2s\ell }\Big ]_+^2 + \varepsilon _T \Big [|k| -\frac{1}{2 d s\ell }\Big ]_+^2. \end{aligned}$$

We will need the following technical lemma to control the number operators.

#### Lemma 8.1

Assume the relation (H27) between the parameters. Let $\Psi \in {\mathscr {F}}_s(L^2(\Lambda ))$ be a normalized state satisfying

8.9 $$\begin{aligned} \mathbb {1}_{[0,{\mathcal {M}}]} (n_+^L)\Psi = \Psi , \qquad \mathbb {1}_{[0, 2 \rho _{\mu }\ell ^2]}(n_+)\Psi = \Psi , \end{aligned}$$

then the following bounds hold

8.10 $$\begin{aligned} \Big \langle \ell ^2 \int _{\{|k| \le 2 K_H \ell ^{-1}\}} (a^{\dagger }_k a_k + \widetilde{a}^{\dagger }_k \widetilde{a}_k) \textrm{d}k \Big \rangle _{\Psi }&\le C {\mathcal {M}}, \end{aligned}$$

8.11 $$\begin{aligned} \Big \langle \ell ^2 \int _{{\mathbb {R}}^2} (a^{\dagger }_k a_k + \widetilde{a}^{\dagger }_k \widetilde{a}_k) \textrm{d}k \Big \rangle _{\Psi }&\le C {\mathcal {M}} + C \langle n_+^H\rangle _{\Psi }. \end{aligned}$$

#### Proof

The proof is analogous for both the addends, therefore we give the proof only for the $a^{\#}_k$. We want to compare localization in terms of kinetic energy with localization in momenta. We use [19, Lemma 5.2] adapted to dimension 2:

8.12 $$\begin{aligned} Q \chi _{\Lambda } \mathbb {1}_{\{|p| \le K_H \ell ^{-1}\}} \chi _{\Lambda } Q&\le C {\overline{Q}}_H + C \Big ( \Big ( \frac{K_H}{\widetilde{K}_H}\Big )^{M} + \varepsilon _N^{3/2}\Big ), \end{aligned}$$

8.13 $$\begin{aligned} Q \mathbb {1}_{\{|p| \le K_H \ell ^{-1}\}} Q&\le C {\overline{Q}}_H + C \Big ( \Big ( \frac{K_H}{\widetilde{K}_H}\Big )^{M} + \varepsilon _N^{3/2}\Big ), \end{aligned}$$

where we recall the definition (7.44) of ${\overline{Q}}_H$. Using (8.12) we have the following inequality in the *N*-th Fock sector

$$\begin{aligned} \frac{\ell ^2}{(2\pi )^2} \int _{\{|k| \le 2 K_H \ell ^{-1}\}} a^{\dagger }_k a_k \textrm{d}k \, \bigg |_N&= \sum _{j=1}^N Q_j\chi _{\Lambda }(x_j) \mathbb {1}_{(0,2K_H \ell ^{-1}]} (\sqrt{-\Delta _j})\chi _{\Lambda }(x_j) Q_j \\&\le C n_+^L + \Big ( \Big (\frac{K_H}{\widetilde{K}_H}\Big )^{M} + \varepsilon _N^{3/2}\Big )n_+. \end{aligned}$$

Using the bounds from (8.9) and the relation (H27) we deduce

8.14 $$\begin{aligned} \Big \langle \ell ^2 \int _{\{|k| \le 2 K_H \ell ^{-1}\}} a^{\dagger }_k a_k \textrm{d}k \Big \rangle _{\Psi } \le C {\mathcal {M}} + C \Big ( \Big (\frac{K_H}{\widetilde{K}_H}\Big )^{M} + \varepsilon _N^{3/2}\Big )\rho _{\mu }\ell ^2 \le C {\mathcal {M}}, \end{aligned}$$

thus proving (8.10). In order to obtain (8.11) it is enough to estimate the integral on the complementary subset. We have, again on the *N*-th sector,

8.15 $$\begin{aligned} \ell ^2 \int _{\{|k| \ge 2 K_H \ell ^{-1}\}} a^{\dagger }_k a_k \textrm{d}k\, \bigg |_N = \sum _{j=1}^N Q_j\chi _{\Lambda }(x_j) \mathbb {1}_{\{|k| \ge 2K_H \ell ^{-1}\}} (\sqrt{-\Delta _j})\chi _{\Lambda }(x_j) Q_j.\nonumber \\ \end{aligned}$$

We insert $1 = \mathbb {1}_{{\mathcal {P}}_L} + \mathbb {1}_{{\mathcal {P}}_L^c}$ and use the Cauchy–Schwarz inequality to estimate the right-hand side,

$$\begin{aligned} Q \chi _{\Lambda }&\mathbb {1}_{[2K_H \ell ^{-1}, +\infty )}(\sqrt{-\Delta })\chi _{\Lambda }Q \\&\le 2 Q \mathbb {1}_{{\mathcal {P}}_L^c}(\sqrt{-\Delta }) \chi _{\Lambda } \mathbb {1}_{[2K_H \ell ^{-1}, +\infty )} (\sqrt{-\Delta }) \chi _{\Lambda } \mathbb {1}_{{\mathcal {P}}_L^c} (\sqrt{-\Delta }) Q. \\&\quad + 2 Q \mathbb {1}_{{\mathcal {P}}_L}(\sqrt{-\Delta }) \chi _{\Lambda } \mathbb {1}_{[2K_H \ell ^{-1}, +\infty )} (\sqrt{-\Delta }) \chi _{\Lambda } \mathbb {1}_{{\mathcal {P}}_L} (\sqrt{-\Delta }) Q. \end{aligned}$$

On ${\mathcal {P}}_L^c$ we can use the bound

$$\begin{aligned} Q \mathbb {1}_{{\mathcal {P}}_L^c}(\sqrt{-\Delta }) \chi _{\Lambda } \mathbb {1}_{[2K_H \ell ^{-1}, +\infty )} (\sqrt{-\Delta }) \chi _{\Lambda } \mathbb {1}_{{\mathcal {P}}_L^c} (\sqrt{-\Delta }) Q \le \Vert \chi _{\Lambda }\Vert ^2_{\infty } Q \mathbb {1}_{{\mathcal {P}}_L^c}(\sqrt{-\Delta })Q. \end{aligned}$$

On ${\mathcal {P}}_L$ we bound the operator norm, multiplying and dividing by an *M* power of the Laplacian and using that $\chi$ has *M* bounded derivatives,

$$\begin{aligned}&\Vert \mathbb {1}_{{\mathcal {P}}_L}(\sqrt{-\Delta }) \chi _{\Lambda } \mathbb {1}_{[2K_H \ell ^{-1}, +\infty )}\Vert \\&\quad \le \Vert \mathbb {1}_{{\mathcal {P}}_L}(\sqrt{-\Delta }) \chi _{\Lambda } (-\Delta )^{M/2}\Vert \Vert (-\Delta )^{-M/2} \mathbb {1}_{[2K_H \ell ^{-1}, +\infty )} \Vert \le C (d^2 K_H)^{-M}. \end{aligned}$$

We deduce

8.16 $$\begin{aligned} \ell ^2 \int _{\{|k| \ge 2 K_H \ell ^{-1}\}} a^{\dagger }_k a_k \textrm{d}k \le C n_+^H + C(d^2 K_H)^{-2M} n_+, \end{aligned}$$

and we conclude using (H27) and the assumptions on $\Psi$. $\square$

### Second quantized Hamiltonian

We can rewrite the ${\mathcal {Q}}_3^{\text {low}}$ term (7.28) in second quantized formalism

8.17 $$\begin{aligned} {\mathcal {Q}}_3^{\text {low}} = \frac{\ell ^2}{(2\pi )^4} \int _{{\mathbb {R}}^2 \times {\mathbb {R}}^2} f_L(p) {\widehat{W}}_1(k) a^{\dagger }_0 \widetilde{a}_p^{\dagger } a_{p-k} a_k \textrm{d}k \textrm{d}p + h.c. \end{aligned}$$

An important consideration is that we can restrict the contributions in ${\mathcal {Q}}_3^{\text {low}}$ to the high momenta. This is the content of the next lemma.

#### Lemma 8.2

(Localization of ${\mathcal {Q}}_3^{\text {low}}$ to high momenta). Assume $R \le \ell$ and the relations (H16), (H17), (H27) between the parameters. If $\Psi \in {\mathscr {F}}_s(L^2(\Lambda ))$ is a *n*-particle state satisfying (7.40) and $\mathbb {1}_{[0,{\mathcal {M}}]} (n_+^L)\Psi = \Psi$ then we have

8.18 $$\begin{aligned} \langle \Psi \,|\, {\mathcal {Q}}_3^{\text {low}} \Psi \rangle \ge \langle \Psi \,|\, {\mathcal {Q}}_3^{\text {high}}\Psi \rangle - \frac{b}{100 \ell ^2} \langle n_{+} \rangle _\Psi , \end{aligned}$$

where

8.19 $$\begin{aligned} {\mathcal {Q}}^{\text {high}}_3 = \frac{\ell ^2}{(2\pi )^4} \int _{{\mathcal {P}}_H \times {\mathbb {R}}^2} f_L(p) {\widehat{W}}_1(k) a^{\dagger }_0 \widetilde{a}_p^{\dagger } a_{p-k} a_k \textrm{d}k \textrm{d}p + h.c., \end{aligned}$$

with ${\mathcal {P}}_H$ defined in (7.25).

#### Proof

First note that

8.20 $$\begin{aligned} \langle \Psi \,|\, ({\mathcal {Q}}_3^{\text {low}} - {\mathcal {Q}}_3^{\text {high}} )\Psi \rangle = \frac{\ell ^2}{(2\pi )^4} \int _{{\mathcal {P}}^c_H \times {\mathbb {R}}^2} f_L(p) {\widehat{W}}_1(k) \langle \Psi \,|\, a^{\dagger }_0 \widetilde{a}_p^{\dagger } a_{p-k} a_k \Psi \rangle \textrm{d}k \textrm{d}p + h.c.\nonumber \\ \end{aligned}$$

For any $\varepsilon >0$, using Cauchy–Schwarz on the creation and annihilation operators,

8.21 $$\begin{aligned}{} & {} \langle \Psi \,|\, ({\mathcal {Q}}_3^{\text {low}} - {\mathcal {Q}}_3^{\text {high}} ) \Psi \rangle \nonumber \\{} & {} \quad \ge - C \delta \ell ^2 \int _{{\mathcal {P}}^c_H \times {\mathbb {R}}^2} f_L(p) \Big ( \varepsilon \langle \Psi \,|\, \widetilde{a}^{\dagger }_p a^{\dagger }_0 a_0 \widetilde{a}_p \Psi \rangle + \varepsilon ^{-1} \langle \Psi \,|\, a^{\dagger }_k a^{\dagger }_{p-k} a_{p-k} a_k \Psi \rangle \Big ) \textrm{d}k \textrm{d}p , \nonumber \\ \end{aligned}$$

where we used the fact that $\Vert {\widehat{W}}_1\Vert _{\infty } \le \Vert W_1\Vert _1 \le C \delta$ (from Lemma 6.4). We now use the following inequalities, obtained by Lemma 8.1 and bounding $f_L$ by 1,

8.22 $$\begin{aligned}{} & {} \ell ^2\int _{{\mathcal {P}}_H^c \times {\mathbb {R}}^2} \, f_L(p) \langle \Psi \,|\, \widetilde{a}^{\dagger }_p a^{\dagger }_0 a_0 \widetilde{a}_p \Psi \rangle \textrm{d}k \textrm{d}p \le n \langle n_+ \rangle _{\Psi } \int _{{\mathcal {P}}_H^c} \textrm{d}k = \frac{n \langle n_+\rangle _{\Psi }}{\ell ^2} K_H^2, \end{aligned}$$

8.23 $$\begin{aligned}{} & {} \ell ^4\int _{{\mathcal {P}}_H^c \times {\mathbb {R}}^2} \, f_L(p)\langle \Psi \,|\, a^{\dagger }_k a^{\dagger }_{p-k} a_{p-k} a_k \Psi \rangle \textrm{d}k \textrm{d}p \le C{\mathcal {M}} \langle n_+\rangle _{\Psi }. \end{aligned}$$

Therefore, applying to (8.21) we obtain

8.24 $$\begin{aligned} \langle \Psi \,|\, ({\mathcal {Q}}_3^{\text {low}} - {\mathcal {Q}}_3^{\text {high}} )\Psi \rangle \ge -C\delta \frac{\langle n_+\rangle _{\Psi }}{\ell ^2} n \Big (\varepsilon K_H^2 + \varepsilon ^{-1} \frac{{\mathcal {M}}}{n}\Big ). \end{aligned}$$

Choosing $\varepsilon = K_H^{-1} \frac{{\mathcal {M}}^{1/2}}{n^{1/2}}$, we obtain

8.25 $$\begin{aligned} \langle \Psi \,|\, ({\mathcal {Q}}_3^{\text {low}} - {\mathcal {Q}}_3^{\text {high}} ) \Psi \rangle \ge -C\delta \frac{\langle n_+\rangle _{\Psi }}{\ell ^2} n \frac{K_H {\mathcal {M}}^{1/2}}{n^{1/2}}. \end{aligned}$$

We use Theorem 7.6 and (H17) to bound $n^{1/2}$ by $2 \rho _\mu ^{1/2} \ell$ and get

$$\begin{aligned} \langle \Psi \,|\, ({\mathcal {Q}}_3^{\text {low}} - {\mathcal {Q}}_3^{\text {high}} ) \Psi \rangle \ge -C\delta \rho _\mu ^{1/2} \ell K_H {\mathcal {M}}^{1/2} \frac{\langle n_+\rangle _{\Psi }}{\ell ^2}. \end{aligned}$$

By the assumption (H16) the error can be absorbed in a small fraction of the spectral gap. $\square$

We are ready to state a bound for the second quantized Hamiltonian.

#### Proposition 8.3

Assume $R \ll (\rho _\mu \delta )^{-1/2}$ and the relations of “Appendix H” between the parameters. Let $\Psi$ be a normalized *n*-particle state satisfying (7.40) and $\Psi = \mathbb {1}_{[0,{\mathcal {M}}]}(n_+^L)\Psi$. Then

8.26 $$\begin{aligned} \langle \Psi \,|\, {\mathcal {H}}_{\Lambda }(\rho _{\mu }) \Psi \rangle \ge \langle \Psi \,|\, {\mathcal {H}}_{\Lambda }^{\text {2nd}}(\rho _{\mu }) \Psi \rangle -C \ell ^2 \rho _{\mu }^2 \delta \Big ( d^{2M-2} + R^2 \ell ^{-2} \Big ), \end{aligned}$$

where

8.27 $$\begin{aligned} {\mathcal {H}}_{\Lambda }^{\text {2nd}}&:= \frac{\ell ^2}{(2\pi )^2} \int _{{\mathbb {R}}^2} (1-\varepsilon _{N}) \tau (k) a^{\dagger }_k a_k \textrm{d}k + \frac{b}{2\ell ^2} n_+ +b \frac{\varepsilon _T}{8 d^2 \ell ^2 } n_+^H +b \frac{\varepsilon _T n_0 n_+^H}{16 d^2 \ell ^2 (\rho _{\mu }\ell ^2)} \end{aligned}$$

8.28 $$\begin{aligned}&\quad + \frac{1}{2\ell ^2} a_0^{\dagger }a_0^{\dagger }a_0 a_0 ({\widehat{g}}_0 + \widehat{g\omega }(0)) -\rho _{\mu } a_0^{\dagger }a_0 {\widehat{g}}_0 \end{aligned}$$

8.29 $$\begin{aligned}&\quad + \Big ( \Big ( \frac{1}{\ell ^2} a_0^{\dagger }a_0 - \rho _{\mu }\Big ) {\widehat{W}}_1(0) \frac{1}{(2\pi )^2} \int _{{\mathbb {R}}^2} {\widehat{\chi }}_{\Lambda }(k) a^{\dagger }_k a_0 \textrm{d}k + h.c. \Big ) \end{aligned}$$

8.30 $$\begin{aligned}&\quad +\Big ( \frac{1}{\ell ^2} a_0^{\dagger }a_0 \widehat{\omega W_1}(0) \frac{1}{(2\pi )^2} \int _{{\mathbb {R}}^2} {\widehat{\chi }}_{\Lambda }(k) a^{\dagger }_k a_0 \textrm{d}k + h.c. \Big ) \end{aligned}$$

8.31 $$\begin{aligned}&\quad +{\mathcal {Q}}_2^{\text {rest}} + {\mathcal {Q}}_3^{\text {high}} \end{aligned}$$

8.32 $$\begin{aligned}&\quad + \Big (\Big (\frac{1}{\ell ^2}a^{\dagger }_0 a_0 - \rho _{\mu }\Big ){\widehat{W}}_1(0) + \frac{1}{\ell ^2}a^{\dagger }_0 a_0 \widehat{W_1 \omega }(0)\Big )\frac{\ell ^2}{(2\pi )^2} \int _{{\mathbb {R}}^2} a^{\dagger }_k a_k \textrm{d}k, \end{aligned}$$

where $\tau (k)$ is defined in (8.8) and with

$$\begin{aligned} {\mathcal {Q}}_2^{\text {rest}}= \frac{1}{(2\pi )^2} \int _{{\mathbb {R}}^2} ({\widehat{W}}_1(k)&+ \widehat{(W_1\omega )}(k)) a^{\dagger }_0 a^{\dagger }_k a_k a_0 \textrm{d}k \\&+\frac{1}{2} \int _{{\mathbb {R}}^2} {\widehat{W}}_1(k) \Big ( a^{\dagger }_0 a^{\dagger }_0 a_k a_{-k} + a^{\dagger }_k a^{\dagger }_{-k} a_0 a_0\Big ) \textrm{d}k. \end{aligned}$$

#### Proof

We use the lower bound for ${\mathcal {H}}_{\Lambda }(\rho _{\mu })$ from Corollary 7.3. First of all, in the kinetic energy expression (6.14) we remove the positive parts depending on the Neumann Laplacian, namely $\varepsilon _N(-\Delta _{{\mathcal {N}}})$ and ${\mathcal {T}}^{\text {Neu,s}}$. Using the quantization, we obtain from (6.14) the expressions in (8.27) with the main kinetic energy term and the spectral gaps. We bounded part of the spectral gap to get the last term in (8.27) using $n_0 \le 2 \rho _\mu \ell ^2$ (which follows from (7.43) and (H17)). This term will be useful later (in particular in the proof of Lemma 9.2).

The expressions (8.28), (8.29), (8.30), ${\mathcal {Q}}_2^{\text {rest}}$ and (8.32) are obtained from (7.16), (7.17), (7.18), (7.19) and (7.20) respectively, via a straightforward application of the quantization rules. Note that in (8.29) and (8.30) we have changed a ${{\widehat{W}}}_1(k)$ (resp. $\widehat{\omega W_1}(k)$) into ${{\widehat{W}}}_1(0)$ (resp. $\widehat{\omega W_1}(0)$). This can be justified by using (6.25) in (7.17) and (7.18), the error being of order $R^2 \rho _\mu ^2 \delta$. We can reabsorb the term

$$\begin{aligned} - C(\rho _{\mu } + \rho _0) \delta R^2 \frac{n_+}{\ell ^2}, \end{aligned}$$

in a fraction of the spectral gap because $R \ll (\rho _\mu \delta )^{-1/2}$. Let us observe that thanks to Lemma 7.5 we can replace ${\mathcal {Q}}_3^{\text {ren}}+ \frac{1}{4} {\mathcal {Q}}_4^{\text {ren}}$ by ${\mathcal {Q}}_3^{\text {low}}$ in ${\mathcal {H}}_{\Lambda }(\rho _{\mu })$. Part of the error is absorbed in the spectral gap, other part appears in (8.26). Finally we change ${\mathcal {Q}}_3^{\text {low}}$ into ${\mathcal {Q}}_3^{\text {high}}$ using Lemma 8.2, the error being absorbed in a fraction of the spectral gap again. $\square$

### *c*-number substitution

In this section we show how the energy can be bounded if we minimize over a specific class of coherent states, which are eigenvectors for the annihilation operator of the condensate. In this way we can turn the action of the condensate operators in the form of multiplication per complex numbers. Let us define

8.33 $$\begin{aligned} |z\rangle = e^{-\big (\frac{|z|^2}{2} + z a^{\dagger }_0\big )}\, \Omega , \end{aligned}$$

for any $z \in {\mathbb {C}}$. As anticipated, we have

8.34 $$\begin{aligned} a_0 |z\rangle = z\, |z \rangle . \end{aligned}$$

Given any state $\Psi$ we define the *z*-dependent state

8.35 $$\begin{aligned} \Phi (z) := \langle z\,|\, \Psi \rangle , \end{aligned}$$

obtained by the partial inner product in ${\mathscr {F}}_s(\textrm{Ran}P)$. One can verify that these states generate the space ${\mathscr {F}}_s (\textrm{Ran}Q)$. Moreover,

8.36 $$\begin{aligned} 1 = \frac{1}{\pi } \int _{{\mathbb {C}}} |z\rangle \langle z| \, \textrm{d}z. \end{aligned}$$

We define the following *z*-dependent density,

8.37 $$\begin{aligned} \rho _z := \frac{|z|^2}{\ell ^2}, \end{aligned}$$

and *z*-dependent Hamiltonian,

8.38 $$\begin{aligned} {\mathcal {K}}(z)&= \frac{1}{2}\rho _z^2 \ell ^2 ({\widehat{g}}_0 + \widehat{g\omega }(0)) - \rho _{\mu }\rho _z {\widehat{g}}_0\ell ^2 \end{aligned}$$

8.39 $$\begin{aligned}&\quad +{\mathcal {K}}^{\text {Bog}} + \frac{b}{2\ell ^2} n_+ + \frac{\varepsilon _T b}{8 d^2 \ell ^2 } n_+^H +b \frac{\varepsilon _T |z|^2 n_+^H}{16 d^2 \ell ^2 (\rho _{\mu } \ell ^2)} + \varepsilon _R (\rho _{\mu } - \rho _z )^2 \delta \ell ^2 \end{aligned}$$

8.40 $$\begin{aligned}&\quad + (\rho _{z} - \rho _{\mu }) {\widehat{W}}_1(0) \frac{\ell ^2}{(2\pi )^2} \int _{{\mathbb {R}}^2} a^{\dagger }_k a_k \textrm{d}k + {\mathcal {Q}}_1^{\text {ex}}(z) + {\mathcal {Q}}_2^{\text {ex}}(z) + {\mathcal {Q}}_3(z), \end{aligned}$$

where $\varepsilon _R \ll 1$ is fixed in “Appendix H”, and

8.41 $$\begin{aligned} {\mathcal {K}}^{\text {Bog}}&:= \frac{\ell ^2}{2(2\pi )^2}\int _{{\mathbb {R}}^2} \Big ( {\mathcal {A}}(k) (a^{\dagger }_k a_{k} +a^{\dagger }_{-k} a_{-k})+ {\mathcal {B}}(k) (a_k a_{-k} + a^{\dagger }_k a^{\dagger }_{-k}) \nonumber \\&\quad \quad \quad \quad \quad \quad \quad \quad +\, {\mathcal {C}}(k) (a^{\dagger }_k + a^{\dagger }_{-k} + a_k + a_{-k}) \Big ) \textrm{d}k, \end{aligned}$$

with

8.42 $$\begin{aligned} {\mathcal {A}}(k)&:= (1-\varepsilon _N) \tau (k) + {\mathcal {B}}(k), \qquad {\mathcal {B}}(k) := \rho _z {\widehat{W}}_1(k),\nonumber \\ {\mathcal {C}}(k)&:= \frac{(\rho _z - \rho _{\mu })}{\ell ^2} {\widehat{W}}_1(0) {\widehat{\chi }}_{\Lambda }(k) z,\nonumber \\ {\mathcal {Q}}_1^{\text {ex}}(z)&:= \rho _z \widehat{(\omega W_1)}(0) \frac{1}{(2\pi )^2} \int _{{\mathbb {R}}^2} {\widehat{\chi }}_{\Lambda }(k) a^{\dagger }_k z \textrm{d}k+ h.c., \nonumber \\ {\mathcal {Q}}_2^{\text {ex}}(z)&:= \frac{\ell ^2}{(2 \pi )^2} \rho _z \int _{{\mathbb {R}}^2} (\widehat{\omega W_1}(0) + \widehat{\omega W_1}(k) ) a^{\dagger }_k a_k \textrm{d}k, \nonumber \\ {\mathcal {Q}}_3(z)&:= \frac{\ell ^2}{(2\pi )^4} \int _{{\mathcal {P}}_H \times {\mathbb {R}}^2} f_L(p) {\widehat{W}}_1(k) \Big ({\bar{z}} \widetilde{a}_p^{\dagger } a_{p-k} a_k + h.c. \Big ) \textrm{d}k \textrm{d}p \end{aligned}$$

and $\tau (k)$ defined in (8.8). With these notations, the following theorem holds. Recall that ${\mathcal {H}}_{\Lambda }^{\text {2nd}}$ is given by Proposition 8.3.

#### Theorem 8.4

Assume $R \le \ell$ and (H17). For any normalized *n*-particle state $\Psi$ satisfying $\Psi = \mathbb {1}_{[0,{\mathcal {M}}]}(n_+^L) \Psi$ and (7.40) we have

8.43 $$\begin{aligned} \langle \Psi \,|\,{\mathcal {H}}_{\Lambda }^{\text {2nd}} \Psi \rangle \ge \inf _{z \in {\mathbb {R}}_+} \inf _{\Phi } \langle \Phi \,|\, {\mathcal {K}}(z) \Phi \rangle - C \rho _{\mu } \delta (1 + \varepsilon _R K_{\ell }^4 K_B^2 |\log Y|), \end{aligned}$$

where the second infimum is over all the normalized states in ${\mathscr {F}}(\textrm{Ran}Q)$ such that

8.44 $$\begin{aligned} \Phi = \mathbb {1}_{[0,{\mathcal {M}}]}(n_+^L) \Phi , \qquad \text {and} \qquad \Phi = \mathbb {1}_{[0,2 \rho _{\mu }\ell ^2]}(n_+) \Phi . \end{aligned}$$

#### Proof

The theorem is proven via a standard technique of calculating the actions of creation and annihilation operators for the condensate on the coherent state and using its eigenvector properties, for details see [19, Theorem 8.5]. Practically speaking it consists in the formal substitutions

8.45 $$\begin{aligned} a_0 \mapsto z, \qquad a^{\dagger }_0 \mapsto {\overline{z}}, \qquad a^{\dagger }_0 a_0 \mapsto |z|^2-1, \end{aligned}$$

and getting rid of the lower order terms in |*z*| because they produce errors of the form

8.46 $$\begin{aligned} \rho _{\mu } \delta = \rho _{\mu }^2 \ell ^2 \delta ^2 K_{\ell }^{-2}. \end{aligned}$$

In order to make the last term in (8.39) appear, we add and subtract $\varepsilon _R (\rho _{\mu } - n_0 \ell ^{-2})^2 \delta \ell ^2$ to ${\mathcal {H}}_{\Lambda }^{\text {2nd}}$ and estimate the negative contribution, recalling the estimates in Theorem 7.7 and that $n_+^2 \le n n_+$ we get

$$\begin{aligned} -\varepsilon _R \Big (\rho _{\mu } - \frac{n_0}{\ell ^{2}}\Big )^2 \delta \ell ^2&\ge -2 \varepsilon _R \delta \ell ^{-2} ((\rho _{\mu }\ell ^2 - n)^2 + n_+ n) \\&\ge - C \varepsilon _R \frac{\delta }{\ell ^2} n^2 K_B^2 Y |\log Y| K_{\ell }^2 = - C \varepsilon _R \rho _{\mu }\delta K_B^2 |\log Y| K_{\ell }^4, \end{aligned}$$

which is coherent with the error terms. $\square$

## Lower Bounds for the Hamiltonian ${{\mathcal {K}}}$

### Estimate of ${\mathcal {K}}$ for $\rho _{z}$ far from $\rho _{\mu }$

The purpose of this section is to show that for values of $\rho _{z}$ far from the density $\rho _{\mu }$ it is possible to prove a rough estimate on the energy and eliminate these values from the analysis. This is the content of the proposition below. We recall that ${\mathcal {K}}(z)$ is defined in (8.38), and we use the notations $\varepsilon _{{\mathcal {M}} }= \frac{{\mathcal {M}}}{\rho _\mu \ell ^2}$ and

9.1 $$\begin{aligned} \delta _1 = \frac{\varepsilon _T^2 \varepsilon _{{\mathcal {M}}} }{d^8 K_{\ell }^4 } \Big ( 1+ \frac{K_{\ell }^2}{K_{H}^2}\Big ), \quad \delta _2 = \varepsilon _{{\mathcal {M}}}^{1/2}, \quad \delta _3 = \delta |\log (d s K_{\ell })| + \frac{(d K_{\ell })^4}{\varepsilon _T^2}. \end{aligned}$$

#### Proposition 9.1

Assume the relations between the parameters in “Appendix H”. There exists a $C>0$, such that if we have $\rho _{\mu }a^2 \le C^{-1}$ and

9.2 $$\begin{aligned} |\rho _{\mu } - \rho _z| \ge C \rho _{\mu } \max \Big ( (\delta _1 + \delta _2 + \delta _3)^{1/2}, \delta ^{1/2}\Big ), \end{aligned}$$

then for any state $\Phi \in {\mathscr {F}}(\textrm{Ran}Q)$ satisfying (8.44), we have

9.3 $$\begin{aligned} \langle \Phi \,|\, {\mathcal {K}}(z) \Phi \rangle \ge -4\pi \rho _{\mu }^2 \ell ^2 \delta + 8\pi \Big (\frac{1}{2} +2\Gamma +\log \pi \Big ) \rho _{\mu }^2 \ell ^2 \delta ^2 . \end{aligned}$$

Notice that the second order term in (9.3) is larger than the one aimed for in Theorem 6.7. So the statement of the proposition is that the energy is too large unless $|\rho _{\mu } - \rho _z|$ is small. The proof of the proposition relies on the technical estimate given by the following lemma.

#### Lemma 9.2

Assume the relations between the parameters in “Appendix H”. For any normalized $\Phi \in {\mathscr {F}}(\textrm{Ran}Q)$ such that (8.44) holds,

9.4 $$\begin{aligned} \langle \Phi \,|\, {\mathcal {K}}(z) \Phi \rangle&\ge -4\pi \rho _{\mu }^2 \ell ^2 \delta + 4\pi \ell ^2(\rho _{\mu }-\rho _z )^2 \delta - C\rho _z \rho _{\mu } \ell ^2\delta \delta _1 \nonumber \\&\quad - C \rho _{\mu }^{1/2} (\rho _{\mu } + \rho _z)^{3/2}\ell ^2 \delta \delta _2 - C\rho _z^2 \ell ^2\delta \delta _3 -C \rho _{\mu } \delta ^{2} K_{\ell }^{-2} (ds)^{-4}. \end{aligned}$$

#### Proof of Lemma 9.2

We start by estimating the $Q_1$ terms. We have for any $\varepsilon >0$

$$\begin{aligned}&\int _{{\mathbb {R}}^2} {\widehat{\chi }}_{\Lambda }(k) (a_k^{\dagger } z+ a_k {\bar{z}}) \textrm{d}k\\&\quad \le \int _{{\mathbb {R}}^2} |{\widehat{\chi }}_{\Lambda }(k)| ( \varepsilon |z|^2 +\varepsilon ^{-1} a^{\dagger }_k a_k ) \textrm{d}k \\&\quad \le C \Big (\varepsilon |z|^2 + \varepsilon ^{-1} |{\widehat{\chi }}_\Lambda (0)|\int _{k \in {\mathcal {P}}_H^c} a^{\dagger }_k a_k \textrm{d}k + \varepsilon ^{-1} \int _{k \in {\mathcal {P}}_H} |{\widehat{\chi }}_{\Lambda }(k)| a^{\dagger }_k a_k \textrm{d}k \Big ). \end{aligned}$$

Considering a $\Phi$ like in the assumption we have, using $|{\widehat{\chi }}_{\Lambda }(0)| = \ell ^2 \Vert \chi \Vert _1$ together with Lemma 8.1,

9.5 $$\begin{aligned}{} & {} \Big \langle |{\widehat{\chi }}_\Lambda (0)|\int _{k \in {\mathcal {P}}_H^c} a^{\dagger }_k a_k \textrm{d}k \; + \int _{k \in {\mathcal {P}}_H} |{\widehat{\chi }}_{\Lambda }(k)| a^{\dagger }_k a_k \textrm{d}k \Big \rangle _{\Phi } \nonumber \\{} & {} \qquad \le C \Big ({\mathcal {M}}+ \rho _{\mu } \ell ^2 \sup _{k \in {\mathcal {P}}_H} (\ell ^{-2} |{\widehat{\chi }}_{\Lambda }(k)|)\Big ). \end{aligned}$$

Now, using (F4) and optimizing with $\varepsilon = \sqrt{{\mathcal {M}}/|z|^2}$,

9.6 $$\begin{aligned}&\langle - \frac{\ell ^2}{(2\pi )^2}\int _{{\mathbb {R}}^2} \, {\mathcal {C}}(k) (a^{\dagger }_k+ a^{\dagger }_{-k} + a_k + a_{-k})\textrm{d}k + {\mathcal {Q}}_1^{\text {ex}}(z) \rangle _{\Phi } \nonumber \\&\quad \ge - C \delta \sqrt{{\mathcal {M}}} |z| (|\rho _z - \rho _{\mu }| + \rho _z) \nonumber \\&\quad \ge - C \Big (\frac{{\mathcal {M}}}{\rho _{\mu }\ell ^2}\Big )^{1/2}\rho _{\mu }^{1/2}\ell ^2 \delta (\rho _{\mu } + \rho _z)^{3/2}. \end{aligned}$$

For the terms that are quadratic in the field operators, we use the estimate

9.7 $$\begin{aligned} \left| \left\langle \ell ^2 \int _{{\mathbb {R}}^2} \, {\widehat{W}}_1(k) a^{\dagger }_k a_k \textrm{d}k\right\rangle _{\Phi }\right| \le C \delta ({\mathcal {M}} + \langle n_+^H\rangle _{\Phi }), \end{aligned}$$

from Lemma 8.1 to obtain that

9.8 $$\begin{aligned}{} & {} \left\langle {\mathcal {Q}}_2^{\text {ex}} + (\rho _{z} - \rho _{\mu }) {\widehat{W}}_1(0) \frac{\ell ^2}{(2\pi )^2} \int _{{\mathbb {R}}^2} \, a^{\dagger }_k a_k \textrm{d}k + \frac{\ell ^2}{(2 \pi )^2 }\int _{{\mathbb {R}}^2} \, {\mathcal {B}}_k a^{\dagger }_k a_k \textrm{d}k \right\rangle _{\Phi } \nonumber \\{} & {} \quad \ge - C (\rho _z + \rho _{\mu }) \big ( \rho _{\mu }\ell ^2 \delta \varepsilon _{{\mathcal {M}}} + \delta \langle n_+^H\rangle _{\Phi } \big ), \end{aligned}$$

where the ${\mathcal {B}}_k$ has been extracted from the expression of the ${\mathcal {A}}_k$. The first term is coherent with the error in the result and the last one can be reabsorbed in a fraction of the spectral gap because of relation (H8).

For the remaining part of ${\mathcal {A}}_k$ involving $\tau _k$ we add and subtract $-\rho _z \delta \varepsilon ^{-1/2} + \varepsilon \tau _k$, with $\varepsilon \ge \varepsilon _N$ and estimate

9.9 $$\begin{aligned} (1-\varepsilon _N) \tau _k&\ge \widetilde{{\mathcal {A}}}_k -\rho _z \delta \varepsilon ^{-1/2} + \varepsilon \tau _k, \end{aligned}$$

with

9.10 $$\begin{aligned} \widetilde{{\mathcal {A}}}_k&= (1-2 \varepsilon ) \Big [ |k| - \frac{1}{2 d s \ell }\Big ]_+^2 + \rho _z \delta \varepsilon ^{-1/2}. \end{aligned}$$

We treat the terms in (9.9) separately, adding them to the remaining parts of the Hamiltonian. The simplest one is

9.11 $$\begin{aligned} -\frac{\ell ^2}{(2\pi )^2}\rho _z \varepsilon ^{-1/2}\delta \Big \langle \int _{{\mathbb {R}}^2} \, a^{\dagger }_k a_k \textrm{d}k \Big \rangle _{\Phi } \ge - C \varepsilon ^{-1/2} \rho _z \delta ({\mathcal {M}} + \langle n_+^H \rangle _{\Phi }), \end{aligned}$$

where we used Lemma 8.1. We use this estimate to fix the choice of $\varepsilon$ in order to absorb the last term in the fraction of the spectral gap represented by the second to last term in (8.39). This yields

9.12 $$\begin{aligned} \varepsilon = C^{-1}\varepsilon _T^{-2} (d K_{\ell })^4, \end{aligned}$$

for some sufficiently large constant *C* and the relations (H23), (H8) ensure that $\varepsilon _N \le \varepsilon \ll 1$. For the $\widetilde{{\mathcal {A}}}$ term plus the *B* terms in the Hamiltonian we use the Bogoliubov diagonalization procedure stated in Theorem B.1 to obtain

9.13 $$\begin{aligned} \frac{\ell ^2}{(2 \pi )^2} \int _{{\mathbb {R}}^2} \widetilde{{\mathcal {A}}}_k a^{\dagger }_k a_k + \frac{{\mathcal {B}}_k}{2} (a^{\dagger }_k a^{\dagger }_{-k} + a_{k} a_{-k}) \,\textrm{d}k \ge -\frac{\ell ^2}{2(2 \pi )^2} \int _{{\mathbb {R}}^2} \widetilde{{\mathcal {A}}}_k - \sqrt{\widetilde{{\mathcal {A}}}_k^2 - {\mathcal {B}}_k^2} \,\textrm{d}k,\nonumber \\ \end{aligned}$$

and then we use Lemma C.5 and its proof choosing the parameters $K_1= \rho _z \varepsilon ^{-1/2} /2$, $K_2 = 2 \rho _z$, $K = (2ds\ell )^{-1}$ and $\kappa = (1-2\varepsilon )$ to derive that

9.14 $$\begin{aligned}{} & {} (9.13) \ge -\frac{\ell ^2}{2(2\pi )^2} \Big (\rho _z^2 \frac{1 + \varepsilon }{1-2\varepsilon } \int _{{\mathbb {R}}^2}\textrm{d}k \frac{{\widehat{W}}_1^2(k)-{\widehat{W}}_1^2(0)\mathbb {1}_{\{|k|\le \ell _{\delta }^{-1}\}}}{2|k|^2} + C\rho _z \varepsilon ^{1/2} \delta (ds\ell )^{-2} \nonumber \\{} & {} \quad + \frac{C}{(1-2 \varepsilon )}\rho _z^2 \delta ^2(1+ R^2 \ell _{\delta }^{-2})+\frac{C \rho _z^2}{1-2\varepsilon }\delta ^2 |\log (( d s \ell )^{-1} \ell _{\delta }) |\Big ). \end{aligned}$$

Using now Cauchy–Schwarz on the second term, Lemma 6.4, writing only the dominant terms due to the relations between the parameters and recalling the definition (6.23) of $\ell _{\delta }$ we obtain

9.15 $$\begin{aligned} (9.13) \ge -\frac{1}{2}\rho _z^2 \ell ^2 \widehat{g\omega }(0) -C \rho _z^2\ell ^2 \delta \big ( \varepsilon + \delta ^2 \rho _{\mu } R^2 + \delta |\log (d s K_{\ell })|\big ) - C\delta \ell ^2(d s\ell )^{-4}. \nonumber \\ \end{aligned}$$

Due to relation (H3) the second term gives $\delta _3$, while the third one gives the last term in (9.4).

We continue considering the third term in (9.9) and adding it to the ${\mathcal {Q}}_3$. The latter is an integral for $k \in {\mathcal {P}}_H$, and dropping the part of the $\tau _k$ for $k \in {\mathcal {P}}_H^c$ and using that for $k \in {\mathcal {P}}_H$ then $\tau _k \ge |k|^2/2$, we have to estimate

9.16 $$\begin{aligned} \frac{\ell ^2}{(2\pi )^2} \int _{k \in {\mathcal {P}}_H} \, \Big ( \frac{\varepsilon }{2} k^2 a^{\dagger }_k a_k + \frac{1}{(2\pi )^2} \int \, f_L(p) {\widehat{W}}_1(k) ({\bar{z}} \widetilde{a}^{\dagger }_p a_{p-k} a_k + a^{\dagger }_k a^{\dagger }_{p-k} \widetilde{a}_p z) \Big )\textrm{d}p \textrm{d}k.\nonumber \\ \end{aligned}$$

We complete the square in the previous expression, introducing the operators

9.17 $$\begin{aligned} \sigma _k := a_k + \frac{2}{(2\pi )^2} \int \,f_L(p) \frac{{\widehat{W}}_1(k)}{\varepsilon |k|^2} z a_{p-k}^{\dagger } \widetilde{a}_p \textrm{d}p, \end{aligned}$$

so that

$$\begin{aligned} (9.16) =&\, \frac{\ell ^2}{(2\pi )^2} \int _{k \in {\mathcal {P}}_H} \, \Big ( \frac{\varepsilon }{2} k^2 \sigma ^{\dagger }_k \sigma _k \\&- \frac{2|z|^2}{\varepsilon (2\pi )^4} \iint f_L(p) f_L(s) \frac{{\widehat{W}}_1(k)^2}{k^2} \widetilde{a}_s^{\dagger } a_{s-k} a^{\dagger }_{p-k}\widetilde{a}_p\Big ) \textrm{d}p \textrm{d}s \textrm{d}k \\ \ge&\,- \frac{2|z|^2\ell ^2}{\varepsilon (2\pi )^6} \int _{k \in {\mathcal {P}}_H} \, \frac{{\widehat{W}}_1(k)^2}{k^2} \\&\iint f_L(p) f_L(s) \widetilde{a}_s^{\dagger }(a^{\dagger }_{p-k} a_{s-k} + [a_{s-k}, a^{\dagger }_{p-k}] )\widetilde{a}_p \textrm{d}p \textrm{d}s \textrm{d}k. \end{aligned}$$

For the term without commutator, estimated on a state $\Phi$ which satisfies (8.44) and using Cauchy–Schwarz

9.18 $$\begin{aligned} \widetilde{a}_s^{\dagger }a^{\dagger }_{p-k} a_{s-k}\widetilde{a}_p \le C (\widetilde{a}_s^{\dagger }a^{\dagger }_{p-k} a_{p-k}\widetilde{a}_s + \widetilde{a}_p^{\dagger }a^{\dagger }_{s-k} a_{s-k}\widetilde{a}_p), \end{aligned}$$

we have

9.19 $$\begin{aligned} \frac{2|z|^2\ell ^2}{\varepsilon (2\pi )^6}&\left\langle \int _{k \in {\mathcal {P}}_H} dk\, \frac{{\widehat{W}}_1(k)^2}{k^2} \iint f_L(p) f_L(s) \widetilde{a}_s^{\dagger }a^{\dagger }_{p-k} a_{p-k}\widetilde{a}_s \textrm{d}p\textrm{d}s\right\rangle _{\Phi } \nonumber \\&\le C|z|^2 \varepsilon ^{-1} \frac{\ell ^4 \delta ^2}{K_H^2} \left\langle \int _{k \in {\mathcal {P}}_H} \int f_L(s) \widetilde{a}_s^{\dagger }a^{\dagger }_{k} a_{k}\widetilde{a}_s \textrm{d}s \textrm{d}k \right\rangle _{\Phi } \int _{p \in {\mathcal {P}}_L} \textrm{d}p \nonumber \\&\le C \varepsilon ^{-1} \frac{\delta ^2}{K_H^2} d^{-4} {\mathcal {M}} \rho _{\mu }\rho _z\ell ^2, \end{aligned}$$

where we used Lemma 8.1 since the support of $f_L$ is included in the complement of ${\mathcal {P}}_H$, and the estimate, for $k \in {\mathcal {P}}_H$,

9.20 $$\begin{aligned} \frac{{\widehat{W}}_1(k)^2}{2 k^2} \le C K_H^{-2} \delta ^2 \ell ^2. \end{aligned}$$

For the commutator part we use the estimate (8.6), the Cauchy–Schwarz inequality

9.21 $$\begin{aligned} \widetilde{a}_s^{\dagger } [a_{s-k}, a^{\dagger }_{p-k}] \widetilde{a}_p \le C \widetilde{a}_s^{\dagger }\widetilde{a}_s + C\widetilde{a}_p^{\dagger } \widetilde{a}_p, \end{aligned}$$

and Lemma 3.9 applied to ${\widehat{W}}_1$ instead of ${\widehat{g}}$ paying a small error, we get

9.22 $$\begin{aligned}{} & {} - \frac{2|z|^2\ell ^2}{\varepsilon (2\pi )^6} \left\langle \int _{k \in {\mathcal {P}}_H} \frac{{\widehat{W}}_1(k)^2}{k^2} \iint f_L(p) f_L(s) \widetilde{a}_s^{\dagger } [a_{s-k}, a^{\dagger }_{p-k}] \widetilde{a}_p \textrm{d}p \textrm{d}s \textrm{d}k \right\rangle _{\Phi } \nonumber \\{} & {} \quad \ge - C\frac{|z|^2\ell ^2}{\varepsilon } \delta \left\langle \iint f_L(p) f_L(s) \widetilde{a}_p^{\dagger } \widetilde{a}_p \textrm{d}p\textrm{d}s \right\rangle _{\Phi } \ge - C \varepsilon ^{-1}\rho _z \delta {\mathcal {M}} d^{-4}, \end{aligned}$$

where in the last inequality we used Lemma 8.1.

Collecting formulas (9.6), (9.8), (9.15), (9.19) and (9.22) and observing that

9.23 $$\begin{aligned} \frac{1}{2} \rho _z^2 \ell ^2 {\widehat{g}}_0 -\rho _z\rho _{\mu } \ell ^2 {\widehat{g}}_0 = \frac{1}{2} (\rho _z - \rho _{\mu })^2 \ell ^2 {\widehat{g}}_0 - \frac{1}{2} \rho _{\mu }^2\ell ^2 {\widehat{g}}_0, \end{aligned}$$

we obtain the result. $\square$

#### Proof of Proposition 9.1

We observe that, thanks to the relations (H6), (H8), (H22), we have $\delta _j \ll 1$ for $j =1,2,3$. Each coefficient of the $\delta _j$ in formula (9.4) can be bounded by

9.24 $$\begin{aligned} C \delta (\rho _{\mu } -\rho _z)^2\ell ^2 +C \rho _{\mu }^2 \ell ^2 \delta . \end{aligned}$$

Therefore, Lemma 9.2 and ${\widehat{g}}_0 = 8 \pi \delta$ implies the bound

$$\begin{aligned} \langle {\mathcal {K}}(z)\rangle _{\Phi } \ge&-\frac{1}{2} \rho _{\mu }^2 \ell ^2 {\widehat{g}}_0 + \frac{1}{2} (\rho _{\mu } - \rho _z)^2 \ell ^2 {\widehat{g}}_0( 1- C (\delta _1 + \delta _2 + \delta _3 ) ) \\&- C \rho _{\mu }^2 \ell ^2 \delta (\delta _1 + \delta _2 + \delta _3 + \delta ^2 (K_\ell d s)^{-4}) \\ \ge&-\frac{1}{2} \rho _{\mu }^2 \ell ^2 {\widehat{g}}_0 + \frac{1}{4} \ell ^2 {\widehat{g}}_0(\rho _{\mu } - \rho _z)^2 - C \rho _{\mu }^2 \ell ^2 \delta (\delta _1 + \delta _2 + \delta _3 + \delta ^{2}(K_\ell d s)^{-4}). \end{aligned}$$

Note that $\delta ^{2}(K_\ell d s)^{-4} \ll \delta$ due to (H12) and (H17). By the assumption on $(\rho _{\mu }-\rho _z)^2$ the second term is of higher order both of the $\delta _j$ errors and of the desired quantity in the statement of the Proposition. $\square$

### Estimate of ${\mathcal {K}}$ for $\rho _{z} \simeq \rho _{\mu }$

We study here the main case, that is when $\rho _{z}$ is close to $\rho _{\mu }$. More precisely, we consider the complementary situation to (9.2), when

9.25 $$\begin{aligned} |\rho _{\mu } - \rho _z| \le K_{\ell }^{-2} \rho _{\mu }, \end{aligned}$$

where we used that, thanks to the choices of the parameters (H8), (H17) and (H22), we have

9.26 $$\begin{aligned} K_{\ell }^2 \max \Big ( (\delta _1 + \delta _2 + \delta _3)^{1/2}, \delta ^{1/2}\Big ) \le C^{-1}. \end{aligned}$$

Using again (9.23) and reabsorbing the term $(\rho _z - \rho _{\mu }) {\widehat{W}}_1(0) \frac{\ell ^2}{(2\pi )^2} \int a^{\dagger }_k a_k \textrm{d}k$ in part of the spectral gap of $n_+$, we have the estimate of ${\mathcal {K}}(z)$ from (8.38),

9.27 $$\begin{aligned} {\mathcal {K}}(z) \ge&-\frac{1}{2} \rho _{\mu }^2 \ell ^2 {\widehat{g}}_0 + \frac{1}{2} \rho _z^2 \ell ^2 \widehat{g\omega }(0) + \frac{1}{2} (\rho _z- \rho _{\mu })^2 \ell ^2 {\widehat{g}}_0 \nonumber \\&+{\mathcal {K}}^{\text {Bog}} + \frac{b}{4\ell ^2} n_+ +b \frac{\varepsilon _T}{8 d^2 \ell ^2 } n_+^H +b \frac{\varepsilon _T \vert z \vert ^2 n_+^H}{16 d^2 \ell ^2 (\rho _{\mu } \ell ^2)} + \varepsilon _R (\rho _{\mu } - \rho _z )^2 \delta \ell ^2 \nonumber \\&+ {\mathcal {Q}}_1^{\text {ex}}(z) + {\mathcal {Q}}_2^{\text {ex}}(z) + {\mathcal {Q}}_3(z), \end{aligned}$$

and in the following we want to give a lower bound for the expression above using a diagonalization method for the Bogoliubov Hamiltonian. In order to do that, let us introduce a couple of new creation and annihilation operators

9.28 $$\begin{aligned} b_k&:=\frac{1}{\sqrt{1-\alpha _k^2}} (a_k + \alpha _k a^{\dagger }_{-k} + c_k), \end{aligned}$$

where

$$\begin{aligned} \alpha _k&:= {\mathcal {B}}(k)^{-1} \Big ( {\mathcal {A}}(k) - \sqrt{{\mathcal {A}}(k)^2 - {\mathcal {B}}(k)^2}\Big ), \\ c_k&:= \frac{2 {\mathcal {C}}(k)}{{\mathcal {A}}(k) + {\mathcal {B}}(k) + \sqrt{{\mathcal {A}}(k)^2 - {\mathcal {B}}(k)^2}} \mathbb {1}_{\{|k| \le \frac{1}{2}K_H \ell ^{-1}\}}, \end{aligned}$$

with ${\mathcal {A}},{\mathcal {B}},{\mathcal {C}}$ are defined in (8.41) and the diagonalized Bogoliubov Hamiltonian

9.29 $$\begin{aligned} {\mathcal {K}}^{\text {Diag}}_H := \frac{\ell ^2}{(2\pi )^2} \int _{\{ \vert k \vert \ge \frac{1}{2} K_H \ell ^{-1} \}} {\mathcal {D}}(k) b^{\dagger }_k b_k \textrm{d}k, \end{aligned}$$

where

9.30 $$\begin{aligned} {\mathcal {D}}(k) := \frac{1}{2} \big ( {\mathcal {A}}(k) + \sqrt{{\mathcal {A}}(k)^2 - {\mathcal {B}}(k)^2}\big ). \end{aligned}$$

#### Theorem 9.3

Assume the relations between the parameters in “Appendix H”. For any state $\Phi \in {\mathscr {F}}_s(L^2(\Lambda ))$ such that (8.44) holds and $\frac{9}{10} \rho _{\mu } \le \rho _z \le \frac{11}{10}\rho _{\mu }$ we have

$$\begin{aligned} \langle {\mathcal {K}}^{\text {Bog}}&\rangle _{\Phi } + \frac{1}{2} \rho _z^2 \ell ^2 (\widehat{g \omega })_0 + \frac{1}{2} (\rho _z- \rho _{\mu })^2 \ell ^2 {\widehat{g}}_0 \\&\ge (1 - \varepsilon _K) \left\langle {\mathcal {K}}^{\text {Diag}}_H\right\rangle _{\Phi } + 4 \pi \Big ( 2 \Gamma + \frac{1}{2} + \log \pi \Big ) \rho ^2_{z} \ell ^2 \delta ^2 \\&\quad - C (\rho _{\mu }-\rho _z)^2 \ell ^2 \delta ^2 \rho _{\mu } R^2 -C \rho _{\mu }^2 \ell ^2 \delta ( K_H^{4-M}K_\ell \delta ^{-1/2}) + C r(\rho _{\mu }) \ell ^2, \end{aligned}$$

where the error term is given by

$$\begin{aligned} r(\rho _{\mu }) := \rho ^2_{\mu } \delta ^2 \big ( \delta \vert \log (\delta ) \vert R^2 \rho _{\mu } +\delta \vert \log (\delta ) \vert + d + \varepsilon _T \vert \log \delta \vert + (sK_\ell )^{-1} + \varepsilon _N \delta ^{-1}\big ). \end{aligned}$$

In the proof of Theorem 9.3 we are going to use the following formulas and estimates for the commutators of the operators, recalling that ${\widehat{\chi }}_{\Lambda }$ is even,

9.31 $$\begin{aligned} {[}b_k,b_h]&= \frac{\alpha _k - \alpha _h}{\sqrt{1-\alpha _k^2}\sqrt{1-\alpha _h^2}} \Big ( \widehat{(\chi ^2)}((k+h)\ell ) -{\widehat{\chi }}(k \ell ) {\widehat{\chi }}(h \ell )\Big ), \end{aligned}$$

9.32 $$\begin{aligned} {[}b_k,b_h^{\dagger }]&= \frac{1 - \alpha _k\alpha _h}{\sqrt{1-\alpha _k^2}\sqrt{1-\alpha _h^2}} \Big ( \widehat{(\chi ^2)}((k-h)\ell ) -{\widehat{\chi }}(k \ell ) {\widehat{\chi }}(h \ell )\Big ), \end{aligned}$$

9.33 $$\begin{aligned} {[}\widetilde{a}^{\dagger }_p,b^{\dagger }_k]&= \frac{\alpha _{k} }{\sqrt{1-\alpha ^2_k}}[\widetilde{a}_p^{\dagger }, a_k]= \frac{\alpha _{k} }{\sqrt{1-\alpha ^2_k}} \ell ^{-2} \langle e^{ipx}, Q \chi _{\Lambda } e^{ikx}\rangle , \end{aligned}$$

9.34 $$\begin{aligned} {[}\widetilde{a}_p, b^{\dagger }_{-k}]&= \frac{1}{\sqrt{1-\alpha _k^2}}[\widetilde{a}_p,a^{\dagger }_{-k}] = \frac{1}{\sqrt{1-\alpha _k^2}}({\widehat{\chi }}((p+k) \ell ) - {\widehat{\theta }}(p\ell ){\widehat{\chi }}(k\ell )). \end{aligned}$$

#### Proof

Let us start by showing that the contribution coming from the ${\mathcal {C}}(k)$ gives an error term for $|k| > \frac{1}{2} K_H \ell ^{-1}$.

By Cauchy–Schwarz we have $a^{\dagger }_k + a_k \le a^{\dagger }_k a_k +1$ and then we recognize $n_+$ (8.7),

$$\begin{aligned} \frac{\ell ^2}{2(2\pi )^2}&\int _{\{|k|> \frac{1}{2} K_H \ell ^{-1}\}} {\mathcal {C}}(k) (a^{\dagger }_k + a^{\dagger }_{-k} + a_k + a_{-k}) \, \textrm{d}k \\&\ge -C |\rho _{\mu }-\rho _z| |{\widehat{W}}_1(0)| |z| \int _{\{|k| > \frac{1}{2} K_H \ell ^{-1}\}} |\widehat{\chi _{\Lambda }}(k)| (a^{\dagger }_k a_k + 1) \,\textrm{d}k \\&\ge - C \rho _{\mu } \delta |z| (n_+ +1) K_H^{4-M}, \end{aligned}$$

where we use the assumption on $\rho _z$ and that by Lemma F.1,

9.35 $$\begin{aligned} \ell ^{-2} \sup _{|k| > \frac{1}{2}K_H \ell ^{-1}} (1+ (k\ell )^2)^2 |\widehat{\chi _{\Lambda }}(k)| \le C K_H^{4-M}. \end{aligned}$$

When we apply to $\Phi$ we have $n_+ \le 2 \rho _{\mu }\ell ^2$ and

9.36 $$\begin{aligned} \frac{\ell ^2}{2(2\pi )^2}\int _{\{|k| > \frac{1}{2} K_H \ell ^{-1}\}} {\mathcal {C}}(k) \langle a^{\dagger }_k + a^{\dagger }_{-k} + a_k + a_{-k} \rangle _\Phi \, \textrm{d}k \ge - C \rho _\mu ^2 \ell ^2 \delta (K_H^{4-M} \sqrt{\rho _\mu } \ell ).\nonumber \\ \end{aligned}$$

Therefore

9.37 $$\begin{aligned} {\mathcal {K}}^{\text {Bog}} \ge \widetilde{{\mathcal {K}}}^{\text {Bog}} -C \rho _{\mu }^2 \ell ^2 \delta ( K_H^{4-M} K_\ell \delta ^{-1/2}), \end{aligned}$$

where $\widetilde{{\mathcal {K}}}^{\text {Bog}}$ is the same as ${\mathcal {K}}^{\text {Bog}}$ but with ${\mathcal {C}}(k)$ substituted by

9.38 $$\begin{aligned} \widetilde{{\mathcal {C}}}(k) := {\mathcal {C}}(k) \mathbb {1}_{\{|k|\le \frac{1}{2}K_H \ell ^{-1}\}}. \end{aligned}$$

The bound on the commutator (8.6) allows us to use Theorem B.1 to diagonalize the Bogoliubov Hamiltonian

$$\begin{aligned} \widetilde{{\mathcal {K}}}^{\text {Bog}}&\ge \widetilde{{\mathcal {K}}}^{\text {Diag}} - \frac{\ell ^2}{2(2\pi )^2} \int _{{\mathbb {R}}^2} \Big ( {\mathcal {A}}(k) - \sqrt{{\mathcal {A}}(k)^2 - {\mathcal {B}}(k)^2}\Big ) \textrm{d}k \\&\quad - (\rho _z - \rho _{\mu })^2 {\widehat{W}}_1(0)^2 \frac{z^2}{(2\pi )^2 \ell ^2} \int _{\{|k|\le \frac{1}{2}K_H \ell ^{-1}\}} \frac{|\widehat{\chi _{\Lambda }}(k)|^2}{{\mathcal {A}}(k) + {\mathcal {B}}(k)} \textrm{d}k, \end{aligned}$$

where

9.39 $$\begin{aligned} \widetilde{ {\mathcal {K}}}^{\text {Diag}} = \frac{\ell ^2}{(2\pi )^2} \int (1-\alpha _k^2 ) {\mathcal {D}}_k b_k^\dagger b_k \textrm{d}k \ge \frac{\ell ^2}{(2\pi )^2} \int _{\{ \vert k \vert > \frac{1}{2} K_H \ell ^{-1} \}} (1-\alpha _k^2 ) {\mathcal {D}}_k b_k^\dagger b_k \textrm{d}k. \end{aligned}$$

Using the inequality $\vert \alpha _k \vert \le C \rho _z \delta k^{-2} \le C K_\ell ^2 K_H^{-2}$ we find

9.40 $$\begin{aligned} \widetilde{ {\mathcal {K}}}^{\text {Diag}} \ge {\mathcal {K}}^{\text {Diag}}_H (1 - C K_\ell ^4 K_H^{-4} ). \end{aligned}$$

The calculation of the Bogoliubov integral is given in “Appendix C”. Combining the results of Lemma C.1, Lemma C.2 and Proposition C.3 and multiplying everything by $\ell ^2$ we find

9.41 $$\begin{aligned}{} & {} - \frac{\ell ^2}{2(2\pi )^2} \int _{{\mathbb {R}}^2} \Big ( {\mathcal {A}}(k) - \sqrt{{\mathcal {A}}(k)^2 - {\mathcal {B}}(k)^2}\Big ) \textrm{d}k+ \frac{1}{2}\widehat{g\omega }(0) \rho _z^2 \ell ^2 \nonumber \\{} & {} \quad \ge 4 \pi \Big ( 2 \Gamma + \frac{1}{2} + \log \pi \Big ) \rho _z^2 \ell ^2 \delta ^2 + r(\rho _{\mu }) \ell ^2, \end{aligned}$$

where $r(\rho _{\mu })$ is defined in the statement of the theorem. For the remaining term we use the estimate

9.42 $$\begin{aligned} {\mathcal {A}}(k) + {\mathcal {B}}(k) \ge 2 \rho _z {\widehat{W}}_1(k) \ge 2 \rho _z {\widehat{W}}_1(0) (1 - C\delta (kR)^2), \end{aligned}$$

where we used a Taylor expansion and the fact that $W_1$ is even. By this last estimate, together with Lemma F.1 and (6.24) we obtain

$$\begin{aligned} - (\rho _z - \rho _{\mu })^2 {\widehat{W}}_1^2(0)&\frac{z^2}{(2\pi )^2 \ell ^2} \int _{\{|k|\le \frac{1}{2}K_H \ell ^{-1}\}} \frac{|\widehat{\chi _{\Lambda }}(k)|^2}{{\mathcal {A}}(k) + {\mathcal {B}}(k)}\\&\ge - (\rho _z - \rho _{\mu })^2 \frac{{\widehat{W}}_1(0)}{2} \ell ^2 \big ( 1 + C \rho _{\mu }\delta ^2 R^2 K_H^2 K_\ell ^{-2} \big )\\&\ge - (\rho _z - \rho _{\mu })^2 \frac{{\widehat{g}}(0)}{2} \ell ^2 \big ( 1 + C \rho _{\mu } \delta R^2 \big ), \end{aligned}$$

where in the last line we used $K_H \ll \delta ^{-1/2}$ from (H16). $\square$

### Contribution of ${\mathcal {Q}}_3$

The aim of this section is to bound the 3*Q* term from below, namely

$$\begin{aligned} {\mathcal {Q}}_3(z) = \frac{{{\bar{z}}} \ell ^2}{(2\pi )^4} \int _{{\mathcal {P}}_H \times {\mathbb {R}}^2} {{\widehat{W}}}_1(k) f_L(p) (\widetilde{a}_p^\dagger a_{p-k} a_k + \text {h.c.}) \textrm{d}k \textrm{d}p, \end{aligned}$$

which turns out to be controlled by the quadratic Hamiltonian ${\mathcal {K}}_H^{\text {Diag}}$ defined in (9.29), absorbing ${\mathcal {Q}}_2^{\text {ex}}$ and ${\mathcal {Q}}_1^{\text {ex}}$. More precisely we prove

#### Theorem 9.4

Assume the relations between the parameters in “Appendix H” to be satisfied. Then there exists a universal constant $C >0$ such that for any state $\Phi$ satisfying (8.44) we have

$$\begin{aligned}&\left\langle (1- \varepsilon _K) {\mathcal {K}}_H^{\text {Diag}} + {\mathcal {Q}}_3(z) + {\mathcal {Q}}_2^{ex} + {\mathcal {Q}}_1^{ex} + \frac{b}{100} \frac{n_+}{\ell ^2} + \frac{\varepsilon _T b}{100}\frac{ n_+^{H}}{(d\ell )^{2}} \right\rangle _{\Phi } \\&\quad \quad \ge - C \rho _z^2 \ell ^2 \delta ^2 \Big ( \delta K_H^{-8} K_{\ell }^{10} d^{-4} + \varepsilon _K^{-1} K_H^{-12} K_\ell ^{10} d^{-8} + d^{-8}K_{\ell }^2 K_H^{-4} \\&\qquad \quad + \varepsilon _K^{-1} K_H^{-2M-8} K_\ell ^6 d^{-8} + \delta K_\ell ^2 \vert \log \delta \vert ^2 + \delta ^{-1} K_\ell ^2 d^{8M-2} \varepsilon _T^{-1} \\&\qquad \quad + \varepsilon _{{\mathcal {M}}}^{1/2} (K_\ell ^4 K_H^{-4} + \delta ^{-1} K_H^{-M} d^{-2}) \Big ). \end{aligned}$$

#### Remark 9.5

Note that we control ${\mathcal {Q}}_3 + {\mathcal {Q}}_2^{\textrm{ex}}$ using a large fraction of ${\mathcal {K}}_H^{\text {Diag}}$. It is important to remember that ${\mathcal {K}}_H^{\text {Diag}}$ is not the kinetic energy, but the Hamiltonian arising from the Bogoliubov diagonalization—sometimes ${\mathcal {K}}^{\text {Diag}}$ is called the excitation Hamiltonian. The kinetic energy is already contributing to main order in the energy, and we use it to obtain the LHY term (Theorem 9.3). The operator ${\mathcal {K}}_H^{\text {Diag}}$ is much smaller than the kinetic energy, and this is why we can use all of it to control ${\mathcal {Q}}_3 + {\mathcal {Q}}_2^{\textrm{ex}}$.

In order to prove this theorem, we start by rewriting ${\mathcal {Q}}_3(z)$ in terms of the $b_k$’s defined in (9.28). Notice that $c_k = c_{p-k} = 0$ if $k \in {\mathcal {P}}_H$ and $p \in {\mathcal {P}}_L$, and

9.43 $$\begin{aligned} a_k = \frac{b_k -\alpha _k b_{-k}^\dagger }{\sqrt{1- \alpha _k^2}}, \qquad a_{p-k} = \frac{b_{p-k} -\alpha _{p-k} b_{k-p}^\dagger }{\sqrt{1- \alpha _{p-k}^2}}. \end{aligned}$$

Therefore,

$$\begin{aligned}a_{p-k} a_k = \frac{ b_{p-k} b_k - \alpha _k b_{p-k} b_{-k}^\dagger - \alpha _{p-k} b_{k-p}^\dagger b_k + \alpha _{p-k} \alpha _k b_{k-p}^\dagger b_{-k}^\dagger }{\sqrt{1-\alpha _k^2}\sqrt{1-\alpha _{p-k}^2}} ,\end{aligned}$$

and $${\mathcal {Q}}_3(z) = {\mathcal {Q}}_3^{(1)} + {\mathcal {Q}}_3^{(2)} + {\mathcal {Q}}_3^{(3)} + {\mathcal {Q}}_3^{(4)}$$ where

9.44 $$\begin{aligned} {\mathcal {Q}}_3^{(1)}&= \frac{z \ell ^2}{(2\pi )^4} \int _{{\mathcal {P}}_H \times {\mathbb {R}}^2} \frac{f_L(p) {\widehat{W}}_1(k)}{\sqrt{1-\alpha _k^2}\sqrt{1-\alpha _{p-k}^2}} \big ( {\widetilde{a}}_p^\dagger b_{p-k} b_k + \alpha _k \alpha _{p-k} {\widetilde{a}}_p^\dagger b_{k-p}^\dagger b_{-k}^\dagger + h.c. \big ), \end{aligned}$$

9.45 $$\begin{aligned} {\mathcal {Q}}_3^{(2)}&= -\frac{z \ell ^2}{(2\pi )^4} \int _{{\mathcal {P}}_H \times {\mathbb {R}}^2} \frac{f_L(p) {\widehat{W}}_1(k) \alpha _k}{\sqrt{1-\alpha _k^2} \sqrt{1-\alpha _{p-k}^2}} \big ( \widetilde{a}^\dagger _p b^\dagger _{-k} b_{p-k} + b_{p-k}^\dagger b_{-k} \widetilde{a}_p \big ), \end{aligned}$$

9.46 $$\begin{aligned} {\mathcal {Q}}_3^{(3)}&= -\frac{z \ell ^2}{(2\pi )^4} \int _{{\mathcal {P}}_H \times {\mathbb {R}}^2} \frac{f_L(p) {\widehat{W}}_1(k) \alpha _{p-k}}{\sqrt{1-\alpha _k^2} \sqrt{1-\alpha _{p-k}^2}} \big ( \widetilde{a}^\dagger _p b^\dagger _{k-p} b_{k} + b_{k}^\dagger b_{k-p} \widetilde{a}_p \big ), \end{aligned}$$

9.47 $$\begin{aligned} {\mathcal {Q}}_3^{(4)}&= - \frac{z \ell ^2}{(2\pi )^4} \int _{{\mathcal {P}}_H \times {\mathbb {R}}^2} \frac{f_L(p) {\widehat{W}}_1(k)}{\sqrt{1-\alpha _k^2} \sqrt{1-\alpha _{p-k}^2}} \alpha _k [b_{p-k} , b_{-k}^\dagger ] ( \widetilde{a}_p^\dagger + {\widetilde{a}}_p) . \end{aligned}$$

In the remaining of this section, we get lower bounds on those four terms (Lemmas 9.7, 9.9 and 9.11 below) hence proving Theorem 9.4.

We collect here some important technical estimates which are going to be useful in the following.

#### Lemma 9.6

The following bounds hold:

9.48 $$\begin{aligned}&|\alpha _k| \le C\rho _z \delta |k|^{-2} \le C K_{\ell }^2 K_H^{-2}, \quad \text {for } |k| \ge \frac{1}{2} K_H \ell ^{-1}, \end{aligned}$$

9.49 $$\begin{aligned}&{\mathcal {D}}_k \ge \frac{1}{2}|k|^2 \ge \frac{1}{8} K_H^2 \ell ^{-2}, \quad \text {for }|k| \ge \frac{1}{2} K_H \ell ^{-1}, \end{aligned}$$

9.50 $$\begin{aligned}&\bigg \vert \rho _z \widehat{(\omega W_1)}(0) -\frac{1}{(2\pi )^2}\int _{ {\mathcal {P}}_H} {\widehat{W}}_1(k) \alpha _k \textrm{d}k \bigg \vert \le C \rho _z \delta ^2 \vert \log \delta \vert , \end{aligned}$$

9.51 $$\begin{aligned}&\bigg \vert \widehat{(\omega W_1)}(0) -\frac{1}{(2\pi )^2}\int _{ {\mathcal {P}}_H} \frac{{\widehat{W}}_1(k)^2}{2 {\mathcal {D}}_k} \textrm{d}k \bigg \vert \le C \delta ^2 \vert \log \delta \vert , \end{aligned}$$

and

9.52 $$\begin{aligned}{} & {} \rho _z\frac{\ell ^2}{(2\pi )^2}\int _{{\mathbb {R}}^2} \widehat{(W_1\omega )}(k) a^{\dagger }_k a_k \textrm{d}k \nonumber \\{} & {} \quad \ge \rho _z\widehat{(W_1\omega )}(0) \frac{\ell ^2}{(2\pi )^2} \int _{{\mathbb {R}}^2} a^{\dagger }_k a_k \textrm{d}k -4 \rho _{z} \delta n_+^H - C \rho _z \delta d^{-2} \frac{R}{\ell }n_+. \end{aligned}$$

#### Proof

The first two inequalities are straightforward from the definitions of the terms. For the third one we split the difference in the following way,

9.53 $$\begin{aligned}&\bigg \vert \rho _z \widehat{(\omega W_1)}(0) -\frac{1}{(2\pi )^2}\int _{k \in {\mathcal {P}}_H} {\widehat{W}}_1(k) \alpha _k \textrm{d}k\bigg \vert \nonumber \\&\le C \bigg \vert \rho _z\int _{k \notin {\mathcal {P}}_H} \frac{{\widehat{W}}_1(k) {\widehat{g}}_k - {\widehat{W}}_1(0){\widehat{g}}_0 \mathbb {1}_{\{|k| \le \ell _{\delta }^{-1}\}}}{2 k^2}\textrm{d}k\bigg \vert + C \left| \int _{k \in {\mathcal {P}}_H} {\widehat{W}}_1(k) \Big ( \alpha _k - \rho _z \frac{{\widehat{g}}_k}{2 k^2} \Big )dk \right| \nonumber \\&=: (I) + (II). \end{aligned}$$

For the first integral we do a further splitting of the domain of integration, considering $(I) \le (I,<) + (I,>)$ for $|k| \le \ell _{\delta }^{-1}$ or otherwise, respectively. For $(I,<)$ we consider a Taylor expansion of the numerator and we get, recalling the symmetry of *g* which in the integration drops the first order,

9.54 $$\begin{aligned} (I,<) \le C \rho _z R^2 \delta ^2 \int _{\{|k| \le \ell _{\delta }^{-1}\}} \le C\rho _z R^2 \delta ^2\ell ^{-2}_{\delta }. \end{aligned}$$

For the $(I,>)$ we proceed by a direct calculation and obtain

9.55 $$\begin{aligned} (I,>) \le C \rho _z \delta ^2 \log (K_H \ell ^{-1}\ell _{\delta }). \end{aligned}$$

Let us analyze the second integral. We have that $|{\mathcal {B}}_k / {\mathcal {A}}_k| \le 1/2$ and therefore we can expand in the following way

9.56 $$\begin{aligned} {\widehat{W}}_1(k) \alpha _k = \rho _z^{-1} {\mathcal {A}}_k \bigg ( 1- \sqrt{1-\frac{{\mathcal {B}}_k^2}{{\mathcal {A}}_k^2}}\bigg ) \simeq \rho _z\frac{{\widehat{W}}_1(k)^2}{2 {\mathcal {A}}_k} + C \rho _z^3 \frac{{\widehat{W}}_1(k)^4}{{\mathcal {A}}_k^3}. \end{aligned}$$

We deduce

$$\begin{aligned} (II)&\le C\bigg \vert \int _{k \in {\mathcal {P}}_H} \Big ({\widehat{W}}_1(k) \alpha _k - \rho _z \frac{{\widehat{W}}_1(k)^2}{2 {\mathcal {A}}_k}\Big ) \textrm{d}k \bigg \vert + C\rho _z\bigg \vert \int _{k \in {\mathcal {P}}_H} {\widehat{W}}_1(k)\bigg ( \frac{{\widehat{W}}_1(k)}{2 {\mathcal {A}}_k} - \frac{{\widehat{g}}_k}{2 k^2} \bigg ) \textrm{d}k \bigg \vert \\&\le C \rho _z^3\int _{k \in {\mathcal {P}}_H} \frac{{\widehat{W}}_1(k)^4}{{\mathcal {A}}_k^3} \textrm{d}k+ C\rho _z\bigg \vert \int _{k \in {\mathcal {P}}_H} {\widehat{W}}_1(k)\bigg ( \frac{{\widehat{W}}_1(k)}{2 {\mathcal {A}}_k} - \frac{{\widehat{g}}_k}{2 k^2} \bigg ) \textrm{d}k \bigg \vert \\&\le C \rho _z^3 \ell ^{4}\delta ^4 K_H^{-4} + C\rho _z \bigg \vert \int _{k \in {\mathcal {P}}_H} {\widehat{W}}_1(k)^2\bigg ( \frac{1}{2 {\mathcal {A}}_k} -\frac{1}{|k|^2} \bigg ) \textrm{d}k\bigg \vert \\ {}&\quad + C \rho _z \bigg \vert \int _{k \in {\mathcal {P}}_H} \bigg ( {\widehat{W}}_1(k)\frac{{\widehat{W}}_1(k)- {\widehat{g}}_k}{2 k^2} \bigg )\textrm{d}k \bigg \vert , \end{aligned}$$

where we used that ${\mathcal {A}}_k \ge \frac{1}{2}|k|^2$ for $k \in {\mathcal {P}}_H$. For the remaining terms, we use that in ${\mathcal {P}}_H$ we have $0 < k^2 - \tau _k \le 2 |k| (d s\ell )^{-1}$,

$$\begin{aligned} C\rho _z \bigg \vert \int _{k \in {\mathcal {P}}_H} \frac{{\widehat{W}}_1(k)^2}{k^2}\bigg ( \frac{k^2 -{\mathcal {A}}_k }{ {\mathcal {A}}_k} \bigg ) \textrm{d}k \bigg \vert&\le C \rho _z \int _{k \in {\mathcal {P}}_H} \frac{{\widehat{W}}_1(k)^2}{ k^2} \bigg ( \frac{2|k|(ds\ell )^{-1}}{k^2} + \rho _z \frac{{\widehat{W}}_1(k)}{k^2}\bigg )\\&\le C \rho _z \delta ^2 (ds)^{-1} K_H^{-1} + C\rho _z^2 \ell ^2 \delta ^3 K_H^{-2}. \end{aligned}$$

By Cauchy–Schwarz inequality we get for the last term

$$\begin{aligned}{} & {} \rho _z \bigg \vert \int _{k \in {\mathcal {P}}_H} \bigg ( {\widehat{W}}_1(k)\frac{{\widehat{W}}_1(k)- {\widehat{g}}_k}{2 k^2} \bigg )\textrm{d}k \bigg \vert \\{} & {} \quad \le C \rho _z \delta \int _{k \in {\mathcal {P}}_H} \frac{{\widehat{W}}_1(k)^2}{2k^2} \textrm{d}k + C \rho _z \delta ^{-1} \int _{k \in {\mathcal {P}}_H} \frac{({\widehat{W}}_1(k) - {\widehat{g}}_k)^2}{2k^2}\textrm{d}k . \end{aligned}$$

We complete the domain of the integrals: by Lemma 6.4 we get

$$\begin{aligned} \rho _z \delta \int _{k \in {\mathcal {P}}_H} \frac{{\widehat{W}}_1(k)^2}{2k^2} \textrm{d}k&\le C \rho _z \delta \widehat{g \omega }(0) + C \rho _z \delta \int _{k \notin {\mathcal {P}}_H} \frac{{\widehat{W}}_1(k)^2 - {\widehat{W}}_1(0)^2 \mathbb {1}_{\{|k|\le \ell _{\delta }^{-1}\}}}{2k^2} \textrm{d}k \\&\le C \rho _z\delta ^2 + C \rho _z \delta ^3 (R^2 \ell ^{-2}_{\delta } + \log (K_H \ell ^{-1}\ell _{\delta })), \end{aligned}$$

and

$$\begin{aligned}&\rho _z \delta ^{-1} \int _{k \in {\mathcal {P}}_H} \frac{({\widehat{W}}_1(k)- {\widehat{g}}_k)^2}{2k^2} \textrm{d}k \\&\le C\rho _z \delta ^{-1} \frac{R^4}{\ell ^4} \widehat{g\omega }(0) + C \rho _z \delta ^{-1} \bigg \vert \int _{k \notin {\mathcal {P}}_H} \frac{({\widehat{W}}_1(k)-{\widehat{g}}_k )^2 - ({\widehat{W}}_1(0) -{\widehat{g}}_0 )^2 \mathbb {1}_{\{|k|\le \ell _{\delta }^{-1}\}}}{2k^2} \textrm{d}k \bigg \vert \\&\le C\rho _z\frac{R^4}{\ell ^4} + C \rho _z \delta \Big (R^2 \ell ^{-2}_{\delta } + \frac{R^2}{\ell ^2}\log (K_H \ell ^{-1}\ell _{\delta })\Big ). \end{aligned}$$

We conclude the proof of (9.50) by collecting all the previous estimates and exploiting the relations between the parameters so that $\rho _z \delta ^2 \vert \log \delta \vert$ is the dominant term.

For the inequality (9.51), we can derive it from (9.50) and the control on the first term of (*II*) above using that, for $k \in {\mathcal {P}}_H$, $\left| 1- \frac{{\mathcal {A}}_k}{{\mathcal {D}}_k} \right| \le \frac{{\mathcal {B}}_k^2}{{\mathcal {A}}_k} \le C \rho _{z}^2 \delta ^2 |k|^{-4}$.

For the last inequality, we estimate the difference, splitting the integral for $|k|\le \xi \ell ^{-1}$ or otherwise,

$$\begin{aligned}{} & {} \rho _z\frac{\ell ^2}{(2\pi )^2}\int _{{\mathbb {R}}^2} (\widehat{(W_1\omega )}(k) -\widehat{(W_1\omega )}(0))a^{\dagger }_k a_k \textrm{d}k \\{} & {} \quad \ge -C \rho _z \xi ^2\delta \frac{R^2}{\ell ^2} n_+ - \frac{2\ell ^2}{(2\pi )^2} \rho _z \delta \int _{{\mathbb {R}}^2} a^{\dagger }_k \mathbb {1}_{\{|k| \ge \xi \ell ^{-1}\}} a_k \textrm{d}k \end{aligned}$$

where we used a Taylor expansion and estimated the integral for $|k| \le \xi \ell ^{-1}$. For the second term we exploit the second quantization in a *N*-bosons sector and we insert symmetrically the sum of projectors $1 = \mathbb {1}_{\{\sqrt{-\Delta } \in {\mathcal {P}}_L\}} + \mathbb {1}_{\{\sqrt{-\Delta } \in {\mathcal {P}}_L^c\}}$

$$\begin{aligned} \frac{\ell ^2}{(2\pi )^2}\int _{{\mathbb {R}}^2} a^{\dagger }_k \mathbb {1}_{\{|k| \ge \xi \ell ^{-1}\}} a_k \textrm{d}k\Big |_N&= \sum _{j=1}^N Q_j \chi _{\Lambda }(x_j) \mathbb {1}_{\{\sqrt{-\Delta _j} \ge \xi \ell ^{-1}\}} \chi _{\Lambda }(x_j) Q_j \\&\ge 2 n_+^H + 2 {\mathcal {N}} n_+ \end{aligned}$$

where we estimated by a Cauchy–Schwarz the cross terms $({\mathcal {P}}_L, {\mathcal {P}}_L^c)$ to make them comparable to the diagonal terms and denoted by

9.57 $$\begin{aligned} {\mathcal {N}} := \Vert \mathbb {1}_{\{\sqrt{-\Delta } \in {\mathcal {P}}_L\}} \chi _{\Lambda } (x) \mathbb {1}_{\{\sqrt{-\Delta } \ge \xi \ell ^{-1}\}}\Vert ^2 \le C \xi ^{-2} d^{-4}. \end{aligned}$$

Here we used the regularity properties of $\chi _{\Lambda }$ dividing and multiplying by $-\Delta$. We conclude optimizing $\xi$ by the choice $\xi ^2 = d^{-2} \frac{\ell }{R }$. $\square$

#### Estimates on ${\mathcal {Q}}_3^{(1)}$

The first part ${\mathcal {Q}}_3^{(1)}$ will absorb ${\mathcal {Q}}_2^{\text {ex}}$ using ${\mathcal {K}}_H^{\text {Diag}}$.

##### Lemma 9.7

(Estimates on ${\mathcal {Q}}_3^{(1)}$). For any state $\Phi$ satisfying (8.44) we have

$$\begin{aligned}{} & {} \left\langle {\mathcal {Q}}_3^{(1)} + {\mathcal {Q}}_2^{ex} + \big (1- 2 \varepsilon _K \big ) {\mathcal {K}}^{\text {Diag}}_H + \frac{b}{100} \frac{n_+}{\ell ^2} + \frac{b}{100}\frac{\varepsilon _T n_+^{H}}{(d\ell )^{2}} \right\rangle _{\Phi } \\{} & {} \quad \ge - C \rho _z^2 \ell ^2 \delta ^2 \Big ( \delta K_H^{-8} K_{\ell }^{10} d^{-4} + \varepsilon _K^{-1} K_H^{-12} K_\ell ^{10} d^{-8} + d^{-8}K_{\ell }^2 K_H^{-4} \Big ). \end{aligned}$$

##### Proof

We first reorder the creation an annihilation operators, applying a change of variables $k \mapsto -k, p \mapsto -p$ in the $\alpha$ terms,

$$\begin{aligned} {\mathcal {Q}}_3^{(1)}&= \frac{z \ell ^2}{(2\pi )^4} \int _{{\mathcal {P}}_H \times {\mathbb {R}}^2} \frac{f_L(p) {{\widehat{W}}}_1(k)}{\sqrt{1-\alpha _k^2}\sqrt{1-\alpha _{p-k}^2}} \\&\quad \times \big ( {\widetilde{a}}_p^\dagger b_{p-k} b_k + \alpha _k \alpha _{p-k} {\widetilde{a}}_{-p}^\dagger b_{p-k}^\dagger b_{k}^\dagger + b_k^\dagger b_{p-k}^\dagger {\widetilde{a}} _p + \alpha _k \alpha _{p-k} b_k b_{p-k} \widetilde{a}_{-p} \big ) \textrm{d}k \textrm{d}p \\&= \frac{z \ell ^2}{(2\pi )^4} \int _{{\mathcal {P}}_H \times {\mathbb {R}}^2} \frac{f_L(p) {{\widehat{W}}}_1(k)}{\sqrt{1-\alpha _k^2}\sqrt{1-\alpha _{p-k}^2}} \Big ( \big ( {\widetilde{a}}_p^\dagger b_{p-k} + \alpha _k \alpha _{p-k} b_{p-k} {\widetilde{a}}_{-p} \big ) b_k \\&\quad + b_k^\dagger \big ( b_{p-k}^\dagger {\widetilde{a}}_p + \alpha _k \alpha _{p-k} {\widetilde{a}}^\dagger _{-p} b_{p-k}^\dagger \big ) \\&\quad + \alpha _k \alpha _{p-k} \big ( \left[ b_k , b_{p-k} {\widetilde{a}}_{-p} \right] + \big [ {\widetilde{a}} _{-p}^\dagger b_{p-k}^\dagger , b_k^\dagger \big ] \big ) \Big ) \textrm{d}k \textrm{d}p . \end{aligned}$$

We can complete the square to get, for $\varepsilon _K \ll 1$ fixed in “Appendix H”,

9.58 $$\begin{aligned} {\mathcal {Q}}_3^{(1)} + (1- 3 \varepsilon _K) {\mathcal {K}}^{\text {Diag}}_H= & {} (1- 3 \varepsilon _K) \frac{\ell ^2}{(2\pi )^2} \int _{{\mathcal {P}}_H} {\mathcal {D}}_k {\tilde{b}}_k^\dagger {\tilde{b}}_k \textrm{d}k \nonumber \\{} & {} + \frac{\ell ^2}{(2\pi )^2} \int _{{\mathcal {P}}_H} \big ( {\mathcal {T}}_1(k) + {\mathcal {T}}_2(k) \big )\textrm{d}k, \end{aligned}$$

where we mantained a small portion of ${\mathcal {K}}^{\text {Diag}}_H$ in order to bound other error terms and we defined

9.59 $$\begin{aligned} {\tilde{b}}_k :=&\, b_k + \frac{z}{{\mathcal {D}}_k (1- 3\varepsilon _K) (2\pi )^2} \nonumber \\&\int \frac{f_L(p) {{\widehat{W}}}_1(k)}{\sqrt{1-\alpha _k^2}\sqrt{1-\alpha _{p-k}^2}} \Big ( b_{p-k}^\dagger {\widetilde{a}}_p + \alpha _k \alpha _{p-k} {\widetilde{a}}_{-p}^\dagger b_{p-k}^\dagger \Big ) \textrm{d}p, \end{aligned}$$

9.60 $$\begin{aligned} {\mathcal {T}}_1(k) :=&\frac{z }{(2\pi )^2} \int _{ {\mathbb {R}}^2} \frac{f_L(p) {{\widehat{W}}}_1(k)}{\sqrt{1-\alpha _k^2}\sqrt{1-\alpha _{p-k}^2}} \alpha _k \alpha _{p-k} \big ( \big [ b_k^\dagger , {\widetilde{a}}_{-p}^\dagger b_{p-k}^\dagger \big ] + h.c. \big ) \textrm{d}p, \end{aligned}$$

9.61 $$\begin{aligned} {\mathcal {T}}_2 (k) :=&- \frac{\vert z \vert ^2 {{\widehat{W}}}_1(k)^2}{(1-3\varepsilon _K) {\mathcal {D}}_k (1-\alpha _k^2)(2\pi )^4} \int \frac{f_L(p) f_L(s)}{\sqrt{1-\alpha _{s-k}^2} \sqrt{1- \alpha _{p-k}^2}} \nonumber \\ {}&\times \big ( {\widetilde{a}}_{p}^\dagger b_{p-k} + \alpha _k \alpha _{p-k} b_{p-k} {\widetilde{a}}_{-p} \big ) \big ( b_{s-k}^\dagger \widetilde{a}_s + \alpha _k \alpha _{s-k} \widetilde{a}_{-s}^\dagger b_{s-k}^\dagger \big ) \textrm{d}p \textrm{d}s. \end{aligned}$$

$\bullet$ Let us estimate the error term ${\mathcal {T}}_1(k)$. We use $\big [ b_k^\dagger , {\widetilde{a}}_{-p}^\dagger b_{p-k}^\dagger \big ] = \widetilde{a}_{-p}^\dagger \big [ b_k^\dagger , b_{p-k}^\dagger \big ] + \big [ b_k^\dagger , {\widetilde{a}}_{-p}^\dagger \big ] b_{p-k}^\dagger$ and the Cauchy–Schwarz inequality with weights $\varepsilon _1$, $\varepsilon _2 >0$,

$$\begin{aligned} {\mathcal {T}}_1(k)&\ge - C z \int _{ {\mathbb {R}}^2} \frac{f_L(p) {{\widehat{W}}}_1(k)}{\sqrt{1-\alpha _k^2}\sqrt{1-\alpha _{p-k}^2}} \vert \alpha _k \alpha _{p-k} \vert \\&\quad \Big ( \big ( \varepsilon _1 {\widetilde{a}}_{-p}^\dagger {\widetilde{a}}_{-p} + \varepsilon _1^{-1} \big ) \vert [ b_k^\dagger , b_{p-k}^\dagger ] \vert + \vert [b_k^\dagger , \widetilde{a}_{-p}^\dagger ] \vert \big ( \varepsilon _2 b_{p-k}^\dagger b_{p-k} + \varepsilon _2^{-1} \big ) \Big )\textrm{d}p. \end{aligned}$$

By (9.33) and (9.31) we have $\vert [ b_k^{\dagger }, \widetilde{a}_{-p}^\dagger ] \vert \le C \vert \alpha _k \vert$ and $\vert [ b_k^{\dagger } , b_{p-k}^{\dagger } ] \vert \le C \vert \alpha _k \vert$. Therefore using (9.48),

$$\begin{aligned}{} & {} \frac{\ell ^2}{(2\pi )^2} \int _{{\mathcal {P}}_H} {\mathcal {T}}_1(k) \textrm{d}k \ge -C \frac{z \ell ^2}{(2\pi )^4} \int _{{\mathcal {P}}_H \times {\mathbb {R}}^2} \frac{\vert f_L(p) \vert \rho _z^3 \delta ^4 }{k^6} \\{} & {} \quad \times \Big ( (\varepsilon _1 {\widetilde{a}}_{-p}^\dagger \widetilde{a}_{-p} + \varepsilon ^{-1}_1) + ( \varepsilon _2 b_{p-k}^\dagger b_{p-k} + \varepsilon _2^{-1} ) \Big ) \textrm{d}k \textrm{d}p . \end{aligned}$$

Due to the presence of the cutoff $f_L$ on low momenta and the bounds

9.62 $$\begin{aligned} \int _{{\mathcal {P}}_L} ( \varepsilon _1 {\widetilde{a}}_{-p}^\dagger {\widetilde{a}}_{-p} + \varepsilon _1^{-1} ) \textrm{d}p&\le C \frac{\varepsilon _1 n_+}{\ell ^2} + \varepsilon _1^{-1} \frac{d^{-4}}{\ell ^2}, \end{aligned}$$

9.63 $$\begin{aligned} \int _{{\mathcal {P}}_L} ( \varepsilon _2 b_{k}^\dagger b_{k} + \varepsilon _2^{-1})\textrm{d}p&\le C \frac{d^{-4}}{\ell ^2} ( \varepsilon _2 b_k^\dagger b_k + \varepsilon _2^{-1}) , \end{aligned}$$

where we changed the *k* variable, we find,

$$\begin{aligned} \frac{\ell ^2}{(2\pi )^2} \int _{{\mathcal {P}}_H} {\mathcal {T}}_1(k) \textrm{d}k\ge -C z \rho _z^3 \delta ^4 \int _{{\mathcal {P}}_H} \frac{1}{k^6} \big ( (\varepsilon _1 n_+ + \varepsilon _1^{-1} d^{-4}) + d^{-4} ( \varepsilon _2 b_k^\dagger b_k + \varepsilon _2^{-1} ) \big ) \textrm{d}k. \end{aligned}$$

We insert ${\mathcal {D}}_k \ge C^{-1} k^{2}$ in front of $b_k^\dagger b_k$ and get the bound

$$\begin{aligned}&\frac{\ell ^2}{(2\pi )^2} \int _{{\mathcal {P}}_H} {\mathcal {T}}_1(k) \textrm{d}k \\&\ge - C z \rho _z^3 \delta ^4 \ell ^6 K_H^{-4} \varepsilon _1 \frac{n_+}{\ell ^2} - C z \varepsilon _1^{-1} d^{-4} \rho _z^3 \delta ^4 \ell ^4 K_H^{-4} \\&\quad - C \varepsilon _2 \ell ^6 z \rho _z^3 \delta ^4 K_H^{-8} d^{-4} \ell ^2 \int _{{\mathcal {P}}_H} {\mathcal {D}}_k b_k^\dagger b_k \textrm{d}k -C \varepsilon _2^{-1} \ell ^4 z \rho _z^3 \delta ^4 d^{-4} K_H^{-4} . \end{aligned}$$

One can choose $\varepsilon _1$, $\varepsilon _2$ such that the first and third terms are absorbed in the positive $\frac{b}{100} \frac{n_+}{\ell ^2}$ and $\varepsilon _K {\mathcal {K}}_{H}^{\text {Diag}}$ respectively. With this choice the second and fourth terms are errors of respective sizes

$$\begin{aligned} C \ell ^2 \rho _z^2 \delta ^2 (\delta K_{\ell }^{10} K_H^{-8} d^{-4}) \quad \text { and } \quad C \ell ^2 \rho _z^2 \delta ^2 ( \delta K_{\ell }^{10} K_H^{-12} d^{-4}\varepsilon _K^{-1}) .\end{aligned}$$

$\bullet$ Let us now focus on the square term ${\mathcal {T}}_2 (k)$ in (9.61). One can write, in normal order,

$$\begin{aligned} {\widetilde{a}}_p^\dagger b_{p-k} + \alpha _k \alpha _{p-k} b_{p-k} {\widetilde{a}}_{-p}&= {\widetilde{a}}_p^\dagger b_{p-k} + \alpha _k \alpha _{p-k} {\widetilde{a}}_{-p} b_{p-k} + \alpha _k \alpha _{p-k} [ b_{p-k}, {\widetilde{a}}_{-p} ] , \end{aligned}$$

and use the Cauchy–Schwarz inequality with weight $\varepsilon _K$ on the cross terms to find

9.64 $$\begin{aligned} {\mathcal {T}}_2(k)&\ge (1+ \varepsilon _K) {\mathcal {T}}_2'(k) + (1+\varepsilon ^{-1}_K) {\mathcal {T}}_2''(k), \end{aligned}$$

with

$$\begin{aligned} {\mathcal {T}}_2'(k)&= - \frac{\vert z \vert ^2 {{\widehat{W}}}_1(k)^2 }{(1-3\varepsilon _K) {\mathcal {D}}_k (1-\alpha _k^2) (2\pi )^4} \int \frac{f_L(p) f_L(s)}{\sqrt{1-\alpha _{p-k}^2}\sqrt{1-\alpha _{s-k}^2}} \\&\qquad \times ( {\widetilde{a}}_p^\dagger + \alpha _k \alpha _{p-k} {\widetilde{a}}_{-p}) b_{p-k} b_{s-k}^\dagger ({\widetilde{a}}_s + \alpha _k \alpha _{s-k} {\widetilde{a}} _{-s}^\dagger ) \textrm{d}p \textrm{d}s,\\ {\mathcal {T}}_2''(k)&= - \frac{\vert z \vert ^2 {{\widehat{W}}}_1(k)^2}{(1-3\varepsilon _K) {\mathcal {D}}_k (1-\alpha _k^2) (2\pi )^4} \int \frac{f_L(p) f_L(s)}{\sqrt{1-\alpha _{s-k}^2} \sqrt{1-\alpha _{p-k}^2}} \\&\qquad \times \alpha _k^2 \alpha _{p-k} \alpha _{s-k} \vert [ b_{p-k}, {\widetilde{a}}_{-p}] \vert \vert [ {\widetilde{a}}_{-s}^\dagger , b_{s-k}^\dagger ] \vert \textrm{d}p \textrm{d}s . \end{aligned}$$

${\mathcal {T}}_2''$ we can estimate (for $k \in {\mathcal {P}}_H$),

$$\begin{aligned} \frac{\ell ^2}{(2\pi )^2}\int _{{\mathcal {P}}_H} {\mathcal {T}}_2 '' (k) \textrm{d}k&\ge - C \rho _z \ell ^4 \Big ( \int _{{\mathcal {P}}_H} \frac{\widehat{W}_1(k)^2}{(1-3\varepsilon _K) {\mathcal {D}}_k} \vert \alpha _k \vert ^4 \textrm{d}k \Big ) d^{-8} \ell ^{-4} \sup \vert [ b_{p-k} , \widetilde{a}_{-s} ] \vert ^2 , \end{aligned}$$

and by (9.51), (9.33) and (9.48) we get

9.65 $$\begin{aligned} (1+ \varepsilon _K^{-1}) \frac{\ell ^2}{(2\pi )^2} \int _{{\mathcal {P}}_H} {\mathcal {T}}_2''(k) \textrm{d}k \ge -C \rho _z^2 \ell ^2\delta ^2( \varepsilon _K^{-1} K_H^{-12} K_{\ell }^{10} d^{-8}). \end{aligned}$$

Now we use a commutator to write ${\mathcal {T}}_{2}' = {\mathcal {T}}_{2,\text {op}}' + {\mathcal {T}}_{2,\text {com}}'$ in normal order for the $b_k$, with

9.66 $$\begin{aligned} {\mathcal {T}}_{2,\text {op}}'(k)&= - \frac{\vert z \vert ^2 {{\widehat{W}}}_1(k)^2}{(2\pi )^4 (1-3 \varepsilon _K) {\mathcal {D}}_k (1-\alpha _k^2)} \int \frac{f_L(p) f_L(s)}{\sqrt{1-\alpha _{p-k}^2}\sqrt{1-\alpha _{s-k}^2}} \nonumber \\&\qquad \times ( {\widetilde{a}}_p^\dagger + \alpha _k \alpha _{p-k} {\widetilde{a}} _{-p}) b_{s-k}^\dagger b_{p-k} ( {\widetilde{a}} _{s} + \alpha _k \alpha _{s-k} \widetilde{a}_{-s}^\dagger ) \textrm{d}p \textrm{d}s, \nonumber \\ {\mathcal {T}}_{2,\text {com}}'(k)&= - \frac{\vert z \vert ^2 {{\widehat{W}}}_1(k)^2}{(2\pi )^4 (1-3\varepsilon _K) {\mathcal {D}}_k (1-\alpha _k^2)} \int \frac{f_L(p) f_L(s)}{\sqrt{1-\alpha _{p-k}^2}\sqrt{1-\alpha _{s-k}^2}} \nonumber \\&\qquad \times ( {\widetilde{a}}_p^\dagger + \alpha _k \alpha _{p-k} {\widetilde{a}} _{-p}) [ b_{p-k} , b_{s-k}^\dagger ] ( {\widetilde{a}} _{s} + \alpha _k \alpha _{s-k} \widetilde{a}_{-s}^\dagger ) \textrm{d}p \textrm{d}s . \end{aligned}$$

$\bullet$ In order to estimate the error term ${\mathcal {T}}_{2,\text {op}}'$, we introduce

9.67 $$\begin{aligned} \tau _s := {\widetilde{a}} _{s} + \alpha _k \alpha _{s-k} \widetilde{a}_{-s}^\dagger \quad \text {and} \quad {\mathcal {C}} := \sup _{p,s \in {\mathcal {P}}_L , k \in {\mathcal {P}}_H} \vert [ b_{p-k} , \tau _s ] \vert . \end{aligned}$$

In ${\mathcal {T}}_{2,\text {op}}'$ we commute the *b*’s trough the *a*’s,

$$\begin{aligned} \tau _p^\dagger b_{s-k}^\dagger b_{p-k} \tau _s&= b_{s-k}^\dagger \tau _p^\dagger \tau _s b_{p-k} + [\tau _p^\dagger , b_{s-k}^\dagger ] \tau _s b_{p-k} \\&\quad + b_{s-k}^\dagger \tau _p^\dagger [b_{p-k}, \tau _s] + [\tau _p^\dagger , b_{s-k}^\dagger ] [b_{p-k}, \tau _s], \end{aligned}$$

and use the Cauchy–Schwarz inequality

$$\begin{aligned} \tau _p^\dagger b_{s-k}^\dagger b_{p-k} \tau _s \le C( b_{s-k}^\dagger \tau _p^\dagger \tau _p b_{s-k} + b_{p-k}^\dagger \tau ^\dagger _s \tau _s b_{p-k} + {\mathcal {C}}^2 ) . \end{aligned}$$

Inserting it in ${\mathcal {T}}_{2,\text {op}}'$, bounding $(1-3 \varepsilon _K)(1-\alpha _k) \ge 1/2$ and noticing that we can exchange *s* and *p* in the integral, we find

$$\begin{aligned} {\mathcal {T}}_{2,\text {op}}'(k) \ge -C \frac{\vert z \vert ^2 {{\widehat{W}}}_1(k)^2}{{\mathcal {D}}_k } \int \frac{f_L(p) f_L(s)}{\sqrt{1-\alpha _{p-k}^2}\sqrt{1-\alpha _{s-k}^2}} (b_{s-k}^\dagger \tau _p^\dagger \tau _p b_{s-k} + {\mathcal {C}}^2) \textrm{d}p \textrm{d}s. \end{aligned}$$

When we apply this operator to the state $\Phi$ which satisfies $\mathbb {1}_{[0,{\mathcal {M}}]}(n_+^L) \Phi = \Phi$ we can apply Lemma 8.1 for the vector $b_{s-k} \Phi$ to get the estimate

$$\begin{aligned} \langle {\mathcal {T}}_{2,\text {op}}'(k) \rangle _{\Phi } \ge - C \frac{ \vert z \vert ^2 {{\widehat{W}}}_1(k)^2}{{\mathcal {D}}_k} \Big ( \ell ^{-2} {\mathcal {M}} \int f_L(s) \langle b_{s-k}^\dagger b_{s-k} \rangle _\Phi \textrm{d}s+ d^{-8} \ell ^{-4} {\mathcal {C}}^2 \Big ) , \end{aligned}$$

and finally, using again (9.51) and (9.49), and the fact that ${\mathcal {C}} \le C K_{\ell }^2 K_H^{-2}$ by (9.33) and Lemma F.1,

9.68 $$\begin{aligned} \frac{\ell ^2}{(2\pi )^2} \int _{{\mathcal {P}}_H} \langle {\mathcal {T}}_{2,\text {op}}'(k) \rangle _{\Phi } \textrm{d}k&\ge - C \rho _z \ell ^2 \delta ^2 K_H^{-4} d^{-4} {\mathcal {M}} \langle {\mathcal {K}}_H^{\text {Diag}} \rangle _{\Phi } - C \rho _z \delta d^{-8} K_{\ell }^4 K_H^{-4} \nonumber \\&\ge -C K_{\ell }^4 K_H^{-4} d^{-4} \varepsilon _{{\mathcal {M}}} {\mathcal {K}}_H^{\text {Diag}} - C \rho _z^2\ell ^2\delta ^2 d^{-8}K_{\ell }^2 K_H^{-4}. \end{aligned}$$

The first part can be absorbed in the positive $\varepsilon _K {\mathcal {K}}_H^{\text {Diag}}$, as long as the relation (H21) holds, and the second part contributes to the error term.

$\bullet$ We now turn to ${\mathcal {T}}_{2,\text {com}}'$ given in (9.66). This term will absorb ${\mathcal {Q}}_2^{ex}$. We first use Lemma F.1, (9.32) and (9.48) to estimate the commutator,

$$\begin{aligned} \left| [\right. \left. b_{p-k}, b_{s-k}^\dagger ]- \widehat{\chi ^2}((p-s)\ell ) \right|&= \left| \alpha _{p-k} \alpha _{s-k} \widehat{\chi ^2}((p-s)\ell )\right| \\ {}&\quad + \left| (1- \alpha _{p-k} \alpha _{s-k}) {\widehat{\chi }} ((p-k)\ell ) {\widehat{\chi }} ((s-k) \ell ) \right| \\&\le C K_{\ell }^{4}K_H^{-4}, \end{aligned}$$

and bounding then by a Cauchy–Schwarz inequality

$$\begin{aligned}{} & {} ( {\widetilde{a}}_p^\dagger + \alpha _k \alpha _{p-k} {\widetilde{a}} _{-p}) (\widehat{(\chi ^2)}((p-s)\ell ) + C K_{\ell }^{4} K_{H}^{-4}) ( {\widetilde{a}} _{s} + \alpha _k \alpha _{s-k} \widetilde{a}_{-s}^\dagger ) \\{} & {} \quad \le {\widetilde{a}}_p^\dagger \widehat{\chi ^2}((p-s)\ell ) \widetilde{a}_s + C ({\widetilde{a}}_{-p}^\dagger {\widetilde{a}}_{-p} + \widetilde{a}_s^\dagger {\widetilde{a}}_s ) K_\ell ^4 K_H^{-4}. \end{aligned}$$

We get, by using Lemma 8.1

$$\begin{aligned} \frac{\ell ^2}{(2\pi )^2} \int _{{\mathcal {P}}_H} {\mathcal {T}}_{2,\text {com}}'(k) \textrm{d}k&\ge - \frac{\ell ^2}{(2\pi )^2} \int _{{\mathcal {P}}_H} \frac{\vert z \vert ^2 {{\widehat{W}}}_1(k)^2}{(2\pi )^4 (1-3\varepsilon _K) {\mathcal {D}}_k (1-\alpha _k^2)} \\&\quad \times \int \frac{f_L(p) f_L(s)}{\sqrt{1-\alpha _{p-k}^2}\sqrt{1-\alpha _{s-k}^2}} {\widetilde{a}}_p^{\dagger } \widehat{\chi ^2}((p-s)\ell ) {\widetilde{a}}_s \textrm{d}p \textrm{d}s \textrm{d}k \\&\quad - C \Big ( \int _{{\mathcal {P}}_H} \frac{|z|^2 \widehat{W}_1(k)^2}{{\mathcal {D}}_k} \textrm{d}k \Big ) d^{-4} K_\ell ^4 K_H^{-4} \frac{n_+}{\ell ^2}. \end{aligned}$$

Using (9.49) the last part is of order $K_{\ell }^6 K_H^{-4} d^{-4} \frac{n_+}{\ell ^2}$ and can be absorbed in a fraction of the positive $\frac{b}{100} \frac{n_+}{\ell ^2}$ by (H8). For the first term we use the following formula valid in a Fock sector with *N* bosons

9.69 $$\begin{aligned} \frac{\ell ^4}{(2\pi )^4} \int f_L(p) f_L(s) \widetilde{a}_p^{\dagger } \widehat{\chi ^2}((p-s)\ell ) a_s \textrm{d}s \textrm{d}p|_N = \sum _{j=1}^N Q_{L,j}^{\dagger } \chi _\Lambda ^2(x_j) Q_{L,j}, \end{aligned}$$

to rewrite, due to (H8) and by (9.49),

$$\begin{aligned} \frac{\ell ^2}{(2\pi )^2}&\int _{{\mathcal {P}}_H} {\mathcal {T}}_{2,\text {com}}'(k) \textrm{d}k\\&\ge - \frac{(1+C\varepsilon _K )}{(2\pi )^{2}} \int \frac{\rho _z {{\widehat{W}}}_1(k)^{2}}{{\mathcal {D}}_k (1-\alpha _k^2)} \textrm{d}k \sum _{j=1}^N Q_{L,j}^{\dagger } \chi _\Lambda ^2(x_j) Q_{L,j} -\frac{b}{200}\frac{n_+}{\ell ^2} \\&\ge - (1+ C \varepsilon _K + C K_\ell ^4 K_H^{-4}) \\ {}&\quad \times \Big ( 2 \rho _z \widehat{(\omega W_1)}(0) + C \rho _z \delta ^2 \vert \log \delta \vert \Big ) \sum _{j=1}^N Q_{L,j}^{\dagger } \chi _\Lambda ^2 Q_{L,j} -\frac{b}{200}\frac{n_+}{\ell ^2}. \end{aligned}$$

In this last expression we want to replace $Q_{L,j}$ by $Q_j$. Using Cauchy–Schwarz with weight $\varepsilon _0$ we find

9.70 $$\begin{aligned} Q_{L,j}^{\dagger } \chi _\Lambda ^2 Q_{L,j} \le (1+\varepsilon _0) Q_j \chi _\Lambda ^2 Q_j + (1+\varepsilon _0^{-1}) Q_j (f_L - 1) \chi _j^2 (f_L - 1) Q_j, \end{aligned}$$

and since $f_L$ localizes on low momenta we can bound the second term by $n_+^H$, and the term $\varepsilon _0 Q_j \chi _j^2 Q_j$ by $C \varepsilon _0 n_+$,

$$\begin{aligned} \frac{\ell ^2}{(2\pi )^2} \int _{{\mathcal {P}}_H} {\mathcal {T}}_{2,\text {com}}'(k)\textrm{d}k&\ge - 2 \rho _z \widehat{(\omega W_1)}(0) \sum _{j=1}^N \big ( Q_j \chi _j^2 Q_j + C \varepsilon ^{-1}_0 n_+^H + C \varepsilon _0 n_+ \big ) \\&\quad - C \Big (\rho _z \ell ^2\delta ^2 \vert \log \delta \vert + \rho _z \ell ^2\delta \varepsilon _K + \rho _z \ell ^2 \delta K_\ell ^4 K_H^{-4} + \frac{b}{200}\Big ) \frac{n_+}{\ell ^2}. \end{aligned}$$

The $n_+^H$-part can be absorbed by the positive $\frac{b}{100} \frac{\varepsilon _T n_+^H}{(d \ell )^2}$ if we choose $\varepsilon _0 \simeq \frac{\rho _z \delta d^2 \ell ^2}{\varepsilon _T} \simeq \frac{d^2 K_\ell ^2}{\varepsilon _T}$. With this choice the $n_+$ terms are of order

9.71 $$\begin{aligned} \Big ( \frac{d^2 K_\ell ^4}{\varepsilon _T} + \delta K_\ell ^2 \vert \log \delta \vert + K_\ell ^2 \varepsilon _K + K_\ell ^6 K_H^{-4} + \frac{b}{200} \Big ) \frac{n_+}{\ell ^2} . \end{aligned}$$

Those terms are absorbed in a fraction of the positive $\frac{b}{100} \frac{n_+}{\ell ^2}$, as long as we have the relations (H8), (H10), (H17) and (H20). We deduce

$$\begin{aligned} \frac{\ell ^2}{(2\pi )^2} \int _{{\mathcal {P}}_H} {\mathcal {T}}_{2,\text {com}}'(k)\textrm{d}k \ge - 2 \rho _z \widehat{(\omega W_1)}(0) \sum _{j=1}^N Q_j \chi _j^2 Q_j - \frac{b}{150} \frac{n_+}{\ell ^2} - \frac{b}{100}\frac{\varepsilon _T n_+^H}{(d\ell )^2}. \end{aligned}$$

To compare the remaining part with ${\mathcal {Q}}_2^{\text {ex}}$ we use (9.52) to find

$$\begin{aligned} {\mathcal {Q}}_2^{\text {ex}}&= \rho _z \frac{\ell ^2}{(2\pi )^2} \int \Big ( \widehat{W\omega }(k) + \widehat{W\omega }(0) \Big ) a_k^\dagger a_k \textrm{d}k \\&\ge 2 \rho _z \frac{\ell ^2}{(2\pi )^2} \widehat{W_1 \omega }(0) \int a_k^\dagger a_k \textrm{d}k - C \rho _z \delta ( d^{-2} R \ell ^{-1} n_+ + C n_+^H)\\&= 2 \rho _z \widehat{W_1 \omega }(0) \sum _j Q_j \chi _\Lambda ^2 Q_j - C \rho _z \delta d^{-2} R \ell ^{-1} n_+ + C \rho _z \delta n_+^H . \end{aligned}$$

Using that $\rho _z \simeq \rho _{\mu }$, the remaining parts are absorbed by the spectral gaps and then we get

$$\begin{aligned} \frac{\ell ^2}{(2\pi )^2} \int _{{\mathcal {P}}_H} {\mathcal {T}}_{2,\text {com}}'(k) \textrm{d}k + {\mathcal {Q}}_2^{\text {ex}} \ge - \frac{b}{100}\frac{n_+}{\ell ^2} - \frac{b}{100} \frac{\varepsilon _T n_+^H}{(d\ell )^2} . \end{aligned}$$

This last estimate, together with (9.58), (9.64), (9.65) and (9.68) concludes the proof. $\square$

##### Remark 9.8

It was necessary to replace $a_k$’s by $b_k$’s before estimating ${\mathcal {Q}}_3(z) + {\mathcal {Q}}_2^{ex}$, otherwise we would need a fraction of the kinetic energy instead of ${\mathcal {K}}_H^{\textrm{diag}}$ in Lemma 9.7, and this we cannot allow (see Remark 9.5). In other words, it is important that the positive term in (9.58) is given in terms of ${\tilde{b}}_k$ (Eq. (9.59)) whose main part is $b_k$.

#### Estimates on ${\mathcal {Q}}_3^{(2)}$ and ${\mathcal {Q}}_3^{(3)}$

##### Lemma 9.9

(Estimates on ${\mathcal {Q}}_3^{(2)}$ and ${\mathcal {Q}}_3^{(3)}$). For any normalized state $\Phi$ satisfying (8.44) we have

$$\begin{aligned} \Big \langle {\mathcal {Q}}_3^{(2)} + {\mathcal {Q}}_3^{(3)} + \frac{\varepsilon _K}{100} {\mathcal {K}}^{\text {Diag}}_H \Big \rangle _\Phi \ge - C \rho _z^2\ell ^2 \delta ^2 \varepsilon _K^{-1} K_H^{-2M-8} K_\ell ^6 d^{-8}. \end{aligned}$$

##### Proof

We focus on ${\mathcal {Q}}_3^{(3)}$ (the estimates on ${\mathcal {Q}}_3^{(2)}$ are similar), and decompose it into ${\mathcal {Q}}_3^{(3)} = I + II$, where

9.72 $$\begin{aligned} I&= - \frac{z \ell ^2}{(2\pi )^4} \int _{{\mathcal {P}}_H \times {\mathbb {R}}^2} \frac{f_L(p) {{\widehat{W}}}_1(k) \alpha _{p-k}}{\sqrt{1-\alpha _k^2} \sqrt{1-\alpha _{p-k}^2}} \big ( b^\dagger _{k-p} \widetilde{a}^\dagger _p b_{k} + b_{k}^\dagger \widetilde{a}_p b_{k-p} \big ) \textrm{d}k \textrm{d}p, \end{aligned}$$

and

9.73 $$\begin{aligned} II&= - \frac{z \ell ^2}{(2\pi )^4} \int _{{\mathcal {P}}_H \times {\mathbb {R}}^2} \frac{f_L(p) {{\widehat{W}}}_1(k) \alpha _{p-k}}{\sqrt{1-\alpha _k^2} \sqrt{1-\alpha _{p-k}^2}} \big ( [{\widetilde{a}}_p^\dagger , b_{k-p}^\dagger ] b_{k} + b_{k}^\dagger [ b_{k-p}, {\widetilde{a}}_p] \big ) \textrm{d}k \textrm{d}p. \end{aligned}$$

The first part we estimate using Cauchy–Schwarz with weight $\varepsilon$, and by (9.48)

$$\begin{aligned} I&\ge - \frac{z \ell ^2}{(2\pi )^4} \int _{{\mathcal {P}}_H \times {\mathbb {R}}^2} \frac{f_L(p) {{\widehat{W}}}_1(k) \alpha _{p-k}}{\sqrt{1-\alpha _k^2} \sqrt{1-\alpha _{p-k}^2}} ( \varepsilon b_{k-p}^\dagger {\widetilde{a}}_p^\dagger {\widetilde{a}}_p b_{k-p} + \varepsilon ^{-1} b_k^\dagger b_k ) \textrm{d}k \textrm{d}p \\&\ge - C z \ell ^2 \delta K_\ell ^2 K_H^{-2} \int _{{\mathcal {P}}_H \times {\mathbb {R}}^2} f_L(p) ( \varepsilon b_{k-p}^{\dagger } \widetilde{a}_p^\dagger {\widetilde{a}}_p b_{k-p} + \varepsilon ^{-1} b_k^\dagger b_k ) \textrm{d}k \textrm{d}p , \end{aligned}$$

and using Lemma 8.1,

9.74 $$\begin{aligned} \langle \Phi , \int f_L(p) b_{k-p}^\dagger {\widetilde{a}}_p^\dagger {\widetilde{a}}_p b_{k-p} \textrm{d}p \Phi \rangle \le C \ell ^{-2} {\mathcal {M}} \langle \Phi , b_{k}^\dagger b_{k} \Phi \rangle . \end{aligned}$$

We choose $\varepsilon = \sqrt{d^{-4}/ {\mathcal {M}}}$, and insert ${\mathcal {D}}_k \ge K_H^2 \ell ^{-2}$,

9.75 $$\begin{aligned} \langle I \rangle _\Phi&\ge - C z \delta K_\ell ^2 K_H^{-2} (\varepsilon {\mathcal {M}} + \varepsilon ^{-1} d^{-4} ) \int _{k \in {\mathcal {P}}_H} \langle b_k^\dagger b_k \rangle _\Phi \textrm{d}k \nonumber \\&\ge - C (\rho _z^{1/2} \ell \delta K_\ell ^2 K_H^{-4} {\mathcal {M}}^{1/2} d^{-2}) \ell ^2 \int _{k \in {\mathcal {P}}_H} {\mathcal {D}}_k \langle b_k^\dagger b_k \rangle _\Phi \textrm{d}k . \end{aligned}$$

Thanks to condition (H21), *I* can be absorbed in the positive $\frac{\varepsilon _K}{100}{\mathcal {K}}^{\text {Diag}}_H$ term. Now we return to the commutator term, which can be estimated using a Cauchy–Schwarz inequality with new weight $\varepsilon$,

$$\begin{aligned} II \ge - 2 \frac{ z \ell ^2}{(2\pi )^4} \int _{{\mathcal {P}}_H \times {\mathbb {R}}^2} \vert [ b_{k-p} , {\widetilde{a}}_p ] \vert f_L(p) \vert \widehat{W}_1(k) \alpha _k \vert ( \varepsilon b_k^\dagger b_k + \varepsilon ^{-1}) \textrm{d}k \textrm{d}p . \end{aligned}$$

We use the commutator bound $\vert [b_{k-p}, {\widetilde{a}}_p] \vert \le C \alpha _{k-p} \sup _{k \in {\mathcal {P}}_H} {{\widehat{\chi }}} (k \ell )$ from (9.33),

$$\begin{aligned} II&\ge -C z K_\ell ^2 K_H^{-2} \Big ( \sup _{k \in {\mathcal {P}}_H} {{\widehat{\chi }}} (k\ell ) \Big ) d^{-4} \Big ( \varepsilon K_\ell ^2 K_H^{-4} \delta \ell ^2 \int _{{\mathcal {P}}_H} {\mathcal {D}}_k b_k^\dagger b_k \textrm{d}k +\varepsilon ^{-1} \int _{{\mathcal {P}}_H} {{\widehat{W}}}_1(k) \alpha _k \textrm{d}k \Big ). \end{aligned}$$

With $\varepsilon ^{-1} \simeq \varepsilon _K^{-1} z d^{-4} K_\ell ^4 K_H^{-6} \delta \big ( \sup {{\widehat{\chi }}} \big )$ and our choice of parameters, the first part is absorbed in the positive $\varepsilon _K {\mathcal {K}}^{\text {Diag}}$ term. We estimate the last part using (9.50) and Lemma F.1, and then *II* contributes with an error of order $\varepsilon _K^{-1} \rho _z^2 \ell ^2 \delta ^2 K_H^{-2M-8} K_\ell ^6 d^{-8}$. $\square$

#### Estimates on ${\mathcal {Q}}_3^{(4)}$

First we rewrite ${\mathcal {Q}}_1^{\text {ex}}$ as a term appearing in ${\mathcal {Q}}_3^{(4)}$.

##### Lemma 9.10

Assume that Assumptions of “Appendix H” are satisfied. Then there exists a universal constant $C>0$ such that

$$\begin{aligned} {\mathcal {Q}}_1^{\text {ex}} + \frac{b}{100} \frac{n_+}{\ell ^2} + \frac{b}{100} \frac{ \varepsilon _T n_+^H}{(d\ell )^2}&\ge \frac{z \ell ^2}{(2\pi )^4}\int _{{\mathcal {P}}_H \times {\mathbb {R}}^2} {{\widehat{W}}}_1(k) \alpha _k \widehat{\chi ^2}(p \ell ) f_L(p) ({\widetilde{a}}_p^{\dagger } + {\widetilde{a}}_p) \textrm{d}k \textrm{d}p \\&\qquad -C\rho ^2_z\ell ^2 \delta ^3 K_\ell ^2 \vert \log \delta \vert ^2 - \rho ^{2}_z \ell ^2 \delta K_\ell ^2 d^{8M-2} \varepsilon _T^{-1}. \end{aligned}$$

##### Proof

First we can rewrite ${\mathcal {Q}}_1^{\text {ex}}$ in terms of the ${\widetilde{a}}_p$’s,

$$\begin{aligned}{\mathcal {Q}}_1^{\text {ex}} = z \rho _z \widehat{ \omega W_1}(0) \frac{\ell ^2}{(2\pi )^2} \int \widehat{\chi ^2}(p \ell ) ( {\widetilde{a}}_p^{\dagger } + {\widetilde{a}}_p ) \textrm{d}p,\end{aligned}$$

and then we use (9.50) to compare $\widehat{\omega W_1}(0)$ with an integral in *k*, and using the bound $K_{\ell }^2 K_H^{-2} \ll 1$,

9.76 $$\begin{aligned} {\mathcal {Q}}_1^{\text {ex}}&\ge \frac{z \ell ^2}{(2\pi )^4} \int _{{\mathcal {P}}_H \times {\mathbb {R}}^2} {\widehat{W}}_1(k) \alpha _k \widehat{\chi ^2}(p \ell ) ({\widetilde{a}}_p^\dagger + {\widetilde{a}}_p) \textrm{d}k \textrm{d}p \nonumber \\&\quad - C \rho _z \delta ^2 \vert \log \delta \vert z \ell ^2 \int _{{\mathbb {R}}^2} \widehat{\chi ^2}(p \ell ) ({\widetilde{a}}_p^\dagger + {\widetilde{a}}_p) \textrm{d}p . \end{aligned}$$

The second integral can be estimated using a Cauchy–Schwarz inequality with weight $\varepsilon$,

9.77 $$\begin{aligned}&\rho _z \ell ^2\delta ^2 z \int _{{\mathbb {R}}^2} \widehat{\chi ^2}(p \ell ) ({\widetilde{a}}_p^\dagger + {\widetilde{a}}_p) \textrm{d}p \nonumber \\&\quad \le \varepsilon \rho _z \ell ^2 \delta ^2 z \int |\widehat{\chi ^2}(p \ell )| + C \varepsilon ^{-1} \rho _z \ell ^2 \delta ^2 z \int |\widehat{\chi ^2}(p \ell )| {\widetilde{a}}_p^\dagger {\widetilde{a}}_p \textrm{d}p \nonumber \\&\quad \le C \varepsilon \rho _z \delta ^2 z + C \varepsilon ^{-1} \rho _z \delta ^2 z n_+ . \end{aligned}$$

where we used Lemma (F.1). With $\varepsilon \simeq z \delta K_{\ell }^2 \vert \log \delta \vert$, the second part is absorbed by the positive fraction of $\frac{n_+}{\ell ^2}$, and the first term is of order $\rho ^2_z \ell ^2\delta ^3 K_\ell ^2 \vert \log \delta \vert$. Hence,

9.78 $$\begin{aligned} {\mathcal {Q}}_1^{\text {ex}}\ge & {} \frac{z \ell ^2}{(2\pi )^4} \int _{{\mathcal {P}}_H \times {\mathbb {R}}^2} {\widehat{W}}_1(k) \alpha _k \widehat{\chi ^2}(p \ell ) ({\widetilde{a}}_p^\dagger + {\widetilde{a}}_p) \textrm{d}k \textrm{d}p \nonumber \\{} & {} - C \rho ^2_z\ell ^2 \delta ^3 K_\ell ^2 \vert \log \delta \vert ^2 - \frac{b}{100} \frac{n_+}{\ell ^2} . \end{aligned}$$

Finally we want to insert the cutoff $f_L(p)$ inside the integral. The error we make is estimated similarly,

$$\begin{aligned}&\frac{z \ell ^2}{(2\pi )^4} \int _{{\mathcal {P}}_H \times {\mathbb {R}}^2} {\widehat{W}}_1(k) \alpha _k \widehat{\chi ^2}(p \ell ) (1-f_L(p)) ({\widetilde{a}}_p^\dagger + {\widetilde{a}}_p) \textrm{d}k \textrm{d}p \\&\ge - C z \ell ^2 \rho _z \delta \int _{{\mathcal {P}}_L^c} \widehat{\chi ^2}(p \ell ) ({\widetilde{a}}_p^\dagger + {\widetilde{a}}_p ) \textrm{d}p \\&\ge -C \varepsilon z \rho _z \delta \ell ^2 \int _{{\mathcal {P}}_L^c} |\widehat{\chi ^2}(p \ell )| \textrm{d}p - C \varepsilon ^{-1} z \rho _z \delta \ell ^2 \int _{{\mathcal {P}}_L^c} |\widehat{\chi ^2}(p \ell )| {\widetilde{a}}_p^\dagger {\widetilde{a}}_p \textrm{d}p \\&\ge - C \varepsilon z \rho _z \delta d^{4M-4} - C \varepsilon ^{-1} z \rho _z \delta d^{4M} n_+^H , \end{aligned}$$

where we used $\sup _{p \in {\mathcal {P}}_L^c} \vert \widehat{\chi ^2}(p\ell ) \vert \le C d^{4M}$. With $\varepsilon \simeq z K_{\ell }^2 d^{4M+2} \varepsilon ^{-1}_T$ the first part is of order $\rho ^{2}_z \ell ^2 \delta K_\ell ^2 d^{8M-2} \varepsilon _T^{-1}$ and the second is absorbed in a fraction of $\frac{\varepsilon _T n_+^H}{(d \ell )^2}$. $\square$

Now we have all we need to estimate ${\mathcal {Q}}_3^{(4)}$.

##### Lemma 9.11

(Estimates on ${\mathcal {Q}}_3^{(4)}$). For any state $\Phi$ satisfying (8.44) we have

$$\begin{aligned} \left\langle {\mathcal {Q}}_3^{(4)} + {\mathcal {Q}}_1^{\text {ex}} + \frac{b}{100} \frac{n_+}{\ell ^2} + \frac{b}{100} \frac{ \varepsilon _T n_+^H}{(d\ell )^2} \right\rangle _{\Phi } \ge&- C \rho _z^2\ell ^2 \delta ^3 K_\ell ^2 \vert \log \delta \vert ^2 - C \rho _z^2\ell ^2 \delta K_\ell ^2 d^{8M-2} \varepsilon _T^{-1} \\&-C \rho _z^2 \ell ^2 \delta ^2 \varepsilon _{{\mathcal {M}}}^{1/2} (K_\ell ^4 K_H^{-4} + \delta ^{-1} K_H^{-M} d^{-2}) . \end{aligned}$$

##### Proof

We use the commutator formula

$$\begin{aligned}{}[ b_{p-k}, b_{-k}^{\dagger }] = (1-\alpha _k \alpha _{p-k}) \Big ( \widehat{\chi ^2}(p\ell ) - {{\widehat{\chi }}} (k \ell ) {{\widehat{\chi }}} ((p-k)\ell ) \Big ), \end{aligned}$$

and split into ${\mathcal {Q}}_3^{(4)} = I + II$, with

$$\begin{aligned} I&= - \frac{z \ell ^2}{(2\pi )^4} \int _{{\mathcal {P}}_H \times {\mathbb {R}}^2} \frac{f_L(p) {{\widehat{W}}}_1(k)}{\sqrt{1-\alpha _k^2} \sqrt{1-\alpha _{p-k}^2}} \alpha _k (1-\alpha _k \alpha _{p-k}) \widehat{\chi ^2}(p\ell ) ({\widetilde{a}}_p^\dagger + {\widetilde{a}}_p) \textrm{d}k \textrm{d}p, \end{aligned}$$

and

$$\begin{aligned} II&= - \frac{z \ell ^2}{(2\pi )^4} \int _{{\mathcal {P}}_H \times {\mathbb {R}}^2} \frac{f_L(p) \widehat{W}_1(k)}{\sqrt{1-\alpha _k^2} \sqrt{1-\alpha _{p-k}^2}}\\&\quad \times \alpha _k (1-\alpha _k \alpha _{p-k}){{\widehat{\chi }}} (k\ell ) \widehat{\chi }((p-k)\ell ) ({\widetilde{a}}_p^\dagger + {\widetilde{a}}_p) \textrm{d}k \textrm{d}p . \end{aligned}$$

In *I* we recognize the lower bound on ${\mathcal {Q}}_1^{\text {ex}}$ given by Lemma 9.10 with opposite sign, up to an error term:

$$\begin{aligned}{} & {} I + {\mathcal {Q}}_1^{\text {ex}} + \frac{b}{100} \frac{n_+}{\ell ^2} + \frac{b}{100} \frac{ \varepsilon _T n_+^H}{(d\ell )^2} + C\rho ^2_z\ell ^2 \delta ^3 K_\ell ^2 \vert \log \delta \vert ^2 + \rho ^{2}_z \ell ^2 \delta K_\ell ^2 d^{8M-2} \varepsilon _T^{-1} \\{} & {} \quad \ge - C z \ell ^2 \int _{{\mathcal {P}}_H \times {\mathbb {R}}^2} f_L(p) \widehat{W}_1(k) \alpha _k^3 \widehat{\chi ^2}(p\ell ) ({\widetilde{a}}_p^\dagger + {\widetilde{a}}_p) \textrm{d}k \textrm{d}p . \end{aligned}$$

This remaining integral can be estimated, by (9.48), as

$$\begin{aligned}&\left| z \ell ^2 \int _{{\mathcal {P}}_H \times {\mathbb {R}}^2} f_L(p) \widehat{W}_1(k) \alpha _k^3 \widehat{\chi ^2}(p\ell ) ({\widetilde{a}}_p^\dagger + {\widetilde{a}}_p) \textrm{d}k \textrm{d}p \right| \\ {}&\le C \vert z \vert \ell ^2 \rho _z^3 \delta ^4 \int _{{\mathcal {P}}_H} k^{-6} \textrm{d}k \int _{{\mathcal {P}}_L} |\widehat{\chi ^2}(p\ell )| (\widetilde{a}_p^\dagger + {\widetilde{a}} _p) \textrm{d}p, \end{aligned}$$

and after applying to the state $\Phi$ we use a Cauchy–Schwarz inequality with weight $\sqrt{\mathcal {M}}$,

$$\begin{aligned} \Big \vert z&\ell ^2 \int _{{\mathcal {P}}_H \times {\mathbb {R}}^2} f_L(p) {{\widehat{W}}}_1(k) \alpha _k^3 \widehat{\chi ^2}(p\ell ) \langle {\widetilde{a}}_p^\dagger + {\widetilde{a}}_p \rangle _{\Phi } \textrm{d}k \textrm{d}p \Big \vert \\&\le C z \rho _z^3\ell ^6 \delta ^4 K_H^{-4} \Big ( \sqrt{ {\mathcal {M}}} \int _{{\mathcal {P}}_L} |\widehat{\chi ^2}(p\ell )| \textrm{d}p + \frac{1}{\sqrt{ {\mathcal {M}}}} \int _{{\mathcal {P}}_L} |\widehat{\chi ^2}(p\ell )| \langle {\widetilde{a}}_p^\dagger {\widetilde{a}}_p \rangle _{\Phi } \textrm{d}p \Big ) \\&\le C z \rho _z^3 \ell ^4 \delta ^4 K_H^{-4} \sqrt{{\mathcal {M}}} \le C \rho _z^2 \ell ^2 \delta ^2 \big (K_\ell ^4 K_H^{-4} \sqrt{\varepsilon _{{\mathcal {M}}}}\big ). \end{aligned}$$

Finally we bound, by (F4) and (9.50),

$$\begin{aligned} \vert \langle II \rangle _{\Phi } \vert&\le z \ell ^2 \sup _{h \in {\mathcal {P}}_H}|{\widehat{\chi }}(h\ell )|\int _{{\mathcal {P}}_H} |{{\widehat{W}}}_1(k)| \alpha _k |{{\widehat{\chi }}}(k \ell )| \textrm{d}k \int _{{\mathcal {P}}_L} \langle {\widetilde{a}}_p^\dagger + {\widetilde{a}}_p \rangle _{\Phi } \textrm{d}p\\&\le C z \rho _z \ell ^2 \delta K_H^{-M}\Big ( d^2 {\mathcal {M}}^{1/2} \int _{{\mathcal {P}}_L} \textrm{d}p + d^{-2} {\mathcal {M}}^{-1/2} \int _{{\mathcal {P}}_L} \langle {\widetilde{a}}_p^\dagger {\widetilde{a}}_p \rangle _{\Phi } \textrm{d}p \Big ), \end{aligned}$$

where we used a Cauchy–Schwarz inequality with weight $d^2 \sqrt{{\mathcal {M}}}$. Thus,

$$\begin{aligned} \vert \langle II \rangle _\Phi \vert \le C \rho ^2_z\ell ^2 \delta K_H^{-M} d^{-2}\varepsilon _{{\mathcal {M}}}^{1/2}. \end{aligned}$$

$$\square $$

### Conclusion: Proof of Theorem 6.7

In Sect. 6 we showed how the proof of Theorem 2.3 is reduced to the proof of Theorem 6.7, which we give here.

#### Proof of Theorem 6.7

Recall the choices of the parameters in “Appendix H”. Let us consider a normalized *n*-particle state $\Psi \in {\mathscr {F}}_s(L^2(\Lambda ))$ which satisfies (7.40) for a certain large constant $C_0>0$,

9.79 $$\begin{aligned} \langle {\mathcal {H}}_{\Lambda }(\rho _{\mu })\rangle _{\Psi } \le -4 \pi \rho _{\mu }^2 \ell ^2 Y \big ( 1 - C_0 K_B^2 Y \vert \log Y \vert \big ). \end{aligned}$$

If such a state does not exists, our desired lower bound follows, because

9.80 $$\begin{aligned} -4 \pi \rho _{\mu }^2 \ell ^2 Y \big ( 1 - C_0 K_B^2 Y \vert \log Y \vert \big )\ge -4\pi \rho _\mu ^2 \ell ^2\delta \Big (1-\Big (2\Gamma +\frac{1}{2} +\log \pi \Big )\delta \Big ). \nonumber \\ \end{aligned}$$

So we can assume the existence of $\Psi$. By Theorem 7.7 there exists a sequence of *n*-particle states $\{\Psi ^m\}_{m \in {\mathbb {Z}}} \subseteq {\mathscr {F}}_s(L^2(\Lambda ))$ and $C_1, \eta _1>0$ such that

$$\begin{aligned}{} & {} \langle \Psi , {\mathcal {H}}_{\Lambda }(\rho _{\mu }) \Psi \rangle \ge \sum _{2|m|\le {\mathcal {M}} }\langle \Psi ^{(m)}, {\mathcal {H}}_{\Lambda }(\rho _{\mu }) \Psi ^m\rangle -C_1 \rho _{\mu }^2 \ell ^2 \delta ^{2+\eta _1} \\{} & {} \quad -4 \pi \rho _{\mu }^2 \ell ^2 Y \big ( 1 - C_1 K_B^2 Y \vert \log Y \vert \big )\sum _{2|m| > {\mathcal {M}}} \Vert \Psi ^m\Vert ^2. \end{aligned}$$

For $|m| \le \frac{{\mathcal {M}}}{2}$, we have that $\Psi ^m = \mathbb {1}_{[0,{\mathcal {M}}]}(n_+^L) \Psi ^m$. If we prove the lower bound for all $\Psi ^m$ such that $|m| \le \frac{{\mathcal {M}}}{2}$ then we would get (using (9.80) with $C_0$ replaced by $C_1$)

$$\begin{aligned} \langle \Psi , {\mathcal {H}}_{\Lambda }(\rho _{\mu }) \Psi \rangle \ge&-4\pi \rho _\mu ^2 \ell ^2\delta \Big (1-\Big (2\Gamma +\frac{1}{2} +\log \pi \Big )\delta \Big )\sum _{m }\Vert \Psi ^{(m)}\Vert ^2 -C_1 \rho _{\mu }^2 \ell ^2 \delta ^{2+\eta _1}, \end{aligned}$$

Therefore, the theorem is proven if we derive the corresponding lower bound for any *n*-particle, normalized state ${{\widetilde{\Psi }}} \in {\mathscr {F}}_s(L^2(\Lambda ))$ such that

9.81 $$\begin{aligned} {{\widetilde{\Psi }}} = \mathbb {1}_{[0,{\mathcal {M}}]}(n_+^L) {\widetilde{\Psi }}. \end{aligned}$$

By Proposition 8.3, for such a state there exists a constant $C_2 >0$ such that

9.82 $$\begin{aligned} \langle {\widetilde{\Psi }}, {\mathcal {H}}_{\Lambda }(\rho _{\mu }) {\widetilde{\Psi }}\rangle \ge \langle {\widetilde{\Psi }}, {\mathcal {H}}_{\Lambda }^{\text {2nd}}(\rho _{\mu }){\widetilde{\Psi }}\rangle -C_2 \ell ^2 \rho _{\mu }^2 \delta \big ( d^{2M-2} + R^2 \ell ^{-2} \big ), \end{aligned}$$

where the last term is an error term of order $\rho _{\mu }^2 \ell ^2 \delta ^{2+\eta _2}$, for some $\eta _2 >0$, thanks to relations (H25) and (H3). Then, by Theorem 8.4, there exists a constant $C_3>0$ such that

9.83 $$\begin{aligned} \langle {\widetilde{\Psi }},{\mathcal {H}}_{\Lambda }^{\text {2nd}} {\widetilde{\Psi }} \rangle \ge \inf _{z \in {\mathbb {R}}_+} \inf _{\Phi } \langle \Phi , {\mathcal {K}}(z) \Phi \rangle - C_3 \rho _{\mu } \delta (1 + \varepsilon _R K_{\ell }^4 K_B^2 |\log Y|), \end{aligned}$$

where the infimum is over the $\Phi$’s which satisfy (8.44). The last term is an error term of order $\rho _{\mu }^2 \ell ^2 \delta ^{2+\eta _3}$ for some $\eta _3>0$, thanks to relation (H19). The proof is reduced now to getting a lower bound for ${\mathcal {K}}(z)$. We have two cases, according to different values of *z*:

- If $|\rho _z -\rho _{\mu }| \ge C \rho _{\mu } \max ((\delta _1+ \delta _2+ \delta _3)^{1/2}, \delta ^{1/2})$ then Proposition 9.1 implies the bound
  9.84 $$\begin{aligned} \langle {\mathcal {K}}(z)\rangle _{\Phi } \ge -\frac{1}{2} \rho _{\mu }^2 \ell ^2 {\widehat{g}}_0 + 8\pi \Big (2\Gamma +\frac{1}{2} +\log \pi \Big )\rho _{\mu }^2 \ell ^2 \delta ^2, \end{aligned}$$
  and the second term is twice the LHY-term and positive, therefore there is nothing more to prove;
- Otherwise $|\rho _z -\rho _{\mu }| \le \rho _\mu K_{\ell }^{-2}$ (see Sect. 9.2). In this case we can use (9.27) and Theorem 9.3 to obtain $C_4,\eta _4 >0$, such that
  9.85 $$\begin{aligned} \langle {\mathcal {K}}(z) \rangle _{\Phi }&\ge -\frac{1}{2} \rho _{\mu }^2 \ell ^2 {\widehat{g}}_0+ (1-\varepsilon _K)\langle {\mathcal {K}}_H^{\text {Diag}}\rangle _{\Phi } +4\pi \Big (2\Gamma +\frac{1}{2} +\log \pi \Big ) \rho ^2_{z} \ell ^2 \delta ^2 \nonumber \\&\quad + \Big \langle b\frac{n_+}{4\ell ^2} + b \frac{\varepsilon _T n_+^H}{8 d^2\ell ^2} + {\mathcal {Q}}_1^{\text {ex}}(z) + {\mathcal {Q}}_2^{\text {ex}} + {\mathcal {Q}}_3(z)\Big \rangle _{\Phi } - C_4\rho _{\mu }^2 \ell ^2\delta ^{2+\eta _4}, \end{aligned}$$
  where we used that $C \rho _{\mu }^2 \ell ^2 \delta ( K_H^{4-M} K_{\ell } \delta ^{-1/2}) + | r(\rho _{\mu }) | \ell ^2 \le C\rho _{\mu }^2 \ell ^2\delta ^{2+\eta _4}$, thanks to the relations (H7), (H8) and that $M >4$. We conclude observing that, thanks to Theorem 9.4, we have the existence of $C_5,\eta _5 >0$ such that
  9.86 $$\begin{aligned} \Big \langle (1-\varepsilon _K){\mathcal {K}}_H^{\text {Diag}} + {\mathcal {Q}}_3(z) + {\mathcal {Q}}_2^{\text {ex}} + {\mathcal {Q}}_1^{\text {ex}} + \frac{b}{100} \frac{n_+}{\ell ^2} + \frac{b\varepsilon _T}{100}\frac{ n_+^{H}}{(d\ell )^{2}} \Big \rangle _{\Phi } \ge - C_5 \rho _z^2\ell ^2 \delta ^{2 + \eta _5}, \nonumber \\ \end{aligned}$$
  where the error has been obtained using relations (H10), (H18), (H21), (H26) and (H27). Thanks to the assumptions on $\rho _z$ and $\rho _{\mu }$, there exist $C_6,\eta _6>0$ such that
  9.87 $$\begin{aligned} |\rho _z^2\ell ^2 \delta ^{2 } - \rho _{\mu }^2\ell ^2 \delta ^{2 }| \le C_6 \rho _{\mu }^2 \ell ^2 \delta ^2 K_{\ell }^{-2}= C_6 \rho _{\mu }^2 \ell ^2 \delta ^{2+\eta _6}, \end{aligned}$$
  so that, plugging (9.86) into (9.85) and substituting the $\rho _z$ by the $\rho _{\mu }$ using (9.87) gives the desired lower bound and the right order for the error terms.

We choose $C = \sum _{j=1}^6 C_j$ and $\eta = \min _{j=1,\ldots , 6} \eta _j$. We conclude using that ${\widehat{g}}_0=8\pi \delta$ to get that

$$\begin{aligned}\inf _{z \in {\mathbb {R}}_+} \inf _{\Phi } \langle \Phi , {\mathcal {K}}(z) \Phi \rangle \ge -4\pi \ell ^2\rho _\mu ^2\delta \Big (1-\Big (2\Gamma +\frac{1}{2} +\log \pi \Big )\delta \Big )-C\rho _{\mu }^2 \ell ^2 \delta ^{2+\eta }.\end{aligned}$$

This finishes the proof of Theorem 6.7. $\square$

## Data Availability

Data sharing not applicable to this article as no datasets were generated or analysed during the current study.

## Appendix A: Reduction to Smaller Boxes for the Upper Bound

We provide here the necessary tools to go from a fixed box with compactly support potentials in the grand canonical setting, Theorem 2.2, to the thermodynamic limit with potentials allowing a tail, Theorem 2.1. The same techniques can be found in [17] with only minor deviations surrounding the non-compactness of the potential.

Given a potential *v*, we define

$$\begin{aligned}e(\rho ):=\lim _{L\rightarrow \infty }e_L(\rho )=\lim _{L\rightarrow \infty }\inf _{\psi \in H_0^1(\Lambda _L^{\rho L^2})}\frac{\langle {\psi , {\mathcal {H}}_v^{\rho L^2}\psi }\rangle }{L^2},\end{aligned}$$

where the limit is taken such that $\rho L^2=N \in {\mathbb {N}}$ and

$$\begin{aligned} {\mathcal {H}}_v^{N}=\sum _{i=1}^N -\Delta _i+\sum _{i<j}^N v(x_i-x_j). \end{aligned}$$

We write $v=v \mathbb {1}_{B(0,R)}+v \mathbb {1}_{B(0,R)^c}=v_R+v_{tail}$ where the $v_{tail}$ will always be treated as an error term. Let $v_{R}^{per}(x)=\sum _{k\in {\mathbb {Z}}^2}v_{R}(x+kL)$. In order for this to be finite we understand *R* to be smaller than *L*. We omit the *N* in the hamiltonian when it is operating on the Fock space.

The result below evaluates the error when going from periodic boundary conditions to Dirichlet boundary conditions.

### Lemma A.1

There exists a universal $C>0$, such that given $R_0>0$ and a periodic, bosonic trial function $\Psi _L\in {\mathscr {F}}(\Lambda _L)$, there exists a Dirichlet trial function $\Psi _{L+2R_0}^D\in {\mathscr {F}}(L^2(\Lambda _{L+2R_0}))$ satisfying, for $j\in {\mathbb {N}}_0$,

A1 $$\begin{aligned} \langle {\Psi _{L+2R_0}^D, {\mathcal {N}}^j\Psi _{L+2R_0}^D}\rangle&=\langle {\Psi _L, {\mathcal {N}}^j\Psi _L}\rangle , \end{aligned}$$

and

A2 $$\begin{aligned} \langle {\Psi _{L+2R_0}^D, {\mathcal {H}}_{v_{R}}\Psi _{L+2R_0}^D}\rangle&\le \langle {\Psi _L, {\mathcal {H}}_{v_{R}^{per}}\Psi _L}\rangle +\frac{C}{LR_0}\langle {\Psi _L, {\mathcal {N}}\Psi _L}\rangle . \end{aligned}$$

### Proof

The result is independent of dimension, see [31, Lemma 2.1.3] or [17, Lemma A.1] for a proof in the 3D case. $\square$

Next step is to glue the Dirichlet boxes together in order to construct a trial function on a thermodynamic box.

### Theorem A.2

Let $\Psi ^D_{L+2R_0}\in {\mathscr {F}}_s(L^2(\Lambda _{L+2R_0}))$ be a trial function with Diriclet boundary conditions and extend it to ${\mathbb {R}}^2$ by 0. Then for ${L}_k=k(L+2R_0+R)$, $k\in {\mathbb {N}}$, we define $\Psi _{{L}_k}\in {\mathscr {F}}_s(L^2(\Lambda _{{L}_k}))$ by

A3 $$\begin{aligned} \Psi ^{(m)}_{L_k}(x_1,\ldots ,x_m)= \frac{1}{\Vert (\Psi _{L+2R_0}^{D })^{(n)}\Vert ^{k^2-1}}\prod _{i=1}^{k^2}(\Psi _{L+2R_0}^{D })^{(n)}(x_{1+n(i-1)}-c_i,\ldots ,x_{in}-c_i),\nonumber \\ \end{aligned}$$

if $m = n k^2$, and $\Psi _{L_k}^{(m)} = 0$ otherwise. Here $c_i$ defines an enumeration of the lattice points on ${\mathbb {Z}}^2(L+2R_0)$. Then $\Psi _{L_k}$ satisfies

A4 $$\begin{aligned} \langle {\Psi _{L_k}, {\mathcal {N}}^j\Psi _{L_k}}\rangle =k^{2j} \langle {\Psi _{L+2R_0}^D, {\mathcal {N}}^j\Psi _{L+2R_0}^D}\rangle ,\qquad j\in {\mathbb {N}}_0. \end{aligned}$$

Furthermore if *v* satisfies the decay condition (1.3) of Theorem 1.1, then there exits a constant *C* only depending on $\eta _0$ and $C_0$ such that

A5 $$\begin{aligned} \langle {\Psi _{L_k}, {\mathcal {H}}_v\Psi _{L_k}}\rangle \le k^{2} \langle {\Psi _{L+2R_0}^D, {\mathcal {H}}_{v_{R}}\Psi ^D_{L+2R_0}}\rangle +k^2\langle {\Psi ^D_{L+2R_0}, {\mathcal {N}}^2\Psi ^D_{L+2R_0}}\rangle \frac{C a^{\eta _0}}{R^{2+\eta _0}}.\qquad \end{aligned}$$

### Proof

The expectation of ${\mathcal {N}}^j$ can be computed using that

$$\begin{aligned}\Vert \Psi _{L_k}^{(m)}\Vert ^2={\left\{ \begin{array}{ll} \Vert \Psi _{L+2R_0}^{(n)}\Vert ^2 &{}\text {if}\quad m=k^2n,\\ 0 &{}\text {otherwise}. \end{array}\right. }\end{aligned}$$

However for the potential energy we need to estimate the interaction between the boxes and the long range interaction inside the box. We observe that

A6 $$\begin{aligned} \langle {\Psi _{L_k}, {\mathcal {H}}_v\Psi _{L_k}}\rangle -k^{2} \langle {\Psi _{L+2R_0}^D, {\mathcal {H}}_{v_{R}}\Psi _{L+2R_0}^D}\rangle&= \sum _{n\ge 0} \sum _{i<j}^{k^2n}\int \vert \Psi _{L_k}^{(k^2n)}\vert ^2v_{tail}(x_i-x_j) \textrm{d}x, \nonumber \\ \end{aligned}$$

where we used that the kinetic energy of the two terms are equal and only the tail of the potential survives due to the corridors between the boxes. We further estimate

A7 $$\begin{aligned} \sum _{n\ge 0} \sum _{i<j}^{k^2n}\int \vert \Psi _{L_k}^{(k^2n)}\vert ^2v_{tail}(x_i-x_j)\textrm{d}x&\le \sum _{n\ge 0}k^2n \sum _{j=2}^{k^2n}\int \vert \Psi _{L_k}^{(k^2n)}\vert ^2v_{tail}(x_1-x_j) \textrm{d}x \nonumber \\&\le \sum _{n\ge 0}k^2n \sum _{j=2}^{k^2n}\int \vert \Psi _{L_k}^{(k^2n)}\vert ^2\frac{C a^{\eta _0}}{|x_1-x_j|^{2+\eta _0}} \textrm{d}x, \end{aligned}$$

where we used (1.3). If $s \in {\mathbb {N}}$ denotes the number of aligned boxes separating $x_1$ from $x_j$, then $|x_1 - x_j| \ge (s-1)L + R$ and there are $4(s+1)+1$ of such possible boxes. Summing on *s* we get

$$\begin{aligned} (A7)&\le \sum _{n\ge 0}k^2n\sum _{s=1 }^k\frac{C_0a^{\eta _0}n(4(s+1)+1)}{((s-1)L+R)^{2+\eta _0}}\Vert \Psi _{L_k}^{(k^2n)}\Vert ^2\\&\le k^2\langle {\Psi ^D_{L+2R_0}, {\mathcal {N}}^2\Psi _{L+2R_0}^D}\rangle C_0\Big (\frac{9a^{\eta _0}}{R^{2+\eta _0}}+\frac{a^{\eta _0}}{L^{2+\eta _0}}\sum _{s=1}^\infty \frac{4}{s^{1+\eta _0}}+\frac{9}{s^{2+\eta _0}}\Big ). \end{aligned}$$

In fact the largest term is the contribution of $v_{tail}$ inside the box and its 8 neighbours which here is represented by the term $\frac{9a^{\eta _0}}{R^{2+\delta }}$. $\square$

We have thus far constructed a sequence of grand canonical trial functions on larger and larger boxes, where we control the energy and the expected number of particles. The last part will be to relate this sequence to $e(\rho )$. For this we will use the continuity and convexity of $e(\rho )$ see [32, Thm. 3.5.8 and 3.5.11] together with the Legendre transformation being an involution on such functions.

### Theorem A.3

Let $\Psi _{L_k}\in {\mathscr {F}}(L^2(\Lambda _{L_k}))$ be a sequence with Dirichlet boundary conditions such that $L_k\rightarrow \infty$ as $k\rightarrow \infty$. Assume that there exist $C,c>0$ such that, for all $k\in {\mathbb {N}}$,

$$\begin{aligned}\langle {\Psi _{L_k}, {\mathcal {N}}\Psi _{L_k}}\rangle \ge \rho (1+c\rho )L_k^2,\qquad \quad \langle {\Psi _{L_k}, {\mathcal {N}}^2\Psi _{L_k}}\rangle \le C(\rho L_k^2)^2,\end{aligned}$$

then

$$\begin{aligned}e(\rho )\le \lim _{k\rightarrow \infty }\frac{\langle {\Psi _{L_k}, {\mathcal {H}}_v\Psi _{L_k}}\rangle }{L_k^2}.\end{aligned}$$

### Proof

We insert a chemical potential $\mu$, and find that, using the positivity of ${\mathcal {H}}_v$ and ${\mathcal {N}}$, for any $\mu \ge 0$ and $M>0$ we have

$$\begin{aligned} \begin{aligned}&\frac{\langle {\Psi _{L_k}, {\mathcal {H}}_v\Psi _{L_k}}\rangle }{L_k^2}\\ {}&\ge \frac{\langle {\Psi _{L_k}, ({\mathcal {H}}_v-\mu {\mathcal {N}})\chi ({\mathcal {N}}\le ML_k^2)\Psi _{L_k}}\rangle }{L_k^2}+\frac{\mu }{L_k^2}\big (\langle {\Psi _{L_k}, {\mathcal {N}}\Psi _{L_k}}\rangle -\langle {\Psi _{L_k}, {\mathcal {N}}\chi ({\mathcal {N}}\ge ML_k^2)\Psi _{L_k}}\rangle \big )\\ {}&\ge \sum _{m=0}^{ML_k^2}\Big (e_{L_k}\Big (\frac{m}{L_k^2}\Big )-\mu \frac{m}{L_k^2}\Big )\Vert \Psi _{L_k}^{(m)}\Vert ^2+\frac{\mu }{L_k^2}\Big (\langle {\Psi _{L_k}, {\mathcal {N}}\Psi _{L_k}}\rangle -\frac{1}{ML_k ^2}\langle {\Psi _{L_k}, {\mathcal {N}}^2\Psi _{L_k}}\rangle \Big ). \end{aligned} \end{aligned}$$

Fixing *M* large enough in terms of *C* and *c* then gives

A8 $$\begin{aligned} \frac{ \langle {\Psi _{L_k}, {\mathcal {H}}\Psi _{L_k}}\rangle }{L_{k}^2}\ge \sum _{m=0}^{ML^2}\Big (e_{L_k}\Big (\frac{m}{L_k^2}\Big )-\mu \frac{m}{L_k^2}\Big )\Vert \Psi _{L_k}^{(m)}\Vert ^2+\mu \rho . \end{aligned}$$

As in Theorem A.2, we glue several copies of a minimizer of $e_{L_k}(\rho )$, each copy living on a different box. We leave corridors of size $L_k^{1-\epsilon }$ between the boxes and this has the consequence of changing the density to $\rho (1+L_k^{-\epsilon })^{-2}$. Assuming further that *v* satisfies the conditions of Theorem 1.1 we estimate the ignored interactions to find

A9 $$\begin{aligned} e(\rho (1+L_k^{-\epsilon })^{-2})\le e_{L_k}(\rho )(1+L_k^{-\epsilon })^{-2}+\frac{C(\rho L_k^2)^2}{L_{k}^{4+\eta _0-(2+\eta _0)\epsilon }}. \end{aligned}$$

Using (A9) in (A8) yields

$$\begin{aligned} L_k^{-2} \langle {\Psi _{L_k}, {\mathcal {H}}\Psi _{{L}_k}}\rangle&\ge \mu \rho +(1+{L}_k^{-\epsilon })^2\sum _{m=0}^{M{L}_k^2}\Big (e\Big (\frac{m}{{L}_k^2}(1+{L}_k^{-\epsilon })^{-2}\Big )\\&\quad -(1+{L}_k^{-\epsilon })^{-2}\mu \frac{m}{{L}_k^2}-\frac{Cm^2}{{L}_k^{4+\eta _0-(2+\eta _0)\epsilon }}\Big )\Vert \Psi _{{L}_k}^{(m)}\Vert ^2\\ {}&\ge \mu \rho -(1+{L}_k^{-\epsilon })^{2}e^*(\mu )-C\rho ^2{L}_k^{-\eta _0+(2+\eta _0)\epsilon }, \end{aligned}$$

where $^*$ defines the Legendre transformation with respect to the interval [0, *M*]. Choosing $\epsilon >0$ small enough and letting *k* go to infinity yields

A10 $$\begin{aligned} \lim _{k\rightarrow \infty }\frac{ \langle {\Psi _{{L}_k}, {\mathcal {H}}\Psi _{{L}_k}}\rangle }{{L}_{k}^2}\ge \sup _{\mu \in [0,\infty )}(\mu \rho -e^*(\mu ))=\sup _{\mu \in {\mathbb {R}}}(\mu \rho -e^*(\mu ))=e(\rho ), \end{aligned}$$

where we used that $e^*(\mu )\ge 0$ for all $\mu \in {\mathbb {R}}$ and that the Legendre transformation is an involution. $\square$

We end the section by giving the proof of the final upper bound Theorem 2.1 using the result of Theorem 2.2.

### Proof of Theorem 2.1

We first cut our potential in order to apply Theorem 2.2. We write

$$\begin{aligned}v=v\mathbb {1}_{B(0,R)}+v\mathbb {1}_{B(0,R)^c}=v_{R}+v_{tail},\end{aligned}$$

where $R=\rho ^{-\frac{1}{2}}Y^{\beta + 2}$. We denote by $a_{R}$ the scattering length of $v_{R}$. To get estimates on the energy density $e(\rho )$ we use the standard theory developed in “Appendix A”. The idea is to extend $L_{\beta }$ with $R_0$ and force the trial function to have Dirichlet boundary conditions on the box of sidelength $L_{\beta }+R_0$. Thereafter one glues together these small Dirichlet boxes, separated by corridors of size *R*. Since this process will slightly change the density, we choose for a given $\rho >0$, the larger density ${\widetilde{\rho }}$ satisfying

$$\begin{aligned}\rho ={\widetilde{\rho }}(1-2C\widetilde{Y}^2)\Big (1+\frac{2R_0}{L_\beta }+\frac{R}{L_\beta }\Big )^{-2},\end{aligned}$$

where *C* is the same as in Theorem 2.2, and $R_0=\rho ^{-\frac{1}{2}} Y^{-\frac{1}{2}}$. This choice of $R_0$ is in fact optimal as one can see from the error term $C \frac{\rho }{L_{\beta }R_0}$ coming from the glueing process in (A17). Here we use the notation $\widetilde{Y} = \vert \log ( {{\widetilde{\rho }}} a_{R}^2) \vert ^{-1}$ and $\widetilde{\delta _0} = \vert \log ( {{\widetilde{\rho }}} a_{R}^2\widetilde{Y}) \vert ^{-1}$. If $\rho a^2$ is small enough then ${\widetilde{\rho }} a_{R}^2 \le C^{-1}$, and we may use Theorem 2.2 to find a periodic trial state $\Psi$ for the density ${\widetilde{\rho }}$ and potential $v_{R}$ satisfying

A11 $$\begin{aligned} \langle {\mathcal {H}}_{v_R} \rangle _\Psi&\le 4\pi L_\beta ^2 {\widetilde{\rho }}^2 \widetilde{\delta _0} \Big (1 + \Big (2\Gamma + \frac{1}{2} + \log (\pi ) \Big ) \widetilde{\delta _0} \Big ) + CL_{\beta }^2 {\widetilde{\rho }}^2 \widetilde{\delta _0}^3\vert \log (\widetilde{\delta _0})\vert , \end{aligned}$$

with $$\langle {\mathcal {N}} \rangle _\Psi \ge {\widetilde{\rho }}L_\beta ^2(1-C\widetilde{Y}^2),$$ and $$\langle {\mathcal {N}}^2 \rangle _{\Psi } \le C {\widetilde{\rho }}^2 L_\beta ^4$$ .

Since $\frac{R}{L_\beta } \ll \frac{R_0}{L_\beta } = Y^{-\frac{1}{2}+\beta } \ll 1$, we have $\vert \rho - {{\widetilde{\rho }}} \vert \le C \rho Y^{-\frac{1}{2}+\beta }$, and we can change ${{\widetilde{\rho }}}$ into $\rho$ in (A11) up to smaller errors if

A12 $$\begin{aligned} \beta \ge \frac{5}{2}. \end{aligned}$$

One can show that the *C* appearing in (A11) only increases in $\beta$ (see (5.21)). Thus we find $\beta =5/2$ to be optimal. We can also change $a_{R}$ into *a* because the right-hand side of (A11) is an increasing function of the scattering length and $a_{R} \le a$. Thus,

A13 $$\begin{aligned} \langle {\mathcal {H}}_{v_{R}} \rangle _\Psi&\le 4\pi L_\beta ^2 \rho ^2 \delta _0 \Big (1 + \Big (2\Gamma + \frac{1}{2} + \log (\pi ) \Big ) \delta _0 \Big ) + CL_{\beta }^2 \rho ^2 \delta ^{3}_0\vert \log (\delta _0)\vert , \end{aligned}$$

and the bounds on the number of particles become

A14 $$\begin{aligned} \langle {\mathcal {N}} \rangle _\Psi \ge (\rho +c\rho ^2) (L_\beta +2R_0+R)^2 , \quad \text {and} \quad \langle {\mathcal {N}}^2 \rangle _{\Psi } \le C(\rho L_\beta ^2)^2, \end{aligned}$$

for some $$c> 0$$.

Now we can use Lemma A.1 and Theorem A.2 to glue small boxes together. We get a sequence $\Psi _{k(L_\beta +2R_0+R)}\in {\mathscr {F}}_s\big (L^{2}\big (\Lambda _{k(L_{\beta }+2R_0+R)}\big )\big )$ with Dirichlet boundary conditions, for $k\in {\mathbb {N}}$, on the box

$$\begin{aligned}\Lambda _{k(L_\beta + 2 R_0 + R)} = \Big [ - \frac{1}{2} k(L_\beta + 2 R_0 + R) , \frac{1}{2} k(L_\beta + 2 R_0 + R) \Big ]^2,\end{aligned}$$

satisfying

A15 $$\begin{aligned} \langle {\mathcal {N}} \rangle _{\Psi _{k(L_{\beta }+2R_0+R)}} = k^2\langle {\mathcal {N}} \rangle _\Psi ,\qquad \langle {\mathcal {N}}^2 \rangle _{\Psi _{k(L_{\beta }+2R_0+R)}} = k^4\langle {\mathcal {N}}^2 \rangle _\Psi , \end{aligned}$$

and

A16 $$\begin{aligned} \langle {\mathcal {H}}_v \rangle _{\Psi _{k(L_\beta +2R_0+R)}}\le k^2\Big (\langle {\mathcal {H}}_{v_{R}} \rangle _{\Psi }+\langle {\mathcal {N}} \rangle _{\Psi }\frac{C}{L_\beta R_0}+\langle {\mathcal {N}}^2 \rangle _{\Psi }\frac{Ca^{\eta _0}}{R^{2+\eta _0}}\Big ), \end{aligned}$$

where *C* only depends on $\eta _0$ and $C_0$. Note that we have the original potential in the left-hand side of (A16) because by (A5), $v_{tail}$ only produces an error term. By construction this sequence satisfies the conditions on the number of particles for Theorem A.3, and we conclude

A17 $$\begin{aligned} e^{2D}(\rho )\le \lim _{k\rightarrow \infty }\frac{\langle {\mathcal {H}}_v \rangle _{\Psi _{k(L_\beta +2R_0+R)}}}{ k^2L_\beta ^2}\le \frac{\langle {\mathcal {H}}_{v_{R}} \rangle _{\Psi }}{L^2_\beta }+ C \frac{\rho }{L_\beta R_0}+ Ca^{\eta _0}\frac{\rho ^2 L_\beta ^2}{R^{2+\eta _0}}, \end{aligned}$$

where in the last inequality we used (A16), (A14) and that $\langle {\mathcal {N}}\rangle ^2_{\Psi } \le \langle {\mathcal {N}}^2\rangle _{\Psi }$. With our choice of parameters including (A12), the two last terms in (A17) are errors. Theorem 2.1 follows from (A17) and (A13). $\square$

## Appendix B: Bogoliubov Diagonalization

### Theorem B.1

Let $a_{\pm }$ be operators on a Hilbert space satisfying $\left[ a_+, a_- \right] = 0$. For ${\mathcal {A}} > 0$, ${\mathcal {B}} \in {\mathbb {R}}$ satisfying either $\vert {\mathcal {B}} \vert < {\mathcal {A}}$ or ${\mathcal {B}} = {\mathcal {A}}$ and arbitrary $\kappa \in {\mathbb {C}}$, we have the operator identity

$$\begin{aligned} {\mathcal {A}}&(a^{\dagger }_+ a_+ + a^{\dagger }_- a_-) + {\mathcal {B}}(a_+^{\dagger } a^{\dagger }_- + a_+ a_-) + \kappa (a^{\dagger }_+ + a_-) + \overline{\kappa }(a_+ + a_-^{\dagger }) \\&= (1- \alpha ^2) {\mathcal {D}} ( b^\dagger _+ b_+ + b^\dagger _- b_- ) -\frac{1}{2} ({\mathcal {A}} - \sqrt{{\mathcal {A}}^2 - {\mathcal {B}}^2}) ([a_+,a^{\dagger }_+] + [a_-, a^{\dagger }_-]) - \frac{2|\kappa |^2}{{\mathcal {A}}+{\mathcal {B}}}, \end{aligned}$$

where $${\mathcal {D}} = \frac{1}{2} \big ( {\mathcal {A}} + \sqrt{{\mathcal {A}}^2 - {\mathcal {B}}^2}\big )$$ and

B1 $$\begin{aligned} b_+ = \frac{1}{\sqrt{1 - \alpha ^2}} \big ( a_+ + \alpha a_-^\dagger + {{\bar{c}}}_0 \big ), \quad b_- = \frac{1}{\sqrt{1 - \alpha ^2}} \big ( a_- + \alpha a_+^\dagger + c_0 \big ), \end{aligned}$$

with

B2 $$\begin{aligned} \alpha = {\mathcal {B}}^{-1} \big ( {\mathcal {A}} - \sqrt{{\mathcal {A}}^2 - {\mathcal {B}}^2} \big ), \quad c_0 = \frac{2 {\bar{\kappa }}}{{\mathcal {A}} + {\mathcal {B}} + \sqrt{{\mathcal {A}}^2 - {\mathcal {B}}^2}}. \end{aligned}$$

### Remark B.2

Note that the normalization of $b_\pm$ is chosen such that

B3 $$\begin{aligned}{}[ b_+, b_+^\dagger ] = \frac{ [ a_+ , a_+^\dagger ] - \alpha ^2 [ a_- , a_-^\dagger ]}{1 - \alpha ^2} , \end{aligned}$$

and we recover the canonical commutation relations $[ b_+, b_+^\dagger ] = 1$ when $a_+$ and $a_-$ satisfies them as well.

### Proof

This follows directly from algebraic computations. $\square$

## Appendix C: Calculation of the Bogoliubov Integral

For functions $\alpha , \beta$, and parameter $\varepsilon \ge 0$, we define

C1 $$\begin{aligned} I_{\varepsilon }(\alpha ,\beta ) :=\frac{1}{2(2 \pi )^2} \int _{{\mathbb {R}}^2}&\Big ( \sqrt{(1-\varepsilon )^2 \alpha ^2(k) + 2 (1-\varepsilon ) \rho \alpha (k) \beta (k) } - (1-\varepsilon ) \alpha (k) - \rho \beta (k) \nonumber \\ {}&\quad +\rho ^2 \frac{\widehat{g}^2_k - {{\widehat{g}}}^2_0\mathbb {1}_{\{|k|\le \ell _{\delta }^{-1}\}}}{k^2}\Big ) \textrm{d}k . \end{aligned}$$

We recall that ${\widehat{g}}_0 = 8 \pi \delta$, where $\delta$ satisfies $\frac{1}{2} Y \le \delta \le 2 Y$. We are mainly interested into two special cases, namely $I_0(k^2,{\widehat{g}})$ and $I_{\varepsilon _N}(\tau , {{\widehat{W}}}_1)$. In this section we estimate these integrals.

### Lemma C.1

We can replace $\tau _k$ by $k^2$ up to the following error,

$$\begin{aligned} \vert I_{\varepsilon _N}(\tau , {{\widehat{W}}}_1) - I_{\varepsilon _N}(k^2, {{\widehat{W}}}_1) \vert \le C \rho ^2 \delta ^2 \big ( d + \varepsilon _T \vert \log Y \vert + (sK_\ell )^{-1} \big ). \end{aligned}$$

### Proof

We recall the definition (8.8) of $\tau _k$,

$$\begin{aligned} \tau _k = (1-\varepsilon _T) \Big ( \vert k \vert - \frac{1}{2s\ell } \Big )^2_+ + \varepsilon _T \Big ( \vert k \vert - \frac{1}{2ds \ell } \Big )^2_+, \end{aligned}$$

from which we deduce the bounds

C2 $$\begin{aligned} \vert \tau _k - k^2 \vert&\le {\left\{ \begin{array}{ll} \frac{1}{2s\ell } \vert k \vert + \frac{1}{2(s\ell )^2}, &{} \text {if} \quad \vert k \vert > \frac{1}{2ds \ell },\\ \varepsilon _T \vert k \vert ^2 + \frac{3}{2 s\ell } \vert k \vert , &{}\text {if} \quad \frac{1}{2 s\ell }< \vert k \vert < \frac{1}{2ds \ell }. \end{array}\right. } \end{aligned}$$

We write the integral as

C3 $$\begin{aligned} I_{\varepsilon _N}(\tau ,{{\widehat{W}}}_1) = \frac{1}{2(2\pi )^2} \int _{{\mathbb {R}}^2} F_k(\tau _k,{{\widehat{W}}}_1(k)) \textrm{d}k, \end{aligned}$$

with

$$\begin{aligned} F_k(\tau ,w)&= \sqrt{(1-\varepsilon _N)^2 \tau ^2 + 2 (1-\varepsilon _N)\rho w \tau } \\ {}&\quad - (1-\varepsilon _N)\tau - \rho w + \rho ^2 \frac{{{\widehat{g}}}^2(k) - {{\widehat{g}}}^2(0) \mathbb {1}_{\{\vert k \vert \le \ell _\delta ^{-1}\}}}{2 k^2}. \end{aligned}$$

We first consider separately the small *k*’s. Indeed, $\tau _k =0$ for $\vert k \vert \le \frac{1}{2s \ell }$ and thus

$$\begin{aligned} \vert F_k(\tau _k, {{\widehat{W}}}_1(k)) - F_k(k^2, {{\widehat{W}}}_1(k)) \vert&= \Big \vert \sqrt{(1-\varepsilon _N)^2 k ^4 + 2 (1-\varepsilon _N)\rho {{\hat{W}}}_1(k) k^2} - (1-\varepsilon )k^2 \Big \vert \\&\le C \sqrt{\rho \delta } \vert k \vert , \end{aligned}$$

(recall that $(s K_\ell )^{-1} \ll 1$) and

C4 $$\begin{aligned} \frac{1}{2(2\pi )^2} \int _{\vert k \vert \le (2s\ell )^{-1}} \vert F_k(\tau _k,{{\widehat{W}}}_1(k)) - F_k(k^2,{{\widehat{W}}}_1(k)) \vert \textrm{d}k \le C \rho ^2 \delta ^2 (sK_\ell )^{-3}. \end{aligned}$$

The part with larger *k* we bound using the derivatives of *F* and deduce

C5 $$\begin{aligned}&\vert I_{\varepsilon _N}(\tau , {{\widehat{W}}}_1) - I_{\varepsilon _N}(k^2,{{\widehat{W}}}_1) \vert \\&\le \frac{1}{2(2\pi )^2} \int _{\{\vert k \vert > (2s \ell )^{-1}\}} \sup _{\tau \in [\tau _k, k^2] } \vert \partial _\tau F_k(\tau ,\widehat{W}_1(k)) \vert \cdot \vert \tau _k - k^2 \vert \textrm{d}k + C \rho ^2 \delta ^2 (sK_\ell )^{-3}.\nonumber \end{aligned}$$

The derivative of *F* is given by

C6 $$\begin{aligned} \partial _\tau F(\tau ,w) = \frac{(1-\varepsilon _N)^2 \tau + (1-\varepsilon _N) \rho w}{\sqrt{(1-\varepsilon _N)^2 \tau ^2 + 2 (1-\varepsilon _N) \rho w \tau }} - (1- \varepsilon _N) \end{aligned}$$

and can be estimated for $\tau \in [ \tau _k, k^2 ]$ as

C7

Indeed, for $\vert k \vert < \sqrt{\rho \delta }$, we just need to bound individualy each term in (C6), whereas for $\vert k \vert > \sqrt{\rho \delta }$, we have $\tau _k > C \rho \widehat{W}_1(k)$ and we use a Taylor expansion of the square root to get

$$\begin{aligned} \vert \partial _\tau F_k(\tau , {{\widehat{W}}}_1(k)) \vert \le C \frac{\rho ^2 {{\widehat{W}}}_1(k)^2}{(1-\varepsilon _N) \tau ^2} \le C \frac{\rho ^2 \delta ^2}{k^4}. \end{aligned}$$

Now we split the integral in (C5) into 3 parts $I_1, I_2, I_3$, corresponding to the integration on the domains $\{(2s\ell )^{-1}< k< \sqrt{\rho \delta }\}$, $\{\sqrt{\rho \delta }< k < (2ds\ell )^{-1}\}$ and $\{k >(2ds\ell )^{-1}\}$, respectively, and we use (C7), (C2) to bound it and find:

$$\begin{aligned} I_1&\le C \rho ^2 \delta ^2 \Big ( \varepsilon _T + \frac{1}{s K_\ell } \Big ),\\ I_2&\le C \rho ^2 \delta ^2 \Big ( \varepsilon _T \vert \log Y \vert + \frac{1}{sK_\ell } \Big ), \\ I_3&\le C \rho ^2 \delta ^2 d. \end{aligned}$$

$$\square $$

### Lemma C.2

We can replace ${{\widehat{W}}}_1(k)$ by ${{\widehat{g}}}_k$ up to the following error

$$\begin{aligned} \vert I_{\varepsilon _N}(k^2, {{\widehat{W}}}_1) - I_{\varepsilon _N}(k^2, {{\widehat{g}}}) \vert \le C \rho ^2 \delta ^2 K_\ell ^{-1} + C \rho ^2 \delta \varepsilon _N.\end{aligned}$$

### Proof

Recall that $I_{\varepsilon _N}(k^2, {{\widehat{W}}}_1)$ is given by (C3). We first use (6.26) and (3.34) to replace the last part,

$$\begin{aligned} \rho ^2 \int _{{\mathbb {R}}^2} \frac{{{\widehat{g}}}_k^2 - {{\widehat{g}}}_0^2 \mathbb {1}_{\{\vert k \vert \le \ell _\delta ^{-1}\}}}{2 k^2} \textrm{d}k&= \rho ^2 \int _{{\mathbb {R}}^2} \frac{{{\widehat{W}}}_1(k)^2 - {{\widehat{W}}}_1(0)^2 \mathbb {1}_{\{\vert k \vert \le \ell _\delta ^{-1}\}}}{2 (1-\varepsilon _N) k^2} \textrm{d}k\\&\quad + {\mathcal {O}}( \rho ^2 \delta \varepsilon _N + \rho ^2 \delta ^2 K_\ell ^{-2}), \end{aligned}$$

so that

C8 $$\begin{aligned} I_{\varepsilon _N}(k^2, {{\widehat{W}}}_1) = J({{\widehat{W}}}_1) + {\mathcal {O}}( \rho ^2 \delta \varepsilon _N + \rho ^2 \delta ^2 K_\ell ^{-2}) \quad \text {and} \quad I_{\varepsilon _N}(k^2, \widehat{g}) = J({{\widehat{g}}}), \end{aligned}$$

with

C9 $$\begin{aligned} J(w) = \frac{1}{2(2\pi )^2} \int _{{\mathbb {R}}^2} G_k(w_k,w_0) \textrm{d}k, \end{aligned}$$

and

$$\begin{aligned} G_k(w,w_0)&= \sqrt{(1-\varepsilon _N)^2 k^4 + 2 (1-\varepsilon _N)\rho w k^2} \\&\quad - (1-\varepsilon _N)k^2 - \rho w + \rho ^2 \frac{w^2 - w_0^2 \mathbb {1}_{\{\vert k \vert \le \ell _\delta ^{-1}\}}}{2 (1-\varepsilon _N) k^2}. \end{aligned}$$

Note that $G_k$ is independent of $w_0$ for $\vert k \vert > \ell _\delta ^{-1}$. Then we split *J*(*w*) into two parts,

C10 $$\begin{aligned} J(w)&= \frac{1}{2(2\pi )^2} \int _{\{\vert k \vert< \ell ^{-1}_\delta \}} G_k(w_k,w_0) \textrm{d}k + \frac{1}{2(2\pi )^2} \int _{\{\vert k \vert> \ell ^{-1}_\delta \}} G_k(w_k) \textrm{d}k \nonumber \\&=: J_<(w) + J_>(w). \end{aligned}$$

For $k > \ell _\delta ^{-1}$ we use

C11 $$\begin{aligned} \vert J_>({{\widehat{W}}}_1) - J_>({{\widehat{g}}}) \vert \le \frac{1}{2(2\pi )^2} \int _{\{\vert k \vert > \ell _\delta ^{-1}\}} \sup _{w \in [{{\widehat{g}}}_k, {{\widehat{W}}}_1(k)] } \vert \partial _w G_k(w) \vert \cdot \vert {{\widehat{W}}}_1(k) - {{\widehat{g}}}_k \vert \textrm{d}k,\nonumber \\ \end{aligned}$$

with

C12 $$\begin{aligned} \partial _w G = \frac{\rho }{\sqrt{1+ \frac{2 \rho w}{(1-\varepsilon _N) k^2}}} - \rho + \frac{\rho ^2 w}{(1-\varepsilon _N)k^2} . \end{aligned}$$

We use a Taylor expansion of the square root to get

$$\begin{aligned} \vert J_>({{\widehat{W}}}_1) - J_>({{\widehat{g}}}) \vert&\le C \rho ^3 \int _{\vert k \vert > \ell ^{-1}_\delta } \frac{{{\widehat{g}}}_k^2}{k^4} \vert {{\widehat{W}}}_1(k) - {{\widehat{g}}}_k \vert \textrm{d}k. \end{aligned}$$

Since $\vert {{\widehat{W}}}_1(k) - {{\widehat{g}}}_k \vert \le C \delta ^2 K_\ell ^{-1}$ (by (6.24)) and $\int {{\widehat{g}}}_k^2 k^{-2} \textrm{d}k < C \delta$ (see (3.34)) we deduce

C13 $$\begin{aligned} \vert J_>({{\widehat{W}}}_1) - J_>({{\widehat{g}}}) \vert \le C \rho ^2 \delta ^2 K_\ell ^{-1} . \end{aligned}$$

For $k < \ell _\delta ^{-1}$ we start by focusing on the first part of $G_k$,

C14 $$\begin{aligned} F_k(w) = \sqrt{(1-\varepsilon _N)^2 k^4 + 2 (1-\varepsilon _N)\rho w k^2} - (1-\varepsilon _N)k^2 - \rho w. \end{aligned}$$

Since $\vert \partial _w F_k \vert \le C \rho$, we have

C15 $$\begin{aligned} \Big \vert \int _{\{\vert k \vert< \ell _\delta ^{-1}\}} F_k(\widehat{W}_1(k)) - F_k({{\widehat{g}}}_k) \textrm{d}k \Big \vert \le C \rho \int _{\{\vert k \vert < \ell _\delta ^{-1}\}} \vert {{\widehat{W}}}_1(k) - {{\widehat{g}}}_k \vert \textrm{d}k \le C \rho ^2 \delta ^3 K_\ell ^{-1}.\nonumber \\ \end{aligned}$$

Now

$$\begin{aligned} \vert J_<({{\widehat{W}}}_1) - J_<({{\widehat{g}}}) \vert&\le C \Big \vert \int _{\{\vert k \vert< \ell _\delta ^{-1}\}} F_k({{\widehat{W}}}_1(k)) - F_k({{\widehat{g}}}_k) \textrm{d}k \Big \vert \\&\quad + C \Big \vert \int _{\{\vert k \vert< \ell _\delta ^{-1}\}} \rho ^2 \frac{{{\widehat{W}}}_1(k)^2 - {{\widehat{W}}}_1(0)^2}{2(1-\varepsilon _T)k^2} \textrm{d}k \Big \vert \\&\quad + C \Big \vert \int _{\{\vert k \vert < \ell _\delta ^{-1}\}} \rho ^2 \frac{{{\widehat{g}}}_k^2 - {{\widehat{g}}}_0^2}{2(1-\varepsilon _T)k^2} \textrm{d}k \Big \vert \\&\le C \rho ^2 \delta ^3 K_\ell ^{-1} + C \rho ^2 R^2 \delta ^2 \ell _\delta ^{-2} \le C \rho ^2 \delta ^2 K_\ell ^{-1}, \end{aligned}$$

where we used (3.35). Combining this with (C8) and (C13) the lemma is proved. $\square$

### Proposition C.3

There exists a universal constant $C >0$ such that, for any $\varepsilon \in [0,1)$,

$$\begin{aligned}{} & {} \Big \vert I_\varepsilon (k^2, {\widehat{g}}_k) - 4 \pi \rho ^2 \delta \Big ( 1 - \frac{\delta }{Y} + \delta \log \delta + \Big (\frac{1}{2} + 2 \Gamma + \log (\pi )\Big ) \delta \Big ) \Big \vert \\{} & {} \quad \le C \rho ^2 \delta ^3 \big ( \vert \log (\delta ) \vert R^2 \rho + 1 \big ) + C \rho ^2 \delta \varepsilon , \end{aligned}$$

where $I_\varepsilon$ is defined in (C1). In particular when $\delta =\delta _0$ we deduce

$$\begin{aligned} \Big \vert I_\varepsilon (k^2, {\widehat{g}}_k) - 4 \pi \rho ^2 \delta ^2_0 \Big (\frac{1}{2} + 2 \Gamma + \log (\pi ) \Big ) \Big \vert \le C \rho ^2 \delta ^3 \big ( \vert \log (\delta ) \vert R^2 \rho + 1 \big ) + C \rho ^2 \delta \varepsilon . \end{aligned}$$

### Proof

At first we want to replace ${\widehat{g}}_k$ by ${\widehat{g}}_0$ in the integral *I*:

C16 $$\begin{aligned} |I_\varepsilon (k^2, {\widehat{g}}_k)- I_\varepsilon (k^2, {\widehat{g}}_0)|&\le \int _{{\mathbb {R}}^{2}} |F(k^2, {\widehat{g}}_k) - F(k^2, {\widehat{g}}_0)| \textrm{d}k \end{aligned}$$

C17 $$\begin{aligned}&\le \int _{{\mathbb {R}}^{2}} \sup _{g\in [{\widehat{g}}_k, {\widehat{g}}_0]}\left| \partial _g F(k^2, g)\right| \left| {\widehat{g}}_{k} -{\widehat{g}}_0 \right| \textrm{d}k =: I_{\leqslant } + I_{\geqslant }, \end{aligned}$$

where we split for values of |*k*| under or above $(\rho \delta )^{1/2}$. Notice that

C18 $$\begin{aligned} \partial _g F(k^2, {{\widehat{g}}}_k) =\frac{\rho k^{2}}{\sqrt{k^{4}+2\rho {\widehat{g}}_kk^{2} }}-\rho +\frac{\rho ^{2} {\widehat{g}}_k}{k^{2}}. \end{aligned}$$

By a Taylor expansion we can prove that

C19 $$\begin{aligned} I_{\leqslant } \le C \int _{\{|k| \le (\rho \delta )^{1/2}\}}\Big ( R^2 (\rho {\widehat{g}}_0)^{1/2} k^3 + \rho {\widehat{g}}_0k^2 + R^2\big (\rho {\widehat{g}}_0\big )^{2} \Big ) \textrm{d}k \le C R^2 (\rho \delta )^3.\nonumber \\ \end{aligned}$$

In the other case we have, by Taylor expansion of the square root in (C18),

$$\begin{aligned} I_{\geqslant }&\le C\rho \int _{\{|k| \ge (\rho \delta )^{1/2}\}} \frac{(\rho {\widehat{g}}_0)^2}{k^4} |{\widehat{g}}_{k} -{\widehat{g}}_0 | \textrm{d}k \\&\le C \left( \rho {\widehat{g}}_0\right) ^3 \Big ( \int _{\{ (\rho \delta )^{1/2}\le |k| \le (\rho \delta )^{1/2} {\widehat{g}}_0^{-1/2}\}} \frac{R^2}{k^2} \textrm{d}k + \int _{\{|k| \ge (\rho \delta )^{1/2} {\widehat{g}}_0^{-1/2}\}} \frac{\textrm{d}k}{k^4} \Big ) \\&\le C \left( \rho \delta \right) ^{3} R^2 |\log \delta | + C \left( \rho \delta \right) ^{2}\delta . \end{aligned}$$

We deduce that $|I_\varepsilon (k^2, {\widehat{g}}_k)- I_\varepsilon (k^2, {\widehat{g}}_0)| \le C \rho ^2 \delta ^3 (1 + R^2 \rho \log (\delta ))$. Now remains to compute $I_\varepsilon (k^2, {\widehat{g}}_0)$. In this integral we use the new variable $q = k (\rho {\widehat{g}}_0)^{-\frac{1}{2}} (1-\varepsilon )^{\frac{1}{2}}$,

C20 $$\begin{aligned} I_\varepsilon (k^2, {\widehat{g}}_0) = \frac{(\rho {\widehat{g}}_0)^2}{2(2\pi )^2(1-\varepsilon )} \int _{{\mathbb {R}}^2} \Big (\sqrt{q^4 + 2 q^2} - q^2 - 1 + \frac{\mathbb {1}_{\{\vert q \vert > (1-\varepsilon )^{\frac{1}{2}} \ell _\delta ^{-1} (\rho {\widehat{g}}_0)^{- \frac{1}{2}}\}}}{2 q^2} \Big )\textrm{d}q.\nonumber \\ \end{aligned}$$

In term of $c_0 = (1-\varepsilon )^{\frac{1}{2}} \ell _\delta ^{-1} (\rho {\widehat{g}}_0)^{- \frac{1}{2}}$, this integral is explicitly computable and equal to

C21 $$\begin{aligned} I_\varepsilon (k^2, {\widehat{g}}_0) = \frac{(\rho {\widehat{g}}_0)^2}{4 \pi (1-\varepsilon )} \Big ( \frac{1}{8} - \frac{\log 2}{4} + \frac{1}{2} \log (c^{-1}_0) \Big ). \end{aligned}$$

With ${\widehat{g}}_0 = 8 \pi \delta$ and $c_0 = 2 e^{-\Gamma } e^{-\frac{1}{2 \delta }} (1-\varepsilon )^{\frac{1}{2}} {\widehat{g}}_0^{-\frac{1}{2}} (\rho a^2)^{-\frac{1}{2}}$ (see (3.30)), we find

C22 $$\begin{aligned} I_\varepsilon (k^2, {\widehat{g}}_0) = 4 \pi \rho ^2 \delta ^2 \Big ( \frac{1}{\delta } - \frac{1}{Y} + \log \delta + \frac{1}{2} + 2 \Gamma + \log (\pi ) \Big ) (1+ {\mathcal {O}}(\varepsilon )). \end{aligned}$$

$$\square $$

### Remark C.4

With the arbitrary parameter $\delta$ (within the range $\frac{1}{2} Y \le \delta \le 2 Y$), one can deduce from Proposition C.3 that our lower bound on the energy is

C23 $$\begin{aligned} e^{\text {2D}}(\rho ) \ge 4\pi \rho ^2 \delta \Big ( 2 - \frac{\delta }{Y} + \delta \log \delta + \Big ( \frac{1}{2} + 2 \Gamma + \log (\pi ) \Big ) \delta \Big ) - C \rho ^2 Y^{2+\eta }. \end{aligned}$$

However, this lower bound is maximized by $\delta = Y( 1 - Y \vert \log Y \vert + o(Y \vert \log Y\vert ))$, thus leading to the optimal choice $\delta = \delta _0$.

We conclude this section by a general bound on Bogoliubov integrals that is used several times throughout the paper.

### Lemma C.5

For two functions ${\mathcal {A}}, {\mathcal {B}} : {\mathbb {R}}^2 \rightarrow {\mathbb {R}}$ such that

C24 $$\begin{aligned} {\mathcal {A}}(k) \ge \kappa [|k| - K]_+^2 + 2 K_1 \delta , \qquad |{\mathcal {B}}(k)| \le K_2 \delta , \qquad |{\mathcal {B}}(k) - {\mathcal {B}}(0)| \le K_2 R^2 \delta |k|^2,\nonumber \\ \end{aligned}$$

for constants $\kappa > 0, 0< K_2 \le K_1, \ell _{\delta }^{-1} < K$, then there exists $C > 0$ such that

C25 $$\begin{aligned} \int _{{\mathbb {R}}^2} \,&\big ({\mathcal {A}}(k) - \sqrt{{\mathcal {A}}(k)^2 - {\mathcal {B}}(k)^2} \big )\textrm{d}k \nonumber \\&\le \kappa ^{-1}\int _{{\mathbb {R}}^2} \, \frac{{\mathcal {B}}^2(k) - {\mathcal {B}}^2(0)\mathbb {1}_{\{|k|\le \ell _{\delta }^{-1}\}}}{2|k|^2}\textrm{d}k\nonumber \\&\quad + C \frac{K_2^2}{K_1} \delta K^2 + C \kappa ^{-1} K_2^2 \delta ^2 (1 + R^2 \ell ^{-2}_{\delta } ) +C \kappa ^{-1} K_2^2\delta ^2 |\log (2K \ell _{\delta })| \nonumber \\&\quad + C \min \Big ( K_2^4 \delta ^4 \kappa ^{-3} K^{-4}, C \frac{K_2^2 }{K_1^2}\kappa ^{-1} \int _{{\mathbb {R}}^2} \frac{{\mathcal {B}}(k)^2 - {\mathcal {B}}(0)^2 \mathbb {1}_{\{|k| < \ell _{\delta }^{-1}\}}}{|k|^2} \textrm{d}k \Big ). \end{aligned}$$

### Proof

We show that the difference

C26 $$\begin{aligned} \int _{{\mathbb {R}}^2} \, \Big (\big ({\mathcal {A}}(k) - \sqrt{{\mathcal {A}}(k)^2 - {\mathcal {B}}(k)^2} \big ) - \kappa ^{-1} \frac{{\mathcal {B}}^2(k) - {\mathcal {B}}^2(0)\mathbb {1}_{\{|k|\le \ell _{\delta }^{-1}\}}}{2|k|^2}\Big )\textrm{d}k, \end{aligned}$$

is bounded by the desired error terms.

For $|k | \le 2K$ we have that

$$\begin{aligned} \int _{|k|\le 2K} \, \big ({\mathcal {A}}(k) - \sqrt{{\mathcal {A}}(k)^2 - {\mathcal {B}}(k)^2} \big )\textrm{d}k&\le C \int _{\{|k|\le 2K\}} \,\frac{{\mathcal {B}}^2(k)}{{\mathcal {A}}(k)}\textrm{d}k \\&\le C \frac{K_2^2}{K_1} \delta \int _{\{|k|\le 2K\}} \textrm{d}k\,= C \frac{K_2^2}{K_1} \delta K^2, \end{aligned}$$

while for the ${\mathcal {B}}$ part, using the assumption on $|{\mathcal {B}}(k) - {\mathcal {B}}(0)|$,

C27 $$\begin{aligned} \kappa ^{-1} \int _{\{|k|\le \ell _{\delta }^{-1}\}} \frac{|{\mathcal {B}}^2(k) - {\mathcal {B}}^2(0)|}{2|k|^2}\textrm{d}k\le C \kappa ^{-1} K_2^2 R^2 \delta ^2 \int _{\{|k|\le \ell _{\delta }^{-1}\}} \textrm{d}k = C \kappa ^{-1} K_2^2 R^2 \delta ^2 \ell ^{-2}_{\delta },\nonumber \\ \end{aligned}$$

and

C28 $$\begin{aligned} \kappa ^{-1} \int _{\{\ell ^{-1}_{\delta } \le |k| \le 2K\}} d\, \frac{{\mathcal {B}}_k^2}{2|k|^2}\textrm{d}k \le C \kappa ^{-1} K_2^2\delta ^2 |\log (2K \ell _{\delta })|. \end{aligned}$$

For $|k | \ge 2K$ we have, by a Taylor expansion,

C29 $$\begin{aligned} {\mathcal {A}}(k) - \sqrt{{\mathcal {A}}(k)^2 - {\mathcal {B}}(k)^2} \le \frac{1}{2} \frac{{\mathcal {B}}(k)^2}{{\mathcal {A}}(k)} + C \frac{{\mathcal {B}}(k)^4}{{\mathcal {A}}(k)^3}. \end{aligned}$$

For the first term we observe that

C30 $$\begin{aligned} \frac{{\mathcal {B}}(k)^2}{{\mathcal {A}}(k)} \le \kappa ^{-1} \frac{{\mathcal {B}}(k)^2}{(|k|-K)^2} \le \kappa ^{-1} \frac{{\mathcal {B}}(k)^2}{|k|^2} \Big ( 1+ \frac{K}{|k|}\Big ), \end{aligned}$$

giving

$$\begin{aligned} \int _{\{|k| \ge 2 K\}} \, \bigg ( \frac{{\mathcal {B}}(k)^2}{{\mathcal {A}}(k)} - \kappa ^{-1}\frac{{\mathcal {B}}(k)^2}{2|k|^2}\bigg )\textrm{d}k\le C K \kappa ^{-1} \int _{\{|k| \ge 2 K\}} \frac{{\mathcal {B}}(k)^2}{|k|^3}\textrm{d}k \le C \kappa ^{-1} K_2^2\delta ^2, \end{aligned}$$

while for the second one we can bound either

C31 $$\begin{aligned} \int _{\{|k| \ge 2 K\}} \, \frac{{\mathcal {B}}(k)^4}{{\mathcal {A}}(k)^3}\textrm{d}k \le C K_2^4 \delta ^4 \kappa ^{-3}\int _{\{|k| \ge 2 K\}} \frac{\textrm{d}k}{|k|^6} \le C K_2^4 \delta ^4 \kappa ^{-3} K^{-4}, \end{aligned}$$

or as in the following,

$$\begin{aligned} \int _{\{|k| \ge 2 K\}} \, \frac{{\mathcal {B}}(k)^4}{{\mathcal {A}}(k)^3}\textrm{d}k \le \frac{K_2^2 }{K_1^2} \kappa ^{-1}\int _{\{|k| \ge 2 K\}} \, \frac{{\mathcal {B}}(k)^2}{{\mathcal {A}}(k)} \textrm{d}k\le C \frac{K_2^2 }{K_1^2} \kappa ^{-1}\int _{\{|k| \ge 2 K\}} \, \frac{{\mathcal {B}}(k)^2}{|k|^2}\textrm{d}k, \end{aligned}$$

adding and subtracting the term $C \frac{K_2^2 }{K_1^2}\kappa ^{-1} \int _{\{|k|<2K\}} \frac{{\mathcal {B}}(k)^2 - {\mathcal {B}}(0)^2 \mathbb {1}_{\{|k| < \ell _{\delta }^{-1}\}}}{|k|^2}$ we have

C32 $$\begin{aligned} \int _{\{|k| \ge 2 K\}} \, \frac{{\mathcal {B}}(k)^4}{{\mathcal {A}}(k)^3}\textrm{d}k \le C \frac{K_2^2 }{K_1^2} \kappa ^{-1}\int _{{\mathbb {R}}^2} \frac{{\mathcal {B}}(k)^2 - {\mathcal {B}}(0)^2 \mathbb {1}_{\{|k| < \ell _{\delta }^{-1}\}}}{|k|^2} \textrm{d}k. \end{aligned}$$

This finishes the proof of Lemma C.5. $\square$

## Appendix D: A Priori Bounds

In this section, we prove Theorem 7.6. We study a localized problem on a shorter length scale $d\ell$ such that

D1 $$\begin{aligned} d \ell \ll \ell _{\delta } \ll \ell . \end{aligned}$$

where we recall that $\ell _{\delta }$ is the healing length. We are able, in this section, to prove Bose–Einstein condensation in boxes with length scale smaller than the healing length. A key point is that, at this scale, we can use a larger Neumann gap to reabsorb the errors. We will show how the proof of Theorem 7.6 reduces to this localized problem.

We introduce the small box centered at $u \in {\mathbb {R}}^2$ to be

D2 $$\begin{aligned} B_u = \Lambda \cap \bigg \{ d\ell u + \Big [-\frac{d\ell }{2} , \frac{d\ell }{2} \Big ]^2 \bigg \}. \end{aligned}$$

The associated localization functions are

D3 $$\begin{aligned} \chi _{B_u}(x) := \chi \left( \frac{x}{\ell }\right) \chi \left( \frac{x}{d\ell } -u \right) , \end{aligned}$$

where we highlight that

D4 $$\begin{aligned} \iint |\chi _{B_u}|^2 \textrm{d}x \textrm{d}u = \ell ^2. \end{aligned}$$

In order to construct the small box Hamiltonian, we introduce the localized potentials

D5 $$\begin{aligned} W^{\text {s}}(x)&:= \frac{W(x)}{\chi *\chi (x/d\ell )},&w_{B_u}(x,y) := \chi _{B_u} (x) W^s(x-y)\chi _{B_u}(y), \end{aligned}$$

D6 $$\begin{aligned} W_1^{\text {s}}(x)&:= \frac{W_1(x)}{\chi *\chi (x/d\ell )},&w_{1,B_u}(x,y) := \chi _{B_u} (x) W_1^s(x-y)\chi _{B_u}(y), \end{aligned}$$

D7 $$\begin{aligned} W_2^{\text {s}}(x)&:= \frac{W_2(x)}{\chi *\chi (x/d\ell )},&w_{2,B_u}(x,y) := \chi _{B_u} (x) W_2^s(x-y)\chi _{B_u}(y), \end{aligned}$$

where we recall that $W, W_1, W_2$ are localized versions of $v, g, (1+ \omega ) g$, respectively (see formulas (6.19) and (3.4)). Since *v* has support in *B*(0, *R*), we see that $W^{\text {s}}$ is well-defined as $d\ell$ is required to be larger then *R*. Clearly $W^{\text {s}}$ depends on $d\ell$ and thus $\rho _\mu$, but we will not reflect this in our notation.

Similarly to Lemma 6.4, $W_1^{\text {s}}$ satisfies the following inequalities which can be proven in analogous ways considering the length scale $d \ell$ in place of $\ell$

D8 $$\begin{aligned}&\int W_2^{\text {s}} \le 2\int W_1^{\text {s}} \le C \delta , \end{aligned}$$

D9 $$\begin{aligned}&0 \le W_1^{\text {s}}(x) - g(x)\le C g(x) \frac{|x|^2}{(d\ell )^2}, \end{aligned}$$

D10 $$\begin{aligned}&\bigg | \frac{1}{(2\pi )^2} \int _{{\mathbb {R}}^2} \frac{{\widehat{W}}_1^{\text {s}}(k)^2-{\widehat{W}}_1^{\text {s}}(0)^2\mathbb {1}_{\{|k| \le \ell _{\delta }^{-1}\}}}{2 k^2}\textrm{d}k - \widehat{g\omega }(0)\bigg | \le C \frac{R^2\delta }{(d \ell )^2}. \end{aligned}$$

We define furthermore, as operators on $L^2(B_u)$,

D11 $$\begin{aligned} P_{B_u} := \frac{1}{|B_u|} | \mathbb {1}_{B_u} \rangle \langle \mathbb {1}_{B_u}|, \qquad Q_{B_u} := \mathbb {1}_{B_u} - P_{B_u}, \end{aligned}$$

i.e., $P_{B_u}$ is the orthogonal projection in $L^2(B_u)$ onto the constant functions and $Q_{B_u}$ is the projection to the orthogonal complement. We can therefore introduce the number operators as well

D12 $$\begin{aligned} n_{B_u} := \sum _{j=1}^N \mathbb {1}_{B_u,j}, \quad n_{B_u,0} := \sum _{j=1}^N P_{B_u,j}, \quad n_{B_u,+} := \sum _{j=1}^N Q_{B_u,j}, \end{aligned}$$

and the small-box kinetic energy

D13 $$\begin{aligned} {\mathcal {T}}_{B_u} := Q_{B_u}\Big ( \chi _{B_u} \Big [ \sqrt{-\Delta } - \frac{1}{ds\ell } \Big ]^2_{+} \chi _{B_u} + \frac{\varepsilon _T}{2} (1 + \pi ^{-2})\frac{1}{(d\ell )^2} \Big ) Q_{B_u}. \end{aligned}$$

We are now ready to define the localized Hamiltonian ${{\mathcal {H}}}_{B_u}$ which acts on the symmetric Fock space ${{\mathcal {F}}}_s (L^2(B_u))$. It preserves particle number and is given as

D14 $$\begin{aligned} {{\mathcal {H}}}_{B_u}(\rho _{\mu })_{N} := \sum _{i=1}^N (1-\varepsilon _N) {\mathcal {T}}_{B_u}^{(i)} - \rho _{\mu }\sum _{i=1}^N \int w_{1, B_u}(x_i,y)\,\textrm{d}y + \frac{1}{2}\sum _{i \ne j} w_{B_u}(x_i,x_j), \end{aligned}$$

on the *N*-particle sector where $\varepsilon _{N}$ was introduced in Lemma 6.2.

An adaptation to dimension 2 of [28, Theorem 3.10] allows us relate ${\mathcal {H}}_B(\rho _{\mu })$ to the original Hamiltonian in the large box, using the condition (H28). This gives the lower bound

D15 $$\begin{aligned} {\mathcal {H}}_{\Lambda }(\rho _{\mu }) \ge (1-\varepsilon _N) \frac{b}{\ell ^2}\sum _{j=1}^N Q_{\Lambda ,j} + \int _{{\mathbb {R}}^2} \, {\mathcal {H}}_{B_u} (\rho _{\mu })\textrm{d}u. \end{aligned}$$

We would like to restrict the previous integral to boxes that are not too small. Therefore, we identify the following sets of integration, for $\xi \in [0,1]$,

D16 $$\begin{aligned} \Lambda _{\xi } := \Big \{u \in {\mathbb {R}}^2\,\Big |\, \vert \ell d u \vert _{\infty } - \frac{\ell }{2}(d+1) \le - \xi d \ell \Big \}, \end{aligned}$$

underlying the property

D17 $$\begin{aligned} \Lambda _{\xi _1} \subseteq \Lambda _{\xi _2} \quad \text {if}\quad \xi _1 \ge \xi _2, \end{aligned}$$

and we observe that integration outside $\Lambda _0$ is zero because there is no more intersection between the small box and $\Lambda$. The following Lemma guarantees that we can restrict the integration for the potential over set $\Lambda _{1/10}$ (where 1/10 is chosen arbitrarily) and estimate the remaining part by a frame inside $\Lambda _{1/10}$.

### Lemma D.1

For all $x \in \Lambda$ we have the estimate

D18 $$\begin{aligned}{} & {} -\rho _{\mu } \iint \, w_{1,{B_u}}(x,y)\textrm{d}y \textrm{d}u \nonumber \\{} & {} \quad \ge -\rho _{\mu } \int _{\Lambda _{\frac{1}{10}}} \,\int \, w_{1,{B_u}}(x,y) \textrm{d}y \textrm{d}u -3\rho _{\mu } \int _{\Lambda _{\frac{1}{10}}\setminus \Lambda _{\frac{1}{5}}} \int \, w_{1,{B_u}}(x,y)\textrm{d}y \textrm{d}u.\qquad \end{aligned}$$

### Proof

The proof follows the same lines as in [19, Lemma E.1]. We split the domain of integration $\Lambda _{1/10}$ and $\Lambda _0 - \Lambda _{1/10}$ and we estimate the integral over the latter. By the definition of $w_{1,B}$ we have simply to estimate the quantity

D19 $$\begin{aligned} \int _{\Lambda _0 \setminus \Lambda _{1/10}} \, \chi \left( \frac{x}{\ell d} - u\right) \chi \left( \frac{y}{\ell d} -u \right) \textrm{d}u. \end{aligned}$$

We use that $\chi$ is a product of decreasing functions in the variables $u_1, u_2$ and observe that

D20 $$\begin{aligned}{} & {} \max _{\frac{1}{2}(d^{-1}+1) -\frac{(\ell d)^{-1}}{10} \le |u_1| \le \frac{1}{2} (d^{-1}+1)} \chi \left( \frac{x}{\ell d} - u\right) \chi \left( \frac{y}{\ell d} -u \right) \nonumber \\{} & {} \quad \le \min _{\frac{1}{2} (d^{-1} + 1) -\frac{2(d\ell )^{-1}}{10} \le |u_1|\le \frac{1}{2d} +\frac{1}{2} -\frac{(d\ell )^{-1}}{10 }} \chi \left( \frac{x}{\ell d} - u\right) \chi \left( \frac{y}{\ell d} -u \right) , \end{aligned}$$

so that we can estimate the integral over the frame pointwise, getting a factor of 3 due to the presence of the corners. $\square$

Thanks to Lemma D.1 we can write

D21 $$\begin{aligned} {\mathcal {H}}_{\Lambda }(\rho _{\mu }) \ge (1-\varepsilon _N) \frac{b}{\ell ^2}\sum _{j=1}^N Q_{\Lambda ,j} + \int _{\Lambda _{\frac{1}{5}}} \, {\mathcal {H}}_{B_u} ( \rho _{\mu }) \textrm{d}u + \int _{\Lambda _{\frac{1}{10}}\setminus \Lambda _{\frac{1}{5}}} \, {\mathcal {H}}_{B_u} (4 \rho _{\mu })\textrm{d}u,\nonumber \\ \end{aligned}$$

where we dropped the positive part of ${\mathcal {H}}_{B_u}$ in $\Lambda _0\setminus \Lambda _{\frac{1}{10}}$. We are now ready to give lower bounds for kinetic and potential energies in terms of the number of particles. From this the lower bound for the small box Hamiltonian is going to follow in Corollary D.6 below.

In order to prove Theorem 7.6, we provide a lower bound on ${\mathcal {H}}_{B_u}(\rho _\mu )$. For notational simplicity we will remove the index *u*. Lemmas D.2 and D.3 below give first lower bounds on the potential and kinetic energy respectively.

### Lemma D.2

There exists a constant $C>0$ depending only on $\chi$ such that

$$\begin{aligned}{} & {} -\rho _{\mu }\int \, \sum _{j =1}^N w_{1,B}(x,y)\textrm{d}y + \frac{1}{2} \sum _{i \ne j } w_B(x_i, x_j) \nonumber \\{} & {} \qquad \ge A_0 + A_2 + \frac{1}{2} {\mathcal {Q}}_{4}^{\text {ren, s}} - C\delta \Big ( \rho _{\mu } + \frac{n_{0,B}}{|B|}\Big ) n_{+,B}, \end{aligned}$$

with

$$\begin{aligned} A_0&:= \frac{n_{0,B}(n_{0,B} + 1)}{2|B|^2} \iint \, w_{2,B}(x,y)\textrm{d}x \textrm{d}y \\&\qquad - \Big ( \frac{\rho _{\mu } n_{0,B}}{|B|} + \frac{1}{4} \big ( \rho _{\mu }- \frac{n_{0,B}-1}{|B|}\big )^2\Big ) \iint \, w_{1,B}(x,y)\textrm{d}x \textrm{d}y,\\ A_2&:= \frac{1}{2} \sum _{i \ne j} P_i P_j w_{1,B} Q_i Q_j + h.c., \end{aligned}$$

and ${\mathcal {Q}}_4^{\text {ren,s}}$ is the analogue of (7.2), but for the small box *B*.

### Proof

The proof follows from an analogous potential splitting like in Lemma 7.1 and Lemma 7.2 and by the same lines as [19, Lemma E.7]. $\square$

### Lemma D.3

For the kinetic energy on the small box in the *N*’th sector we have

$$\begin{aligned} Q_B&\chi _B \Big [ \sqrt{-\Delta } - \frac{1}{ds\ell } \Big ]^2_{+} \chi _B Q_B + A_2 \\&\ge -\frac{1}{2}\widehat{g\omega }(0) \frac{N(N+1)}{|B|^2} \int \chi _B^2 + {\mathcal {E}}_2(N) + {\mathcal {E}}_4(N) - C \delta \frac{N +1}{|B|} n_{+,B}, \end{aligned}$$

where

D22 $$\begin{aligned} {\mathcal {E}}_2(N)&:= - C \delta \Big ( \frac{R^2}{(d\ell )^2} + \delta |\log (d s K_{\ell })| + \delta ^2\Big ) \frac{N(N+1)}{|B|^2} \int \chi _{B}^2, \end{aligned}$$

D23 $$\begin{aligned} {\mathcal {E}}_4(N)&:= -C \Big ( \delta ^4 (ds\ell )^4 \Big ( \frac{N+1}{|B|}\Big )^3 + \delta (ds\ell )^{-2}\Big ) \frac{N}{|B|} \int \chi _{B}^2. \end{aligned}$$

### Proof

Let us introduce the operators

D24 $$\begin{aligned} d^{\dagger }_p := \frac{1}{|B|^{1/2}} a^{\dagger }(Q_B \chi _B e^{-ipx}) a_0, \end{aligned}$$

where $a_0 = \frac{1}{\ell } a (1)$ and $a,a^{\dagger }$ are the annihilation and creation operators on ${\mathscr {F}}_s(L^2(\Lambda ))$. Further we introduce

D25 $$\begin{aligned} A_1 := \frac{{\widehat{W}}_1^s(0)}{(2\pi )^2} \int _{{\mathbb {R}}^2} \, (d^{\dagger }_p d_p + d^{\dagger }_{-p} d_{-p})\textrm{d}p. \end{aligned}$$

Now using that on the *N*’th sector we have

D26 $$\begin{aligned} \int \, \Big [ \vert p \vert - \frac{1}{ds\ell } \Big ]^2_{+} d^{\dagger }_p d_p \textrm{d}p= \frac{(n_0+1)}{|B|} \sum _{j=1}^N Q_{B,j}\chi _{B}(x_j)\Big [ \sqrt{-\Delta } - \frac{1}{ds\ell } \Big ]^2_{+} \chi _B (x_j) Q_{B,j},\nonumber \\ \end{aligned}$$

and that $n_0 \le N$, we get, adding $A_1$ and $A_2$ to the kinetic energy

D27 $$\begin{aligned} \sum _{j=1}^{N}Q_{B,j}\chi _{B,j} \Big [ \sqrt{-\Delta } - \frac{1}{ds\ell } \Big ]^2_{+} \chi _{B,j} Q_{B,j} + A_1 +A_2 \ge \frac{1}{2(2\pi )^2} \int \,h_p \textrm{d}p, \end{aligned}$$

where

D28 $$\begin{aligned} h_p := {\mathcal {A}}_p (d^{\dagger }_p d_p + d^{\dagger }_{-p} d_{-p} ) + {\mathcal {B}}_p (d^{\dagger }_p d^{\dagger }_{-p} + d_{-p} d_p), \end{aligned}$$

with

D29 $$\begin{aligned} {\mathcal {A}}_p := (1- \varepsilon _N) \frac{|B|}{N+1} \Big [ |p| - \frac{1}{ds\ell }\Big ]^2_+ + 2 {\widehat{W}}_1^s(0), \qquad {\mathcal {B}}_p := {\widehat{W}}_1^s(p). \end{aligned}$$

The additional $A_1$ term is estimated, thanks to (D8), by

D30 $$\begin{aligned} A_1 \le C \delta \frac{n_0+1}{|B|} n_{+,B}, \end{aligned}$$

which contributes to the last term in the result of the lemma. By an application of Theorem B.1 we get the bound

D31 $$\begin{aligned} \frac{1}{2(2\pi )^2} \int \,h_p\textrm{d}p \ge -\frac{1}{2(2\pi )^2} \frac{N}{|B|} \int \chi _B^2\int \, \Big ({\mathcal {A}}_p -\sqrt{{\mathcal {A}}_p^2 - {\mathcal {B}}^2_p}\,\Big )\textrm{d}p, \end{aligned}$$

and therefore we want to bound the latter. We observe that, thanks to (D9), ${{\widehat{W}}}_1^{\text {s}}(0) \ge C \delta$ for a certain $C < 8\pi$. Choosing the parameters

D32 $$\begin{aligned} K = (d s\ell )^{-1}, \quad \kappa = (1-\varepsilon _N)\frac{|B|}{N+1}, \quad K_1 =1, \quad K_2 = C, \end{aligned}$$

we can apply Lemma C.5 to obtain

$$\begin{aligned}&-\frac{1}{2(2\pi )^2} \int \, ({\mathcal {A}}_p -\sqrt{{\mathcal {A}}_p^2 - {\mathcal {B}}^2_p})\textrm{d}p \\&\ge -\frac{N+1}{|B|(1-\varepsilon _N)} \widehat{g\omega }(0) - \frac{N+1}{|B|}\frac{R^2}{(d\ell )^2}\delta + C \delta (ds\ell )^{-2} \\&\quad -C \frac{N+1}{|B|} \delta ^2 \Big (1 + \Big (\frac{R}{ \ell _{\delta }}\Big )^{2} \Big ) -C \frac{N+1}{|B|} \delta ^2 |\log (2d s K_{\ell })| - C \delta ^4 \frac{(N+1)^3}{|B|^3} (ds\ell )^{4}, \end{aligned}$$

where we used that $\varepsilon _N \le 1/2$ and (D10) to approximate the leading term by $\widehat{g\omega }(0)$ getting the second term as an error. Plugging the last estimate in (D31) we get the result with the error terms coherent with the definitions of ${\mathcal {E}}_2(n)$ and ${\mathcal {E}}_4(n).$ $\square$

We will also need the following estimates.

### Lemma D.4

Let $\ell _{\text {min}}$ denote the shortest length of the box *B*, then there exists a constant $C>0$ such that

D33 $$\begin{aligned}&\bigg |\iint \, w_{1,B}(x,y) \textrm{d}x \textrm{d}y- 8\pi \delta \int \chi _B^2 \bigg | \le C \delta \Big (\frac{R}{\ell _{\text {min}}}\Big )^2 \int \chi _B^2, \end{aligned}$$

and

D34 $$\begin{aligned}&\iint \, w_{2,B} (x,y)\textrm{d}x \textrm{d}y \ge \iint w_{1,B} (x,y)\textrm{d}x \textrm{d}y + \widehat{g\omega }(0)\int \chi _B^2 - C \frac{R\delta ^2}{\ell _{\min }^2} \int \chi _B^2. \end{aligned}$$

### Proof

Since $8\pi \delta = \int g$, and thanks to (D9), we can write the inequality

D35 $$\begin{aligned} \bigg |\iint (W^s_1(x) -g(x) )\chi _B^2(y) \textrm{d}x \textrm{d}y \bigg | \le C\delta \Big (\frac{R}{\ell _{\text {min}}} \Big )^2 \int \chi _B^2, \end{aligned}$$

where we used $\ell _{\text {min}}\le d\ell$. By a Taylor expansion for the localization function and the fact that *W* is spherically symmetric and (D8), we have, on the other hand,

D36 $$\begin{aligned} \left| \iint \, w_{1,B}(x,y)\textrm{d}x \textrm{d}y - \int \, W^s_1(x) \textrm{d}x \int \chi _B^2\right|&\le C R^2\Vert \nabla ^2\chi _B\Vert _{\infty } \int W_1^s (x) \textrm{d}x \int \chi _B \nonumber \\&\le C \Big ( \frac{R}{\ell _{\text {min}}}\Big )^2 \delta \int \chi _B^2, \end{aligned}$$

and where we used that $|B|^{-1} (\int \chi _B)^2 \le \int \chi _B^2$ and the bound (F6).

Then inequality (D33) follows by (D35) and (D36). The inequality (D34) follows from a very similar argument. $\square$

Combining the results of Lemma D.2 and D.3, we deduce that the Hamiltonian on the small box has the following lower bound, which is coherent with the main order of the energy expansion.

### Theorem D.5

Assume the conditions from “Appendix H”, then for any box *B* we have the following bound on the *N*’th sector

D37 $$\begin{aligned} {\mathcal {H}}_{B} (\rho _{\mu })|_N \ge \Big ( \frac{1}{4} \Big ( \rho _{\mu } - \frac{N}{|B|}\Big )^2- \frac{1}{2}\rho _{\mu }^2 \Big ) \iint w_{1,B} + \frac{1}{2} {\mathcal {Q}}_4^{\text {ren,s}} + {\mathcal {E}}_2(N) + {\mathcal {E}}_4(N), \nonumber \\ \end{aligned}$$

with ${\mathcal {E}}_2$ and ${\mathcal {E}}_4$ defined in (D22).

### Proof

The combination of Lemmas D.2, D.3 gives

$$\begin{aligned} {\mathcal {H}}_B (\rho _{\mu }) \ge&\,\sum _{j=1}^n Q_{B,j}\Big ( \frac{\varepsilon _T}{2} (1 + \pi ^{-2})\frac{1}{(d\ell )^2} \Big ) Q_{B,j} +A_0 -\frac{1}{2}\widehat{g\omega }(0) \frac{N(N+1)}{|B|^2} \int \chi _B^2 \\&+ \frac{1}{2} {\mathcal {Q}}_4^{\text {ren,s}}+{\mathcal {E}}_2(N) + {\mathcal {E}}_4(N) - C \delta \rho _{\mu } n_{+,B}. \end{aligned}$$

We observe that we can choose a constant $C'>0$ such that

D38 $$\begin{aligned} \sum _{j=1}^N Q_{B,j}\Big ( \frac{\varepsilon _T}{2} \frac{1}{(d\ell )^2} \Big ) \ge C' \rho _{\mu } \delta n_{+,B}, \end{aligned}$$

where we used (H8) and, choosing the right $C'$, we can cancel the last term with $n_{+,B}$ for a lower bound. The same can be said for the errors produced by replacing $n_0 = N- n_{+}$ by *N*. By Lemma D.4 we have

$$\begin{aligned} A_0&-\frac{1}{2}\widehat{g\omega }(0) \frac{N (N+1)}{|B|} \int \chi _B^2 \\&\ge \Big ( -\frac{N^2}{2|B|^2} - \Big ( \rho _{\mu }\frac{N}{|B|} + \frac{1}{4} \Big ( \rho _{\mu } - \frac{N}{|B|}\Big )^2 \Big ) \Big ) \iint w_{1,B} - C \frac{N^2}{|B|^2}\frac{R\delta ^2}{\ell _{\min }^2} \int \chi _B^2 \\&\ge \Big ( \frac{1}{4} \Big ( \rho _{\mu } - \frac{N}{|B|}\Big )^2- \frac{1}{2}\rho _{\mu }^2 \Big ) \iint w_{1,B} - C \frac{N^2}{|B|^2}\frac{R\delta ^2}{\ell _{\min }^2} \int \chi _B^2, \end{aligned}$$

and this gives the result since the last term can be reabsorbed in the ${\mathcal {E}}_2$ term. $\square$

We deduce the following corollary.

### Corollary D.6

Assume *B* is a small box with shortest side length $\ell _{\text {min}} \ge \frac{d\ell }{10 }$ and that the conditions of “Appendix H” hold true. Then we have the following lower bound

$$\begin{aligned} {\mathcal {H}}_{B}(\rho _{\mu }) \ge - \frac{1}{2}\rho _{\mu }^2 \iint \, w_{1,B}(x,y)\textrm{d}x \textrm{d}y - C \rho _{\mu }^2 \delta ^2 (d s K_{\ell })^{-2} \int \chi _{B}^2 -C \rho _{\mu } \delta \frac{1}{|B|} \int \chi _{B}^2. \end{aligned}$$

### Proof

We split the particles in *m* subsets of $n'$ particles and a remaining group of $n''$, with $n''< n' < n$. If we ignore the positive interactions between the subsets, and denoting by $e_B(n, \rho _{\mu })$ the ground state energy of ${\mathcal {H}}_B(\rho _{\mu })$ restricted to states with *n* particles in the box *B*, then

D39 $$\begin{aligned} e_B(n, \rho _{\mu }) \ge m e_B(n', \rho _{\mu }) + e_B(n'' , \rho _{\mu }). \end{aligned}$$

From formula (D37) in Theorem D.5 applied for $n'$ in place of *n* and, choosing $n' = 3 \rho _{\mu } |B|$, we see that the first term becomes, thanks to Lemma D.4

D40 $$\begin{aligned} \frac{1}{2} \rho ^2_{\mu } \iint w_{1,B} \ge 4 \pi \rho ^2_{\mu } \delta \Big ( 1- C \Big (\frac{R}{\ell _{\text {min}}}\Big )^2\Big ) \int \chi _B^2. \end{aligned}$$

From the following controls on the error terms

D41 $$\begin{aligned} {\mathcal {E}}_2(n')&\le C \rho ^2_{\mu } \delta ^2 (d K_\ell )^{-2} \int \chi _{B}^2, \end{aligned}$$

D42 $$\begin{aligned} {\mathcal {E}}_4(n')&\le C \rho ^2_{\mu } \delta ^2 (d s K_{\ell })^{-2} \int \chi _B^2, \end{aligned}$$

D43 $$\begin{aligned} C \rho _{\mu }^2\delta \Big (\frac{R}{\ell _{\text {min}}}\Big )^2\int \chi _B^2&\le C\rho _{\mu }^2 \delta \frac{R^2}{(d\ell )^2}\int \chi _B^2 \le C \rho _{\mu }^2 \delta ^2 (d K_{\ell })^{-2} \int \chi _B^2, \end{aligned}$$

we see that the first term is the leading term of the energy. Since it is clearly positive, we obtain that with the aforementioned choice of $n'$, we have $e_{B}(n', \rho _{\mu }) \ge 0$ and, then, using the previous equality, we can state that

D44 $$\begin{aligned} e_B(n, \rho _{\mu }) \ge e_B(n'', \rho _{\mu }). \end{aligned}$$

The Corollary follows using again Theorem D.5 with $n''$ in place of *n* to obtain the lower bound and using (D41) and (D42) for $n''$ to control the errors, using that $s^{-1}\gg 1$ to obtain one of the error terms in the statement. A further error term of order

$$\begin{aligned}C \rho _{\mu } \delta \frac{1}{|B|} \int \chi _B^2,\end{aligned}$$

is created by the substitutions of the terms $n'' \pm 1$ by $n''$. $\square$

We are finally ready to use the lower bound on the small box Hamiltonian to obtain a bound on the number of excited particles in the large box, result stated in the Theorem below. By an abuse notation, from now on, the operators $n, n_+, n_0$ start again to denote the number operators in the large box.

### Theorem D.7

We have the following lower bound for the large box Hamiltonian

D45 $$\begin{aligned} {\mathcal {H}}_{\Lambda }(\rho _{\mu }) \ge -4 \pi \rho _{\mu }^2 \ell ^2 Y \Big (1 - \frac{1}{2} Y |\log Y| \Big ) + \frac{b}{2 \ell ^2} n_+, \end{aligned}$$

and if there exists a normalized $\Psi \in {\mathscr {F}}_s(L^2(\Lambda ))$ with *n* particles in $\Lambda$ such that (7.40) holds:

D46 $$\begin{aligned} \langle {\mathcal {H}}_{\Lambda }(\rho _{\mu })\rangle _{\Psi } \le -4 \pi \rho _{\mu }^2\ell ^2 Y (1- C K_B^2 Y|\log Y|), \end{aligned}$$

then the bound (7.41) for the number of excitations holds:

D47 $$\begin{aligned} \langle n_+\rangle _{\Psi } \le C n K_B^2 K_{\ell }^2 Y |\log Y|. \end{aligned}$$

### Proof

We study the integration over $\Lambda _{1/10} \setminus \Lambda _{1/5}$ from formula (D21). By [18, (C.6)] we have $|\chi _{B_u}| \le C (\ell _{\text {min}} \ell ^{-1})^{M}$ and then

D48 $$\begin{aligned} \int _{\Lambda _{1/10}-\Lambda _{1/5}} \int \chi _{B_u}(x)^2 \textrm{d}x \textrm{d}u \le C \Big ( \frac{\ell _{\text {min}}}{ \ell } \Big )^{2M} (\ell d )^2 d^{-2} \le C \Big ( \frac{\ell _{\text {min}} }{\ell } \Big )^{2M} \ell ^2. \end{aligned}$$

By the joint action of Corollary D.6 and Lemma D.4 we get

D49 $$\begin{aligned} {\mathcal {H}}_{B_u} (4\rho _{\mu }) \ge -C \rho _{\mu }^2 \delta \int \chi _{B_u}^2 - C \rho _{\mu } \delta \Big ( \rho _{\mu }\delta (dsK_{\ell })^{-2} + \frac{1}{|B_u|} \Big )\int \chi _{B_u}^2, \end{aligned}$$

and therefore, using (D48) and that $|B|= d^2 \ell ^2$ we have

D50 $$\begin{aligned} \int _{\Lambda _{1/10} \setminus \Lambda _{1/5}} {\mathcal {H}}_{B_u} (4\rho _{\mu }) \textrm{d}u \ge - C \rho _{\mu }^2\ell ^2 \delta (1 + \delta (dsK_{\ell })^{-2} )\Big ( \frac{\ell _{\text {min}}}{\ell }\Big )^{2M} - C \rho _{\mu } \delta d^{-2},\qquad \end{aligned}$$

Using the definition of $\ell _{\text {min}} = d\ell /10$ and the relations between the parameters (H12) we get

D51 $$\begin{aligned} \Big ( \frac{\ell _{\text {min}}}{\ell }\Big )^{2M} \le d^{2M} \le \delta ,\qquad \rho _{\mu } \delta d^{-2} \le \rho _{\mu }^2 \ell ^2 \delta ^2 (K_{\ell } d)^{-2} \le \rho _{\mu }^2 \ell ^2 \delta ^2 K_B^2, \qquad \end{aligned}$$

which makes the integral coherent with the statement of the Theorem using the expansion of $\delta$,

D52 $$\begin{aligned} \delta \simeq Y - Y^2 |\log Y| + {\mathcal {O}}(Y^3 \vert \log Y \vert ^2). \end{aligned}$$

For the remaining integral in formula (D21) we use Corollary D.6 and Lemma D.4 to get

$$\begin{aligned} \int _{\Lambda _{1/10}}&{\mathcal {H}}_{B_u} (\rho _{\mu }) \textrm{d}u \\ \ge&-\int _{\Lambda _{1/10}} \left[ \iint \textrm{d}x\textrm{d}y \, \frac{1}{2}\rho _{\mu }^2 w_{1,B_u}(x,y) + C \rho _{\mu } \delta \Big ( \rho _{\mu }\delta (dsK_{\ell })^{-2} + \frac{1}{|B_u|} \Big )\int \chi _{B_u}^2\right] \textrm{d}u\\ \ge&-4\pi \rho _{\mu }^2 \ell ^2 \delta - C \rho _{\mu }^2 \ell ^2 \delta ^2 K_B^2, \end{aligned}$$

where we used (H3), (H12) and

$$\begin{aligned} \iiint \, w_{1,B_u} (x,y) \textrm{d}x \textrm{d}y\textrm{d}u = 8 \pi \delta \ell ^2, \qquad \iint \chi _{B_u}(x)^2\textrm{d}u \textrm{d}x = \ell ^2. \end{aligned}$$

Collecting the previous estimates, together with (D21) and the fact that $\varepsilon _N \le \frac{1}{2}$, we finally get (D45), using the expansion (D52) of $\delta$.

The proof of the bound on $n_+$ is proven noting that, joining together the a priori bound (7.40) with the obtained lower bound we get

D53 $$\begin{aligned} \frac{b}{2 \ell ^2} \langle n_+ \rangle _{\Psi } \le C K_B^2\rho _{\mu } \ell ^2 Y^2 |\log Y|, \end{aligned}$$

and conclude recalling that $\ell = \rho _{\mu }^{-1/2}Y^{-1/2} K_{\ell }$. $\square$

We follow now a similar strategy to obtain a lower bound for the large box Hamiltonian and get an a priori bound on the number of particles and a control on ${\mathcal {Q}}_4^{\text {ren}}$ in the large box.

### Corollary D.8

If there exists a *n*-particles state $\Psi \in {\mathscr {F}}_s(L^2(\Lambda ))$ such that (7.40) holds, then the a priori bounds on *n* and ${\mathcal {Q}}_4^{\text {ren}}$ hold:

D54 $$\begin{aligned} \left| \rho _{\mu } - \frac{n}{\ell ^2}\right| \le C K_B K_{\ell } \rho _{\mu } Y^{1/2} |\log Y|^{1/2} ,\qquad \langle {\mathcal {Q}}_4^{\text {ren}}\rangle _{\Psi } \le C K_B^2 K_{\ell }^2\rho _{\mu }^2 \ell ^2 Y^2 |\log Y|.\nonumber \\ \end{aligned}$$

### Proof

We observe that we have the following lower bound, reproducing analogous estimates for potential and kinetic energies from Lemmas D.2 and D.3 but adapted to the large box $\Lambda$ (for details, see [19, Appendix E.2]), where we estimate the $n_+$ contributions thanks to Theorem D.7,

D55 $$\begin{aligned} \langle {\mathcal {H}}_{\Lambda } (\rho _{\mu }) \rangle _\Psi \ge \frac{1}{2} \langle {\mathcal {Q}}_4^{\text {ren}}\rangle _\Psi - 4 \pi \rho ^2_{\mu } \ell ^2 \delta + 2 \pi \Big (\rho _{\mu } - \frac{n}{\ell ^2} \Big )^2\ell ^2 \delta - CK_B^2 K_{\ell }^2 \rho _{\mu }^2 \ell ^2\delta ^2. \nonumber \\ \end{aligned}$$

By the assumption, the expansion of $\delta$ in terms of *Y* and (D55) we get

D56 $$\begin{aligned} \Big (\frac{n}{\ell ^2} - \rho _{\mu } \Big )^2\ell ^2 \delta + \langle {\mathcal {Q}}_4^{\text {ren}} \rangle _\Psi \le C K_B^2 K_{\ell }^2\rho _{\mu }^2 \ell ^2 Y^2 |\log Y|, \end{aligned}$$

which implies the desired bounds. $\square$

## Appendix E: Technical Estimates for Off-Diagonal Excitation Terms

We give here a proof of Lemma 7.9, bounding the terms $d_1^L$ and $d_2^L$ defined in (7.48) and (7.49). We are going to use the following dimension independent estimates which are proven in [19, Corollary F.6] in order to prove the technical lemma below. There exists $C>0$, such that, for any $\varphi \in \textrm{Ran}{\overline{Q}}_{H}$,

E1 $$\begin{aligned} \Vert \Delta (\chi _{\Lambda }\varphi )\Vert \le C \varepsilon _N^{-1/2} \frac{\widetilde{K}_H^2}{\ell ^2}, \qquad \Vert \Delta ^{{\mathcal {N}}} \varphi \Vert \le C \varepsilon _N^{-1} \frac{\widetilde{K}_H^2}{\ell ^2}. \end{aligned}$$

### Lemma E.1

If we assume the relations between the parameters in “Appendix H”, then there exists $C>0$ such that

E2 $$\begin{aligned} \Vert {\overline{Q}}_{H,x} w(x,y) {\overline{Q}}_{H,x}\Vert \le C \varepsilon _N^{-1/2} \frac{\widetilde{K}_H^2}{\ell ^2} \Vert v\Vert _1. \end{aligned}$$

### Proof

The proof is an adaptation to 2 dimensions of [19, Lemma 5.3]. Let $\varphi \in \textrm{Ran}{\overline{Q}}_{H,x}$ with $\Vert \varphi \Vert _2 = 1$, then

E3 $$\begin{aligned} \Vert {\overline{Q}}_{H,x} w(x,y) {\overline{Q}}_{H,x} \varphi \Vert \le I_1 + I_2, \end{aligned}$$

where

$$\begin{aligned} I_1&= \int _{{\mathbb {R}}^2} \, \chi _{\Lambda }(x)^2 |\varphi (x)|^2 v(x-y)\textrm{d}x,\\ I_2&= \int _{{\mathbb {R}}^2} \, \chi _{\Lambda }(x)\, |\chi _{\Lambda }(x) - \chi _{\Lambda }(y)| \,|\varphi (x)|^2 v(x-y)\textrm{d}x. \end{aligned}$$

We use the technical Lemma E.2 below to get

E4 $$\begin{aligned} |I_1| \le \Vert \chi _{\Lambda } \varphi \Vert ^2_{\infty }\Vert v\Vert _1 \le C \Vert v\Vert _1 \Vert \chi _{\Lambda }\varphi \Vert \Vert \Delta \chi _{\Lambda } \varphi \Vert \le C\frac{\widetilde{K}_H^2}{\ell ^2} \varepsilon _N^{-1/2} \Vert v\Vert _1, \end{aligned}$$

by (E1) and (E6) and

$$\begin{aligned} |I_2|&\le C\frac{R}{\ell } \Vert \chi _{\Lambda } \varphi \Vert _{\infty } \Vert \varphi \Vert _{\infty }\Vert v\Vert _1 \le C \frac{R}{\ell } \Vert \Delta (\chi _{\Lambda }\varphi ) \Vert ^{1/2} \Vert \Delta ^{{\mathcal {N}}} \varphi \Vert ^{1/2}_{L^2\big (\left[ -\frac{L}{2},\frac{L}{2} \right] ^2\big )} \Vert v\Vert _1 \\ {}&\le C\varepsilon _N^{-1/2} \Big ( \frac{R}{\ell }\varepsilon _N^{-1/4} \Big ) \frac{\widetilde{K}_H^2}{\ell ^2}\Vert v\Vert _1 \le C \varepsilon _N^{-1/2} \frac{\widetilde{K}_H^2}{\ell ^2}\Vert v\Vert _1, \end{aligned}$$

by a Taylor expansion for the localization function, (E5) and (E6) for $\varphi$ and $\chi _{\Lambda }\varphi$, respectively, (E1) and the choice of the parameters in (H11), (H3), (H14) and this concludes the proof. $\square$

In the proof of Lemma E.1 we used the following result.

### Lemma E.2

Let $$-\Delta ^{{\mathcal {N}}}$$ denote the Neumann Laplacian on $$[-\frac{L}{2}, \frac{L}{2}]^2$$. There exists $$C>0 $$ such that, for all $$f \in {\mathcal {D}}(-\Delta ^{{\mathcal {N}}})$$ such that $$\int _{[-\frac{L}{2},\frac{L}{2}]^2}f(x) \textrm{d}x = 0$$, we have

E5 $$\begin{aligned} \Vert f\Vert _{\infty } \le C \Vert f\Vert _{L^2([-\frac{L}{2}, \frac{L}{2}]^2)}^{1/2} \Vert -\Delta ^{{\mathcal {N}}}f\Vert _{L^2([-\frac{L}{2}, \frac{L}{2}]^2)}^{1/2}. \end{aligned}$$

Also, for all $f \in H^2({\mathbb {R}}^2)$,

E6 $$\begin{aligned} \Vert f\Vert _{\infty } \le C \Vert f\Vert ^{1/2}\Vert \Delta f\Vert ^{1/2}. \end{aligned}$$

### Proof

Let us prove the last inequality, the first one being proven by an adaptation for the box (essentially the only difference is to replace sums by integrals). We use a scaling argument defining $f_{\lambda }(x) := f(\lambda x),$ for $x \in {\mathbb {R}}^2$. Given an $f \in H^2({\mathbb {R}}^2)$, it is clearly possible to choose $\lambda$ such that $\Vert f_{\lambda }\Vert = \Vert \Delta f_{\lambda } \Vert$. Now, for the given $\lambda$, we have

$$\begin{aligned} \Vert f_{\lambda }\Vert ^2_{\infty } \le \frac{1}{(2\pi )^2} \Big ( \int _{{\mathbb {R}}^2} \, |{\hat{f}}_{\lambda }(p)|\textrm{d}p\Big )^2 \le C \int _{{\mathbb {R}}^2} \, (1+|p|^4)\, |{\hat{f}}_{\lambda }(p)|^2\textrm{d}p = C \Vert \Delta f_{\lambda }\Vert ^2, \end{aligned}$$

where we multiplied and divided by $(1+|p|^4)^{1/2}$ and used the Cauchy–Schwarz inequality and the choice of $\lambda$. Applied to $f_{\lambda }$ with the $\lambda$ chosen above the previous inequality becomes

E7 $$\begin{aligned} \Vert f\Vert _{\infty }^2 = \Vert f_{\lambda }\Vert _{\infty }^{2} \le C \Vert \Delta f_{\lambda }\Vert ^2 = C \Vert f_{\lambda }\Vert \Vert \Delta f_{\lambda }\Vert = C \Vert f\Vert \Vert \Delta f\Vert , \end{aligned}$$

where in the last equality we used the scaling properties of the dilatation in $\lambda$ w.r.t. the $L^2$ norm. $\square$

### Proof of Lemma 7.9

Let $\Psi \in {\mathscr {F}}_s(L^2(\Lambda ))$ be satisfying the assumptions of Lemma 7.9. Our goal is to prove the following estimate

E8 $$\begin{aligned}{} & {} \langle (d_{1}^L + d_2^L)\rangle _{{\widetilde{\Psi }}} \nonumber \\{} & {} \quad \le \rho _{\mu } \Vert v\Vert _1 \big ( \langle n_+ \rangle _{{\widetilde{\Psi }}} + n^{1/2} \langle n_+^L\rangle _{{\widetilde{\Psi }}}^{1/2} + \langle (n_+^L)^2\rangle _{{\widetilde{\Psi }}}^{1/2} \varepsilon _N^{-1/4} \widetilde{K}_H + \langle n_+^L \widetilde{n}_+^H\rangle _{{\widetilde{\Psi }}} \varepsilon _N^{-1/4} \widetilde{K}_H \big . \nonumber \\{} & {} \quad + \left. \big ( \langle \widetilde{n}_+^H n_+^L \rangle _{{\widetilde{\Psi }}}^{1/2} \langle (n_+^L)^2 \rangle _{{\widetilde{\Psi }}}^{1/2} + \langle \widetilde{n}_+^H n_+^L \rangle _{{\widetilde{\Psi }}} \big )n^{-1} \varepsilon _N^{-1/2} \widetilde{K}_H^2 \right) + C \langle {\mathcal {Q}}_4^{\text {ren}}\rangle _{{\widetilde{\Psi }}}. \end{aligned}$$

We split the $d_{j}^L$ in several terms multiplying out the parentheses in (7.48) and (7.49). All these terms we treat individually using Cauchy–Schwarz inequalities. Similar bounds have been carried out in [19]. Here we just bound some representative examples to illustrate the procedure and the role played by Lemma E.1.

Let us start using the Cauchy–Schwarz inequality for any $\varepsilon >0$ to get

$$\begin{aligned} \Big |\Big \langle -\rho _{\mu } \sum _{i} P_i \int \textrm{d}y \, w_1(x_i,y)\; {\overline{Q}}_{H,i} + h.c. \Big \rangle _{\Psi }\Big | \le \frac{n}{\ell ^2} \Vert w_1\Vert _1 \big (\varepsilon n + \varepsilon ^{-1} \langle n_+^L\rangle _{\Psi }\big ), \end{aligned}$$

observing that $\Vert w_1\Vert _1 \le C\delta$ and choosing $\varepsilon = \langle n_+^L\rangle _{\Psi }^{1/2} n^{-1/2}$, we obtain the desired quantity.

For the following term, for any $\varepsilon >0$, we use the Cauchy–Schwarz inequality and Lemma E.1,

$$\begin{aligned} \Big |\Big \langle \sum _{i,j} P_i {\overline{Q}}_{H,j} w {\overline{Q}}_{H,i} {\overline{Q}}_{H,j}\Big \rangle _{\Psi }\Big |&\le \varepsilon \frac{n}{\ell ^2} \Vert w\Vert _1\langle n^L_+ \rangle _{\Psi } + \varepsilon ^{-1} \Vert {\overline{Q}}_H w{\overline{Q}}_H\Vert \sum _{i \ne j} \langle {\overline{Q}}_{H,i} {\overline{Q}}_{H,j} \rangle _{\Psi }\\&\le \frac{\Vert w\Vert _1}{\ell ^2}( \varepsilon n^2 + \varepsilon ^{-1} \varepsilon _N^{-1/2} \widetilde{K}_H^2 \langle (n_+^L)^2 \rangle _{\Psi }) \end{aligned}$$

where we used that $n_+^L \le n_+$. Choosing $\varepsilon = \varepsilon _N^{-1/4} \widetilde{K}_H \langle (n_+^L)^2 \rangle _{\Psi }^{1/2} n^{-1}$, we obtain

E9 $$\begin{aligned} \Big |\Big \langle \sum _{i,j} P_i {\overline{Q}}_{H,j} w {\overline{Q}}_{H,i} {\overline{Q}}_{H,j}\Big \rangle _{\Psi }\Big | \le n\frac{\langle (n_+^L)^2\rangle _{\Psi }^{1/2}}{\ell ^2} \varepsilon _N^{-1/4} \widetilde{K}_H \Vert w\Vert _1. \end{aligned}$$

For the next term we want to apply a Cauchy–Schwarz inequality to reobtain a $Q_4^{\text {ren}}$ term. In order to do that we are going to complete the $Q_H$ to a $Q= Q_H + {\overline{Q}}_H$.

$$\begin{aligned}&\Big |\Big \langle \sum _{i \ne j} {\overline{Q}}_{H,i} P_j w Q_{H,i} Q_{H,j} + h.c.\Big \rangle _{\Psi } \Big |\\&\quad \le \Big |\Big \langle \sum _{i \ne j} {\overline{Q}}_{H,i} P_j w Q_i Q_j \Big \rangle _{\Psi } + h.c. \Big | \\&\qquad + \Big |\Big \langle \sum _{i \ne j} {\overline{Q}}_{H,i} P_j w (Q_{H,i} {\overline{Q}}_j + {\overline{Q}}_{H,i} Q_{H,j}) \Big \rangle _{\Psi } + h.c. \Big | \\&\qquad + \Big |\Big \langle \sum _{i,j} P_i {\overline{Q}}_{H,j} w {\overline{Q}}_{H,i} {\overline{Q}}_{H,j} \Big \rangle _{\Psi }\Big |. \end{aligned}$$

The second term and the third terms can be estimated in the same manner as above, so let us focus on completing the first term in order to obtain the $Q_4$.

E10 $$\begin{aligned} \Big |\Big \langle&\sum _{i \ne j} {\overline{Q}}_{H,i} P_j w Q_i Q_j \Big \rangle _{\Psi } + h.c. \Big | \end{aligned}$$

E11 $$\begin{aligned}&\le \Big |\Big \langle \sum _{i \ne j} {\overline{Q}}_{H,i} P_j w (Q_i Q_j + \omega (P_i P_j + P_i Q_j + Q_i P_j)) \Big \rangle _{\Psi } + h.c. \Big | \end{aligned}$$

E12 $$\begin{aligned}&\quad + \Big |\Big \langle \sum _{i \ne j} {\overline{Q}}_{H,i} P_j w \omega ( P_i Q_j + Q_i P_j)) \Big \rangle _{\Psi } + h.c. \Big | \end{aligned}$$

E13 $$\begin{aligned}&\quad + \Big |\Big \langle \sum _{i \ne j} {\overline{Q}}_{H,i} P_j w \omega P_i P_j) \Big \rangle _{\Psi } + h.c. \Big |. \end{aligned}$$

The second and the third terms are treated as above, using that $0 \le \omega \le 1$ on the support of *w*. By a Cauchy–Schwarz inequality on the first term we get

E14 $$\begin{aligned} (E11) \le \langle {\mathcal {Q}}_4^{\text {ren}}\rangle _{\Psi } + C \frac{n}{\ell ^2} \Vert w\Vert _1 \langle n_+\rangle _{\Psi }. \end{aligned}$$

Collecting the previous estimates including the ones not explicitly treated, we obtain (E8).

Bounding $n_+^L \le \widetilde{{\mathcal {M}}}$ in (E8) where it appears for higher moments than 1, using that $\widetilde{n}_+^H \le n$ and that $\varepsilon _N^{-1/4} \widetilde{K}_H \ge 1$ by (H15) gives the result. This finishes the proof of Lemma 7.9. $\square$

## Appendix F: Properties of the Localization Function

We collect here the definition and some important properties of the localization function that are used throughout the paper.

We define

F1 $$\begin{aligned} \chi (x) := C_M (\zeta _1(x_1) \zeta _2(x_2))^{M+2}, \end{aligned}$$

where

F2 $$\begin{aligned} \zeta (y) := {\left\{ \begin{array}{ll} \cos (\pi y), &{}|y| \le 1/2,\\ 0, &{}|y| >1/2, \end{array}\right. } \end{aligned}$$

where $M \in {\mathbb {N}}$ is chosen even and large enough. The normalization constant $C_M>0$ is chosen in order to obtain $\Vert \chi \Vert _2 = 1$. We have $0 \le \chi \in C^M({\mathbb {R}}^2)$. We also define $\chi _\Lambda (x) = \chi (x/\ell )$.

### Lemma F.1

Let $\chi$ be the localization fuction defined above and let $M \in 2 {\mathbb {N}}$. Then, for all $k \in {\mathbb {R}}^2$,

F3 $$\begin{aligned} |{\widehat{\chi }}(k)| \le \frac{C_{\chi }}{(1+ |k|^2)^{M/2}}, \end{aligned}$$

where $C_{\chi } = \int |(1-\Delta )^{M/2}\chi |$. If, furthermore, $|k| \ge \frac{1}{2} K_K \ell ^{-1}$,

F4 $$\begin{aligned} |\widehat{\chi _{\Lambda }}(k)| = \ell ^2 |{\widehat{\chi }}(k\ell )| \le C \ell ^2 K_{H}^{-M}. \end{aligned}$$

An important property for the localization function $\chi _{B_u}$, $u \in {\mathbb {R}}^2$, on the small boxes, namely

F5 $$\begin{aligned} \chi _{B_u}(x) := \chi _{\Lambda }(x) \chi \Big ( \frac{x}{d\ell }-u\Big ), \end{aligned}$$

which is used in “Appendix D”, is the following bound

F6 $$\begin{aligned} \Vert \nabla ^2\chi _{B_u}\Vert _{\infty } \le C_M \frac{1}{|B_u|\ell _{\min }^2} \int \chi _{B_u}, \end{aligned}$$

which is taken from [18, Appendix C]. Here it is key the fact that we do not consider a smooth function but we require $\chi$ to have a finite degree of regularity measured by the parameter *M*.

## Appendix G: Comparing Riemann Sums and Integrals

We will show in this section that we could approximate integrals on ${\mathbb {R}}^2$ by Riemann sums when it was needed in (4.13) to prove the upper bound. Recall that the assumptions of Theorem 4.1 were

G1 $$\begin{aligned} R \le \rho ^{-1/2} Y^{1/2}, \qquad L_{\beta } = \rho ^{-1/2} Y^{-\beta }. \end{aligned}$$

We divide ${\mathbb {R}}^2$ into small squares $\square _p$ of size $\frac{2\pi }{L}$ centered at $p \in \Lambda _L^* = \frac{2\pi }{L} {\mathbb {Z}}^2$. Then, clearly

G2 $$\begin{aligned} \Big \vert \frac{4 \pi ^2}{ L^2} \sum _{p \in \Lambda _L^*} f(p) - \int _{{\mathbb {R}}^2} f(k) \textrm{d}k \Big \vert&\le \frac{C}{L^3}\sum _{p \in \Lambda _L^*} \sup _{\square _p} \vert \nabla f \vert . \end{aligned}$$

We consider the functions present in the two sums of (4.13). With $\alpha _p$ and $\gamma _p$ given in (4.6) the first term is

G3 $$\begin{aligned} f(p)&=p^{2}+\rho _0{\widehat{g}}_{p}-\sqrt{p^{4}+2\rho _0{\widehat{g}}_{p}p^{2}}+\rho _0 \big ({\widehat{v}}_{p}-{\widehat{g}}_{p}\big )\big (\gamma _{p}+\alpha _{p}\big ) \nonumber \\&=p^{2}+\rho _0{\widehat{g}}_{p}-\sqrt{p^{4}+2\rho _0{\widehat{g}}_{p}p^{2}}+\rho _0 ( {\widehat{v}}_{p}-{\widehat{g}}_{p} )\Big (\frac{p^{2}}{2\sqrt{p^{4}+2\rho _0{\widehat{g}}_{p}p^{2}}}-\frac{1}{2}\Big ), \end{aligned}$$

and the second term

G4 $$\begin{aligned} d (p,r)={\widehat{v}}_{r}\alpha _{p+r}\alpha _{p}. \end{aligned}$$

We then have the following estimates

### Lemma G.1

Let *f*, *d* be as in (G3) and (G4). Then,

G5 $$\begin{aligned} \Big \vert \frac{1}{\vert \Lambda _{\beta } \vert }\sum _{p\in \Lambda _{\beta }^{*}}f(p)-\int _{{\mathbb {R}}^{2}}f(k) \frac{\textrm{d}k}{4\pi ^2} \Big \vert&\le C \rho ^{2}Y^{1/2 + \beta }{\widehat{v}}_0, \end{aligned}$$

and

G6 $$\begin{aligned} \Big \vert \frac{1}{\vert \Lambda _{\beta } \vert ^{2}}\sum _{p,r\ne 0}d(p,r)-\int _{{\mathbb {R}}^{4}}d(p,r) \frac{\textrm{d}p\textrm{d}r}{\left( 4\pi ^2\right) ^{2}} \Big \vert&\le C \rho ^{2}Y^{1/2 + \beta }{\widehat{v}}_0. \end{aligned}$$

### Proof

In order to apply (G2), we start by calculating the gradient

G7 $$\begin{aligned} \partial _{p}f&=2p +\rho _0 \partial _{p}{\widehat{g}}_{p}-2p\frac{\big ( 1+\frac{\rho _0{\widehat{g}}_{p} }{p^{2}}+\frac{\rho _0\partial _{p}{\widehat{g}}_{p} }{2p}\big )}{\sqrt{1+\frac{2\rho _0{\widehat{g}}_{p} }{p^{2}}}}\nonumber \\&\quad +\rho _0 \big (\partial _{p}{\widehat{v}}_{p}-\partial _{p}{\widehat{g}}_{p}\big )\Big (\frac{p^{2}}{2\sqrt{p^{4}+2\rho _0{\widehat{g}}_{p}p^{2}}}-\frac{1}{2}\Big )\nonumber \\&\quad +\rho _0^{2} \left( {\widehat{v}}_{p}-{\widehat{g}}_{p}\right) \frac{{\widehat{g}}_{p} p^{3}-\frac{1}{2}\partial _{p}{\widehat{g}}_{p}p^{4}}{\left( p^{4}+2\rho _0{\widehat{g}}_{p}p^{2}\right) ^{\frac{3}{2}}}\nonumber \\&:= A_p+B_p+C_p. \end{aligned}$$

We will now systematically omit the constants and study separately the cases $p\le \sqrt{2\rho _0 {\widehat{g}}_{0}}$ (case 1 referring to $A_{p}^{1}$) and $p\geqslant \sqrt{2\rho _0 {\widehat{g}}_{0}}$ (case 2 referring to $A_{p}^{2}$). We then get by elementary inequalities

$$\begin{aligned} \left| A_p^{1}\right|&\le (\rho {\widehat{g}}_0)^{1/2},&\left| A_p^{2}\right|&\le \frac{\rho ^2 R {\widehat{g}}_0 {\widehat{g}}_p}{p^2} + \frac{(\rho {\widehat{g}}_{p})^2}{p^3} , \qquad \quad \,\, \\ \left| B_p^{1}\right|&\le \rho R({\widehat{v}}_0-{\widehat{g}}_0),&\left| B_p^{2}\right|&\le \frac{R\rho ^{2} ({\widehat{v}}_0-{\widehat{g}}_{0}){\widehat{g}}_{p}}{p^{2}}, \qquad \qquad \qquad \\ \left| C_p^{1}\right|&\le \frac{\rho ^{1/2}({\widehat{v}}_0-{\widehat{g}}_0)}{{\widehat{g}}_0^{1/2}},&\left| C_p^{2}\right|&\le ({\widehat{v}}_0-\widehat{g_0})\Big ( \frac{\rho ^2 {\widehat{g}}_{0}}{p^{3}}+\frac{R\rho ^{2} {\widehat{g}}_{p}}{p^{2}} \Big ), \end{aligned}$$

where we used $\vert {\widehat{g}}_0-{\widehat{g}}_p\vert \le \vert {\widehat{g}}_0^{3/2}\vert$ for $p\le (\rho {\widehat{g}}_0)^{1/2}$. This way we can use inequality (G2) and the decay of ${\widehat{g}}_{p}$ (3.41) to get

G8 $$\begin{aligned} \frac{1}{L_{\beta }^3} \sum _{p \in \Lambda ^*}\left| \partial _{p}f \right| \textrm{d}p&\le \frac{C}{L_{\beta }} \big ((\rho {\widehat{g}}_0)^{3/2}+ \rho ^{3/2}{\widehat{g}}_0^{1/2}({\widehat{v}}_0-{\widehat{g}}_0) +\rho ^2{\widehat{g}}_0R+R\rho ^2({\widehat{v}}_0-{\widehat{g}}_0)\big )\\ {}&\le C {{\widehat{v}}}_0 \rho ^2 Y^{1/2 + \beta }, \nonumber \end{aligned}$$

where we used (G1), and ${\widehat{g}}_0\le {\widehat{v}}_0$. We use the same method to prove (G6). We have

G9 $$\begin{aligned} \vert \alpha _{p} \vert \le {\left\{ \begin{array}{ll}\frac{\sqrt{\rho {\widehat{g}}_{0} }}{p}, &{} \text { for } p\le \sqrt{\rho {\widehat{g}}_{0} }, \\ \frac{\rho \left| {\widehat{g}}_{p}\right| }{p^{2}}, &{} \text { for } p\ge \sqrt{\rho {\widehat{g}}_{0} }. \end{array}\right. } \end{aligned}$$

We have to calculate

$$\begin{aligned} \partial _{p}\alpha _{p}=-\frac{\rho \partial _{p} {\widehat{g}}_{p}}{2\sqrt{p^{4}+2\rho {\widehat{g}}_{p}p^{2} }}-\frac{\rho {\widehat{g}}_{p}\left( 4p^{3}+4\rho {\widehat{g}}_{p}p+2\rho \partial _{p}{\widehat{g}}_{p}p^{2}\right) }{2({p^{4}+2\rho {\widehat{g}}_{p}p^{2} })^{3/2}}, \end{aligned}$$

yielding

G10 $$\begin{aligned} \vert \partial _{p}\alpha _{p} \vert \le {\left\{ \begin{array}{ll}\frac{\sqrt{\rho {\widehat{g}}_{0}}R}{p}+\left( \rho {\widehat{g}}_{0}\right) ^{-1/2}+\frac{ \sqrt{\rho {\widehat{g}}_{0} }}{p^{2}}+\frac{ \sqrt{\rho {\widehat{g}}_{0} }R}{p}, &{} \text { for }p\le \sqrt{\rho {\widehat{g}}_{0} },\\ \frac{\rho {\widehat{g}}_{p}R}{p^{2}}+\frac{\rho {\widehat{g}}_{p}}{p^{3}}+\frac{\left( \rho {\widehat{g}}_{p}\right) ^{2}}{p^{5}}+\frac{\left( \rho {\widehat{g}}_{p}\right) ^{2}R}{p^{4}}, &{} \text { for } p\ge \sqrt{\rho {\widehat{g}}_{0} }. \end{array}\right. } \end{aligned}$$

The divergence in $p\rightarrow 0$ implies to remove a little box around the point 0

$$\begin{aligned}&\Big \vert \frac{16\pi ^{4}}{L_{\beta }^{4}}\sum _{p,r\ne 0} d (p,r)-\int _{{\mathbb {R}}^{4}} d(p,r )\textrm{d}p\textrm{d}r\Big \vert \\&\le \Big \vert \frac{16\pi ^{4}}{L_{\beta }^{4}}\sum _{p,r\ne 0} d(p,r)-\int _{\big ({\mathbb {R}}^{2}\backslash [-\frac{1}{L},\frac{1}{L}]^{2}\big )^{2}} d(p,r)\textrm{d}p\textrm{d}r\Big \vert \\&\quad + \Big \vert \int _{{\mathbb {R}}^{2}\times [-\frac{1}{L},\frac{1}{L}]^{2}} d(p,r)\textrm{d}p\textrm{d}r\Big \vert + \Big \vert \int _{[-\frac{1}{L},\frac{1}{L}]^{2}\times {\mathbb {R}}^{2}} d(p,r)\textrm{d}p\textrm{d}r\Big \vert . \end{aligned}$$

where the last two terms in the above can be bounded by $\rho ^{2}Y^{1/2+\beta }{\widehat{v}}_{0}$. Finally a direct computation using the decay of ${\widehat{g}}_{p}$, the bounds (4.2), (G9), (G10), and (G2) yields

G11 $$\begin{aligned}&\Big \vert \frac{16\pi ^{4}}{L_{\beta }^{4}}\sum _{p,r\ne 0} d (p,r)-\int _{\big ({\mathbb {R}}^{2}\backslash [-\frac{1}{L},\frac{1}{L}]^{2}\big )^{2}} d(p,r)\textrm{d}p\textrm{d}r\Big \vert \nonumber \\&\le \frac{1}{L_{\beta }^{5}}\sum _{p,r\ne 0}\sup _{\square _p\times \square _r}\left| \nabla _{p,r} d(p,r)\right| \nonumber \\&\le C\frac{{\widehat{v}}_{0}}{L_{\beta }^{5}}\sum _{p\ne 0}\vert \partial _{p}\alpha _{p}\vert \sum _{r\ne 0}\vert \alpha _{r}\vert +C\frac{R{\widehat{v}}_{0}}{L_{\beta }^{5}}\Big (\sum _{r}\vert \alpha _{r}\vert \Big )^{2}\nonumber \\&\le C{\widehat{v}}_0\rho ^2Y^{1/2 + \beta }, \end{aligned}$$

where we used the estimates of Lemma 4.4. This concludes the proof. $\square$

## Appendix H: Fixing Parameters for the Lower Bound

Here we collect all the relations and dependencies of the several parameters involved in the lower bound for the convenience of the reader. Furthermore, we end the section by making an explicit choice that satisfies all the relations. Recall that we have the small parameter

$$\begin{aligned} Y = Y_{\mu } = |\log (\rho _{\mu }a^2)|^{-1}. \end{aligned}$$

We use the following notation throughout the article

H1 $$\begin{aligned} A \ll B \quad \text {if and only if there exist } C, \varepsilon > 0 \;\text { s.t. } A \le CY^{\varepsilon } B. \end{aligned}$$

In the proof of the lower bound, a number of positive parameters are needed. These are the following

H2 $$\begin{aligned} d, s, \varepsilon _T, \varepsilon _K, \varepsilon _N, \varepsilon _{{\mathcal {M}}} \ll 1 \ll {{\mathcal {M}}}, K_{\ell }, K_H,\widetilde{K}_H, K_N, K_B. \end{aligned}$$

These will be chosen below.

Furthermore, there are length scales $\ell _{\delta }$ and *R*. These will be chosen to satisfy

H3 $$\begin{aligned}&R \le \rho _{\mu }^{-1/2},&\text {Condition on the radius of the support }, \end{aligned}$$

H4 $$\begin{aligned}&\ell _{\delta } = \frac{e^{\Gamma }}{2} \rho ^{-1/2}_{\mu } Y^{-1/2},&\text {healing length condition}. \end{aligned}$$

Some first relations between the parameters are

H5 $$\begin{aligned}&d \ll 1 \ll K_{\ell },&\text {sep. of small and large boxes}, \end{aligned}$$

H6 $$\begin{aligned}&d^{-2} \ll K_H \ll \widetilde{K}_H,&\text {sep. of low and high momenta}, \end{aligned}$$

H7 $$\begin{aligned}&d \ll (s K_{\ell })^{-1} \ll 1,&\text {condition for Bog. integral} , \end{aligned}$$

H8 $$\begin{aligned}&d^2 K_{\ell }^4 \ll \varepsilon _T \ll d s K_{\ell },&\text {spectral gap condition} , \end{aligned}$$

H9 $$\begin{aligned}&ds^{-1} \le C,&\text {localization to small boxes}. \end{aligned}$$

The combination of (H6) and (H8) implies the following relations:

H10 $$\begin{aligned} K_\ell \ll K_{\ell }^2 \ll s d^{-1} \ll d^{-1} \ll d^{-2} \ll K_H. \end{aligned}$$

Defining

H11 $$\begin{aligned} \varepsilon _N := K_N^{-1} Y, \qquad \varepsilon _{{\mathcal {M}}} := \frac{{\mathcal {M}}}{\rho _{\mu }\ell ^2}, \end{aligned}$$

we give the following conditions which control the magnitude of the large parameters in terms of *Y*:

H12 $$\begin{aligned}&(dsK_{\ell })^{-1} \ll K_B,&\text {condition errors in small box}, \end{aligned}$$

H13 $$\begin{aligned}&K_B K_{\ell } \widetilde{K}_H K_N^{1/4} \ll Y^{-1/4},&\text {small error in large matrices}, \end{aligned}$$

H14 $$\begin{aligned}&K_{\ell }^{-1} K_N^{1/4} \ll Y^{-1/2},&\text {technical estimate in large matrices} , \end{aligned}$$

H15 $$\begin{aligned}&\widetilde{K}_H K_N^{-1/4} \gg Y^{1/4},&\text {technical estimate in large matrices}, \end{aligned}$$

H16 $$\begin{aligned}&K_{\ell }^2 K_H^2 {\mathcal {M}} \ll Y^{-1},&\text {second localization of 3Q term}, \end{aligned}$$

H17 $$\begin{aligned}&K_B^2 K_{\ell }^2 \ll Y^{-1/4},&\text {number for high momenta} , \end{aligned}$$

H18 $$\begin{aligned}&K_{\ell }^{10} K_H^{-8} d^{-4} \ll Y^{-1},&\text {condition error in } {\mathcal {T}}_1. \end{aligned}$$

Here the magnitude of the small parameters:

H19 $$\begin{aligned}&\varepsilon _R \ll K_B^{-2} K_{\ell }^{-2} |\log Y|^{-1},&\hbox { Condition on}\ \varepsilon _R, \end{aligned}$$

H20 $$\begin{aligned}&\varepsilon _K \ll K_{\ell }^{-2},&\text {error in } {\mathcal {T}}_{2,com}', \end{aligned}$$

H21 $$\begin{aligned}&\varepsilon _K \gg K_{\ell }^4 K_H^{-4} (d^{-2} \varepsilon _{{\mathcal {M}}}^{1/2}+ d^{-4} \varepsilon _{{\mathcal {M}}}),&\text {condition error in } {\mathcal {T}}_1 \text { and }{\mathcal {T}}_2, \end{aligned}$$

H22 $$\begin{aligned}&\varepsilon _{{\mathcal {M}}} \ll d^{8}K_{\ell }^{4}\varepsilon _T^{-2},&\text {condition for error } \delta _1. \end{aligned}$$

H23 $$\begin{aligned}&\varepsilon _N \le \varepsilon _T^{-2} d^4 K_\ell ^4,&\text {bound from Lemma}~9.2. \end{aligned}$$

We use the fundamental property of the system that the number of excitations of our state is relatively small compared to the number of particles (expressed by the condition $\varepsilon _{{\mathcal {M}}} \ll 1$) but still larger that a certain threshold. This property is expressed by the following condition:

H24 $$\begin{aligned} {\mathcal {M}} \gg Y^{-7/8} |\log Y|^{1/4} K_B^{1/2} K_{\ell }^{1/2} K_N^{1/8} \widetilde{K}_H^{1/2}\Vert v\Vert _1^{1/2}. \end{aligned}$$

The following are conditions that impose constraints on the size of *M*, the degree of regularity of the localization function $\chi$:

H25 $$\begin{aligned}&d^{2M-2} \ll Y,&\text {error in localization 3Q}, \end{aligned}$$

H26 $$\begin{aligned}&d^2 K_{\ell }^{4} \ll \varepsilon _T ,&\text {error in localization 3Q}, \end{aligned}$$

H27 $$\begin{aligned}&\varepsilon _N^{3/2} + \Big ( \frac{K_H}{\widetilde{K}_H}\Big )^M + (d^2K_H)^{-2M} \le \varepsilon _{{\mathcal {M}}},&\text {number for high momenta} , \end{aligned}$$

H28 $$\begin{aligned}&(s^{-2} + d^{-2})(s d )^{-2} s^M \le C,&\text {localization to small boxes}. \end{aligned}$$

A choice of parameters, non-optimal in the size of the error produced, fitting the previous conditions, is the following,

H29 $$\begin{aligned} M&= 258,&{\mathcal {M}}&= Y^{-\frac{31}{32}},&\varepsilon _T&= Y^{\frac{1}{512} - \frac{1}{8192}}, \nonumber \\ K_{\ell }&= Y^{-\frac{1}{2048}},&K_H&= Y^{- \frac{1}{128}},&\widetilde{K}_H&= Y^{-\frac{1}{64}},\nonumber \\ d&= Y^{\frac{1}{512}},&K_N&= Y^{-\frac{1}{512}},&s&= Y^{\frac{1}{4096}},\nonumber \\ K_B&= Y^{-\frac{1}{512}},&\varepsilon _K&= Y^{\frac{1}{512}},&\varepsilon _{{\mathcal {M}}}&= Y^{{\frac{1}{32}} + \frac{1}{1024}}. \end{aligned}$$

This choice is not made with any particular view towards optimality.

## Footnotes

1 The power $\frac{1}{8}$ is not optimal but chosen for convenience, in particular to be in agreement with (H24).
